# Design, Synthesis and Molecular Modeling Study of Conjugates of ADP and Morpholino Nucleosides as A Novel Class of Inhibitors of PARP-1, PARP-2 and PARP-3

**DOI:** 10.3390/ijms21010214

**Published:** 2019-12-27

**Authors:** Yuliya V. Sherstyuk, Nikita V. Ivanisenko, Alexandra L. Zakharenko, Maria V. Sukhanova, Roman Y. Peshkov, Ilia V. Eltsov, Mikhail M. Kutuzov, Tatiana A. Kurgina, Ekaterina A. Belousova, Vladimir A. Ivanisenko, Olga I. Lavrik, Vladimir N. Silnikov, Tatyana V. Abramova

**Affiliations:** 1 Institute of Chemical Biology and Fundamental Medicine SB RAS, Lavrent’ev Ave, 8, 630090 Novosibirsk, Russia; yuliya.tarasenko2012@gmail.com (Y.V.S.); sashaz@niboch.nsc.ru (A.L.Z.); mary@niboch.nsc.ru (M.V.S.); kutuzov.mm@mail.ru (M.M.K.); t.a.kurgina@gmail.com (T.A.K.); rina@niboch.nsc.ru (E.A.B.); lavrik@niboch.nsc.ru (O.I.L.); silnik@niboch.nsc.ru (V.N.S.); 2 Federal Research Centre, Institute of Cytology and Genetics SB RAS, Lavrent’ev Ave, 10, 630090 Novosibirsk, Russia; n.ivanisenko@gmail.com (N.V.I.); salix@bionet.nsc.ru (V.A.I.); 3 Novosibirsk State University, Pirogova St., 2, 630090 Novosibirsk, Russia; peshkov@nioch.nsc.ru (R.Y.P.); eiv@fen.nsu.ru (I.V.E.)

**Keywords:** morpholino nucleosides, molecular modeling, NAD+ analogs, DNA repair, PARP

## Abstract

We report on the design, synthesis and molecular modeling study of conjugates of adenosine diphosphate (ADP) and morpholino nucleosides as potential selective inhibitors of poly(ADP-ribose)polymerases-1, 2 and 3. Sixteen dinucleoside pyrophosphates containing natural heterocyclic bases as well as 5-haloganeted pyrimidines, and mimicking a main substrate of these enzymes, nicotinamide adenine dinucleotide (NAD+)-molecule, have been synthesized in a high yield. Morpholino nucleosides have been tethered to the β-phosphate of ADP via a phosphoester or phosphoramide bond. Screening of the inhibiting properties of these derivatives on the autopoly(ADP-ribosyl)ation of PARP-1 and PARP-2 has shown that the effect depends upon the type of nucleobase as well as on the linkage between ADP and morpholino nucleoside. The 5-iodination of uracil and the introduction of the P–N bond in NAD+-mimetics have shown to increase inhibition properties. Structural modeling suggested that the P–N bond can stabilize the pyrophosphate group in active conformation due to the formation of an intramolecular hydrogen bond. The most active NAD+ analog against PARP-1 contained 5-iodouracil 2ʹ-aminomethylmorpholino nucleoside with IC50 126 ± 6 μM, while in the case of PARP-2 it was adenine 2ʹ-aminomethylmorpholino nucleoside (IC50 63 ± 10 μM). In silico analysis revealed that thymine and uracil-based NAD+ analogs were recognized as the NAD+-analog that targets the nicotinamide binding site. On the contrary, the adenine 2ʹ-aminomethylmorpholino nucleoside-based NAD+ analogs were predicted to identify as PAR-analogs that target the acceptor binding site of PARP-2, representing a novel molecular mechanism for selective PARP inhibition. This discovery opens a new avenue for the rational design of PARP-1/2 specific inhibitors.

## 1. Introduction

Poly(ADP-ribose)polymerases (PARP; EC 2.4.2.30) belong to a family of eukaryotic proteins with diverse cellular functions mainly related to DNA repair, maintenance of genomic stability and cell death. The PARP family members [1] perform the catalytic activity of transferring ADP-ribose from the beta-nicotinamide adenine dinucleotide molecule (NAD+) to the acceptors; however, only three of them PARP-1, PARP-2 and PARP-3 possess DNA-dependent (ADP-ribose)transferase activity [1,2]. PARP-1 and PARP-2 catalyze the synthesis of a long stretch of poly(ADP-ribose polymers, whereas PARP-3 is a mono(ADP-ribose)transferase [3]. Acceptors of ADP-ribose, which is synthesized by PARP-1–3, can be both proteins and DNA [4,5,6]. The catalytic domain (CAT) of PARP-1–3 consists of two subdomains, a helical domain (HD) and the (ADP-ribosyl)transferase (ART) domain. This ART domain contains the active site, and is highly conserved in all members of the PARP family [7]. HD is an autoinhibitory domain to the PARP catalytic activity, and is conserved among DNA damage-dependent PARPs including PARP-1, PARP-2 and PARP-3 [7]. Due to a high homology between the catalytic domains of PARP-1 and PARP-2, small molecule inhibitors of PARP-1 usually possess the inhibitory affect to PARP-2 [8].

PARP-3 has two distinct functions: (a) participation in double strand break (DSB) repair pathway(s) and (b) the regulation of mitotic progression [9]. PARP-3 inhibitors sensitize breast cancer cells to vinorelbine, a vinca alkaloid used for the treatment of metastatic breast cancer [10]. Thus, there is a need for selective inhibitors of PARP-3, both for probing the functions of PARP-3 as an enzyme and evaluating its potential as a therapeutic target.

PARPs have been used as pharmacological targets for the treatment of different types of tumors with defects in DNA repair using synthetic lethality. PARP inhibition is considered a promising cancer treatment strategy, and a number of PARP inhibitors are currently undergoing clinical trials; for a review, see [11]. Four PARPi, olaparib, rucaparib, niraparib and talazoparib, have already been approved in the U.S. or Europe, mainly for the treatment of BRCA-deficient cancer [12,13,14].

**Figure 1:**
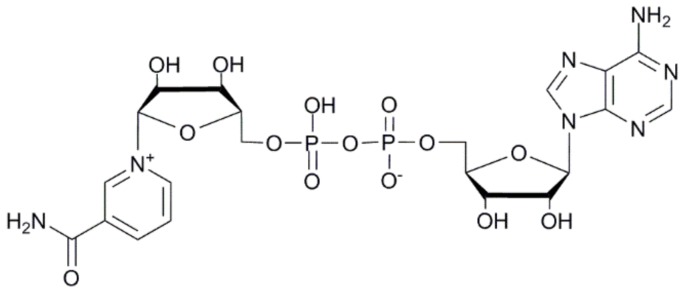
The structure of the beta-oxidized nicotinamide adenine dinucleotide (NAD+)-molecule.

Nicotinamide adenine dinucleotide (Figure 1) plays a key role in such vital processes, as maintaining the integrity of the genome, energy supply, cell death and others [15]. NAD+-metabolizing enzymes are considered by researchers as targets for the treatment of a variety of human diseases, including cancer, multiple sclerosis, neurodegeneration and Huntington’s disease [16]. Nicotinamide, which is one of the structure elements of NAD+ and one of the major products of ADP-ribozylation, inhibits PARP-1 [17]. A large number of existed so far enzyme inhibitors, including some approved by the United States Food and Drug Administration (FDA), have been developed on the basis of nicotinamide [18]. Binding features of nicotinamide analogs include the formation of hydrogens bonds between the lactam or the carboxamide group of the inhibitor with the Gly863 of backbone chain and Ser904 of the side chain, as well as pi-stacking interactions with Tyr907 [19,20,21]. In spite of a great amount of developed analogs of nicotinamide for PAPR-1–3 inhibition, there is an increasing interest in creating novel ones, as evidenced by the growing number of publications in scientific journals [22,23,24]. However, a pharmaceutical usage of existing PARPi is still limited for a number of reasons: the presence of side effects, the appearance of tumor resistance to PARPi and the different efficacy in combination with traditional chemotherapy [14,25]. Therefore, the development of new structural classes of compounds with improved properties, such as selectivity to different types of PARP enzymes, increased inhibitor efficacy, lessened toxicity and higher bioavailability, remains an urgent task.

Natural and modified nucleosides and their phosphorylated derivatives are indispensable tools in searching new anticancer, antiviral and antibacterial drugs [26,27]. However, a small number of studies on nucleoside derivatives as PARPi have been reported in the literature, although some of them exhibit moderate PARP-1 inhibition activity [28]. It has been shown that some thymidine derivatives modified at the 5- and/or 5′-position inhibited PARP-1 more efficiently than 3-aminobenzamide, which is the first generation of the PARP-1 inhibitors and the closest structural analog of nicotinamide [29]. Among phosphorylated adenosines (AMP, ADP, ATP, 3′,5′-cycloAMP, 3′,5′-diphosphoadenosine) a 3′,5′-diphosphoadenosine inhibits PARP-1 most effectively [30]. Ueda’s group show that 5-substituted derivatives of uridine and uracil exhibited an inhibitory activity against PARP-1 too [31]. Additionally, a number of small molecules containing uracil derivatives could be seen as a class of potent PARP-1 inhibitors [32]. There are known PARP-1 inhibitors with a high activity constructed on the base of isoindolinone derivatives and their conjugates with adenosine joined by various aliphatic spacers [33]. This fact may be explained by the dual binding of both parts of the inhibitor, nicotinamide- and adenosine-mimicking. According to the literature data, compounds binding simultaneously into the nicotinamide and adenosine binding sites of the active center of PARP family enzymes could be more selective inhibitors [34].

The NAD+ molecule may be modified at adenine or nicotinamide heterocyclic bases, ribose moieties or the pyrophosphate chain. The investigation of the applicability of fluorescent NAD+ analogs modified at different positions of the adenine moiety has been performed [35]. Recently, fluorescent NAD+ derivatives have been used for real-time cellular imaging of protein poly(ADP-ribosyl)ation [36]. Clickable NAD+ analogs modified at C8 of adenine are promising tools to illuminate the ADP-ribosylated proteome and investigate the molecular mechanisms carried out by individual PARPs upon different cellular signals [37]. Moreover, the well-known non-hydrolyzable NAD+ analog of benzamide adenine dinucleotide (BAD) has been successfully applied to establish the mechanism of PARP-1 interaction with DNA substrate [38]. All these studies demonstrate the power of modified NAD+ derivatives as potential tools for goals of molecular and cellular biology.

Previously, some naturally occurring dinucleoside 5′,5′-pyrophosphates were investigated as potential PARP-1 inhibitors [39]. Diadenosine 5′,5′-tetraphosphate was shown to inhibit the ADP-ribosylation of histone H1 by PARP from the bovine thymus [40]. However, there are published significantly fewer modern investigations devoted to the study of dinucleoside pyrophosphates or NAD+ derivatives as inhibitors of enzymes using NAD+ as a substrate and not as a cofactor. These were found to inhibit NAD kinase [41], bacterial DNA ligases [42] and CD38 NAD glycohydrolase [43]. Recently, we have developed a number of NAD+ mimetics presented by conjugates of ADP and nicotinamide riboside analogs [44,45].

It was shown that some of those provided an inhibitory effect to the autopoly(ADP-ribosyl)ation of PARP-1; IC_50_ of the most effective conjugate consisting of ADP and morpholino-glycine thymine nucleoside [46] was 41.5 ± 3.5 μM [45]. The combination of data from in silico models of PARP-1 with the NAD+-molecule [47] and the crystal structure of the CAT-ΔHD domain of PARP-1 with the non-hydrolyzable NAD+ analog of BAD [38] suggests the requirement of conducting a rational searching for the details of mechanisms of action and specificity of new NAD+ analogs, being the conjugates of ADP and morpholino nucleosides.

**Figure 2:**
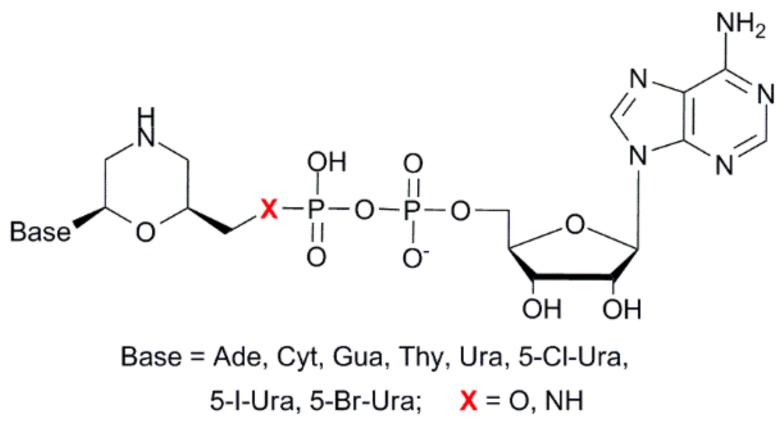
Morpholino nucleoside adenosine dinucleotides (MorXppA).

Morpholino nucleosides are widely used for the synthesis of oligonucleotide mimetics [48]. However, there are only few examples of the utilization of morpholino nucleoside monomers or their phosphorylated derivatives for the goal of molecular biology and biochemistry [49,50]. In the present study we describe the synthesis, inhibition activity and the molecular modeling of the novel conjugates combining from ADP and morpholino nucleosides (morpholino nucleoside adenine dinucleotides, MorXppA), where morpholino nucleosides mimic the nicotinamide riboside fragment of the NAD+ molecule (Figure 2).

## 2. Results and Discussion

### 2.1. Chemistry

#### 2.1.1. Synthesis of ADP Conjugates Containing Phosphoester (P–O) Bond 

**Scheme 1:**
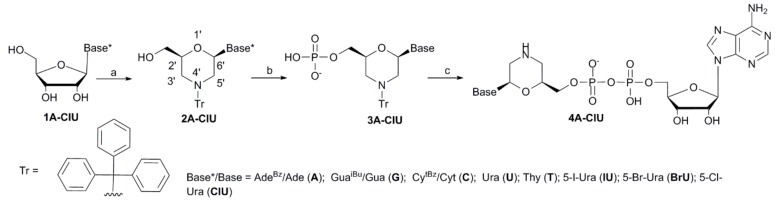
Synthesis of the conjugates of adenosine diphosphate (ADP) with 2′-hydroxymethylmorpholino nucleosides. Reagents and conditions: (a) NaIO_4_, EtOH/H_2_O, 15 min; (NH_4_)_2_B_4_O_7_·4H_2_O, Et_3_N, 1.5 h; NaBH_3_CN, 40 min; trifluoroacetic acid (TFA), pH 3–4, 1 h; TrCl, Et_3_N, dimethylformamide (DMF), 3 h; yield 60%–70%; (b) POCl_3_, Py, –15 °C, 15 min; 1 M triethylammonium bicarbonate (TEAB), yield 75%–90%; (c) Ph_3_P/(PyS)_2_, MeIm, 1,3-dimethyl-2-imidazolidinone (DMI); n-Bu_3_NH^+^ salt of AMP; conc. aq. NH_3_ for compounds **4A**,**G**,**C**; 80% aq. AcOH (v/v); yield 70%–80%.

Recently we have proposed a versatile method for the synthesis of ADP conjugates functionalized at the terminal phosphate [44,45]. In that study, we developed an effective protocol for the coupling of two monoester phosphate derivatives under the action of the redox coupling pair triphenylphosphine/2,2′-dipyridyldisulfide (Ph_3_P/(PyS)_2_) in the presence of 1-methylimidazole (MeIm). We applied the same strategy for the synthesis of the sought-for pyrophosphates MorXppA **4**, where X represents an oxygen atom (Figure 2, Scheme 1).

At the first step, we provided preparation of *N*-Tr-protected morpholino nucleosides. The standard protocol for nucleosides with natural heterocyclic bases was Summerton’s procedure [51]. Recently we have published a detailed protocol for the synthesis of 5-iodopyrimidine morpholino nucleosides based on this method [52]. Continuing our research, we have succeeded in obtaining *N*-Tr-protected morpholino nucleosides **2** from acyl-*N*-base-protected (when necessary) ribonucleosides **1** without chromatographic purification in an overall yield of 60%–70%. 

To obtain monophosphate **3U**, we treated protected morpholino nucleoside **2U** with 2 eq of POCl_3_ in dry pyridine (Py) under cooling in ice bath according to [53]. Unfortunately, the product **3U** was obtained in a yield of less than 50% after reverse phase chromatography (RPC). We varied the excess of POCl_3_ (2–4 eq), the temperature (0 –−15 °С) and reaction time (5–30 min) and found optimal conditions for the monophosphorylation of nucleosides **2** (Scheme 1). The monophoshates **3A,G,C,IU** were obtained without chromatographic purification in a yield of over 90%; and monophoshates **3U,T,BrU,ClU** in a yield of 75%–80% after RPC.

To prepare ADP conjugates **4**, we activated a phosphate group in derivatives **3** by Ph_3_P/(PyS)_2_ in a presence of MeIm in dry 1,3-dimethyl-2-imidazolidinone (DMI) at room temperature for 15–20 min according to [44]. Further coupling of phosphoro(*N*-methyl)imidazolidate of morpholino nucleoside with AMP was carried out in situ in dry DMI for 1 h. After chromatographic purification and deblocation procedures, we obtained target conjugates **4** in an overall yield of 70%–80% (Scheme 1).

#### 2.1.2. Synthesis of ADP Conjugates Containing Phosphoramide (P–N) Bond

**Scheme 2:**
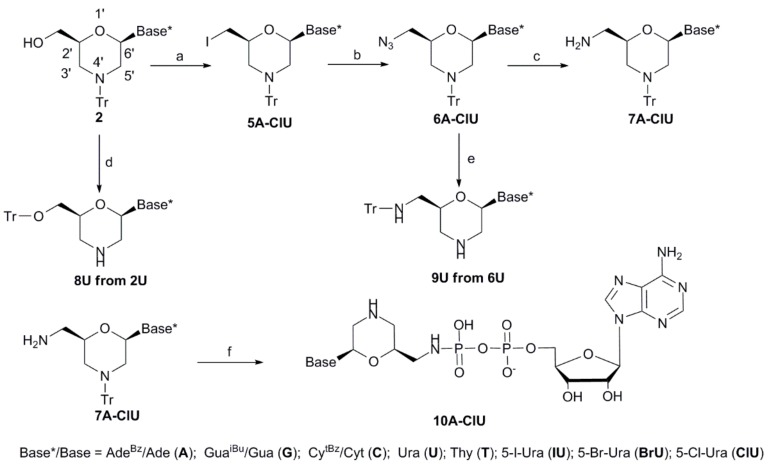
Synthesis of the conjugates of ADP with 2′-aminomethylmorpholino nucleosides. Reagents and conditions: (a) Ph_3_P, Im, DCE, I_2_, 0 °C → rt, 5 h; (b) NaN_3_, DMF, 12 h; (c) H_2_, 10% Pd/C, MeOH; (d) Ph_3_P, CBr_4_, DMI; (e) Ph_3_P (2 eq), Py; conc. aq. NH_3_; (f) ADP n-Bu_3_N salt, Ph_3_P, (PyS)_2_, MeIm, DMI; conc. aq. NH_3_ for compounds **10A**,**G**,**C**; 80% aq. AcOH (*v*/*v*).

For the synthesis of these conjugates we prepared 2′-aminomethylmorpholino nucleosides **7** (Scheme 2).

An azido group is a convenient source for amino functions. In the literature, several methods for obtaining 5′-azidoribonucleosides containing natural heterocyclic bases are known. Methods are based on conversion of the hydroxyl group to halogen [54,55,56,57,58,59], sulfonate [60,61,62] or phosphate ester group [63] under anhydrous conditions. The further reaction with lithium or sodium azide leads to the formation of 5′-azidoribosyl derivatives in an overall yield of 14%–92%. Similar approaches were proposed for the synthesis of 2′-aminomethylmorpholino nucleosides containing natural heterocyclic bases. One of them is based on the one-pot synthesis of 2′-azidomethylmorpholino nucleosides under the treatment of compounds **2A,G,C,U,T** with Ph_3_P/CBrCl_3_/NaN_3_ in dry dimethylformamide (DMF). After reduction of the N_3_-group and a full deblocation procedure, 2′-aminomethylmorpholino nucleosides were obtained in an overall yield of 39%–64% [46,64]. 

Another procedure is based on obtaining 2′-sulfonylmethylmorpholino nucleosides by the treatment of compounds **2A,G,C,T** with mesyl chloride in dry pyridine and a further substitution of mesyl with an azide anion. After the reduction of the N_3_-group 2′-aminomethylmorpholino nucleosides were obtained in an overall yield of 34%–45% [65].

A literature search for the synthesis of 5′-azidonucleosides containing 5-halosubstituted pyrimidines provided only a few reports. Most of suggested methods are based on the ribosylation of modified heterocyclic bases with 5′-azidosugar derivatives [66,67,68]. In 1976, Prusoff et al. reported a synthesis of the 5′-azido-5-halouridine by substitution of the sulfonate group of 5′-tosyl-5-halouridine with the azide one in a yield of 33%–73% [69]. The attempts to reduce the N_3_-group of 5′-azido-5-chlorouridine under the action of H_2_/PtO_2_ resulted in the dehalogenation of 5-chlorouridine. 5′-Amino-5-halouridine was obtained by the halogenation of 5′-aminouridine with Hg(OAc)_2_ and *N*-bromosuccinimide (NBS) or I_2_. Later, in a search for obtaining 5′-aminoarabinonucleosides containing 5-bromo- or 5-iodouracil, Prusoff et al. found that the halogenation of 5′-aminoarabinouridine by NBS or *N*-iodosuccinimide (NIS) did not lead to the formation of the target compounds [70]. In addition, attempts to replace a sulfonate group of 5′-tosyl-5-iodoarabinouridine with the azido one under the action of lithium azide failed. The only successful approach was the halogenation of 5′-azidoarabinouridine by NBS or NIS in a yield of 94% (for the Br-derivative) and 52% (for the I-derivative), and then the reduction of this azido function in the presence of Ph_3_P in Py in a yield of 56% (for the Br-derivative) and 41% (for the I-derivative). The authors noted that the reduction of the azido group by NaBH_4_ or H_2_/Pd/C led to dehalogenation of the 5-bromo- or 5-iodouracil derivatives.

We did not find any data concerning the synthesis of 2′-aminomethylmorpholino nucleosides **7IU, BrU, ClU** containing 5-halosubstituited uracil or any modified heterocyclic base in the literature. Thus, at first, we tried to synthesize the azido derivative **6BrU** following the one-pot procedure using Ph_3_P/CBrCl_3_/NaN_3_ in dry DMF according to the published method [46]. However, our attempts failed. Under using DMI instead of DMF and changing the order of the reagent’s addition [58], we obtained azido derivative **6BrU** in a yield of 24% (Scheme 2). Since the one-pot method did not provide a satisfactory yield of the target compound, we applied a “step-by-step” approach and tested the method on the compound **2U**. To our surprise, after treatment of the compound **2U** with Ph_3_P/CBrCl_3_ or Ph_3_P/CBr_4_ in dry MeCN or DMI we observed a migration of the Tr-group from the 4′-*N* position to 2′-OH-methyl one giving *O*-tritylated morpholino nucleoside **8U**. Then, we tried to convert the hydroxyl group of morpholino nucleosides **2** into an iodine function and applied the I_2_/Ph_3_P/Im reagent [71]. The reaction of 2′-hydroxymethylmorpholino nucleosides **2** with this reagent in a variety of solvents (DMI, *N*-methyl-2-pyrrolidone, DCE, DCM, MeCN, THF) leads to a quantitative conversion of the compounds **2** into the compounds **5** in 5 h according to HPLC analysis. Further treatment of the reaction mixture with aq. sol. of NaHSO_3_ and NaHCO_3_ and chromatographic purification results in iodine derivatives **5** in a yield of 85%–93%. When the reaction mixture was treated with aq. sol. of NaHCO_3_ only a partial detritylation of the target products were observed. Reaction of the hydroxyl group substitution with iodine also proceeds when pyridine was used instead of imidazole in the reagent mixture I_2_/Ph_3_P/Im [55,71,72]. However, when the reaction of **2T** with I_2_/Ph_3_P/Py was carried out in DCE, we observed the formation of two products **5T** and **8T** in a ratio of 1:2.

The reaction of the compounds **5** with sodium azide in DMF afforded morpholino nucleosides **6** in a quantitative yield. We tried to reduce the azido to an amino group by Pd/C-catalyzed hydrogenation and by Ph_3_P/Py treatment. However, in a case of treatment of the compound **6U** with Ph_3_P in dry Py we observed the reduction of the azido group and the migration of the trityl moiety to the 2′-aminomethyl position of the morpholine ring resulted in the compound **9U**. The successful reduction of azido derivatives **6** was carried out by Pd/C-catalyzed hydrogenation. In this way, 2′-aminomethylmorpholino nucleosides **7** were obtained in a nearly quantitative yield by “step-by-step” approach. It should be noted that under reducing the 2′-azidmethylmorpholino nucleosides containing 5-halo-substituited uracil **6IU, BrU, ClU** by both Pd/C-catalyzed hydrogenation and Ph_3_P/pyridine, we did not observe any dehalogenation of the heterocyclic base [52].

The synthesis of ADP conjugates **10** containing the phosphoramide (P–N) bond was carried out by Ph_3_P/(PyS)_2_/MeIm activation of the β-phosphate of ADP and further coupling with the 2′-aminomethylmorpholino nucleosides **7** (Scheme 2). Previously, the approach consisting in the Ph_3_P/(PyS)_2_/MeIm activation of terminal phosphate in oligonucleotides to obtain phosphoroamide derivatives was widely used [73]. After chromatographic purification and a deblocation procedure ADP conjugates **10** were obtained in an overall yield of 70%–75%.

### 2.2. PARP-1, PARP-2 and PARP-3 Inhibition

#### 2.2.1. Inhibition of PARP-1 and PARP-2 by ADP Conjugates

The effects of 16 compounds MorXppA **4** and **10** on the autopoly(ADP-ribosyl)ation of PARP-1 and PARP-2 were studied. The inhibitory properties of NAD+ analogs are summarized in Table 1. As it can be seen from the data presented in Table 1, phosphodiester dinucleotide pyrophosphates **4** containing morpholino nucleosides conjugated to ADP through the P–O bond have a little effect on the activity of PARP-1 and PARP-2 (residual enzyme activity in the presence of 1 mM compounds is 40%–90%). Among compounds **4** an inhibition efficiency of PARP-1 increases depending on the heterocyclic base of morpholino nucleoside in the order Cyt < Ura < Ade < 5-Cl-Ura < Gua < Thy < 5-Br-Ura < 5-I-Ura. In a case of PARP-2, this order has been changed, (Cyt, Ura) < 5-Cl-Ura < Gua < (5-Br-Ura, Thy) < Ade < < 5-I-Ura for conjugates **4.** The only compound that has a significant effect on the activity of both enzymes is the 5-iodouracil derivative **4IU**.

Analogs of these compounds with the P–N bond **10** generally have a more pronounced effect on the activity of PARP-1 and PARP-2. The residual activity of the enzymes in 1 mM concentration of compounds is from 10% to 90%. The 5-iodouracil derivative **10IU** is also active, with the IC_50_ value for PARP-1 being equal to 126 μM. Close to **10IU** in effectiveness of inhibition of both enzymes is the thymidine derivative **10T** with IC_50_ value for PARP-1 220 μM. Activity of conjugates **10** with the P–N bond against PARP-1 changes as Ura < Cyt < Gua < 5-Br-Ura < 5-Cl-Ura < Ade < <Thy < <5-I-Ura depending on the heterocyclic base. In a case of PARP-2, this order has been changed, 5-Cl-Ura < (Cyt, Ura) < (5-Br-Ura, Gua) < Thy,5-I-Ura < <Ade for conjugates **10**. 

The most active compound in the series **4** against both enzymes is the 5-I-Ura-containing analog **4IU** with IC_50_ 255 ± 5 μM for PARP-1 and 160 ± 10 μM for PARP-2. However, compounds **10** containing a P–N bond generally have a more pronounced effect on the activity of both enzymes. 

It is interesting that the most active NAD+ mimetic among the **10A,G,C,U,T,IU** series is different for PARP-1 and PARP-2 in contrast to **4** series: for PARP-1 it is compound **10IU** with IC_50_ 126 ± 6 μM, for PARP-2 it is compound **10A** with IC_50_ 63 ± 10 μM. The influence of the 2′-aminomethyl group of morpholino nucleosides is more pronounced in the inhibition of PARP-2 than of PARP-1 (see adenine- and thymine-containing compounds **4A** and **10A**, and **4T** and **10T**). On the other hand, compounds **4IU** and **10IU** inhibit PARP-1 almost equally independently of the P–O or P–N bond presence.

#### 2.2.2. Inhibition of PARP-1 and PARP-2 by Natural and Modified Nucleosides

Morpholino nucleosides and ribonucleosides were used to study the contribution of the morpholine cycle to enzyme inhibition. The inhibitory properties of these compounds are shown in Table 2. Natural ribonucleosides were weakly active as inhibitors except **rThd** (IC_50_ values 277 μМ for PARP-1 and 374 μМ for PARP-2), while iodine- and bromine-containing derivatives inhibited both enzymes. The 5-I-uridine was five times more effective than 5-Br-uridine for both enzymes. The ratios of IC_50_ values for PARP-1:PARP-2 are also approximately 1:1. The 5-Cl-uridine had a little effect on the activity of PARP-1, while PARP-2 was inhibited with an IC_50_ value of 196 μM. Basically, the replacement of ribose by the morpholine cycle leads to an increase in the inhibitory ability of the compounds.

Among the morpholino nucleosides, Ade- and Ura-containing derivatives (**11A** and **11U**, respectively, Table 2) were the least active against both enzymes. For PARP-1, the inhibition activity of nucleosides increases depending on the heterocyclic base of the morpholino nucleoside in the order Ade, Ura < (Cyt, 5-Cl-Ura) < (Gua, Thy) < (Ura, 5-Br-Ura); for PARP-2 the order is (Ade, Ura) < Cyt < Gua < 5-Cl-Ura < 5-Br-Ura < Thy < 5-I-Ura.

Thus, morpholino nucleosides proved to be more effective inhibitors of both enzymes than dinucleotides. Regardless of the other part of the molecule, i.e., nucleobase, linker type, the presence of the adenosine part of the molecule, the most effective inhibitors contained 5-iodouracil. The 5-iodouracil derivatives are characterized by the ratio of IC_50_ values for PARP-1 and PARP-2 as 1:1. Adenine dinucleotide **10A** with the P–N bond stands apart inhibiting the activity of PARP-2 but not PARP-1, with the efficacy comparable to 5-iodoururacil containing derivatives. Adenine containing dinucleotide **4A** with the P–O bond is a little active against both enzymes, whereas adenine mononucleosides are completely inactive.

#### 2.2.3. Determination of the Type of Inhibition

We conducted kinetic studies of the inhibition of PARP-1 by the most effective inhibitors — NAD+ mimetics. For studies we selected compounds **4IU, 10IU, 11IU**. For comparison, kinetic parameters of the autopoly(ADP-ribosyl)ation in the presence of compound **10A**, which more effectively inhibits PARP-2, were determined. The 3-aminobenzamide (**3-AB**) is a well known PARP inhibitor [74,75] and has been taken for comparison.

The type of inhibition was determined based on at what stage of the reaction the inhibitor binds to the enzyme (before or after the substrate binding) and where it binds (at the substrate binding site or elsewhere). There are four types of inhibition: competitive, whereby an inhibitor binding occurs at the substrate-binding site; noncompetitive, where an inhibitor binds at the allosteric center; mixed with inhibitor binding at both the active and allosteric center; or uncompetitive under an inhibitor binding to the enzyme-substrate complex. Determination of the type of inhibition is necessary to confirm the computer simulation data and to plan further modifications of the inhibitor structure. The type of inhibition can be determined from the plots of the reaction parameters, such as maximum rates, Vmax and the apparent Michaelis constant, Km, values, versus inhibitor concentration [76]. So, under conditions of competitive inhibition, the Vmax value does not change, and the Km value does grow. Noncompetitive inhibition leads to a decrease of Vmax and retention of the Km values. Mixed inhibition is a superposition of the first two, and is characterized by a decrease in the reaction rate and an increase in Km values. In the case of uncompetitive inhibition, both values increase.

**Figure 3:**
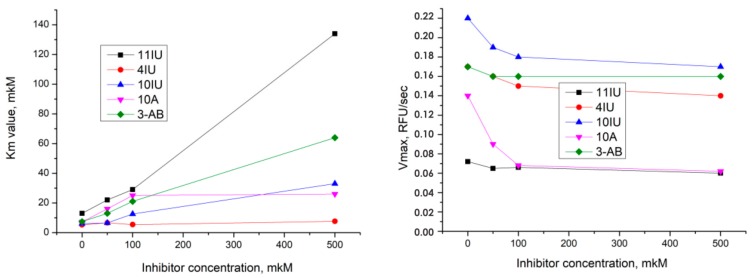
Graphs of Km (left) and Vmax (right) values versus inhibitor concentration. **3-AB**, a commercially-available PARP-1 inhibitor [75], was used as a positive control.

To determine the type of inhibition of the studied compounds, the activity of PARP-1 was measured at presence of three different inhibitor concentrations at nine NAD+ substrate concentrations by the fluorescence anisotropy technique [77]. Based on the obtained data, dependence graphs of the initial reaction rate on the substrate concentration were constructed, and reaction parameters such as maximum reaction rate (Vmax) and apparent Michaelis constant (Km) values were found (Tables S3 and S4, Supporting). To determine the type of inhibition, we plotted the dependence graph of Vmax and Km values on the inhibitor concentration using OriginPro 8.6.0 software. (Figure 3). As can be seen from Table S3 and Figure 3, the Km values have been grown, and the maximum reaction rate remains unchanged; that is, compounds **10IU** and **11IU** are competitive inhibitors for PARP-1 catalyzed reaction. Therefore, the results of modeling of the binding type of these compounds in the enzyme’s active center are legitimate. Compound **4IU** may be characterized as a mixed inhibitor, although its influence on Km and Vmax values is weak. Compound **10A** turned out to be a mixed inhibitor because the Km values grow and the Vmax values decreased. These results indicate that compounds containing adenine and iodouracil differ in their binding mode. 

#### 2.2.4. PARP-3 Inhibition

**Figure 4:**
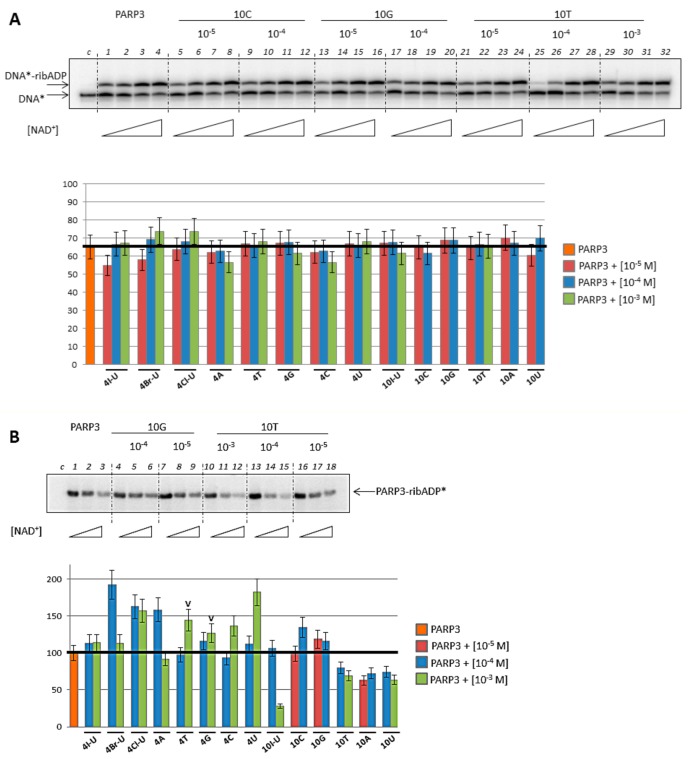
Basic activity of PARP-3 in DNA (**A**) and protein ADP-ribosylation (**B**) and the influence of the inhibitors on these reactions. (**A**) Activity of PARP-3 on [^32^P]-labeled one-window gapped DNA substrate in the absence (lanes 1–4) or presence of inhibitors in the different concentrations (lanes 5–32) on the upper panel. The reactions were performed using increasing concentration of NAD+. Lane c corresponds to initial electrophoretic mobility of the DNA substrate. The chart on the bottom panel is reflected of the reaction yield of the ADP-ribosylated DNA in percentage terms. (**B**) Activity of PARP-3 on gap1 DNA substrate in the absence (lanes 1–3) or presence of inhibitors in the different concentrations (lanes 4–18) on the upper panel. The reactions were performed using increasing concentration of NAD+ in the presence of [^32^P]-labeled NAD+. Lane c corresponds to reaction mixture without PARP-3. The chart on the bottom panel is reflected of the reaction yield of the ADP-ribosylated PARP-3 in the presence of inhibitor normalized on the yield of the autoribosylation of PARP-3 in percentage terms. The ticks on the chart mark the bars with the 0.5 mM of inhibitor in the experiment.

The effects of 16 compounds MorXppA **4** and **10** on the auto(ADP-ribosyl)ation of PARP-3 and ADP-ribosylation of DNA by PARP-3 were studied. The results are summarized in Figure 4. None of the compounds affected DNA modification (Figure 4A). In the auto(ADP-ribosyl)ation reaction, also none of the compounds showed significant inhibitory activity except **10IU** used in 1 mM concentration (Figure 4B).

### 2.3. Structural Studies

#### 2.3.1. Binding Modes of Guanine and 2,4-dioxopyrimidine Containing NAD+ Analogs to PARP-1 and PARP-2

**Figure 5:**
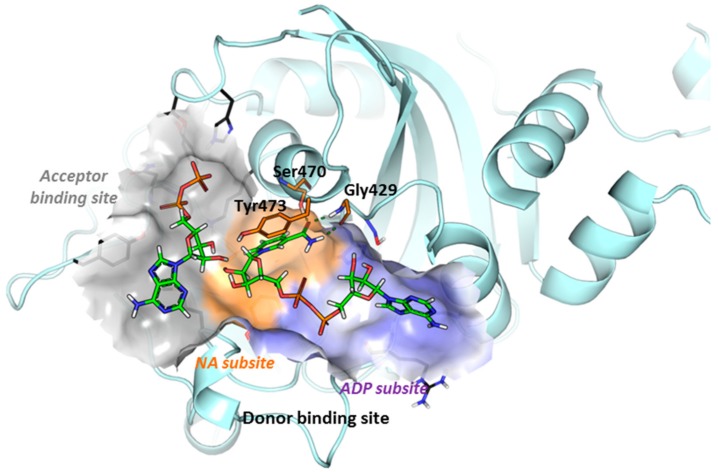
Structural model of the PARP-2 catalytic domain in complex with NAD+ at the donor binding site and ADP fragment at the acceptor binding site. The molecular surface illustrates ADP binding subsites of acceptor substrate (gray color), nicotinamide riboside fragment of donor NAD+ substrate (orange color) and the ADP fragment of donor NAD+ substrate (blue color). HD domain is not shown for simplicity. Substrates are shown in green color.

**Figure 6:**
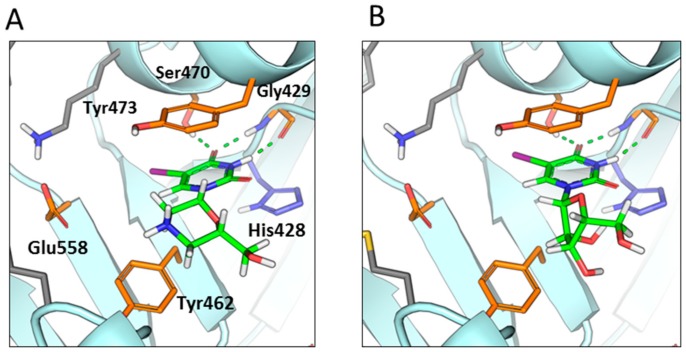
Predicted binding poses of **11IU** (**A**) and **5-I-Urd** (**B**) bound to the NA binding site of the PARP-2 catalytic domain. Hydrogen bonds are depicted as green dashed lines. Small molecules are shown in green color.

Based on observed activity of 2,4-dihydroxypyrimidine nucleosides (including thymine and uracil derivatives) against PARP-1/2, one can assume that they can form canonical hydrogen bonds with an active site of PARP-1 and PARP-2, similar to those observed at the binding with natural substrate NAD+ (Figure 5). We carried out a molecular docking study to assess the efficiency of morpholino and ribonucleosides binding with the donor binding site of NAD+ (Figure 6A). 

It includes the formation of hydrogen bonds with Gly863/Ser904 of PARP-1 or Gly429/Ser470 of PARP-2 and pi-stacking interaction with Tyr907 and Tyr473 of PARP-1 and PARP-2 correspondingly. The 5-halogen derivatives **11IU** (XP score −8.25 and −9.857 for PARP-1 and PARP-2 respectively (Figure 6A)) and **5-I-Urd** (XP score −8.328 and −8.902 for PARP-1 and PARP-2 respectively (Figure 6B)) were predicted to be among the top ranking binding scores due to enhanced hydrophobic interactions of iodine in the nicotinamide binding pocket, which is in agreement with experimental data. 

Besides uracil-containing derivatives only guanine-containing compounds **Guo** (XP score −8.387 and −9.582 for PARP-1 and PARP-2 respectively) and **11G** (XP score −9.615 and −9.207 for PARP-1 and PARP-2 respectively) were able to form canonical hydrogen bonds in the nicotinamide binding subpocket of PARP-1/2, and have shown relatively high scoring function values. At the same time the presence of unsatisfied hydrogen bond acceptor groups of guanine residue located in the hydrophobic region of the nicotinamide binding subpocket likely leads to decreased binding affinity, observed in vitro. Lack of unsatisfied hydrogen bond groups in the hydrophobic nicotine amide subpocket is necessary according to analysis of all known co-crystallized PARP-1/2 inhibitors. Lack of this penalty likely leads to an overestimation of the scoring function value in case of PARP-1/2. 

**11A**, **Ado**, **11C** and **Cyd** were unable to form canonical hydrogen bonds in the nicotine amide binding pocket with PARP1/2, and in agreement with experimental data have shown lowest scoring function values with dG > −7.7. 

We found out that morpholino nucleoside derivatives in general have stronger inhibition activity to PARP-1/2 in comparison with corresponding ribonucleosides (Table 2). We could not correlate these observations with XP scoring functional values, but according to molecular docking predictions, the ribose fragment of ribonucleosides has a different localization in the PARP-1/2 catalytic site in comparison with the corresponding ribose fragment of the NAD+ substrate (Figure 5, Figure 6B). Presumably, it leads to reduced hydrophobic interactions in the subpocket formed by Tyr473, while the morpholine fragment occupies the binding site volume more efficiently (Figure 6A). 

Overall, the conducted analysis shows that uracil-containing compounds, including thymine, can be used for the development of efficient PARP-1/2 inhibitors. 

**Figure 7:**
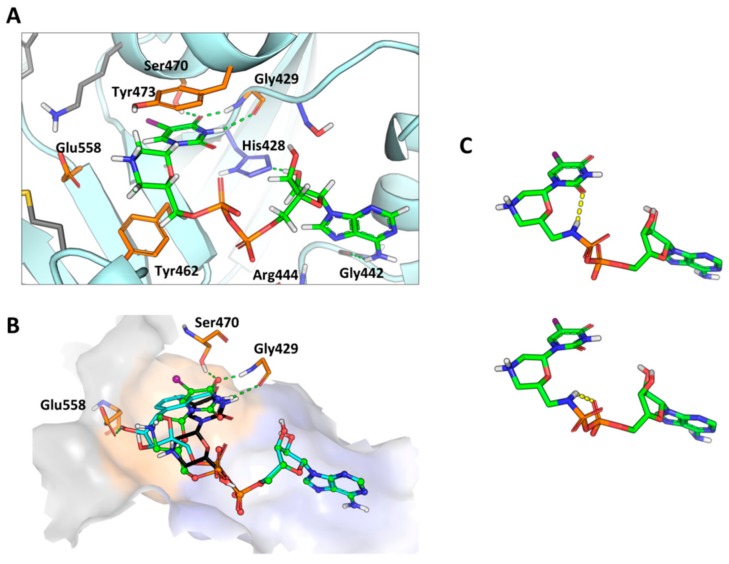
Predicted binding mode of **10IU** with the donor binding site of the PARP-2 catalytic domain. (**A**) The close-view of the donor binding site. (**B**) Structural alignment of the binding poses of **10IU** (green color) with NAD+ (cyan color) and **5-I-Urd** (black color). (**C**) Representative conformations of **10IU** from the molecular dynamics trajectory, the conformation with the intramolecular hydrogen bond with the non-bridging α-phosphate oxygen of ADP (bottom) and the conformation with the hydrogen bond with carbonyl oxygen of the heterocyclic base of the modified nucleoside (top) are shown. The molecular surface of the binding site is shown. Hydrogen bonds are depicted as green dashed lines.

On the next step, we carried out a prediction of the binding modes of uracil-based NAD+ analogs synthesized in the current work, including **10T, 10IU, 4T** and **4IU**. We could not obtain the binding pose of the compound mimicking the natural substrate NAD+ binding pose using a molecular docking tool, expecting that uracil-containing derivatives would be bound to the nicotinamide binding subpocket, and adenosine would be bound to the adenine subpocket simultaneously (Figure 7A). One of the reasons could be due to the low propensity of the compound to fit the donor binding site or to the high number of conformational degrees of freedom of compounds. Therefore, we applied an alternative approach by combining the results of **5-I-Urd** and **11IU** molecular docking results and predicted the localization of the ADP fragment of the donor NAD+ nucleoside fragment based on the crystal structure of PARP-1 bound to BAD [38]. The predicted fragments were manually linked to each other, and molecular mechanics minimization using Schrödinger software with constraints on the heavy atoms of the PARP-1/2 and AMP fragment of compounds was carried out. By applying this protocol, we succeeded to obtain the molecular pose of compound **10IU** bounded to nicotinamide and ADP binding sites simultaneously in a similar way as the NAD+ substrate (Figure 7B). The visual inspection of the obtained binding poses shows the close proximity of the negatively-charged phosphate group and carbonyl oxygen of the pyrimidine group, potentially leading to a reduction of binding affinity. Another feature is the distant localization of the positively-charged group of the morpholine fragment from negatively-charged side chain of Glu558 of PARP-2. This side chain is involved in hydrogen bond formation with the ribose group of NAD+, while lack of this interaction can potentially lead to reduction of the binding affinity of **10IU.** The obtained results can explain why addition of the ADP group to **11IU** and **5-I-Urd** does not enhance its activity, and it also provides the basis for further compound optimization. 

As can be seen from Table 1, a substitution of P–O to P–N bond leads to an enhanced activity of the most tested NAD+ analogs. These effects can be explained by the formation of an intramolecular hydrogen bond that stabilizes pyrophosphate group conformation. To further study this phenomenon we carried out 100 ns molecular dynamics simulation of the complex of PARP-1 with the 5-I-uracil-containing ADP conjugate **10IU** with positional restraints on the heavy atoms of the adenine-ribose fragment of the compound and the CA-atoms of the protein. In this obtained MD trajectory, two types of hydrogen bonds of the P–NH group were observed: the hydrogen bond with the non-bridging α-phosphate oxygen of ADP (Figure 7C) and the hydrogen bond with the carbonyl oxygen of heterocyclic base of the modified nucleoside (Figure 7C). It has to be noted that in the first case the stabilization is independent from a type of heterocyclic base of morpholino nucleosides and is expected to be observed in all studied NAD+ analogs that is in agreement with in vitro data. 

#### 2.3.2. Micromolar Inhibition of PARP-2 Activity by Targeting Acceptor Substrate Binding Site

As mentioned above, the dependence of compounds **4** efficacy on the heterocyclic base looks like Cyt < Ura < Ade < 5-Cl-Ura < Gua < Thy < 5-Br-Ura < 5-I-Ura for PARP-1 and (Cyt, Ura) < 5-Cl-Ura < Gua < (5-Br-Ura, Thy) < Ade < < 5-I-Ura for PARP-2. The main discrepancy between PARP-1 and PARP-2 series is the position of the Ade-containing conjugate **4A**: this compound does not influence on the activity of PARP-1, but weakly inhibits PARP-2. For P–N-containing compounds **10**, these series turn into Ura < Cyt < Gua < Ade << Thy << 5-I-Ura for PARP-1 and (Cyt, Ura) < Gua < Thy < 5-I-Ura << Ade for PARP-2. We noticed that these series were almost the same for PARP-1, but they had significant difference for PARP-2 in the position of the Ade-containing inhibitor. Namely, compound **10A** weakly inhibits PARP-1 (similar to its P–O analog **4A**), but proves to be the most effective for PARP-2. These results are counterintuitive due to the low activity of **10A** and **Ado** compounds predicted in silico and observed in vitro. Indeed, **10A** and **Ado** cannot be efficiently bound by the nicotinamide subpocket according to the molecular docking predictions. In particular, this is due to a lack of pharmacophores necessary for the formation of critical hydrogen bonds with the NA site, including lactam or carboxamide groups [20]. 

Therefore, first we assumed that the adenine-MorXpp fragment of **10A** can compete for binding with the ADP fragment of NAD+. It has to be noted that NAD+ can be bound to PARP-1/2 at least at two binding sites of PARP-1/2, including donor (NAD+) and acceptor (PAR) binding ones (Figure 5). On the first step we evaluated the efficiency of adenine-MorXpp binding to the donor ADP binding site using molecular docking simulation. Molecular docking predictions show that adenine-MorXpp is not expected to bind more efficiently than Ado-5′-pp or NAD+ in the donor binding site, based both on bindings score values and visual inspection of high-ranked binding poses. Visual inspection indicates that the morpholine group does not form hydrogen bonds with residues His862 and Ser864 of PARP-1 or His428 and Ser430 of PARP-2, while the hydroxyl group of ribose in Ado-5′-pp does. The conducted analysis strongly suggests that **10A** cannot bind to donor NA and ADP binding sites. 

**Figure 8:**
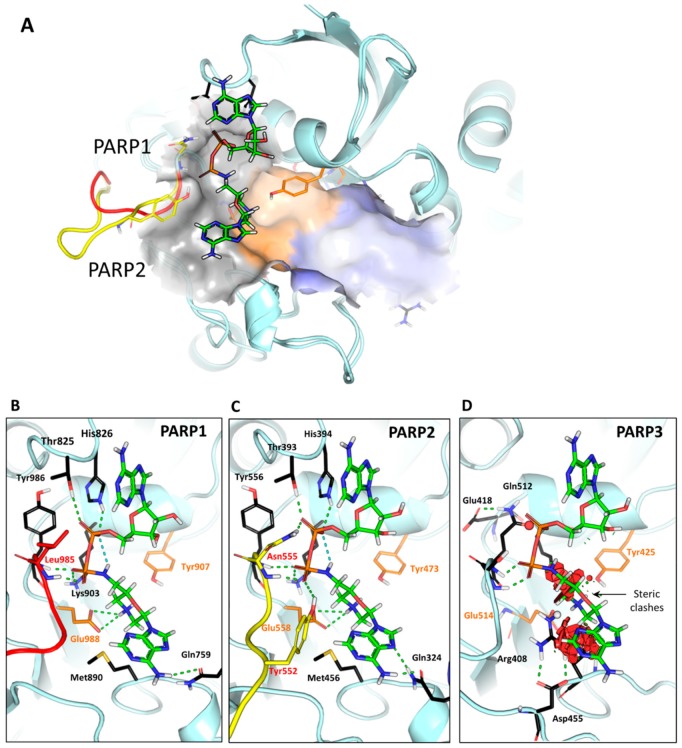
Predicted binding pose of **10A** with the acceptor binding site of the PARP-1/2 catalytic domain. (**A**) The structural alignment of PARP-1 and PARP-2. Variable loops are indicated in red and yellow colors for PARP-1 and PARP-2, respectively. Detailed view of **10A** interaction with PARP-1 (**B**) and PARP-2 (**C**) is shown. (**D**) PARP-3 acceptor binding site with superimposed binding pose of **10A** from PARP-2/**10A** complex**.** Steric clashes are shown with red disks. Unsatisfied hydrogen bond donor and acceptor atoms of the PARP-3 acceptor binding site hindered by ligand are shown as spheres. Hydrogen bonds are depicted as dashed lines. HD domain is not shown for simplicity.

On the next step we evaluated the hypothesis whether **10A** and **4A** can compete for binding with the acceptor substrate. We found that the adenine-MorXpp fragment of these compounds can efficiently mimic the interactions of the ADP fragment of the acceptor substrate. The latter was predicted based on the crystal structure of PARP from *Gallus gallus* (red junglefowl, PDB identifier 1A26; [78]). Moreover, the NH^+^ moiety of the morpholine ring of the **10A** compound can form a salt bridge with Glu988 or Glu558 residues of PARP-1 or PARP-2, respectively. In this case, the morpholine ring can mimic an interaction of the 2′–OH group of adenosine and expected to further enhance binding affinity in comparison with natural acceptor substrate (Figure 8A). Visual inspection of binding poses shows that PO→PN substitution leads to the formation of an additional intramolecular hydrogen bond with phosphate oxygen leading to an increased stability of interaction, which is supported by an enhanced docking score and enhanced in vitro activity. In accordance with the analyzed activity of the **10IU** compound we suggest that PO→PN substitution can be used as a general strategy to stabilize the active conformation of the compounds containing diphosphate groups. 

It has to be noted that the binding mode of **10A** with the acceptor binding site of PARP-2 is characterized by the high solvent exposure of the compound and multiple polar interactions, which include at least 10 hydrogen bonds. The hydrophilic nature of the stabilizing interactions with the PARP-1/2 of the compound can explain the moderate activity of the **10A** compound, and provides further strategies of compound optimization. 

The observed selectivity of **10A** to PARP-2 can be explained by the variable region of PARP-1/2 in proximity to the acceptor binding site. In particular, loops of PARP-1 (978–986) and PARP-2 (544–556) have a distinct conformation and amino acid composition (Figure 8A–C), while according to the structural model, Asn555 of PARP-2 is involved in the formation of a hydrogen bond with phosphate oxygen of **10A** and replaced by Leu985 in the case of PARP-1 (Figure 8B,C). Additionally, PARP-1 lacks stabilizing interactions with Tyr552 due to shortening of the corresponding loop. This is supported by the lower XP binding score for PARP-1 that was −7.975 and −10.574 in case of PARP-2. 

Furthermore, lack of activity of **10A** against PARP-3 supports the before suggested mechanism of action. Indeed, PARP-3 structure was reported to be different from PARP-1/2 and characterized as mono(ADP-ribose) transferase [79,80]. In accordance with this data, molecular docking predicted that **10A** does not have the same mode of binding to PARP-3 as to PARP-1/2 nor other MorXppA compounds. Analysis of the binding site revealed that this could be due to steric hindrance caused by Arg408 and Lys421 residues, as well as the hindrance of the hydrogen bond acceptor and donor groups of Gln512 and Arg408, correspondingly, by small molecule (Figure 8D). 

On the whole, structural analysis of the binding modes of **10A** together with experimental data suggests that this compound acts by a different mechanism than known PARP-1/2 potent inhibitors and binds to the acceptor substrate binding site, but not to the donor NAD+ binding site. Additionally, the presence of variable regions in proximity of the acceptor binding site may lead to enhanced PARP-2 specificity of **10A**. 

## 3. Materials and Methods 

### 3.1. Chemistry

General information, NMR, mass and IR spectra can be found in Supplementary Materials.

#### 3.1.1. General Procedure for the Synthesis of Morpholino Nucleosides **2**

Ribonucleosides **1** (4 mmol) were suspended in EtOH (80 mL). A warm solution of NaIO_4_ (4.2 mmol, 0.9 g) in water (4 mL) was added to the suspension under vigorous stirring. In 15 min, (NH_4_)_2_B_4_O_7_∙4H_2_O (4.8 mmol, 1.26 g) was added. The pH of the reaction mixture was maintained between 8.5 and 9.0 by adding triethylamine (TEA, total amount 0.6–1.0 mL). After 1.5 h of stirring, the precipitate was separated by filtration and washed with EtOH (2 × 4 mL). NaCNBH_3_ (5.2 mmol, 0.326 g) was added to the combined filtrates and the stirring was continued. In 1 h, trifluoroacetic acid (TFA) was added till pH 3.0–4.0. In 2 h, solvents were evaporated and the residue was dried by coevaporation with MeCN (3×10 mL) and toluene (3×10 mL), suspended in DMF (16 mL) and Et_3_N (12 mmol, 1.7 mL). Triphenylmethyl chloride (TrCl) (3.5 mmol, 0.975 g) was added to the suspension, and the stirring was performed overnight. The reaction was quenched by the addition of MeOH (5 mL); the solution was evaporated up to half of a volume and poured into the water (300 mL). The precipitate was separated by filtration and dried. After that, the precipitate was dissolved in CH_2_Cl_2_ (10 mL), and the target morpholino nucleoside was precipitated by the petroleum ether (250 mL). The suspension was cooled (−20 °С, 2 h) and the precipitate was separated by filtration, washed by petroleum ether, and dried under vacuum.

#### 3.1.2. General Procedure for the Synthesis of Morpholino Nucleosides **11**

Morpholino nucleosides **2A,G,C** (0.1 mmol) were dissolved in 1.5 mL of EtOH, conc. aq. ammonia (3 mL) was added to each solution and the reaction mixtures were stirred at room temperature. After the deblocation of heterocyclic bases was completed (1–2 days), the reaction mixtures were evaporated. Aq. 80% AcOH (1.5 mL) was added to the residues and to the morpholino nucleosides **2U,T,IU,BrU,ClU** (0.1 mmol). In 1.5 h, all reaction mixtures were diluted with water (5 mL) and washed by CH_2_Cl_2_ (5 × 5 mL). Aqueous layers were evaporated, coevaporated with toluene (3 × 5 mL) and again with water (3 × 5 mL). After drying in vacuum, morpholino nucleosides 11 were obtained in a yield of 90% as partial acetate salts.

#### 3.1.3. General Procedure for the Phosphorylation of Morpholino Nucleosides **2**

Morpholino nucleosides **2** (0.1 mmol) were dissolved in dry Py (1 mL). The solution was cooled in an ice bath (−15 °С). POCl_3_ (0.4 mmol, 0.037 mL) was added to the solution under stirring. In 20 min, 1 M TEAB (2 mL) was added to the cold reaction mixture. The reaction mixture was stirred for 10 min at room temperature. A) Compounds **2A,G,C,IU**: the reaction mixture was distributed between CH_2_Cl_2_ (10 mL) and water (10 mL). An aqueous layer was washed with CH_2_Cl_2_ (10 mL). Combined organic layers were dried (Na_2_SO_4_), filtrated and evaporated. CH_2_Cl_2_ (1 mL) was added to the residue, and the target products **3A,G,C,IU** were precipitated by petroleum ether (12 mL). The suspension was cooled (−20 °С, 2 h), the precipitate was separated by centrifugation, washed by petroleum ether and dried under vacuum. B) Compounds **2U,T,BrU,ClU**: the reaction mixture was distributed between CH_2_Cl_2_ (10 mL) and water (10 mL). An aqueous layer was evaporated, and the target monophosphates **3U,T,BrU,ClU** were purified by RPC in a gradient of MeCN (0%–75 %) in water. Appropriate fractions were combined and evaporated. The residue was dried under vacuum. Morpholino nucleotides **3A-ClU** were obtained as TEA salts

#### 3.1.4. General Procedure for the Synthesis of Conjugates **4**

Morpholino nucleotides **3A-ClU** (TEA salt, 0.05 mmol) and Ph_3_P (0.15 mmol, 0.039 g) were dissolved in 1,3-dimethyl-2-imidazolidinone (DMI, 0.5 mL). *N*-Methylimidazole (MeIm, 0.6 mmol, 0.05 mL) and (PyS)_2_ (0.15 mmol, 0.03 g) were added to the solution, and the reaction mixture was stirred for 15 min. Adenosine 5′-phosphate (*n*-Bu_3_N salt, 0.2 mmol in 0.5 mL of DMI) was added to the reaction mixture and stirring was continued for 1 h. Conc. aq. NH_3_ (2 mL) was added to the reaction mixture in case of the synthesis of conjugates **4A,G,C,** and after the deblocation of heterocyclic bases was completed (24 h at room temperature) the reaction mixture was evaporated up to its initial volume (1 mL). Then the reaction mixture was poured into Et_2_O (20 mL) and cooled (−20 °С, 2 h). The precipitate was separated by centrifugation, washed by Et_2_O and dried. *N*-Tr-protected conjugates **4A-ClU** were purified by RPC in a linear gradient of MeCN in water in the presence of 0.1 M NH_4_HCO_3_. Appropriate fractions were collected and evaporated. Traces of a buffer were removed by repeating the evaporation from aq. EtOH. To remove the Tr protective group, the residue was dissolved in 80% aq. AcOH (1 mL). In 1 h, the conjugates **4A-ClU** were precipitated by 4% NaClO_4_ in acetone (10 mL), cooled (−20 °С, 2 h), and centrifuged. The precipitate was washed with acetone and dried. 

#### 3.1.5. General Procedure for the Synthesis of 2′-aminomethylmorpholino Nucleosides **7**

Morpholino nucleosides **2A-ClU** (0.7 mmol), Ph_3_P (1.4 mmol, 0.367 g) and imidazole (1.4 mmol, 0.095 g) were dissolved in dichloroethane (DCE) (2.8 mL). The solution was cooled in an ice bath (−5 °С). A solution of I_2_ (0.9 mmol, 0.23 g) in DCE (0.4 mL) was added under vigorous stirring. The cooling was removed and the reaction mixture was stirred for 5 h at r.t. The reaction mixture was diluted with CH_2_Cl_2_ (20 mL) and washed with aq. 5 M NaHSO_3_ (20 mL), sat. aq. NaHCO_3_ (20 mL) and water (2×20 mL). The organic layer was dried (Na_2_SO_4_), filtered and evaporated. 2′-Iodomethylmorpholino nucleosides **5A-ClU** were purified by silica gel chromatography in a gradient of acetone in CH_2_Cl_2_ (0%–20%). Appropriate fractions were combined and evaporated. The residue was dissolved in CH_2_Cl_2_ (1 mL) and precipitated with petroleum ether (15 mL). The suspension was cooled (−20 °С, 2 h), the precipitate was separated by centrifugation, washed with petroleum ether and dried under vacuum. Morpholino nucleosides **5A-ClU** (0.5 mmol) were dissolved in DMF (2 mL), then NaN_3_ (2.5 mmol, 0.163 g) was added. The suspension was stirred overnight at r.t. and then was diluted with CH_2_Cl_2_ (20 mL) and washed with water (4×10 mL). The organic layer was dried with Na_2_SO_4_ and filtered. The evaporated residue was dissolved in CH_2_Cl_2_ (1 mL) and precipitated by petroleum ether (15 mL). The suspension was cooled (−20 °С, 2 h), the precipitate was separated by centrifugation, washed by petroleum ether and dried under vacuum. 10% Pd/C (0.01 mmol, 10 mg) was added to a solution of 2′-azidomethylmorpholino nucleosides **6A-ClU** (0.1 mmol) in MeOH (1 mL). The mixture was stirred for 48 h under H_2_. The reaction mixture was filtered and evaporated. The residue was dissolved in CH_2_Cl_2_ (0.1 mL) and precipitated by petroleum ether (1.5 mL). The suspension was cooled (−20 °С, 2 h), the precipitate was separated by centrifugation, washed by petroleum ether and dried under vacuum. 

#### 3.1.6. General Procedure for the Synthesis of Conjugates **10**

ADP (*n*-Bu_3_N salt, 0.02 mmol) was dissolved in dimethyl sulfoxide (DMSO) (0.2 mL) and activated as described above for morpholino nucleotides **3A-ClU**. In 1 h, a solution of 2’-aminomethylmorpholino nucleosides **7A-ClU** (0.06 mmol) in DMI (0.2 mL) and *n*-Bu_3_N (0.06 mmol, 0.014 mL) were added to the reaction mixture. In 3 h, the reaction was completed. Further treatment of the reaction mixture, deblocation and purification of target conjugates **10A-ClU** were carried out as described for conjugates **4A-ClU**. 

### 3.2. Biology

#### 3.2.1. Materials

The oligonucleotides were purchased from Biosset (Novosibirsk, Russia). The reagents, solvents and the basic components of buffers were purchased from Sigma (St. Louis, MO, USA), Acros organics (Waltham, MA, USA) or Promega (Madison, WI, USA). γ–[^32^P]-ATP and α–[^32^P]-ATP (with specific activities of 5000 and 3000 Ci/mmol, respectively) were from the Laboratory of Biotechnology (Institute of Chemical Biology and Fundamental Medicine, Novosibirsk, Russia). T4 polynucleotide kinase was from Biosan (Novosibirsk, Russia). SUMO fusion expression vector pETHSUL (GenBank: EF205333.1) and pSUPER vector coding for the catalytic domain of the S. cerevisiae SUMO hydrolase dtUD1 were kindly provided by Dr. P. Loll (Drexel University, Philadelphia, USA). His6 and Strep-II double-tagged dtUD1 hydrolase was expressed and purified as described in ref. [81]. Human recombinant poly(ADP-ribose)-polymerase 1 (EC 2.4.2.30) was prepared as described previously [82]. Murine recombinant poly(ADP-ribose)-polymerase 2 (EC 2.4.2.30) was purified as described previously [83]. Plasmid encoding human PARP-3 was kindly provided by Dr. A. Ishchenko (Gustave Roussy, Université Paris-Saclay, France). Poly(ADP-ribose)-polymerase 3 (EC 2.4.2.30) was purified according to [6].

#### 3.2.2. Oligonucleotide Substrates

The oligodeoxyribonucleotides were 5′-[^32^P]-phosphorylated with T4 polynucleotide kinase as described. Unreacted γ–[^32^P]-ATP was removed using MicroSpinTM G-25 column (Amersham Pharmacia Biotech, Little Chalfont, UK, and GE Healthcare, Chicago, IL, USA ). The 5′-[^32^P]-phosphorylated oligonucleotides were precipitated by 4% LiClO_4_ in acetone and dissolved in water. Complementary oligodeoxynucleotides were annealed as described [6]. The following oligonucleotide sequences were used for the construction of DNA duplexes: whole strands 5′-(d)GGC TTC ATC GTT GTC TCA GAC CTG GTG GAT ACC G-3′; upstream oligonucleotides 5′-(d)CGG TAT CCA CCA GGT CTG-3′ with or without 5′-phosphate groups; downstream oligonucleotide 5′-p-(d)GAC AAC GAT GAA GCC-3′.

#### 3.2.3. Synthesis of [^32^P]-NAD+

The radioactive NAD+ was synthesized from α–[^32^P]-ATP. The reaction mixture containing 1 mM ATP, 10 MBq of α–[^32^P]-ATP, 20 mM MgCl2, 2 mM beta-nicotinamide mononucleotide, and 5 mg/mL nicotinamide nucleotide adenylyltransferase 1 in 25 mM Tris-HCl, pH 7.5, was incubated at 37 °C for 60 min, then stopped by heating to 80 °C for 5 min. The denatured protein was removed by centrifugation.

#### 3.2.4. PARP-1 and PARP-2 Enzyme Assay

The reaction of autopoly(ADP-ribosyl)ation was carried out as follows: for PARP-1 50 mM tris-HCl, pH 8.0, 20 mM MgCl_2_, 150 mM NaCl, and 7 mM β-mercaptoethanol, activated DNA 2 OE/mL, 0.3 mM [^32^P] NAD+ at 37 °C. The reaction was initiated by adding PARP-1 to 200 nM and the reaction mixtures were incubated for 1.5 min. For PARP-2: 50 mM tris-HCl, pH 8.0, 3 mM spermin, 150 mM NaCl, and 7 mM β-mercaptoethanol, activated DNA 2 OE/mL, 0.6 mM [^32^P] NAD+ at 37 °C. The reaction was initiated by adding PARP-2 to 800 nM and the reaction mixtures were incubated for 5 min. The reaction was stopped by placing 10 μL aliquots onto Whatman 1 paper filters soaked with 5 % TCA. The filters were washed with 5% TCA for four times and dried in air after the removal of TCA with 90% ethanol. The incorporation of radioactivity into the product was calculated with a Tri-Carb 2800 scintillation counter (Perkin Elmer, Waltham, MA, USA) according to the Cherenkov method or using Typhoon FLA 9500 scanner (GE Healthcare, Chicago, IL, USA). To determine the IC_50_ value of inhibitors (concentration of the compound required reducing the enzyme activity by 50%), we studied the activity of the enzyme at different concentrations of inhibitors. Measurements were done in at least two independent experiments. IC_50_ values were calculated using the Origin Pro 8.0 software by nonlinear regression analysis. 

The determination of the kinetic parameters of inhibition Km and Vmax was carried out as described [76]. In all experiments, the points on the experimental curves represent the average of a minimum three independent experiments. Standard deviation did not exceed 10%.

#### 3.2.5. Inhibitors Screening, ADP-ribosylation of DNA by PARP-3

The reactions were performed in the presence of 2 mM MgCl_2_ as a cofactor in HDB buffer solution containing HEPES-KOH, pH 8.6, 0.25 mg/mL BSA and 0.5 mM DTT. The reaction mixtures (final volume 10 μL) contained 0.02 μM [^32^P]-DNA substrate, 0.1 μM PARP-3, 5, 10, 100 or 500 μM NAD+ in the absence or presence of various concentrations of the inhibitor, which are indicated on the figures. The reaction was carried out for 20 min at 37 °С. The mixtures were separated in a standard 20% denaturing acrylamide gel. The gels were dried and subjected to autoradiography and/or phosphorimaging for quantitation using the Typhoon imaging system (GE Healthcare Life Sciences, Chicago, IL, USA). Quantitative processing was carried out using OriginPro7.5, Microcal Software (Origin Systems, Houston, TX, USA). In all experiments, the points on the experimental curves represent the average of minimum three independent experiments. Standard deviation did not exceed 10%.

#### 3.2.6. Inhibitors Screening, ADP-ribosylation of PARP-3

The reactions were performed in the presence of 2 mM MgCl_2_ as a cofactor in HDB buffer. The reaction mixtures (final volume 10 μL) contained 0.1 μM DNA substrate, 0.5 μM PARP-3, 5 μM [^32^P]-NAD+ and 0, 35 or 95 μM NAD+ in the absence or presence of various concentrations of the inhibitor, which are indicated on the figures. The reaction mixtures were incubated for 20 min at 37 °С then terminated by adding the 5x gel loading Laemmli sample buffer and heating at 85 °C for 10 min. The mixtures were resolved on a 10% SDS-PAG as described in [84] The gels were dried and subjected to autoradiography and/or phosphorimaging for quantitation using the Typhoon imaging system from GE Healthcare Life Sciences. Quantitative processing was carried out using OriginPro7.5, Microcal Software, USA. In all experiments, the points on the experimental curves represent the average of a minimum three independent experiments. Standard deviation did not exceed 10%.

### 3.3. Structural Studies

Reference crystal structures of PARP-1 and PARP-2 were taken from the PDB database with identifiers 4ZZZ [85] and 3KJD [86], correspondingly. Localization of the ADP fragment of acceptor NAD+ was predicted by a structural alignment of the reference PARP-1/2 crystal structures with the crystal structure of PARP from *Gallus gallus* in complex with NAD+ (PDB identifier 1A26, [78]). The localization of donor NAD+ was predicted by structural alignment with catalytic domain of PARP-1 (PDB identifier 6BHV) [38] in complex with BAD, structural analog of NAD+. In this case C1 carbon of benzamide was manually replaced by nitrogen. Structural alignment was carried out using Maestro (Schrodinger Inc.). Obtained complexes were minimized with Protein Preparation Wizard (Schrodinger Inc.). Preliminary the side chains of amino acid residues containing steric hindrance with BAD were removed and re-added using Prime Module (Schrödinger Inc.). 

3D structures of small molecules under study were prepared using LigPrep module (Schrödinger Inc.). Molecular docking was carried out using Glide in the extra-precision mode (XP Score). Small molecule binding pose with the best XP score were selected [87]. Two grids were calculated for the molecular docking study. First grid was calculated based on the center of mass of the acceptor ADP fragment of NAD+ and the second was calculated based on the center mass of the donor NAD+ ligand. Molecular dynamics simulation was carried out using the pmemd module of Amber12 software [88]. The RESP charges were calculated using the ANTECHAMBER module based on ab initio calculations of electrostatic potentials using theHF/6-31G* level of theory using Gaussian 03 [89,90]. Amberff99SB and GAFF force fields were used for the simulation of molecular dynamics of protein and small molecules, respectively. Implicit solvent generalized Born (GB) Onufriev was used for MD simulation with mbondi2 radii set [91]. 

Molecular dynamics protocol included 1000 steps of steepest descent minimization, 100 ps heating from 0K to 300K, and 100 ns production molecular dynamics simulation. Langevin thermostat with a 5 ps^−1^ collision rate was used [92]; 1 kcal/mole restraints on protein CA atoms and heavy atoms of the adenosine fragment of NAD+ analogs were applied at all steps of MD simulation.

## 4. Conclusions

Morpholino nucleosides are readily available derivatives of natural ribonucleosides that found a broad application in the synthesis of morpholino oligonucleotides as nucleic acid mimetics. These are important and widely used molecular tools in molecular biology and are now even in clinic for an antisense-based treatment. However, a high potential of morpholino nucleoside monomers as terminators in gene sequencing, polymerase inhibitors (in phosphorylated form), or as a part of other low molecular weight biological regulators is not investigated in its entirety. In this regard, it is essential that the morpholine ring is a well-known and important pharmacophore widely used in numerous medicines [93]. The present study allowed for a broader involvement of morpholino nucleosides into a pool of potentially biologically active compounds. We proposed a novel class of PARP-1 and PARP-2 inhibitors consisting of various types of morpholino nucleosides and ADP [45].

The highest inhibition activity against PARP-1 was found for ADP conjugates containing 5-iodouracil morpholino nucleosides. These compounds were predicted to target the NA binding site by a common mechanism to known for potential PARP-1 inhibitors.

Surprisingly, in relation to PARP-2 the most active compound with IC_50_~50 μM was dinucleotide-containing adenine morpholino nucleoside. The activity of 2′-aminomethylmorpholino nucleoside of adenine could be achieved by targeting to the acceptor substrate binding site by mimicking interactions of the PAR substrate. In particular, it has been shown that the amine group of the morpholine ring can mimic an interaction of the 2′–OH group of adenine riboside with Glu558. The replacement of P–O to P–N bond leads to the enhanced stabilization of pyrophosphate conformation due to the formation of an intramolecular hydrogen bond, and followed to a subsequently higher inhibition activity. Stabilizing interactions with variable regions lead to enhanced selectivity in relation to PARP-2. Strikingly, these predictions are validated by a fluorescence anisotropy assay that suggests the mixed mode of inhibitory activity by targeting the active and allosteric centers simultaneously. This is in contrast to 5-iodouracil morpholino nucleosides as well as the **3-AB** compound, a well known PARP inhibitor targeting nicotinamide binding site. 

Lack of activity of **10A** against PARP-3 that has a different amino acid composition of the acceptor binding site as well as substrates selectivity further confirms the predicted mechanism of action. It has to be noted that only 5-iodouracil morpholino nucleosides **10IU** demonstrated low activity against PARP-3 at mM concentration. These data are in accordance with generally observed up to two orders lower activity of known PARP-1/2 inhibitors against PARP-3, targeting the donor nicotinamide binding pocket [94].

Interestingly, it has been shown that occupancy of the NAD+-binding site with benzamide adenine dinucleotide (BAD) locks PARP-1 on a DNA break [38]. It makes a design of new NAD+ analogs a promising strategy to create a wide spectrum of the compounds for future clinical usage under PARP inhibitor therapy. Conducted analysis suggests that compounds based on 2′-aminomethylmorpholino nucleosides target PARP-1/2 by a novel molecular mechanism distinct from a known class of potent inhibitors directed on the NA donor subsite, but not on the acceptor substrate binding site. It opens new horizons for the development of additional classes of PARP-1/2 selective inhibitors. 

Taking into account our enzyme assay and molecular modeling calculations, a new strategy for conformation stabilization and enhancement of the activity and selectivity of a novel class of NAD+ analogs may be proposed.

## Figures and Tables

**Table 1:** Residual PARP-1 and PARP-2 activity (%) in the presence of 1 mM compounds **4** and **10** or IC_50_ (μМ)**.**

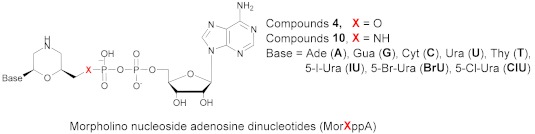					
Compound	PARP-1	PARP-2	Compound	PARP-1	PARP-2
**4A** **4G** **4C** **4U** **4T** **4IU** **4BrU** **4ClU**	80% ± 14% 45.5% ± 3.5% 96.0% ± 2.8% 93.0% ± 9.9% 40% ± 20% 255 ± 5 μM 36.0% ± 8.5% 57.5% ± 7.8%	421 ± 6 μM 34% ± 18% 66.0% ± 8.5% 67% ± 4% 474 ± 14 μM 160 ± 10 μM 474 ± 71 μM 46.8% ± 9.5%	**10A** **10G** **10C** **10U** **10T** **10IU** **10BrU** **10ClU**	353 ± 4 μM 74% ± 21% 87.5% ± 7.8% 89% ± 26% 220 ± 50 μM 126 ± 6 μM 60% ± 27% 46% ± 5%	63 ±10 μM 224 ± 24 μM 34.5% ± 0.7% 33% ± 5% 136 ± 2 μM 110 ± 4 μM 254 ± 100 μM 47% ± 3%

**Table 2:** Residual PARP-1 and PARP-2 activity (%) or IC_50_, μМ, in the presence of 1 mM morpholino nucleosides and ribonucleosides.

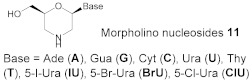			Ribonucleosides		
Compound	PARP-1	PARP-2	Compound	PARP-1	PARP-2
**11A**	82% ± 6%	90 % ± 11%	**Ado**	77% ± 21%	86% ± 1%
**11G**	139% ± 21 μМ	64% ± 25%	**Guo**	60% ± 13%	46% ± 25%
**11C**	200% ± 20 μМ	58% ± 6%	**Cyd**	81% ± 16%	85% ± 4%
**11U**	91% ± 24%	97% ± 8%	**Urd**	7% 4 ± 20%	96% ± 10%
**11T**	148 ± 53 μМ	114 ± 32 μМ	**rThd**	277 ± 107 μМ	330 ± 61 μМ
**11IU**	53 ± 12 μМ	85 ± 9 μМ	**5-I-Urd**	49 ± 9 μМ	26.5 ± 3.5 μМ
**11BrU**	61 ± 16 μМ	210 ± 48 μМ	**5-Br-Urd**	244 ± 83 μМ	190 ± 16 μМ
**11ClU**	233 ± 25 μМ	378 μМ	**5-Cl-Urd**	82% ±17 %	196 ± 25 μМ

## Supplementary Materials

Supplementary materials can be found at https://www.mdpi.com/1422-0067/21/1/214/s1.
