# Supplementary material for: Design, Synthesis and Molecular Modeling Study of Conjugates of ADP and Morpholino Nucleosides as A Novel Class of Inhibitors of PARP-1, PARP-2 and PARP-3
Source: Int J Mol Sci. 2019 Dec 27;21(1):214. doi: 10.3390/ijms21010214 (PMC6982223; doi:10.3390/ijms21010214)
Supplement: Supplementary file 1 [file ijms-21-00214-s001.pdf]

Design, synthesis and molecular modeling study of conjugates of ADP and morpholino nucleosides as a novel class of inhibitors of PARP-1, PARP-2 and PARP-3

Yuliya V. Sherstyuk [1], Nikita V. Ivanisenko [2], Alexandra L. Zakharenko [1], Maria V. Sukhanova [1], Roman Y. Peshkov [3], Ilia V. Eltsov [3], Mikhail M. Kutuzov [1], Tatiana A. Kurgina [1], Ekaterina A. Belousova [1], Vladimir A. Ivanisenko [2], Olga I. Lavrik [1], Vladimir N. Silnikov [1] and Tatyana V. Abramova [1]*

1 Institute of Chemical Biology and Fundamental Medicine SB RAS, Lavrent'ev Ave, 8, Novosibirsk, 630090, Russia; yuliya.tarasenko2012@gmail.com (Y.V.S.); sashaz@niboch.nsc.ru (A.L.Z.); mary@niboch.nsc.ru (M.V.S.); kutuzov.mm@mail.ru (M.M.K.); t.a.kurgina@gmail.com (T.A.K.); rina@niboch.nsc.ru (E.A.B.); lavrik@niboch.nsc.ru (O.I.L.); silnik@niboch.nsc.ru (V.N.S); abramova@niboch.nsc.ru (T.V.A.)

2 Federal Research Centre, Institute of Cytology and Genetics SB RAS, Lavrent'ev Ave, 10, Novosibirsk, 630090, Russia; n.ivanisenko@gmail.com (N.V.I.); salix@bionet.nsc.ru (V.A.I.)

3 Novosibirsk State University, Pirogova St., 2, Novosibirsk 630090, Russia; peshkov@nioch.nsc.ru (R.Y.P.); eiv@fen.nsu.ru (I.V.E.)

* Correspondence: abramova@niboch.nsc.ru

Materials and methods

General, chemistry

We used ribonucleosides from ChemGenes (USA). All other reagents and solvents were purchased from Sigma-Aldrich (USA), Alfa Aesar (USA) and Reachem (Russia). Organic solvents were dried and purified by standard procedures. NMR spectra were acquired on Bruker AM-400, AV-300 and AV-500 instruments (Bruker, Germany) in appropriate deuterated solvents at 30 °C. Chemical shifts (δ) are reported in ppm relative to the TMS signal. In the case of ^{31}P, external standard of 85 % H_3PO_4 was used. Coupling constants *J* are reported in Hertz. IR spectra were recorded on a Vector 22 (Bruker Optics, Germany). MALDI TOF and ESI mass spectra were registered on Autoflex III mass spectrometer (Bruker Daltonics, Germany) using 2,5-dihydroxybenzoic acid as a matrix (MALDI TOF), or on Agilent ESI MSD XCT Ion Trap (Agilent Technologies, USA) in positive or negative mode at The Center of Cooperative Use ("Proteomics," Russian Academy of Sciences). Monitoring of a reaction progress and quantitative analytical HPLC were performed on a Milichrom A02 chromatograph system equipped with the MultiChrom program package (Econova, Russia) on a ProntoSIL 125 C18 column (2 × 75 mm) in a gradient of buffer B (0.1 M TEA–AcOH, pH 7.0, 80% MeCN) in buffer A (0.1M TEA–AcOH, pH 7.0, water) with an elution rate of 0.2 mL/min and UV detection at 250, 260, 280, and 300 nm. TLC was carried out on Kieselgel 60 F254 plates (Merck, Germany) in the proper solvent systems and visualized by UV irradiation, ninhydrin (amine groups) or cysteine/aqueous sulfuric acid (nucleoside and trityl group). Preparative silica gel column chromatography, RPC, and anion exchange chromatography were performed using silica gel 60 (40–63 μm/230–400 mesh, Macherey-Nagel, Germany), Poligoprep 100-50 C18 Macherey-Nagel, Germany) and Q-Sepharose Fast Flow (GE Healthcare, USA), respectively. The compositions of all liquid mixtures are indicated as (v/v) percent. All evaporations were performed under reduced pressure.

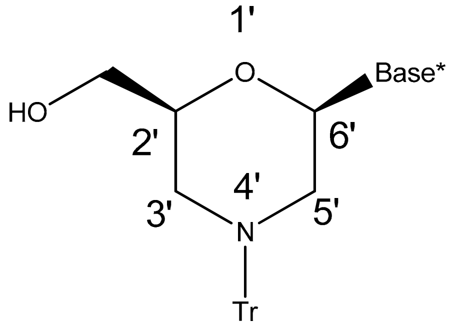

2A,G,C,U,T,IU,BrU,ClU

2’-Hydroxymethyl-4’-*N*-trityl-6’-(N^6-benzoyladenine-9-yl)-morpholine (2A)

^{1}H (400 MHz, acetone-d$_6$): 9.98 (s, 1H, *NH*), 8.65 (s, 1H, *H8*-Ade), 8.26 (s, 1H, *H2*-Ade), 8.09 (br d, *J* 7.5, 2H, *o*-Bz), 7.66-7.50 (m, 9H, *p*-Bz, *m*-Bz, Tr), 7.33 (br t, *J* 7.8, 6H, Tr), 7.21 (br t, *J* 7.2, 3H, Tr), 6.49 (dd, *J* 9.8, 2.5, 1H, *H6’*), 4.46-4.39 (m, 1H, *H2’*), 3.94 (t, *J* 6.1, 1H, CH$_2$-*OH*), 3.64-3.50 (m, 3H, *CH$_2$*-OH, *H5’*), 3.32 (dt, *J* 11.9, 2.4, 1H, *H3’*), 2.09-2.06 (m, 1H, *H3’*), 1.66 (dd, *J* 11.7, 10.6, 1H, *H5’*).

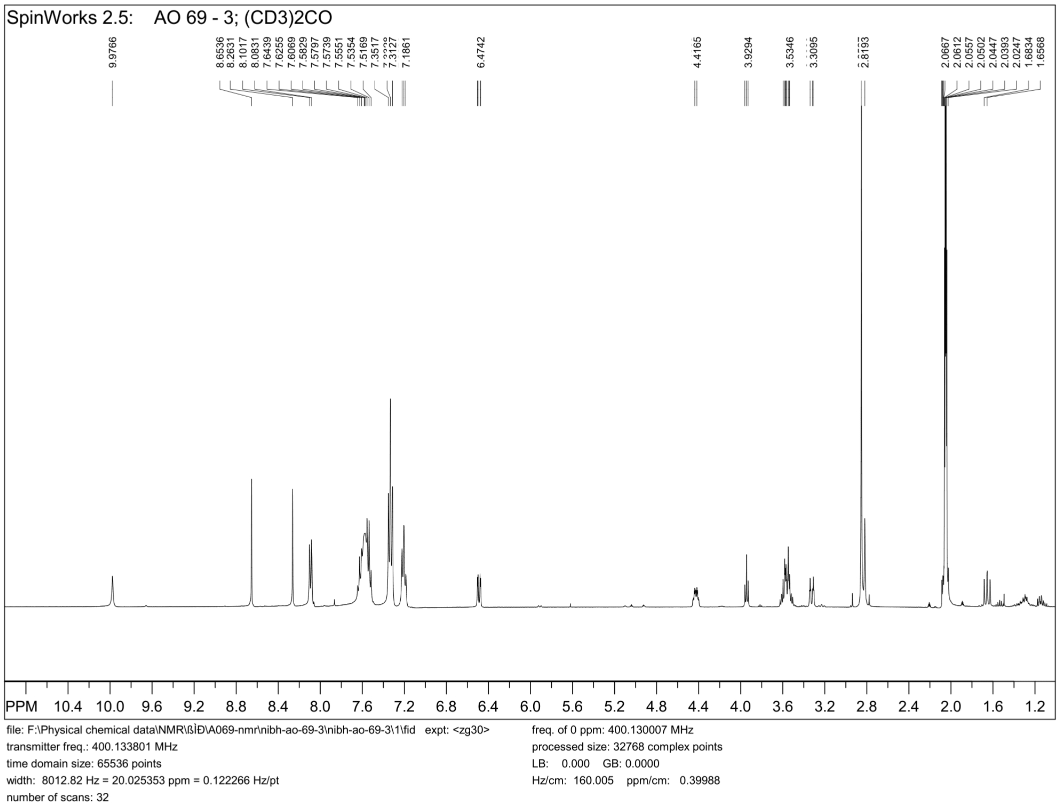

2’-Hydroxymethyl-4’-*N*-trityl-6’-(N^2-isobutyrylguanine-9-yl)-morpholine (2G)

^{1}H (400 MHz, acetone-d$_6$): 12.06 (s, 1H, *NH*), 10.49 (s, 1H, *NH*-Gua), 7.73 (s, 1H, *H8*-Gua), 7.57-7.49 (m, 6H, Tr), 7.33 (br t, *J* 7.8, 6H, Tr), 7.21 (br t, *J* 7.3, 3H, Tr), 6.09 (dd, *J* 9.8, 2.2, 1H, *H6’*), 4.30-4.23 (m, 1H, *H2’*), 3.88 (br s, 1H, CH$_2$-*OH*), 3.52 (qd, *J* 27.6, 5.1, 2H, *CH$_2$*-OH), 3.40 (dt, *J* 11.3, 2.3, 1H, H5’), 3.27 (dt, *J* 11.9, 2.3, 1H, H5’), 2.99 (p, *J* 6.8, 1H, *CH*-iBu), 1.94 (dd, *J* 11.2, 9.9, 1H, *H3’*), 1.57 (dd, *J* 12.1, 10.4, 1H, *H5’*), 1.29 (d, *J* 6.8, 3H, *CH$_3$*-iBu), 1.26 (d, *J* 6.8, 3H, *CH$_3$*-iBu).

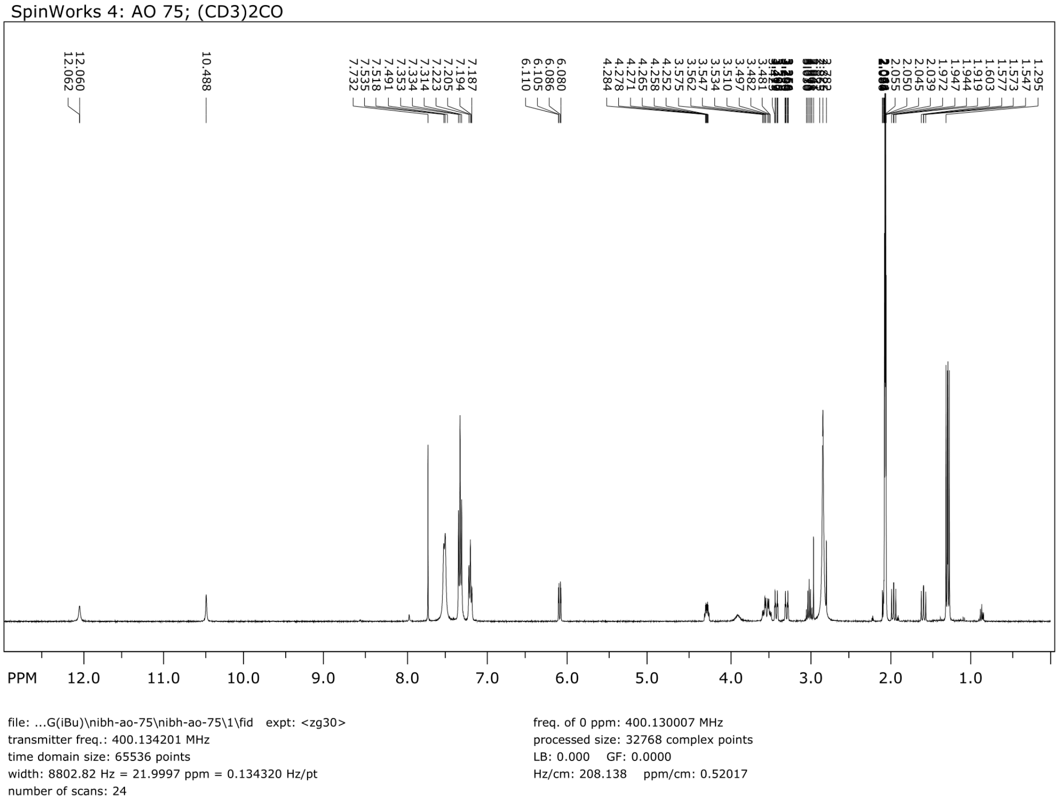

2'-Hydroxymethyl-4'-*N*-trityl-6'-(N^4-benzoylcytosine-1-yl)-morpholine (2C)

^{1}H (400 MHz, acetone-d_6): 8.11 (dt, *J* 7.1, 1.2, *o*-Bz), 7.94 (d, *J* 7.5, 1H, *H6*-Cyt), 7.64 (tt, J 7.4, 1.2, 1H, *m*-Bz), 7.53 (br t, *J* 7.5, 8H, Tr, *p*-Bz), 7.32 (br t, *J* 7.6, 7H, *H5*-Cyt, Tr), 7.20 (br t, *J* 7.2, 3H, Tr), 6.22 (dd, *J* 9.2, 2.4, H6'), 4.37-4.31 (m, 1H, H2'), 3.90 (ap s, 1H, CH_2-*OH*), 3.65-3.54 (m, 2H, *CH_2*-OH), 3.51 (dt, *J* 11.2, 2.4, 1H, H5'), 3.26 (dt, *J* 11.9, 2.4, 1H, H3'), 1.57 (dd, *J* 11.8, 10.6, 1H, *H3'*), 1.38 (dd, *J* 11.2, 9.3, 1H, *H5'*).

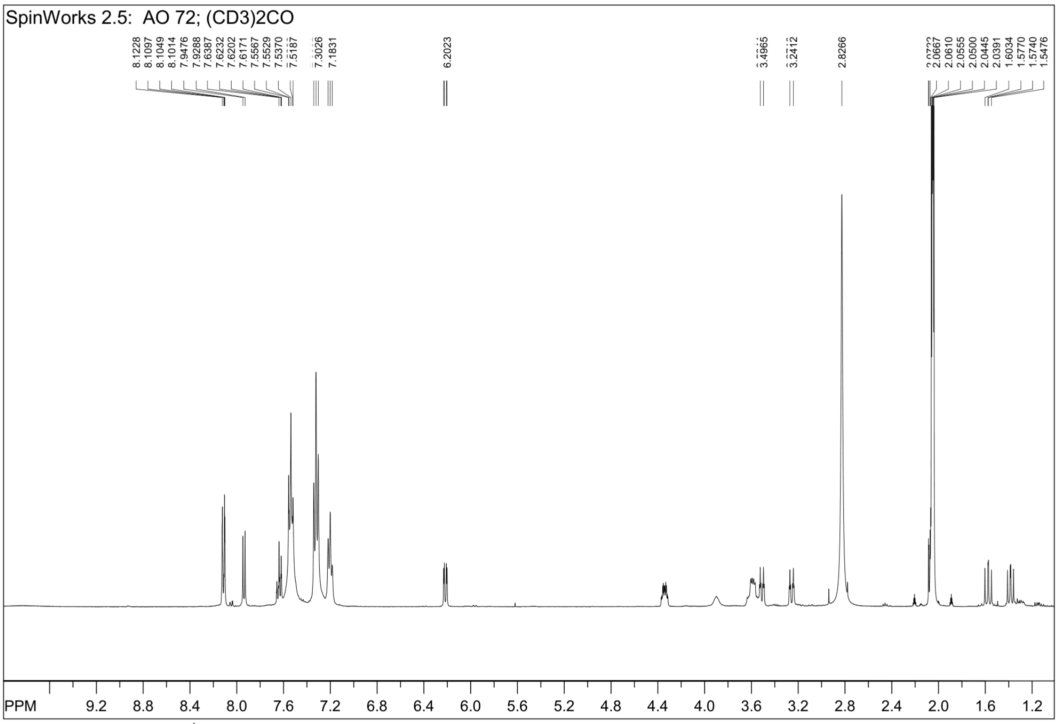

file: F:\Physical chemical data\NMR\ßÎÐ\A072-nmr\nibh-ao-72\nibh-ao-72\1\fid expt: <zg30>
transmitter freq.: 400.134201 MHz
time domain size: 65536 points
width: 8802.82 Hz = 21.999661 ppm = 0.134320 Hz/pt
number of scans: 40

freq. of 0 ppm: 400.130007 MHz
processed size: 32768 complex points
LB: 0.000 GB: 0.0000
Hz/cm: 143.787 ppm/cm: 0.35935

2’-Hydroxymethyl-4’-*N*-trityl-6’-(uracil-1-yl)-morpholine (2U)

^{1}H (400 MHz, acetone-d_6): 9.97 (s, 1H, *NH*-Ura), 7.58-7.47 (m, 6H, Tr), 7.44 (d, *J* 8.2, 1H, *H6*-Ura), 7.32 (br t, *J* 7.6, 6H, Tr), 7.20 (br t, *J* 7.2, 3H, Tr), 6.12 (dd, *J* 9.5, 2.5, 1H, *H6’*), 5.48 (d, *J* 8.2, 1H H5-Ura), 4.34-4.26 (m, 1H, *H2’*), 3.87 (t, *J* 6.2, 1H, CH_2-*OH*), 3.62-3.47 (m, 2H, *CH_2*-OH), 3.34 (dt, *J* 11.3, 2.5, 1H, *H5’*), 3.22 (dt, *J* 11.8, 2.4, 1H, *H3’*), 1.52 (dd, *J* 11.8, 10.6, 1H, *H3’*), 1.32 (dd, *J* 11.2, 9.6, 1H, *H5’*).

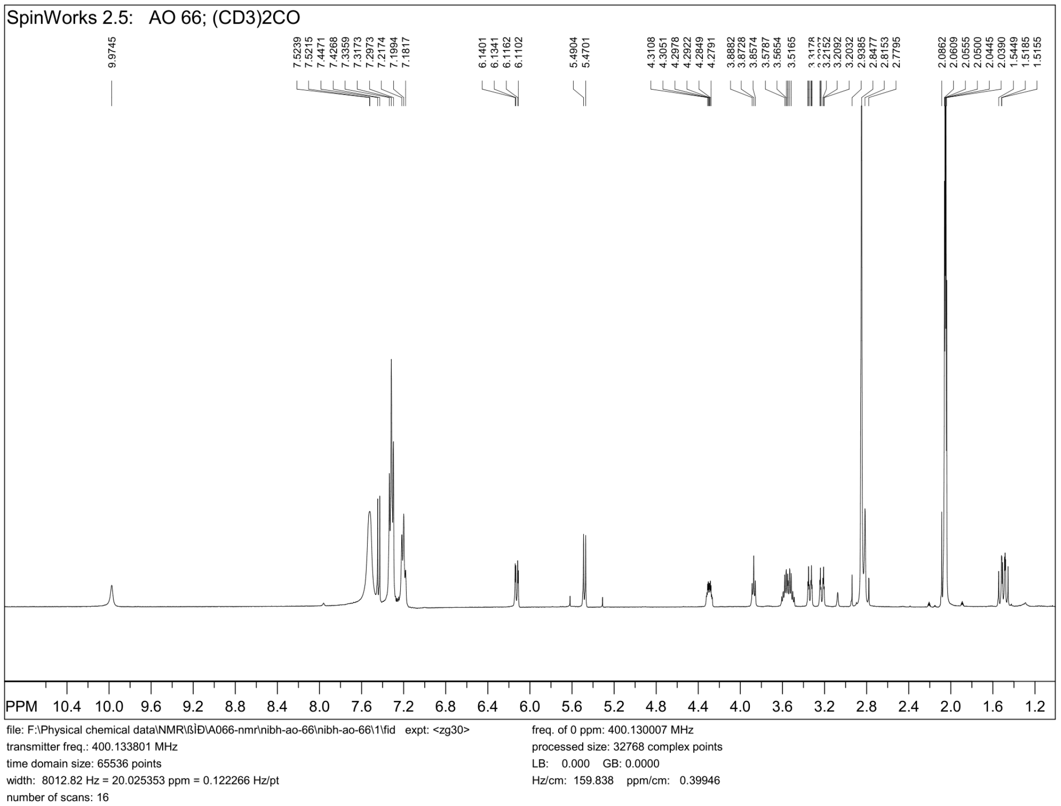

2'-Hydroxymethyl-4'-*N*-trityl-6'-(thymine-1-yl)-morpholine (2T)

^{1}H (400 MHz, acetone-d_6): 9.96 (s, 1H, *NH*-Thy), 7.59-7.45 (m, 6H, Tr), 7.31 (br t, *J* 7.6, 6H, Tr), 7.96 (d, *J* 1.3, 1H, *H6*-Ura), 7.20 (br t, *J* 7.2, 3H, Tr), 6.13 (dd, *J* 9.5, 2.5, 1H, *H6'*), 4.32-4.25 (m, 1H, *H2'*), 3.85 (t, *J* 6.0, 1H, CH_2-*OH*), 3.61-3.48 (m, 2H, *CH_2*-OH), 3.30 (dt, *J* 11.2, 2.5, 1H, *H5'*), 3.22 (dt, *J* 11.9, 2.5, 1H, *H3'*), 1.72 (d, *J* 1.2, 3H, *CH_3*-Thy), 1.56-1.48 (m, 2H, *H3'*, *H5'*).

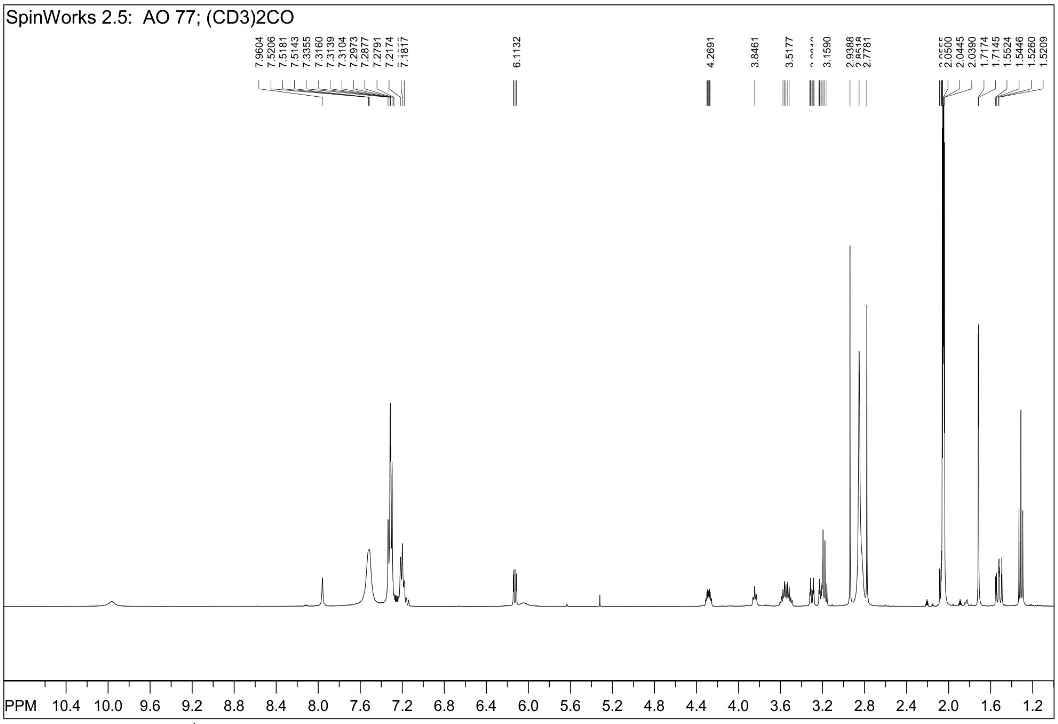

file: F:\Physical chemical data\NMR\ßÌÐ\A077-nmr\nibh-ao-77\nibh-ao-77\1\fid expt: <zg30>
transmitter freq.: 400.133801 MHz
time domain size: 65536 points
width: 8012.82 Hz = 20.025353 ppm = 0.122266 Hz/pt
number of scans: 40

freq. of 0 ppm: 400.130007 MHz
processed size: 32768 complex points
LB: 0.000 GB: 0.0000
Hz/cm: 160.005 ppm/cm: 0.39988

2'-Hydroxymethyl-4'-*N*-trityl-6'-(5-iodouracil-1-yl)-morpholine (2IU)

^{1}H (500 MHz, DMSO-d$_6$): 11.69 (s, 1H, *NH*-Ura), 7.86 (s, 1H, *H6*-Ura), 7.50-7.36 (m, 6H, Tr), 7.34-7.28 (m, 6H, Tr), 7.22-7.17 (m, 3H, Tr), 5.96 (dd, *J* 9.3, 1.9, 1H, *H6'*), 4.75 (t, *J* 5.9, 1H, CH$_2$-*OH*), 4.20-4.13 (m, 1H, *H2'*), 3.39 (br t, *J* 5.2, 2H, *CH$_2$*-OH), 3.20 (br d, *J* 11.1, 1H, *H5'*), 2.99 (br d, *J* 11.8, 1H, *H3'*), 1.43 (t, *J* 11.2, 1H, *H3'*), 1.32 (br t, *J* 10.4, 1H, *H5'*).

^{13}C (100 MHz, DMSO-d$_6$): 160.44, 149.52, 147.79, 144.30, 127.80, 127.55, 126.67, 80.59, 80.53, 76.99, 76.27, 69.51, 61.94, 45.72.

MS MALDI-TOF (*m/z*): [M-H]$^-$ calcd for $C_{28}H_{25}IN_3O_4^-$ 594.089; found 594.039

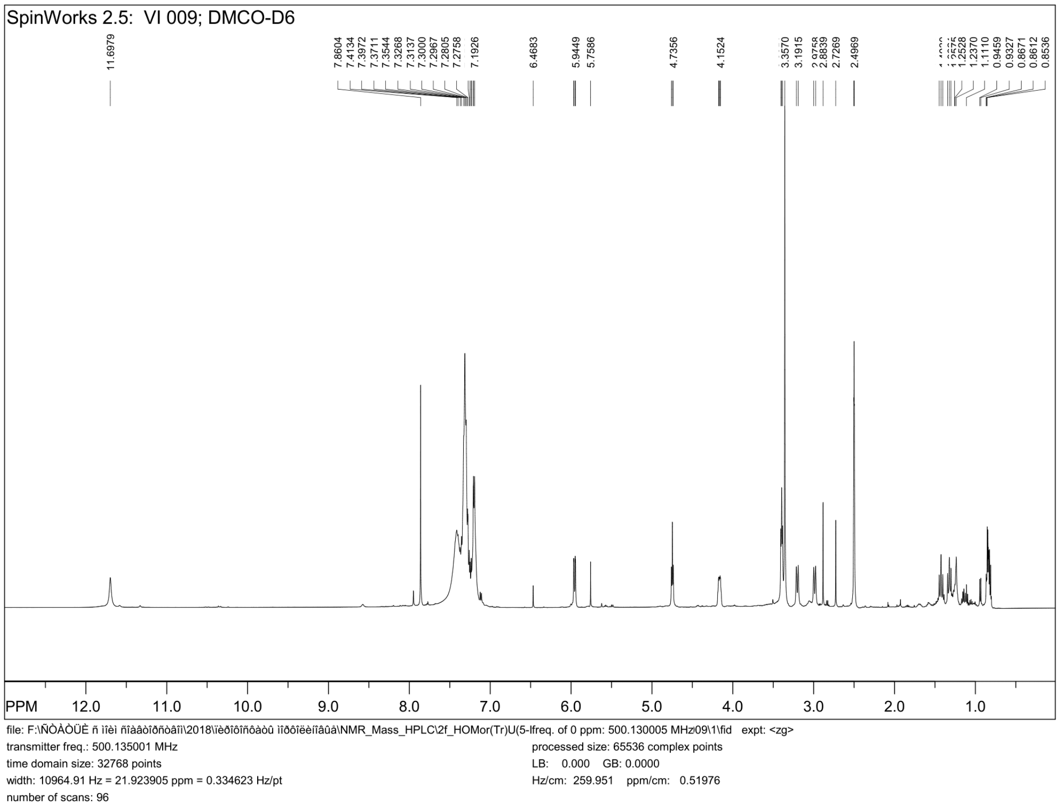

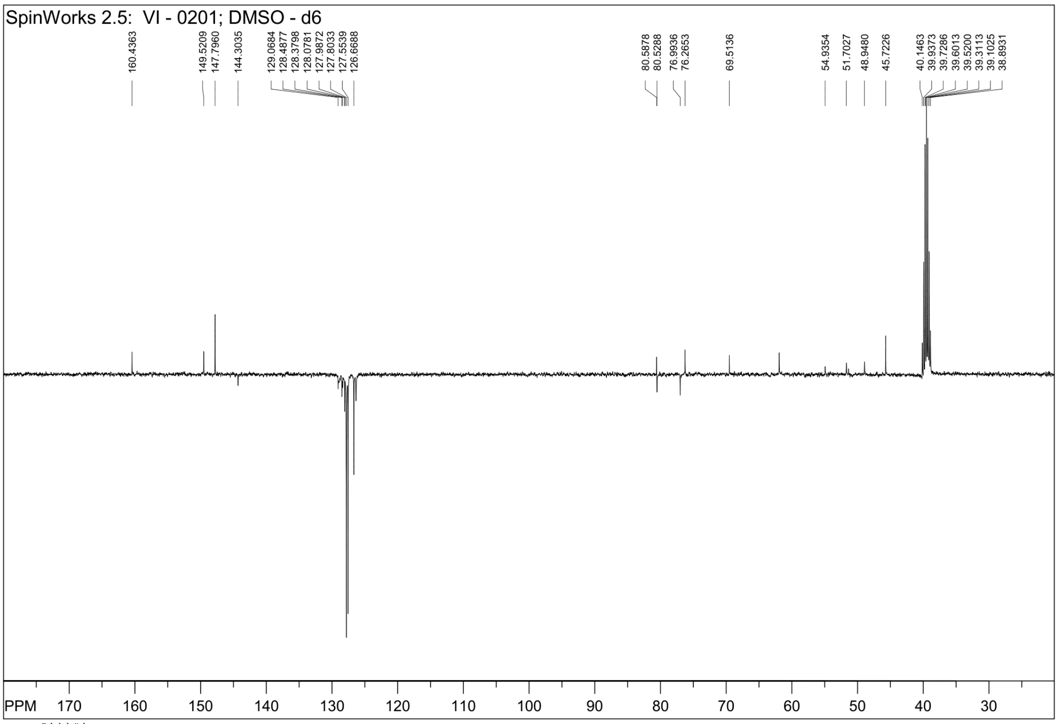

file: F:\ÑÒÀÒÜÈ ñ ìîèì ñîàâòîðñòâîì\2018\ïèðîôîñôàòû ìîðôîëèíîâûå\NMR_Mass_HPLC\2f_HOMor(Tr)U(5-lfreq. of 0 ppm: 100.612811 MHz expt: <jmod>
transmitter freq.: 100.624339 MHz
time domain size: 65536 points
width: 24038.46 Hz = 238.893111 ppm = 0.366798 Hz/pt
number of scans: 336
processed size: 131072 complex points
LB: 0.000 GB: 0.0000
Hz/cm: 643.704 ppm/cm: 6.39710

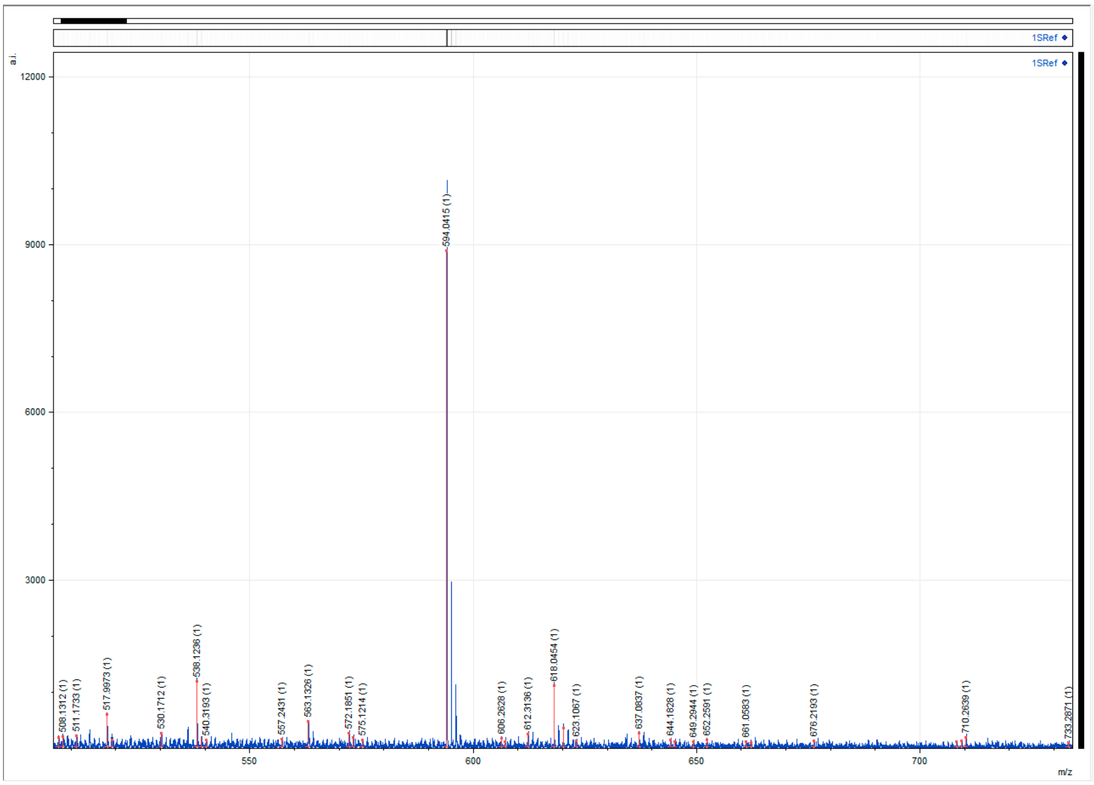

2’-Hydroxymethyl-4’-*N*-trityl-6’-(5-bromouracil-1-yl)-morpholine (2BrU)

^{1}H (400 MHz, DMSO-d_6): 7.88 (s, 1H, *H6*-Ura), 7.42-7.33 (m, 6H, Tr), 7.31-7.24 (m, 6H, Tr), 7.19-7.14 (m, 3H, Tr), 5.94 (dd, *J* 9.3, 1.9, 1H, *H6’*), 4.71 (ap s, 1H, CH_2-*OH*), 4.18-4.11 (m, 1H, *H2’*), 3.40-3.34 (m, 2H, *CH_2*-OH), 3.19 (br d, *J* 11.1, 1H, *H5’*), 2.96 (br d, *J* 11.6, 1H, *H3’*), 1.41 (t, *J* 11.3, 1H, *H3’*), 1.32 (br t, *J* 10.2, 1H, *H5’*).

^{13}C (100 MHz, DMSO-d_6): 159.11, 149.17, 147.81, 139.93, 128.55-126.02, 95.77, 80.57, 76.95, 76.27, 61.94, 51.56, 45.56.

MS ESI (*m/z*): $[M-H]^-$ calcd for $C_{28}H_{25}BrN_3O_4^-$ 547.11; found 546.99

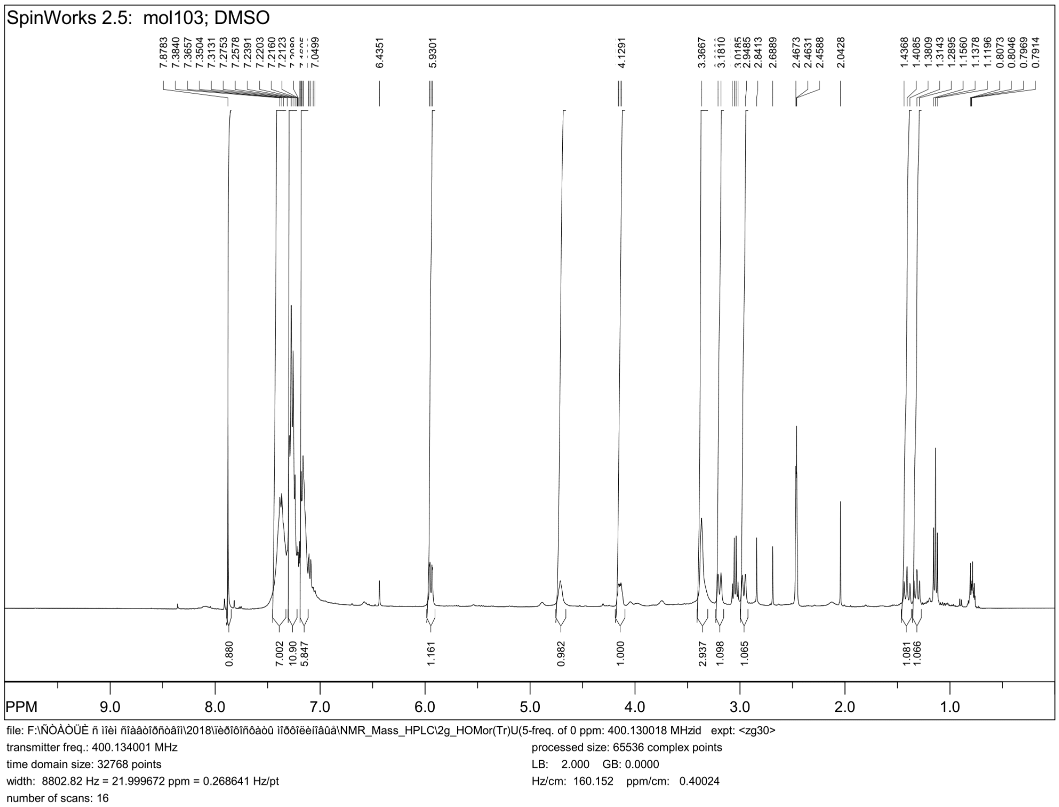

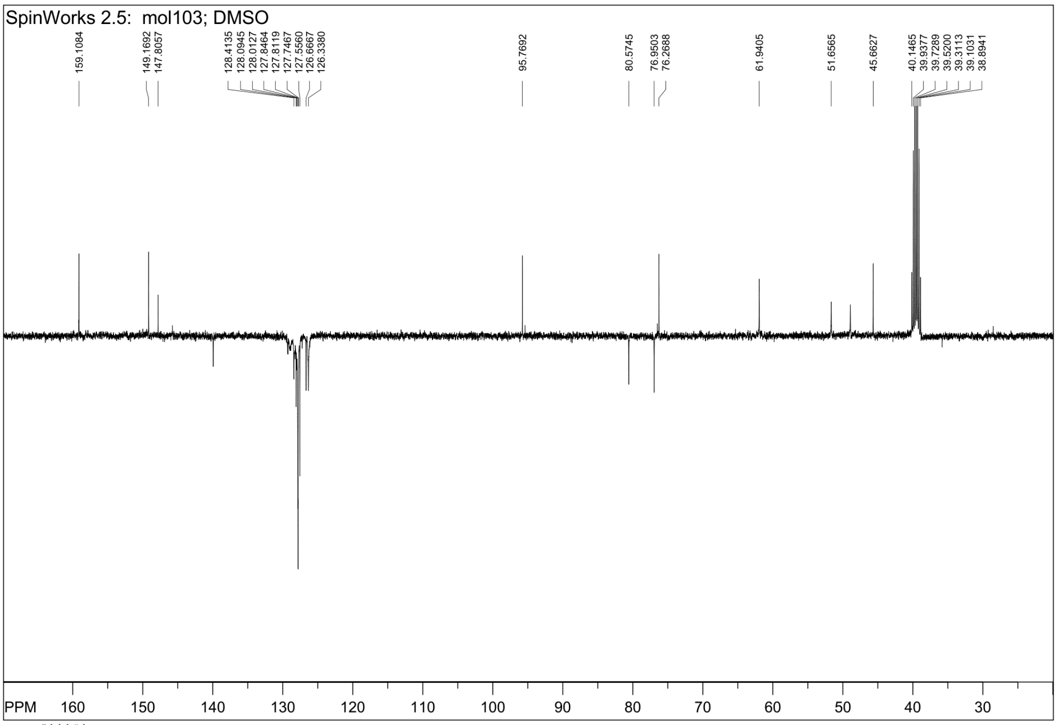

file: F:\ÑÒÀÒÜÈ ñ ïîèì ñîàâòîðñòâîì\2018\ïèðîîñôàòû ïîðôîëèíîâûå\NMR_Mass_HPLC\2g_HOMor(Tr)U(5-freq. of 0 ppm: 100.612812 MHz7\fid expt: <jmod>
transmitter freq.: 100.624339 MHz
time domain size: 65536 points
width: 24038.46 Hz = 238.893111 ppm = 0.366798 Hz/pt
number of scans: 256

processed size: 131072 complex points
LB: 0.000 GB: 0.0000
Hz/cm: 603.535 ppm/cm: 5.99790

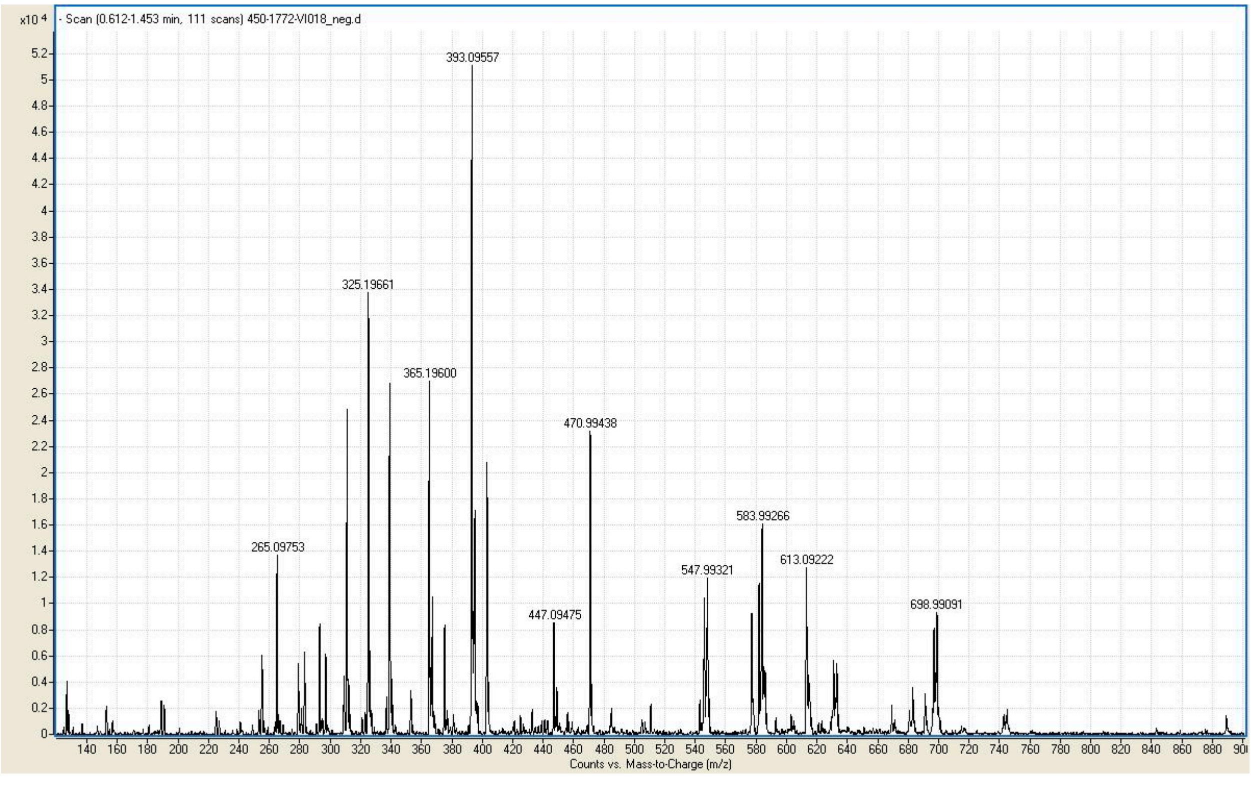

2'-Hydroxymethyl-4'-*N*-trityl-6'-(5-chlorouracil-1-yl)-morpholine (2ClU)

^{1}H (400 MHz, Pyridine-d_5): 8.04 (s, 1H, *H6*-Ura), 7.70-7.45 (m, 6H, Tr), 7.30 (br t, *J* 7.6, 6H, Tr), 7.15 (br t, *J* 6.6, 3H, Tr), 6.59 (dd, *J* 9.7, 2.1, 1H, *H6'*), 4.62-4.52 (m, 1H, *H2'*), 3.84 (qd, J 40.7, 5.2, 2H, *CH_2*-OH), 3.61 (dt, *J* 11.1, 2.1, 1H, *H5'*), 3.46 (dt, *J* 11.7, 2.1, 1H, *H3'*), 1.79 (br t, *J* 10.5, 1H, *H3'*), 1.74 (br t, *J* 10.9, 1H, *H5'*).

^{13}C (100 MHz, Pyridine-d_5): 160.46, 150.73, 138.16, 130.00, 128.61, 127.16, 109.29, 81.67, 79.12, 77.58, 63.57, 52.89, 50.45.

MS ESI (*m/z*): [M-H]$^-$ calcd for $C_{28}H_{25}ClN_3O_4^-$ 502.16; found 502.49.

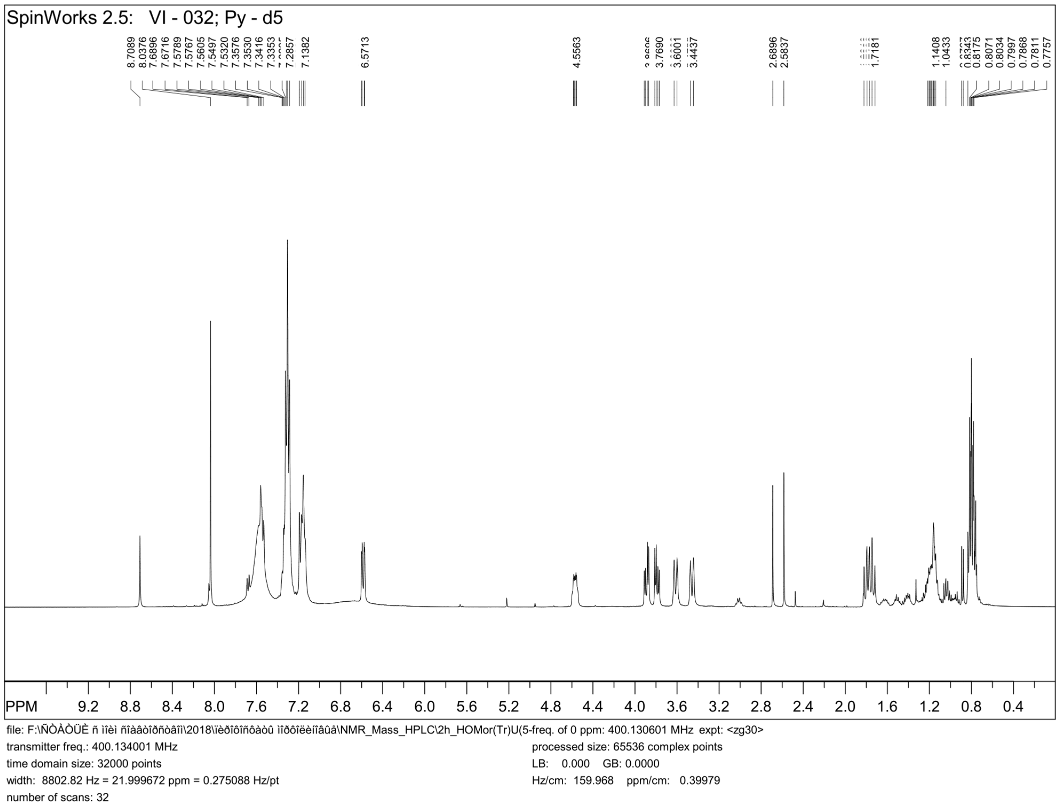

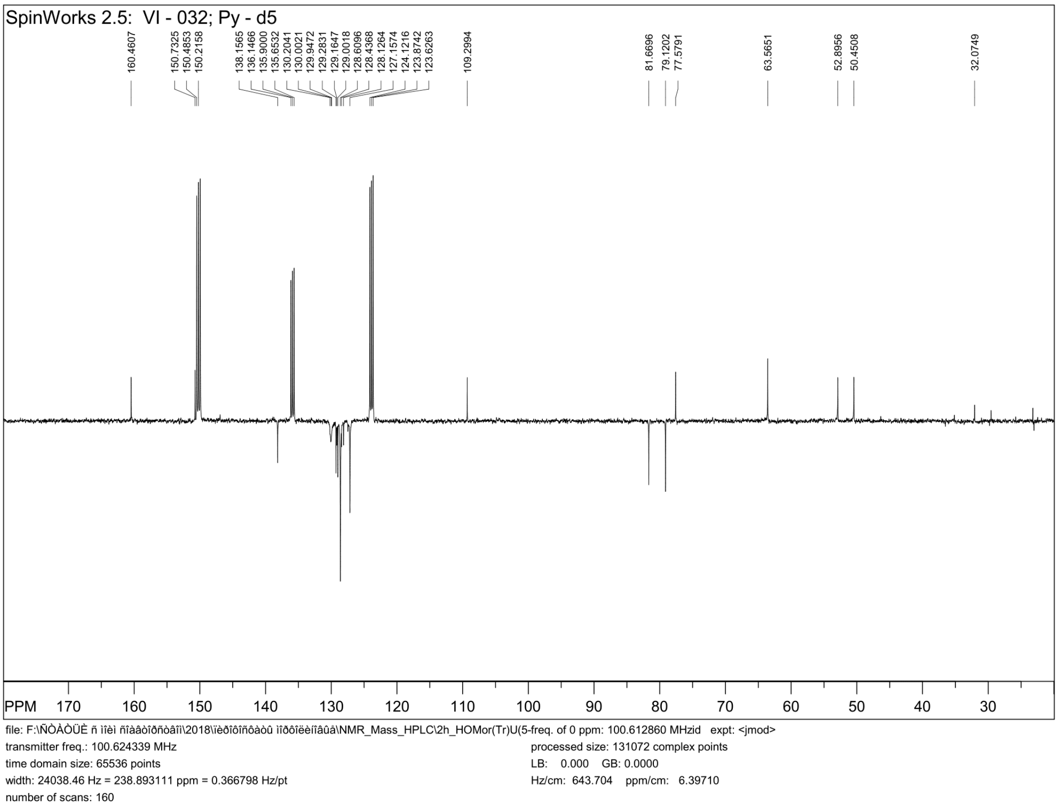
SpinWorks 2.5: VI - 032; Py - d5
160.4607
150.7325
150.4853
150.2158
138.1565
136.1466
135.9000
135.6532
130.2041
130.0021
129.9472
129.2831
129.1647
129.0018
128.6096
128.4368
128.1264
127.1574
124.1216
123.8742
123.6263
109.2994
81.6696
79.1202
77.5791
63.5651
52.8956
50.4508
32.0749
PPM 170 160 150 140 130 120 110 100 90 80 70 60 50 40 30
file: F:\ÑÒÀÒÜÈ ñ ìîèì ñîàâòîðñòâîì\2018\ìèðîòîíñòàòû ìîðôîëèíîâûå\NMR_Mass_HPLC\2h_HOMor(Tr)U(5-freq. of 0 ppm: 100.612860 MHzid expt: <jmod>
transmitter freq.: 100.624339 MHz
time domain size: 65536 points
width: 24038.46 Hz = 238.893111 ppm = 0.366798 Hz/pt
number of scans: 160
processed size: 131072 complex points
LB: 0.000 GB: 0.0000
Hz/cm: 643.704 ppm/cm: 6.39710

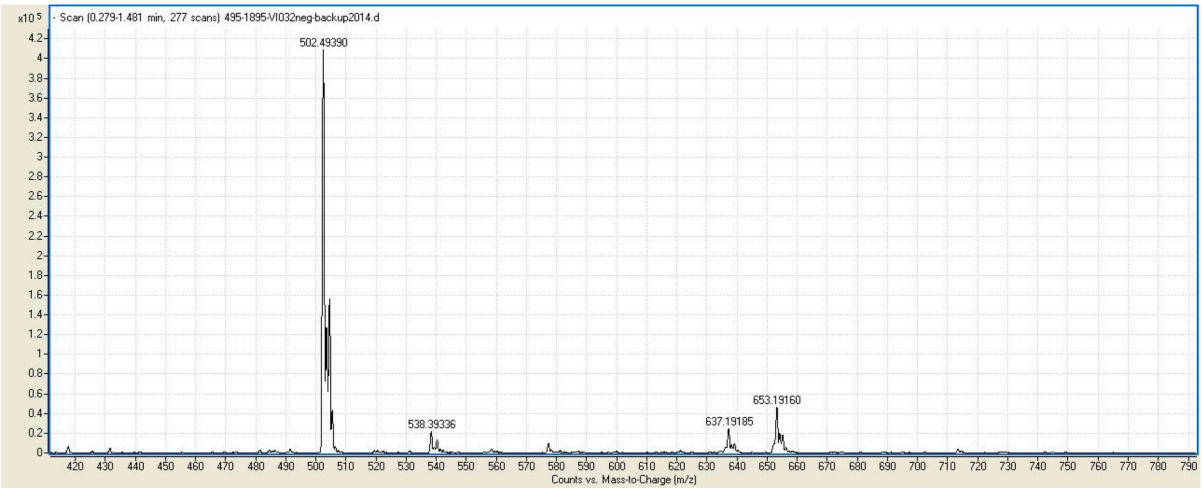
x10 5
- Scan (0.279-1.481 min, 277 scans) 495-1895-VI032neg-backup2014.d
502.49390
538.39336
637.19185
653.19160
Counts vs. Mass-to-Charge (m/z)

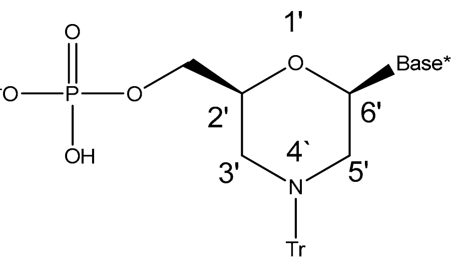

3A,G,C,U,T,IU,BrU,ClU

2’-*O*-Phosphomethyl-4’-*N*-trityl-6’-(*N*^{6}-benzoyladenine-9-yl)-morpholine (3A)

^{1}H (300 MHz, DMSO-d$_6$): 8.73 (s, 1H, *H8*-Ade), 8.45 (s, 1H, *H2*-Ade), 8.02 (dt, *J* 7.0, 1.5, 2H, *o*-Bz), 7.63 (tt, 7.3, 1.3, 1H, *p*-Bz) 7.57-7.41 (m, 8H, *m*-Bz, Tr), 7.33 (br t, *J* 7.4, 6H, Tr), 7.20 (br t, *J* 7.1, 3H, Tr), 6.40 (dd, *J* 10.3, 2.1, 1H, *H6’*), 4.49-4.36 (m, 1H, *H2’*), 3.77-3.34 (m, 3H, *CH$_2$*-Op, *H5’*), 3.37 (br d, *J* 11.7, 1H, *H3’*), 2.89 (q, *J* 7.1, 6H, (*CH$_2$*CH$_3$)$_3$N), 2.09 (br t, *J* 10.5, 1H, *H3’*), 1.46 (br t, *J* 11.0, 1H, *H5’*), 1.08 (t, *J* 7.2, 9H, (CH$_2$*CH$_3$*)$_3$N).

^{31}P (121 MHz, DMSO-d$_6$): 0.35 (s).

MS ESI (*m/z*): [M-H]$^-$ calcd for $C_{36}H_{32}N_6O_6P^-$ 675.212; found 674.991.

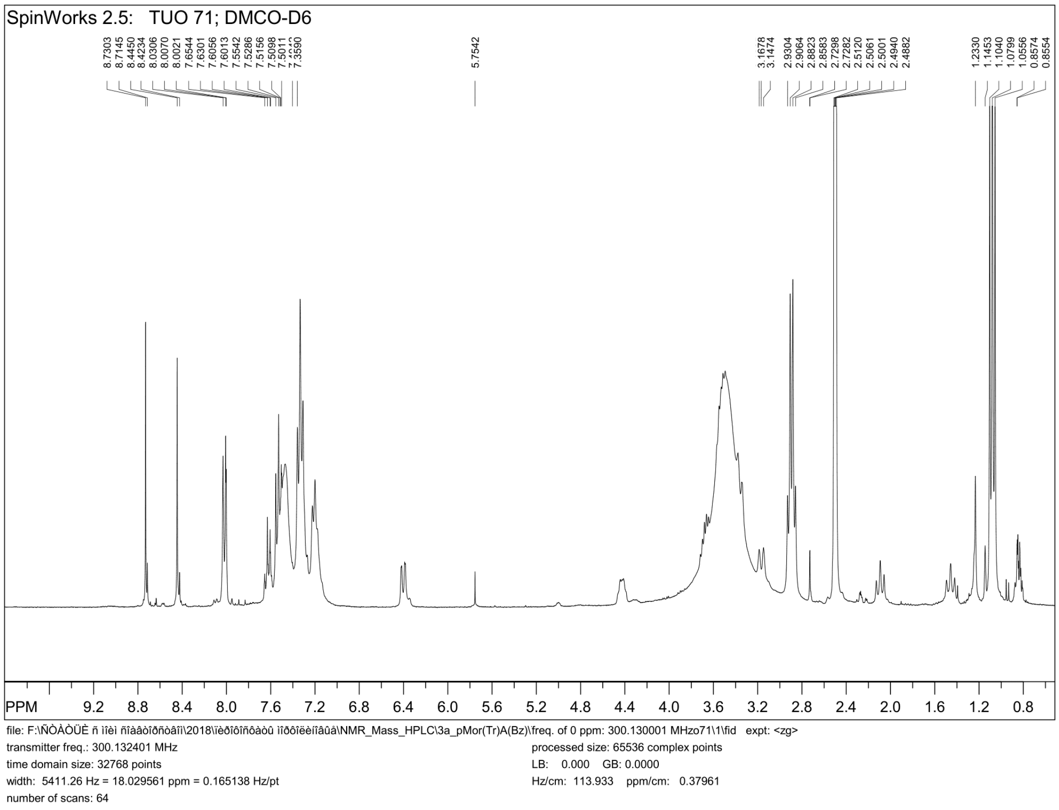

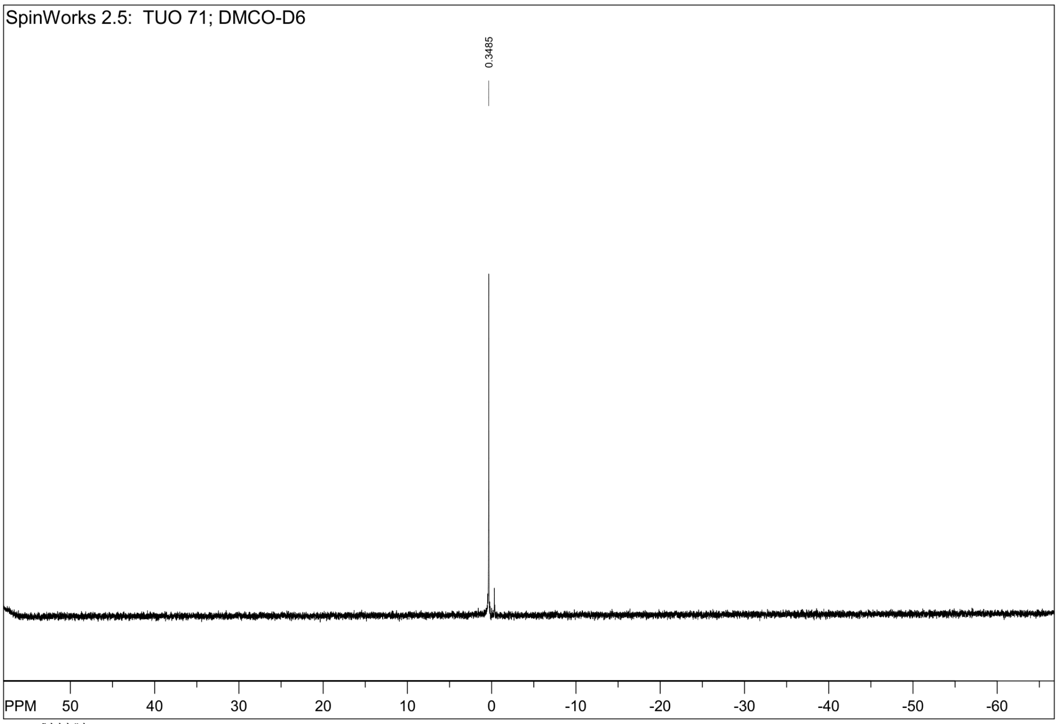

file: F:\ÑÒÀÒÜÈ ñ ìîèì ñîàâòîðñòâîì\2018\ïèðîôîñôàòû ìîðôîëèíîâûõ\NMR_Mass_HPLC\3a_pMor(Tr)A(Bz)\freq. of 0 ppm: 121.494734 MHzo71\31\fid expt: <zgpg>
transmitter freq.: 121.494197 MHz
time domain size: 32768 points
width: 15151.52 Hz = 124.709784 ppm = 0.462388 Hz/pt
number of scans: 112

processed size: 65536 complex points
LB: 0.000 GB: 0.0000
Hz/cm: 606.063 ppm/cm: 4.98841

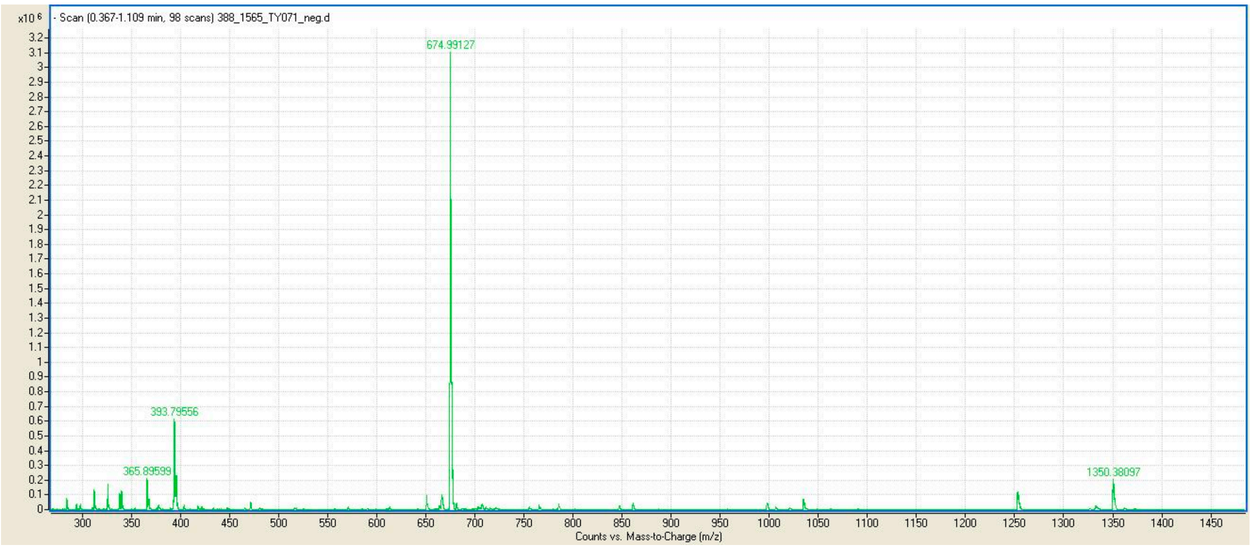

2'-*O*-Phosphomethyl-4'-*N*-trityl-6'-(N^2-isobutyrylguanine-9-yl)-morpholine (3G)

^{1}H (300 MHz, $CDCl_3$): 12.30 (s, 1H, *NH*), 11.00 (br s, 1H, *NH*), 7.54-7.01 (m, 16H, *H8*-Gua, Tr), 5.88-5.76 (m, 1H, *H6'*), 4.39-4.24 (m, 1H, *H2'*), 4.00-3.71 (m, 2H, CH_2-Op), 3.34-3.13 (m, 2H, *H5'*, *H3'*), 2.96 (q, *J* 7.2, 6H, (CH_2CH_3)$_3$N), 2.85-2.74 (m, 1H, *CH*-*i*Bu), 1.82 (br t, *J* 10.4, 1H, *H3'*), 1.58-1.46 (m, 1H, *H5'*), 1.29-1.06 (m, 15H, (CH_2CH_3)$_3$N, 2CH_3-*i*Bu).

^{31}P (121 MHz, $CDCl_3$): 1.69 (s).

MS ESI (*m/z*): [M-H]$^-$ calcd for $C_{33}H_{34}N_6O_7P^-$ 657.223; found 657.092.

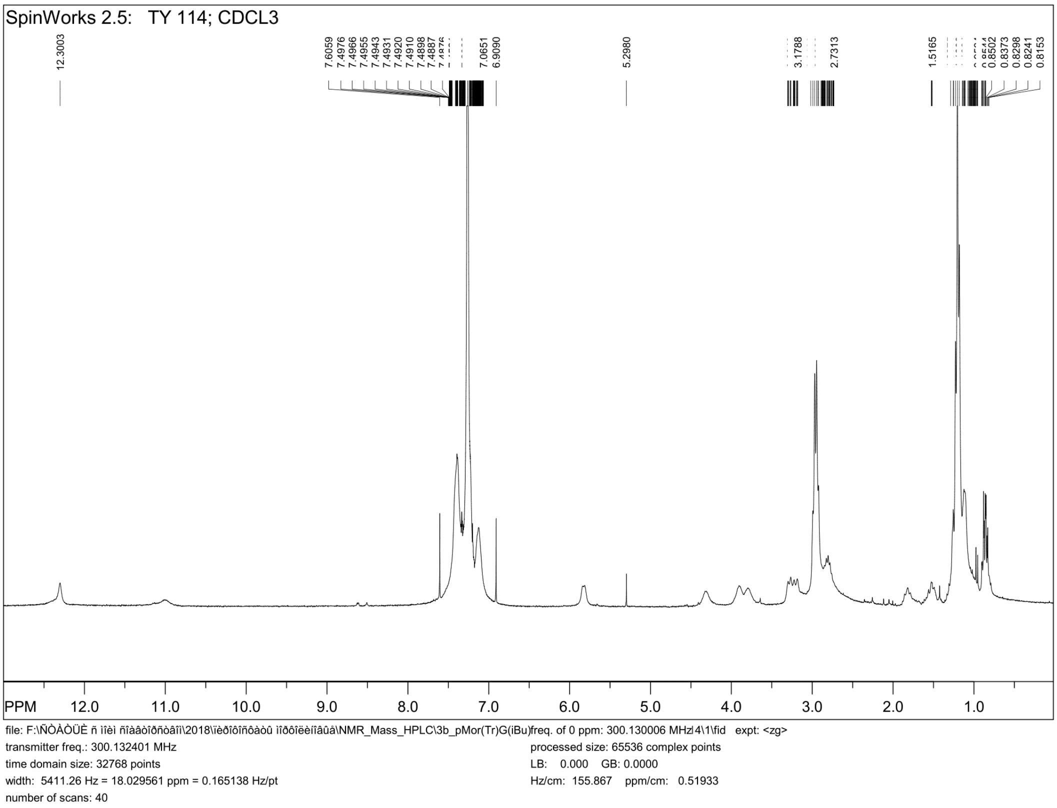

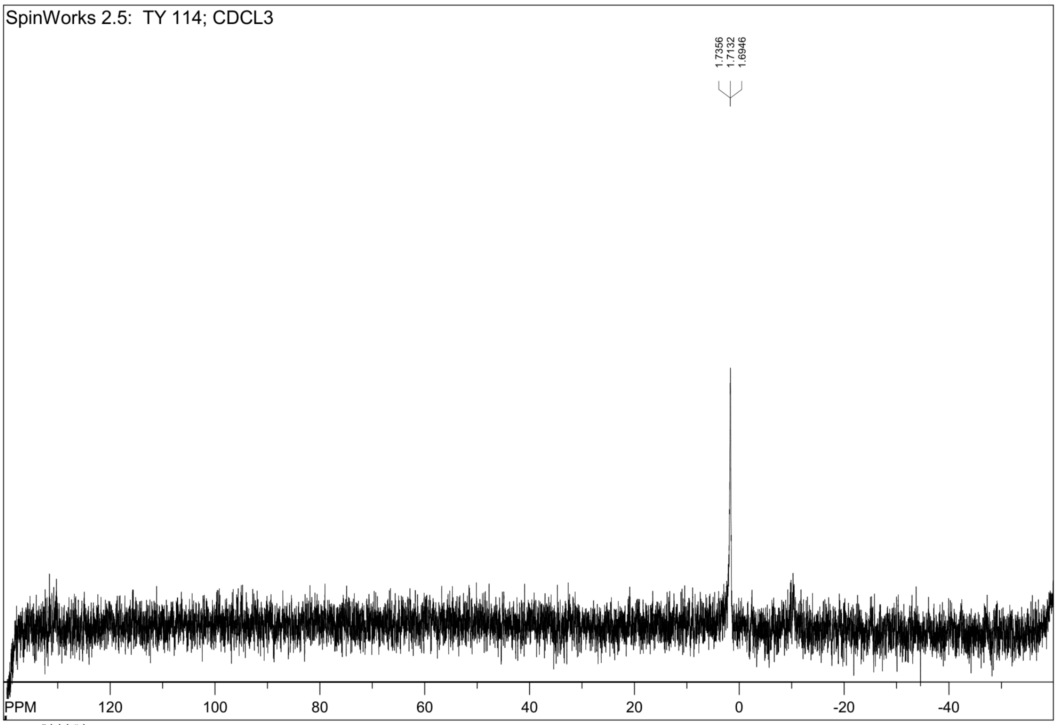

file: F:\ÑÒÀÒÜÈ ñ ìîèì ñîàâòîðñòâîì\2018\ïèðîîíñôàòû ìîðôîëèíîâûå\NMR_Mass_HPLC\3b_pMor(Tr)G(iBu)freq. of 0 ppm: 121.494824 MHz\4\31\fid expt: <zgpg>
transmitter freq.: 121.499711 MHz
time domain size: 32768 points
width: 24330.90 Hz = 200.254800 ppm = 0.742520 Hz/pt
number of scans: 80
processed size: 65536 complex points
LB: 0.000 GB: 0.0000
Hz/cm: 973.236 ppm/cm: 8.01019

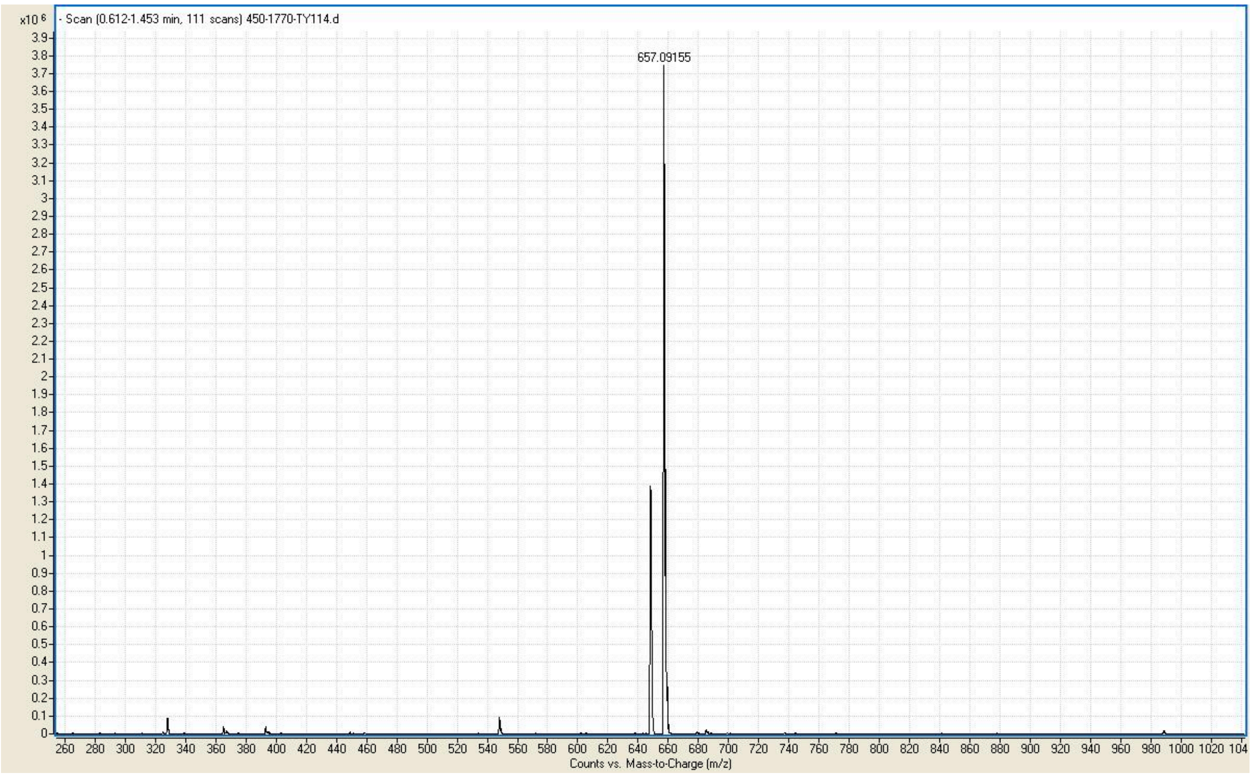

2'-*O*-Phosphomethyl-4'-*N*-trityl-6'-(N^4-benzoylcytosine-1-yl)-morpholine (3C)

^{1}H (300 MHz, DMSO-d_6): 7.95 (dt, *J* 7.0, 1.2, 2H, *o*-Bz), 7.92 (d, *J* 7.5, 1H, *H6*-Cyt), 7.61 (tt, J 7.3, 1.2, 1H, *m*-Bz), 7.49 (tt, *J* 7.5, 1.3, 2H, *p*-Bz), 7.46-7.38 (m, 6H, Tr), 7.32 (br t, *J* 7.4, 6H, Tr), 7.24 (d, *J* 7.6, 1H, *H5*-Cyt), 7.20 (br t, *J* 7.4, 3H, Tr), 6.11 (dd, *J* 9.4, 2.0, H6'), 4.40-4.30 (m, 1H, H2'), 3.75-3.65 (m, 2H, CH_2-Op), 3.34 (br d, *J* 10.5, 1H, H5'), 3.11 (br d, *J* 11.6, 1H, H3'), 2.91 (q, *J* 7.2, 6H, $(CH_2CH_3)_3N$), 1.37 (br t, *J* 11.2, 1H, *H3'*), 1.25 (br t, *J* 10.1, 1H, *H5'*), 1.11 (t, *J* 7.2, 9H, $(CH_2CH_3)_3N$).

^{31}P (121 MHz, DMSO-d_6): 0.43 (s).

MS ESI (*m/z*): [M-H]$^-$ calcd for $C_{35}H_{32}N_4O_7P^-$ 651.201; found 650.992.

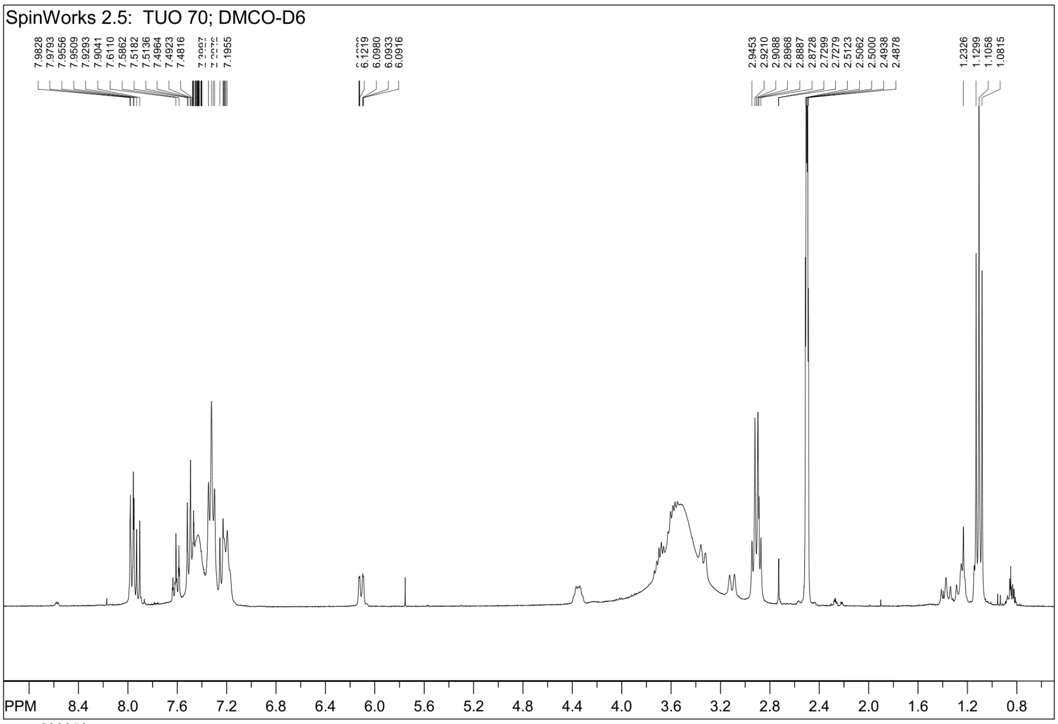

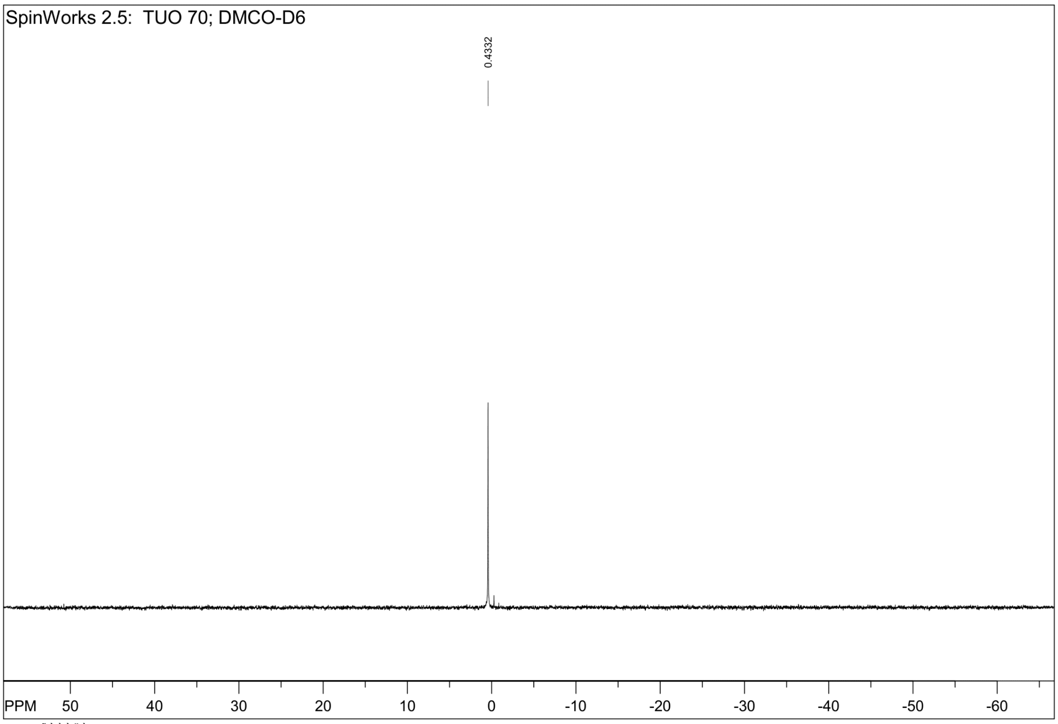

file: F:\ÑÒÀÒÜÈ ñ ìîèì ñîàâòîðñòâîì\2018\ïèðîôîñôàòû ïîðôîëèíîâûå\NMR_Mass_HPLC\3c_pMor(Tr)C(Bz)\freq. of 0 ppm: 121.494734 MHzo70\31\fid expt: <zgpg>
transmitter freq.: 121.494197 MHz
time domain size: 32768 points
width: 15151.52 Hz = 124.709784 ppm = 0.462388 Hz/pt
number of scans: 120
processed size: 65536 complex points
LB: 0.000 GB: 0.0000
Hz/cm: 606.063 ppm/cm: 4.98841

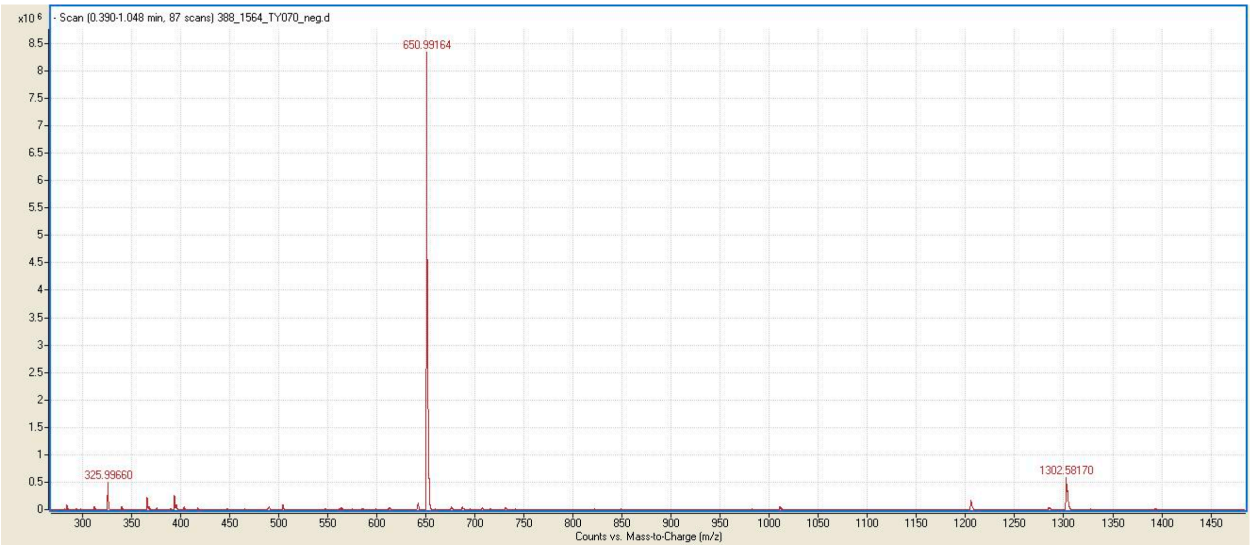

2’-*O*-Phosphomethyl-4’-*N*-trityl-6’-(uracil-1-yl)-morpholine (3U)

^{1}H (300 MHz, CD_3CN-d_3): 7.50-7.39 (m, 6H, Tr), 7.43 (d, *J* 8.1, 1H, *H6*-Ura), 7.27 (br t, *J* 7.2, 6H, Tr), 7.17 (br t, *J* 7.4, 3H, Tr), 6.02 (dd, *J* 9.7, 2.3, 1H, *H6’*), 5.57 (d, *J* 8.2, 1H, *H5*-Ura), 4.41-4.31 (m, 1H, *H2’*), 3.71-3.64 (m, 2H, *CH_2*-Op), 3.23 (dt, *J* 11.5, 2.3, 1H, *H5’*), 3.06 (q, *J* 7.2, 7H, (CH_2CH_3)$_3$N, *H3’*), 1.43-1.28 (m, 2H, *H3’*, *H5’*), 1.17 (t, J 7.3, 9H, (CH_2CH_3)$_3$N).

^{31}P (121 MHz, CD_3CN-d_3): -0.11 (s).

MS ESI (*m/z*): $[M\text{-}H]^-$ calcd for $C_{28}H_{27}N_3O_7P^-$ 548.159; found 548.093.

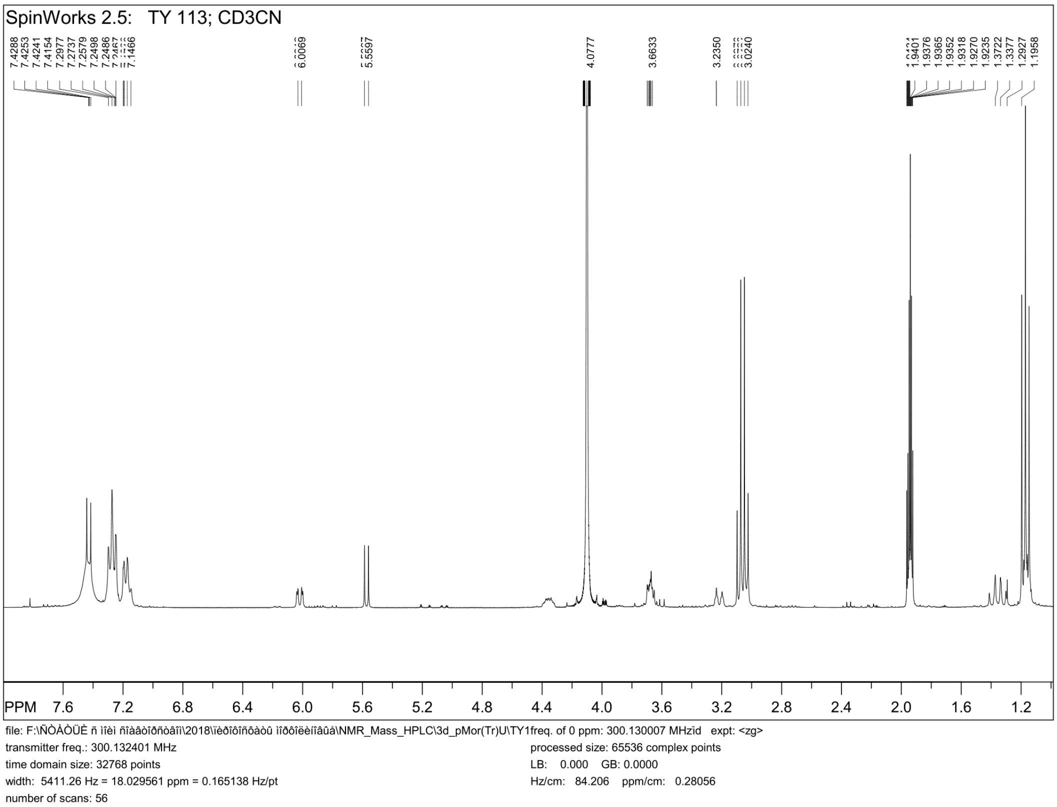

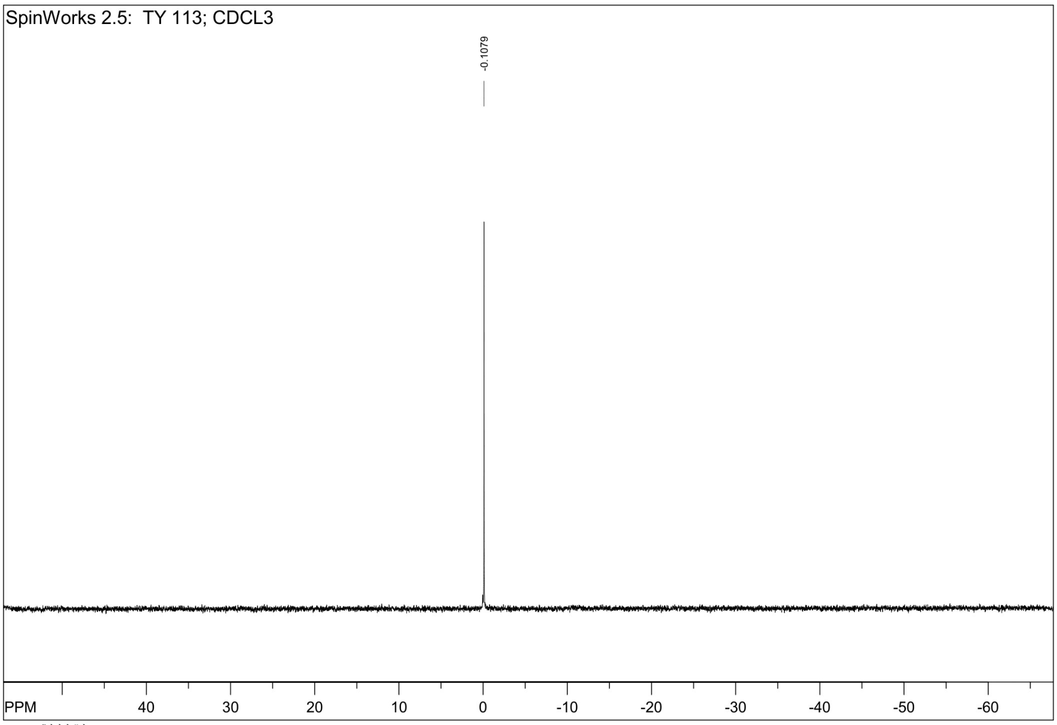

file: F:\ÑÒÀÒÜÈ ñ ìîèì ñîàâòîðñòâîì\2018\ïèðîôîñôàòû ìîðôîëèíîâûà\NMR_Mass_HPLC\3d_pMor(Tr)U\TY1freq. of 0 ppm: 121.494851 MHz\fid   expt: <zgpg>
transmitter freq.: 121.494197 MHz
time domain size: 32768 points
width: 15151.52 Hz = 124.709784 ppm = 0.462388 Hz/pt
number of scans: 32
processed size: 65536 complex points
LB: 0.000 GB: 0.0000
Hz/cm: 606.064 ppm/cm: 4.98842

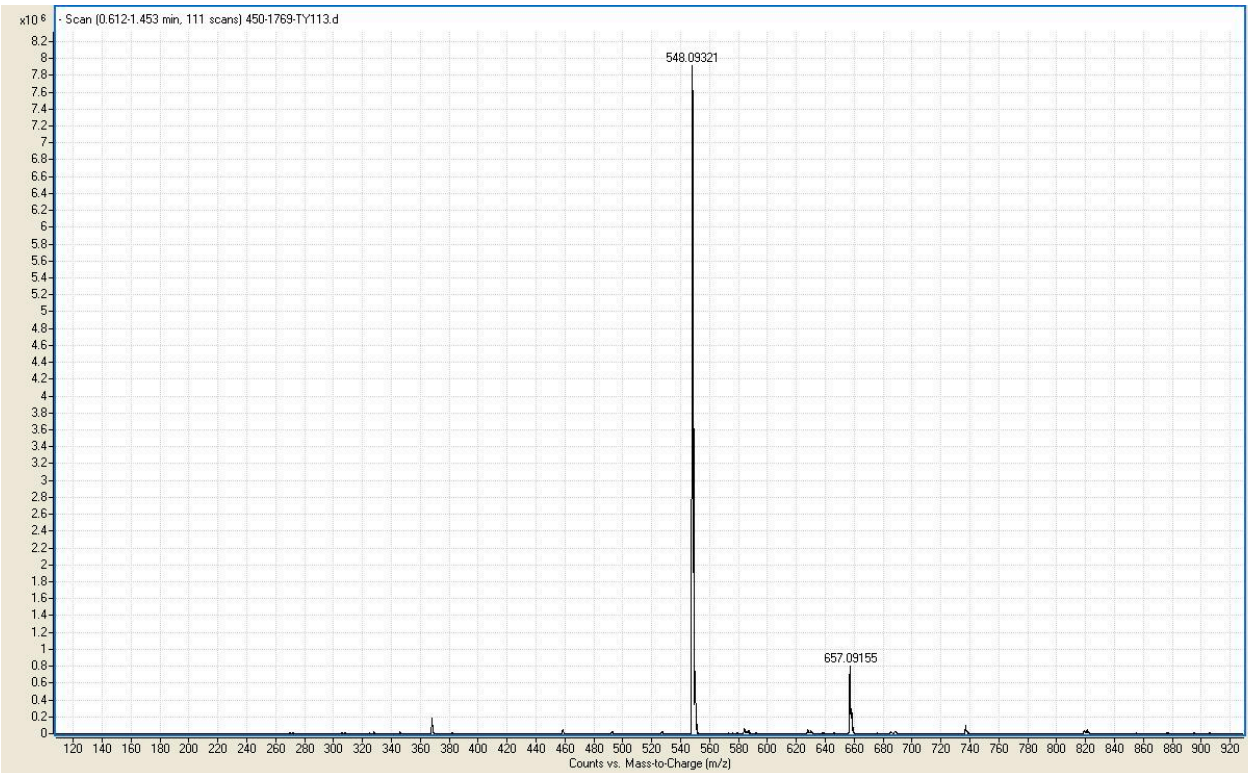

2’-*O*-Phosphomethyl-4’-*N*-trityl-6’-(thymine-1-yl)-morpholine (3T)

^{1}H (300 MHz, DMSO-d_6): 7.59-7.45 (m, 6H, Tr), 7.32 (br t, *J* 7.2, 6H, Tr), 7.25 (d, *J* 1.1, 1H, *H6*-Ura), 7.19 (br t, *J* 6.7, 3H, Tr), 5.99 (dd, *J* 9.6, 2.3, 1H, *H6’*), 4.35-4.23 (m, 1H, *H2’*), 3.69-3.59 (m, 2H, *CH_2*-Op), 3.13 (br d, *J* 11.3, 1H, *H5’*), 3.05 (br d, *J* 11.3, 1H, *H3’*), 2.90 (q, *J* 6.5, 6H, ($CH_2CH_3)_3$N), 1.67 (br d, *J* 0.9, 3H, *CH_3*-Thy), 1.44-1.27 (m, 2H, *H3’*, *H5’*), 1.10 (t, J 7.3, 9H, ($CH_2CH_3)_3$N).

^{31}P (121 MHz, DMSO-d_6): 0.33 (s).

MS ESI (*m/z*): [M-H]$^-$ calcd for $C_{29}H_{29}N_3O_7P^-$ 562.174; found 561.893.

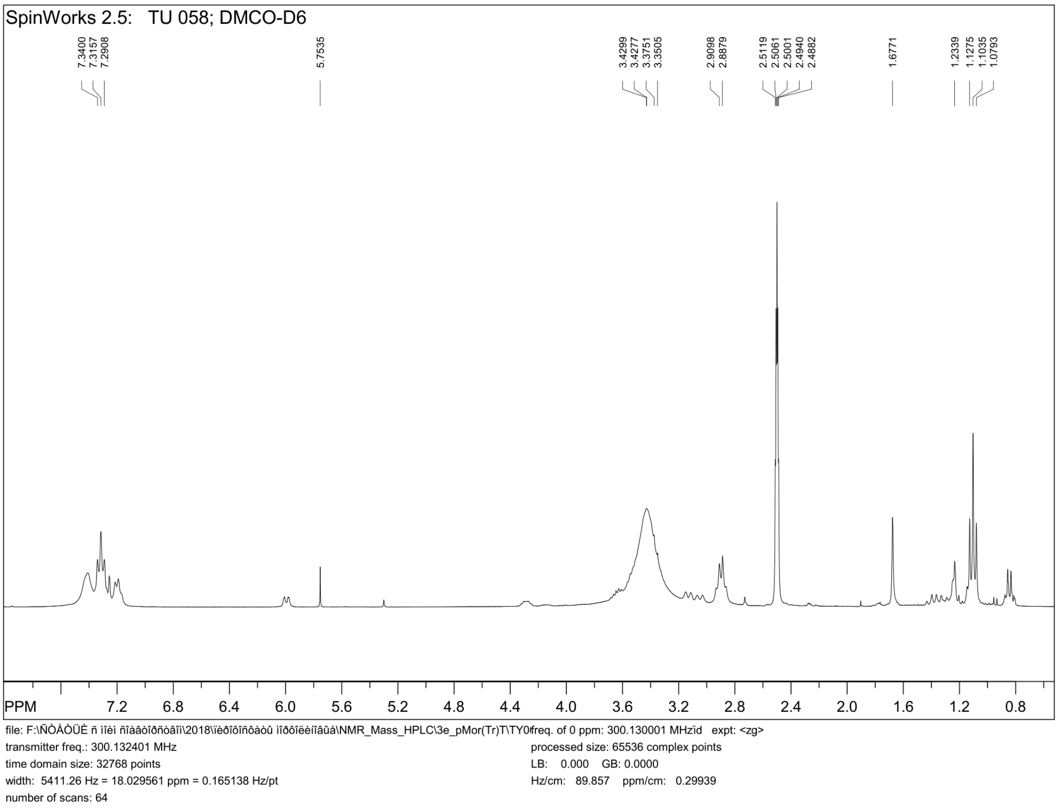

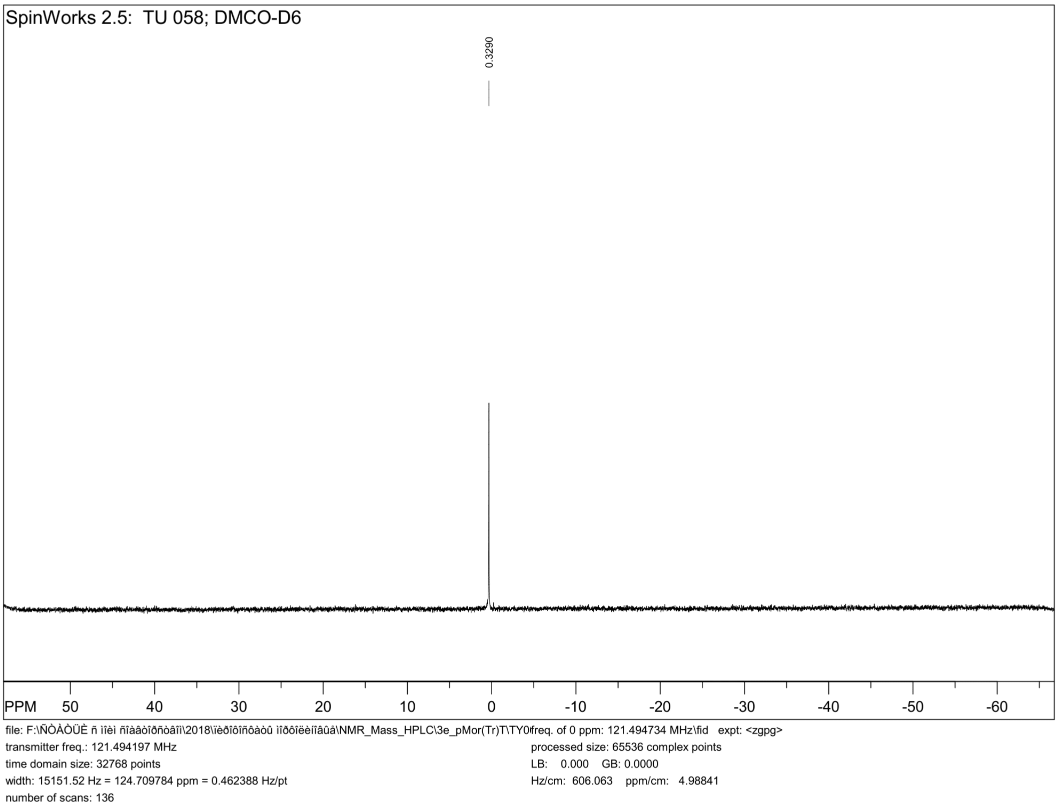
SpinWorks 2.5: TU 058; DMCO-D6
0.3290
PPM 50 40 30 20 10 0 -10 -20 -30 -40 -50 -60
file: F:\ÑÒÀÒÜÈ ñ ìîèì ñîàâòîðñòâîì\2018\ïèðîôîñôàòû ìîðôîëèíîâûå\NMR_Mass_HPLC\3e_pMor(Tr)T\TY0freq. of 0 ppm: 121.494734 MHz\fid expt: <zgpg>
transmitter freq.: 121.494197 MHz
time domain size: 32768 points
width: 15151.52 Hz = 124.709784 ppm = 0.462388 Hz/pt
number of scans: 136
processed size: 65536 complex points
LB: 0.000 GB: 0.0000
Hz/cm: 606.063 ppm/cm: 4.98841

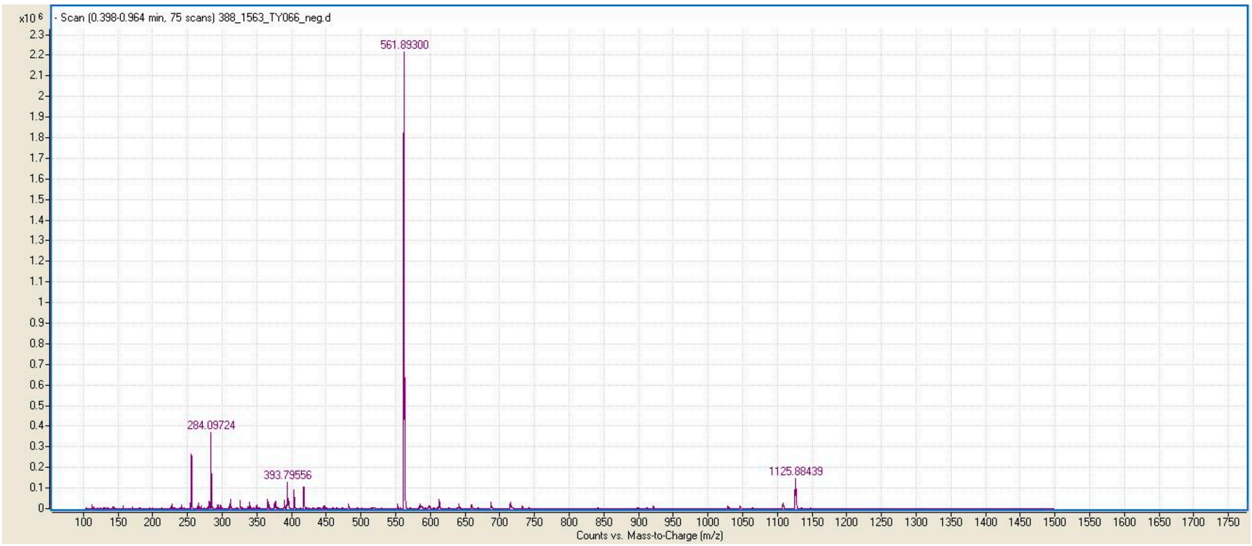
x10 6
- Scan (0.398-0.964 min, 75 scans) 388_1563_TY066_neg.d
561.89300
284.09724
393.79556
1125.88439
Counts vs. Mass-to-Charge (m/z)

2’-*O*-Phosphomethyl-4’-*N*-trityl-6’-(5-iodouracil-1-yl)-morpholine (3IU)

^{1}H (300 MHz, DMSO-d$_6$): 8.35 (s, 1H, *H6*-Ura), 8.08-8.91 (m, 6H, Tr), 7.83 (br t, *J* 7.2, 6H, Tr), 7.73 (br t, *J* 6.9, 3H, Tr), 6.56 (dd, *J* 9.6, 1.9, 1H, *H6’*), 4.97-4.86 (m, 1H, *H2’*), 4.24 (br t, *J* 5.8, 2H, *CH$_2$*-Op), 3.76 (br d, *J* 11.6, 1H, *H5’*), 3.62 (q, *J* 7.3, 7H, (*CH$_2$*CH$_3$)$_3$N), *H3’*), 2.00-1.75 (m, 2H, *H3’*, *H5’*), 1.74 (t, *J* 7.3. 9H, (CH$_2$*CH$_3$*)$_3$N).

^{31}P (121 MHz, DMSO-d$_6$): 0.93 (s)

MS ESI (*m/z*): $[M\text{-}H]^-$ calcd for $C_{28}H_{26}IN_3O_7P^-$ 674.06; found 673.99.

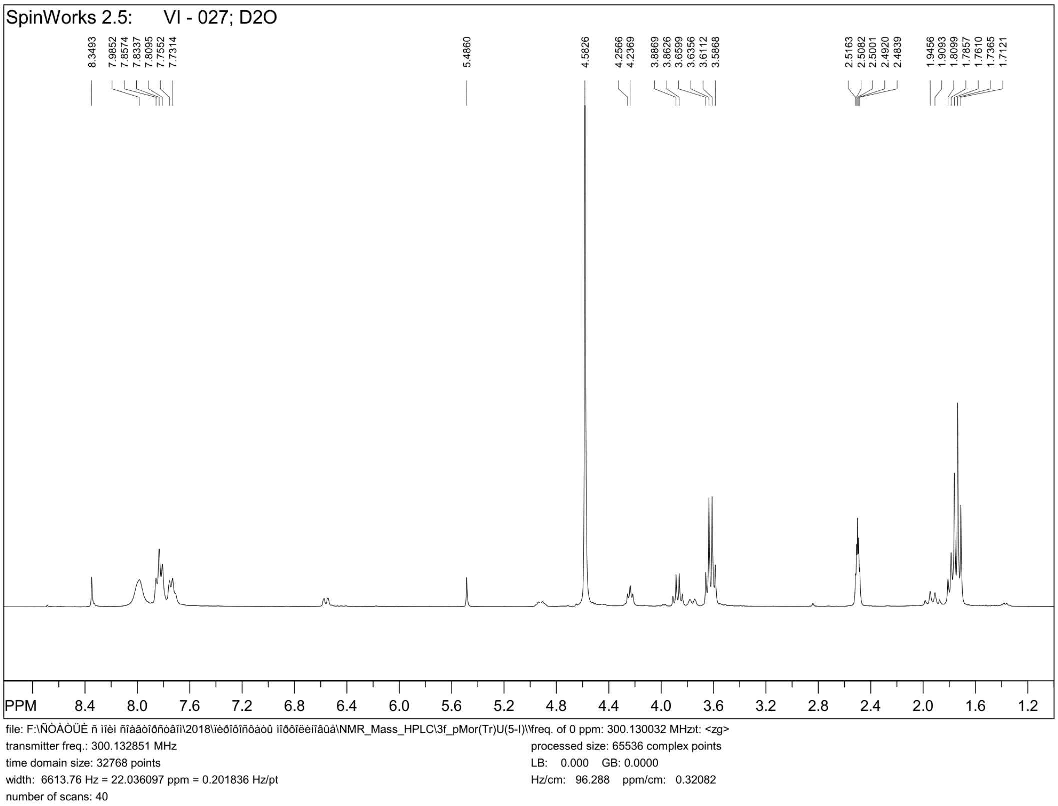

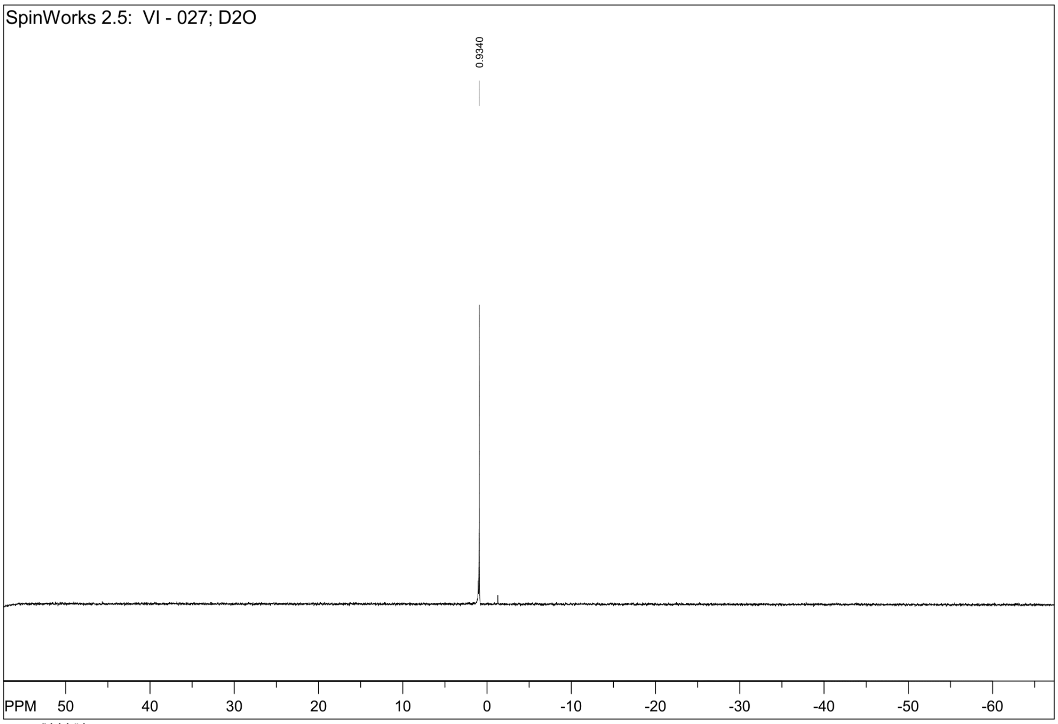

file: F:\ÑÒÀÒÜÈ ñ ìîèì ñîàâòîðñòâîì\2018\ïèðîôîñôàòû ìîðôîëèíîâûå\NMR_Mass_HPLC\3f_pMor(Tr)U(5-I)\\freq. of 0 ppm: 121.494801 MHz;pt: <zgpg>
transmitter freq.: 121.494197 MHz | processed size: 65536 complex points
time domain size: 32768 points | LB: 0.000 GB: 0.0000
width: 15151.52 Hz = 124.709784 ppm = 0.462388 Hz/pt | Hz/cm: 606.064 ppm/cm: 4.98842
number of scans: 128

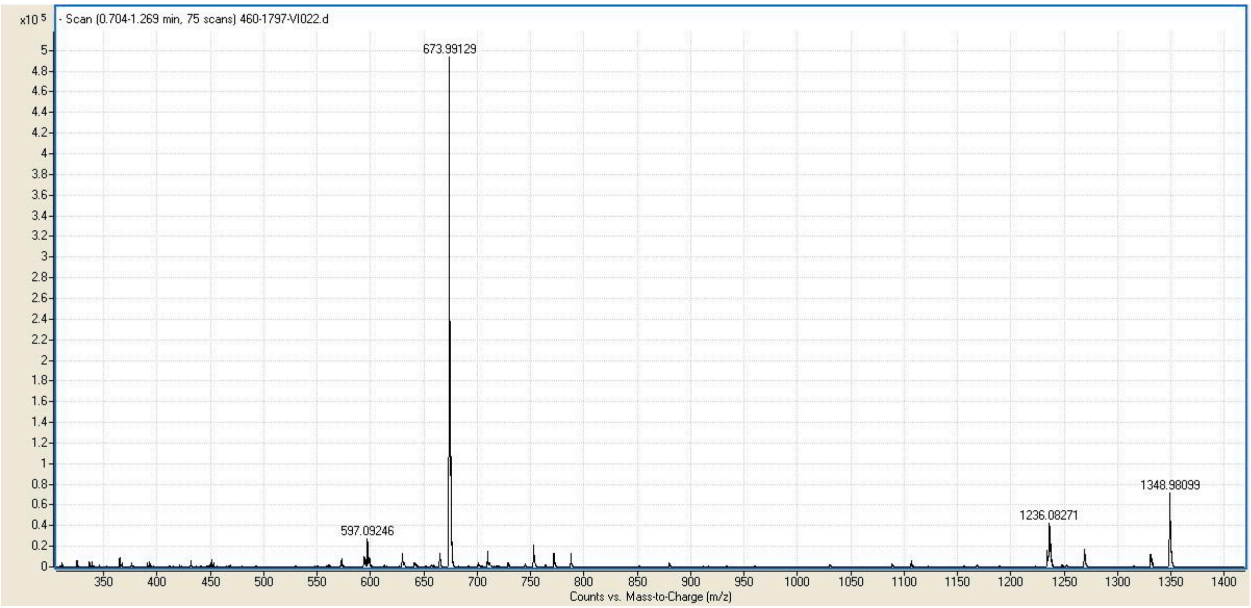

2'-*O*-Phosphomethyl-4'-*N*-trityl-6'-(5-bromouracil-1-yl)-morpholine (3BrU)

^{1}H (300 MHz, DMSO-d$_6$): 7.77 (s, 1H, *H6*-Ura), 7.47-7.36 (m, 6H, Tr), 7.30 (br t, *J* 7.3, 6H, Tr), 7.18 (br t, *J* 6.5, 3H, Tr), 5.95 (dd, *J* 9.3, 1.8, 1H, *H6'*), 4.37-4.34 (m, 1H, *H2'*), 3.63-3.32 (m, 3H, *CH$_2$*-Op, *H5'*), 3.20 (br d, *J* 11.4, 1H, *H3'*), 2.96 (q, *J* 7.2, 6H, (*CH$_2$*CH$_3$)$_3$N)), 1.43-1.25 (m, *2*H, *H3'*, *H5'*), 1.12 (t, *J* 7.2. 9H, (CH$_2$*CH$_3$*)$_3$N).

^{31}P (121 MHz, DMSO-d$_6$): -0.02 (s)

MS ESI (*m/z*): [M+H]$^+$ calcd for $C_{28}H_{28}BrN_3O_7P^+$ 628.084; found 628.092.

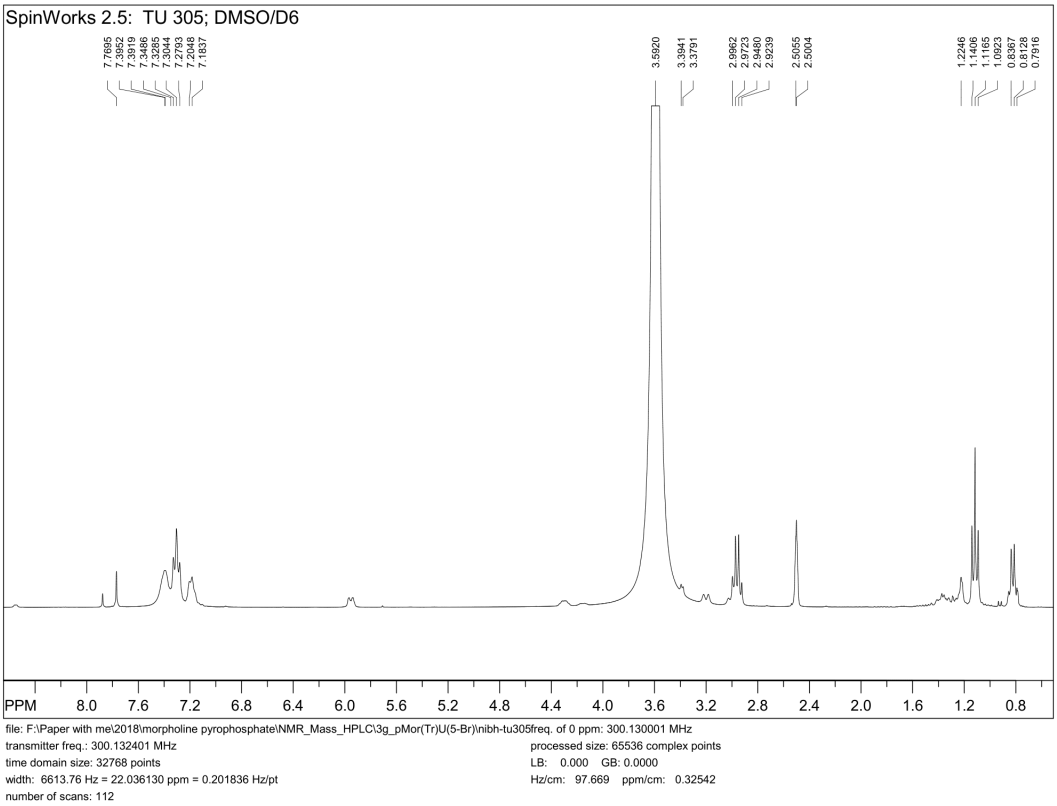

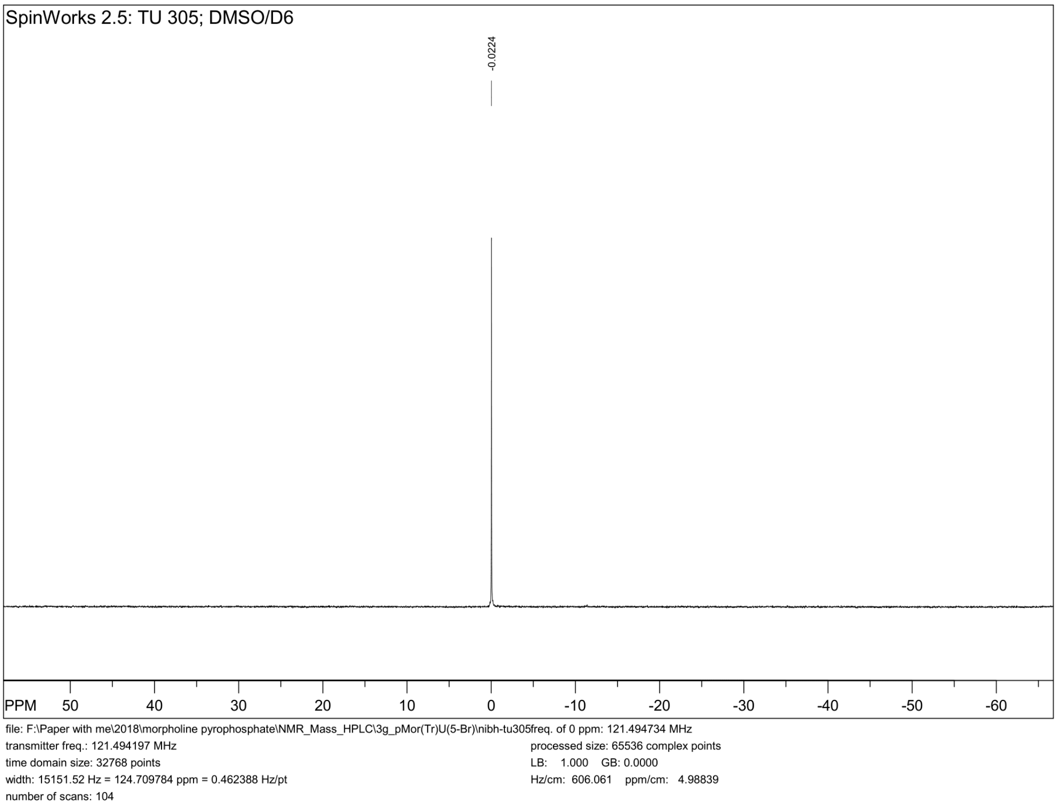

SpinWorks 2.5: TU 305; DMSO/D6
-0.0224
PPM 50 40 30 20 10 0 -10 -20 -30 -40 -50 -60
file: F:\Paper with me\2018\morpholine pyrophosphate\NMR_Mass_HPLC\3g_pMor(Tr)U(5-Br)\nibh-tu305
freq. of 0 ppm: 121.494734 MHz
transmitter freq.: 121.494197 MHz
processed size: 65536 complex points
time domain size: 32768 points
LB: 1.000 GB: 0.0000
width: 15151.52 Hz = 124.709784 ppm = 0.462388 Hz/pt
Hz/cm: 606.061 ppm/cm: 4.98839
number of scans: 104

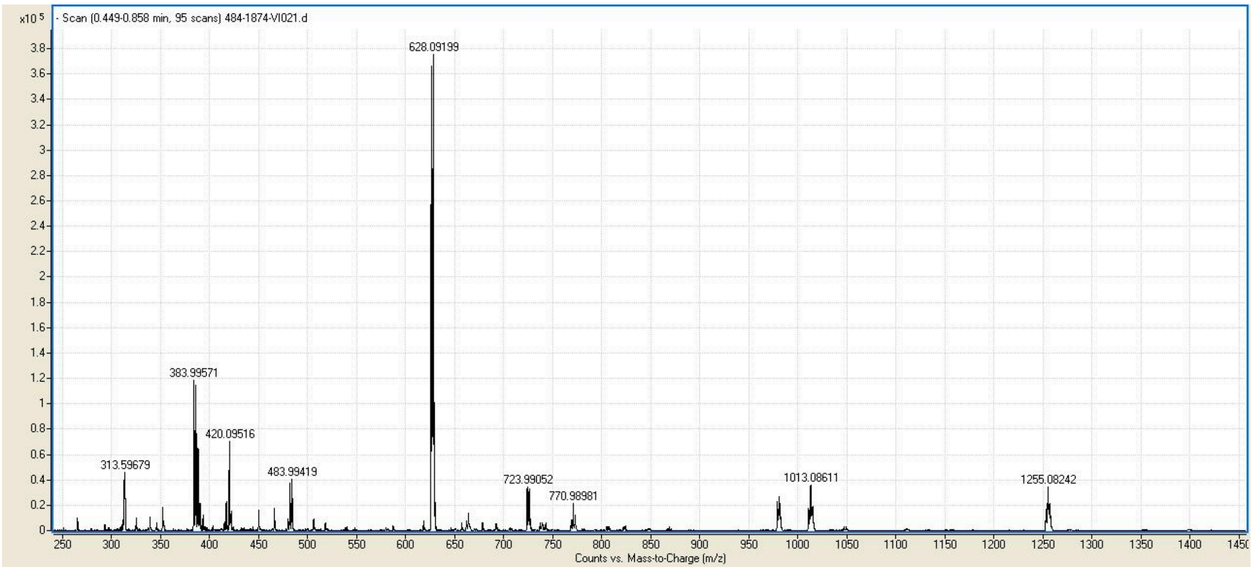

x10 5
- Scan (0.449-0.858 min, 95 scans) 484-1874-VI021.d
628.09199
383.99571
420.09516
313.59679
483.99419
723.99052
770.98981
1013.08611
1255.08242
Counts vs. Mass-to-Charge (m/z)

2’-*O*-Phosphomethyl-4’-*N*-trityl-6’-(5-chlorouracil-1-yl)-morpholine (3ClU)

[1]H (CD_3CN): 7.52-7.36 (m, 6H, Tr), 7.43 (s, 1H, *H6*-Ura), 7.27 (br t, *J* 7.6, 6H, Tr), 7.16 (br t, *J* 7.2, 3H, Tr), 6.04 (dd, *J* 9.9, 1.9, 1H, *H6’*), 4.53-4.51 (m, 1H, *H2’*), 3.61-3.48 (m, 2H, *CH₂*-OH), 3.13 (br d, *J* 11.8, 1H, *H5’*), 3.02 (br d, *J* 11.8, 1H, *H3’*), 1.39 (br t, *J* 10.5, 1H, *H3’*), 1.32 (br t, *J* 11.2, 1H, *H5’*).

[31]P (CD_3CN): 4.71 (s)

MS ESI (*m/z*): $[M\text{-}H]^-$ calcd for $C_{28}H_{26}ClN_3O_7P^-$ 582.120; found 582.192.

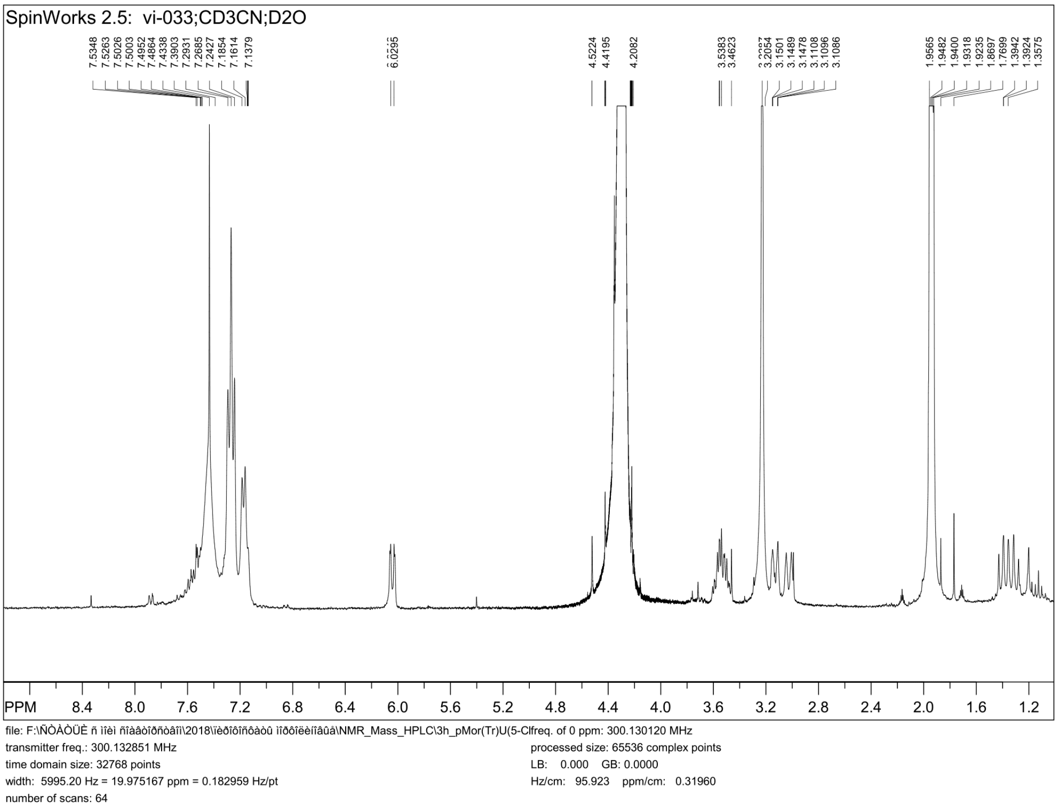

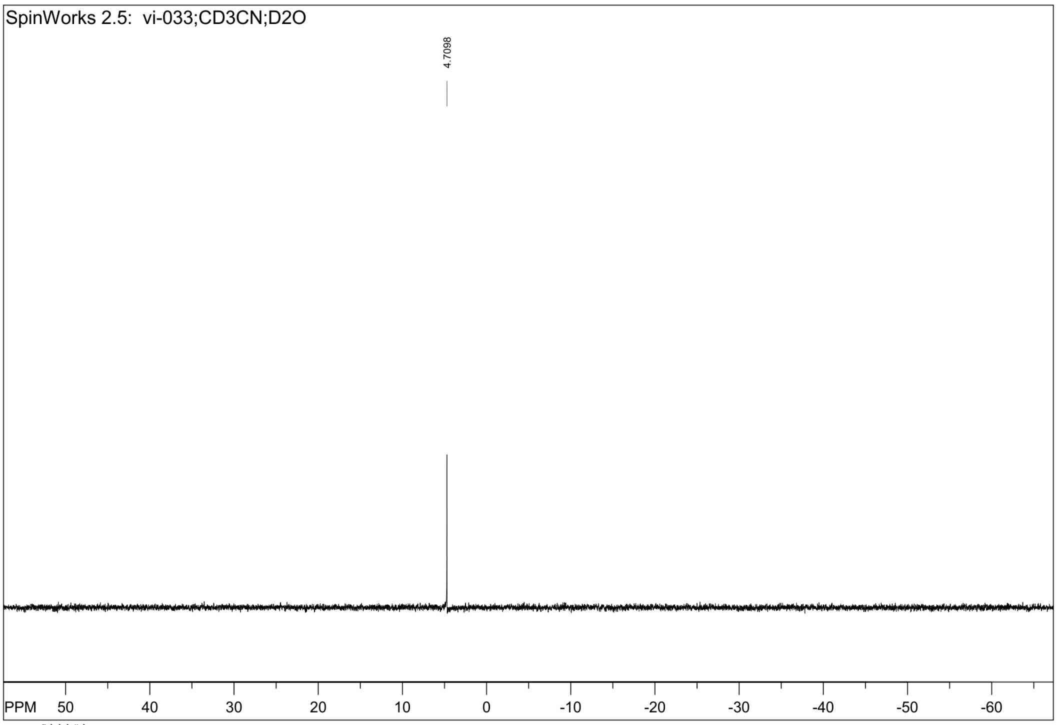

file: F:\ÑÒÀÒÜÈ ñ ìîèì ñîàâòîðñòâîì\2018\ïèðîôîñôàòû ìîðôîëèíîâûå\NMR_Mass_HPLC\3h_pMor(Tr)U(5-Clfreq. of 0 ppm: 121.494801 MHz>
transmitter freq.: 121.494197 MHz
time domain size: 32768 points
width: 15151.52 Hz = 124.709784 ppm = 0.462388 Hz/pt
number of scans: 40

processed size: 65536 complex points
LB: 0.000 GB: 0.0000
Hz/cm: 606.064 ppm/cm: 4.98842

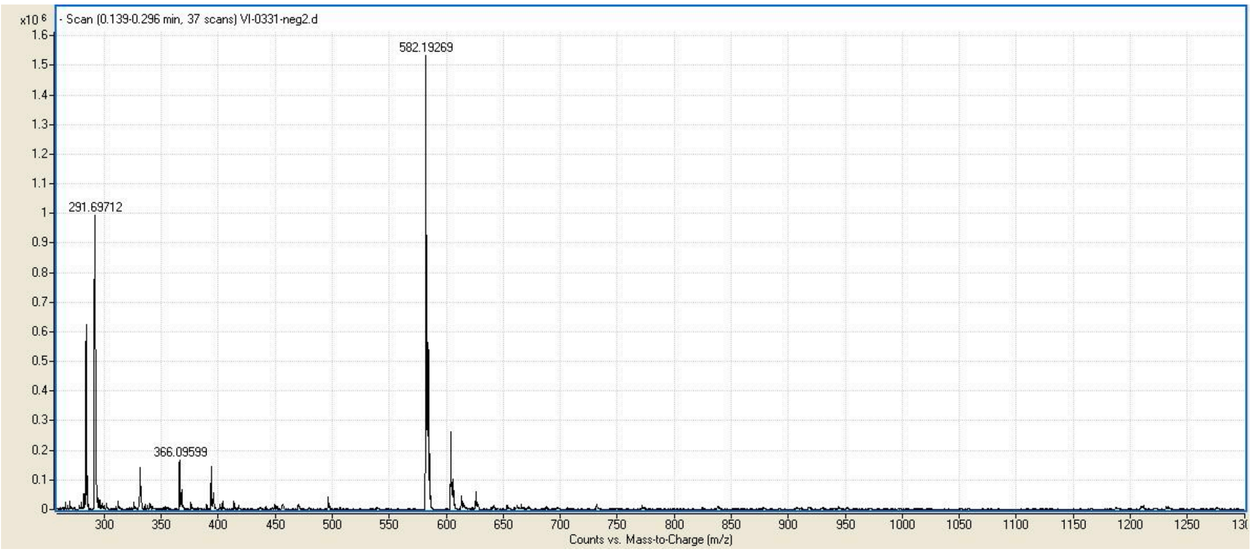

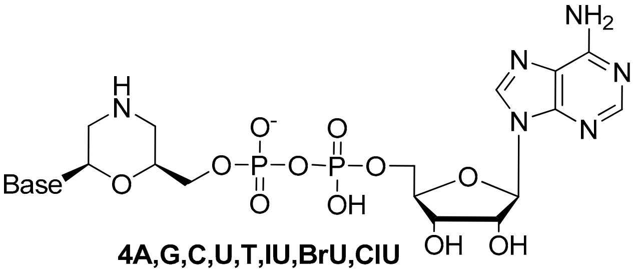

4A,G,C,U,T,IU,BrU,ClU

Adenosine-5’-*O*-{β-[6’-(adenine-9-yl)-morpholine-2’-*O*-methyl]pyrophosphate} (4A)

[1]H (D_2O): 8.19 (s, 1H, *H2*-Ade), 8.11 (s, 1H, *H2*-Ade), 8.07 (s, 1H, *H8*-Ade), 7.95 (s, 1H, *H8*-Ade), 6.02 (dd, *J* 7.6, 5.6, 1H, *H6’*), 5.82 (d, *J* 5.2, 1H, *H1*’-rA), 4.49 (br d, *J* 11.5, 1H, *H2’*), 4.43 (t, *J* 5.2, 1H, *H3’*-rA), 4.34 (t, *J* 4.4, 1H, *H2*’-rA), 4.31-4.26 (m, 2H, H5’-rA), 4.24-4.21 (m, 1H, H4’-rA), 4.21-4.15 (m. 2H, 2’-CH_2O), 3.62-3.57 (m, 2H, *H3’*, *H5’*), 3.54 (br d, 1H, *J* 12.3, *H3’*), 3.32 (t, 1H, *J* 12.3, H5’).

[31]P (D_2O): -11.14 – (-11.89) (m).

MS ESI (*m/z*): $[M-H]^-$ calcd for $C_{20}H_{26}N_{11}O_{11}P_2^-$ 658.129; found 657.892.

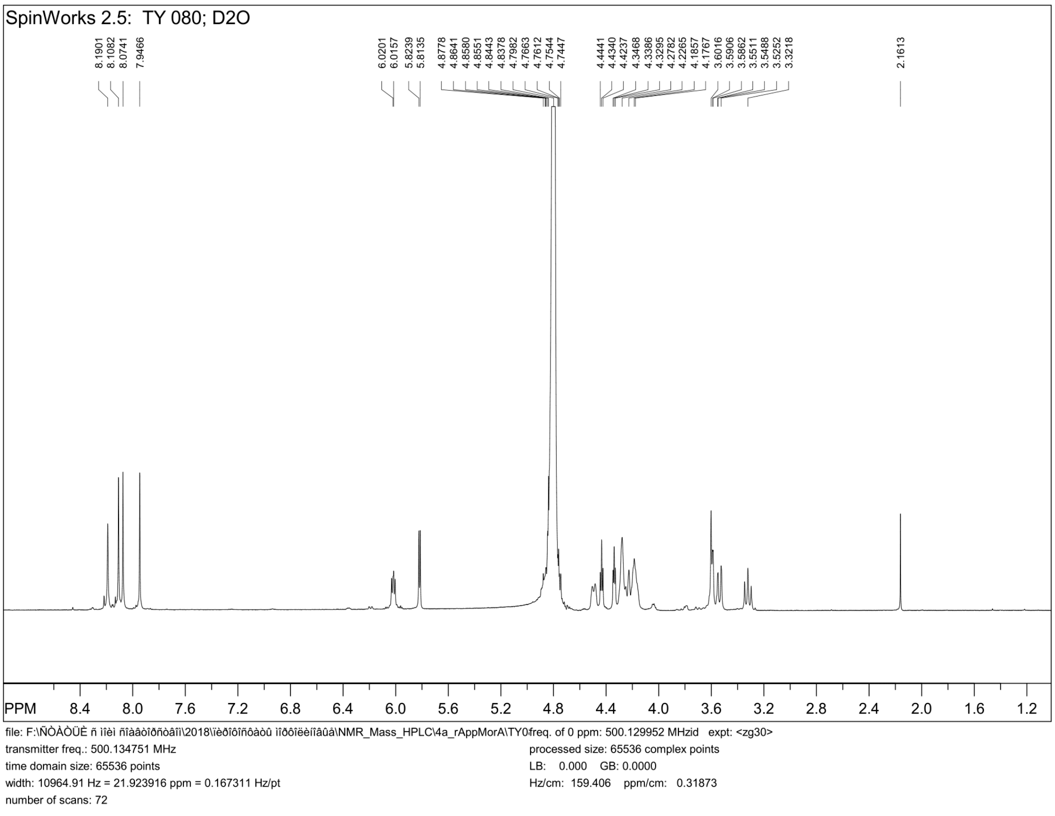

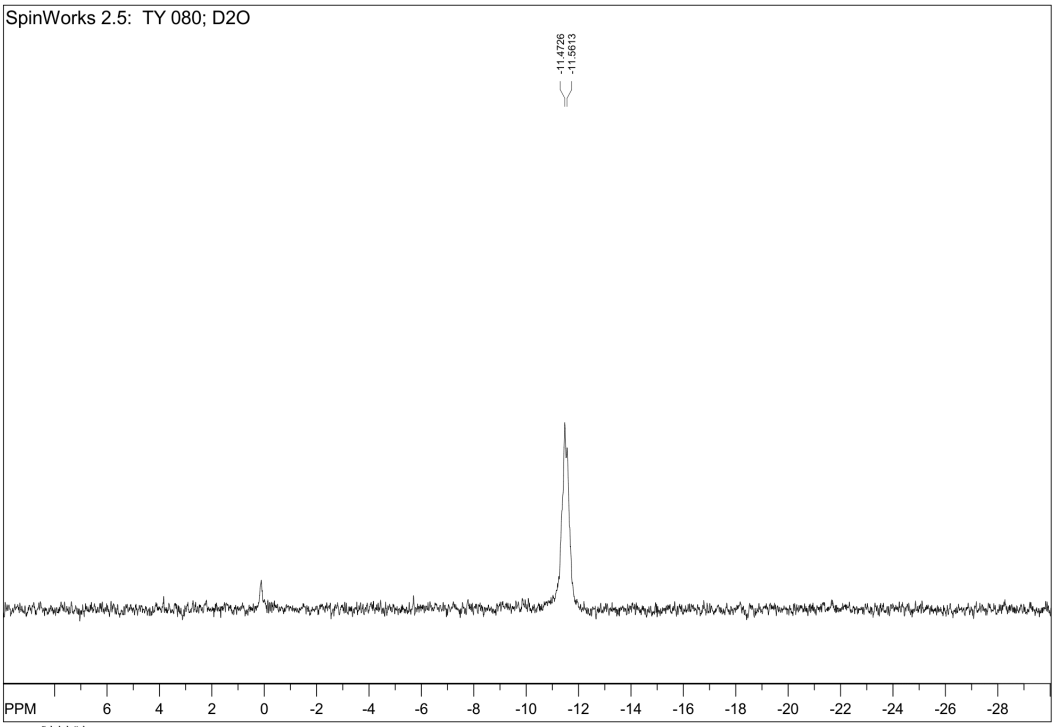

file: F:\ÑÒÀÒÜÈ ñ ïîèì ñîàâòîðñòâîì\2018\ïèðîôîñôàòû ïîðîîëèíâûà\NMR_Mass_HPLC\4a_rAppMorA\TY0freq. of 0 ppm: 202.456335 MHz\fid expt: <zg30>
transmitter freq.: 202.458794 MHz
time domain size: 65536 points
width: 29761.90 Hz = 147.002282 ppm = 0.454131 Hz/pt
number of scans: 120
processed size: 32768 complex points
LB: 0.000 GB: 0.0000
Hz/cm: 323.884 ppm/cm: 1.59975

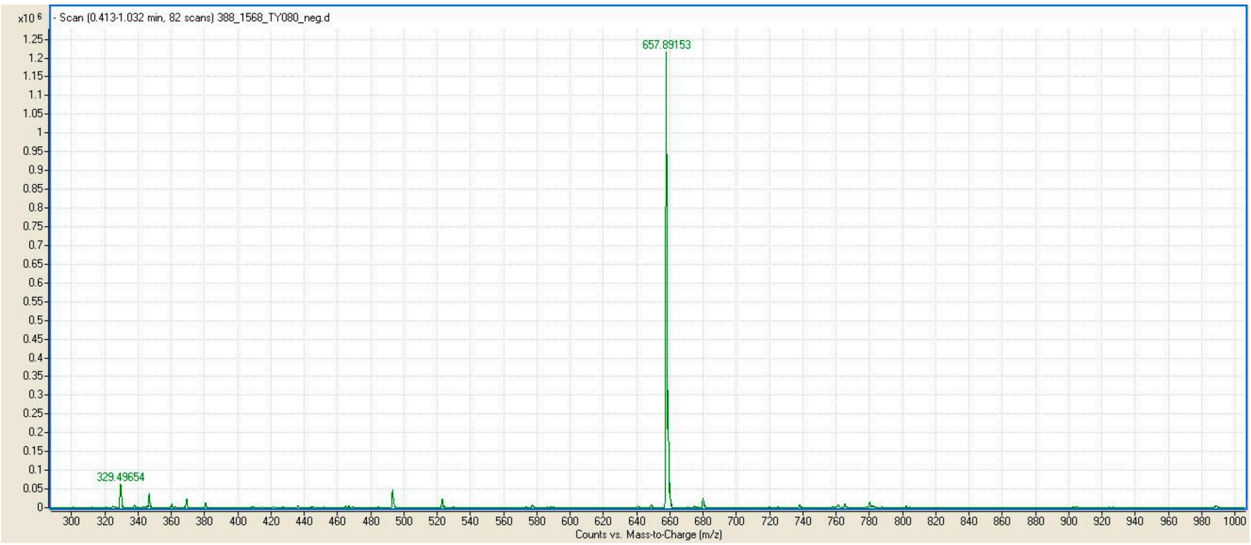

Adenosine-5’-*O*-{β-[6’-(guanine-9-yl)-morpholine-2’-*O*-methyl]pyrophosphate} (4G)

[1]H (D_2O): 8.22 (s, 1H, *H2*-Ade), 8.09 (s, 1H, *H8*-Ade), 7.80 (1H, *H8*-Gua), 5.85 (d, *J* 4.8, 1H, *H1’*-rA), 5.78 (dd, *J* 10.1, 3.0, 1H, H6’), 4.47-4.39 (2H, м, *H2’*, *H3’*-rA), 4.34 (t, *J* 4.5, 1H, *H2*’-rA), 4.30-4.23(m, 3H, *H4’*-rA, H5’-rA), 4.21-4.14 (m, 2H, 2’-*CH₂*O), 3.55-3.45 (m, 3H, H3’, H5’), 3.28 (br t, *J* 12.2, 1H, H5’).

[31]P (D_2O): -11.35 – (-11.79) (m).

MS MALDI-TOF (*m/z*): [M] calcd for $C_{20}H_{27}N_{11}O_{12}P_2$ 675.132; found 675.439; [M-H+Na] calcd for $C_{20}H_{26}N_{11}NaO_{12}P_2$ 697.114, found 697.707.

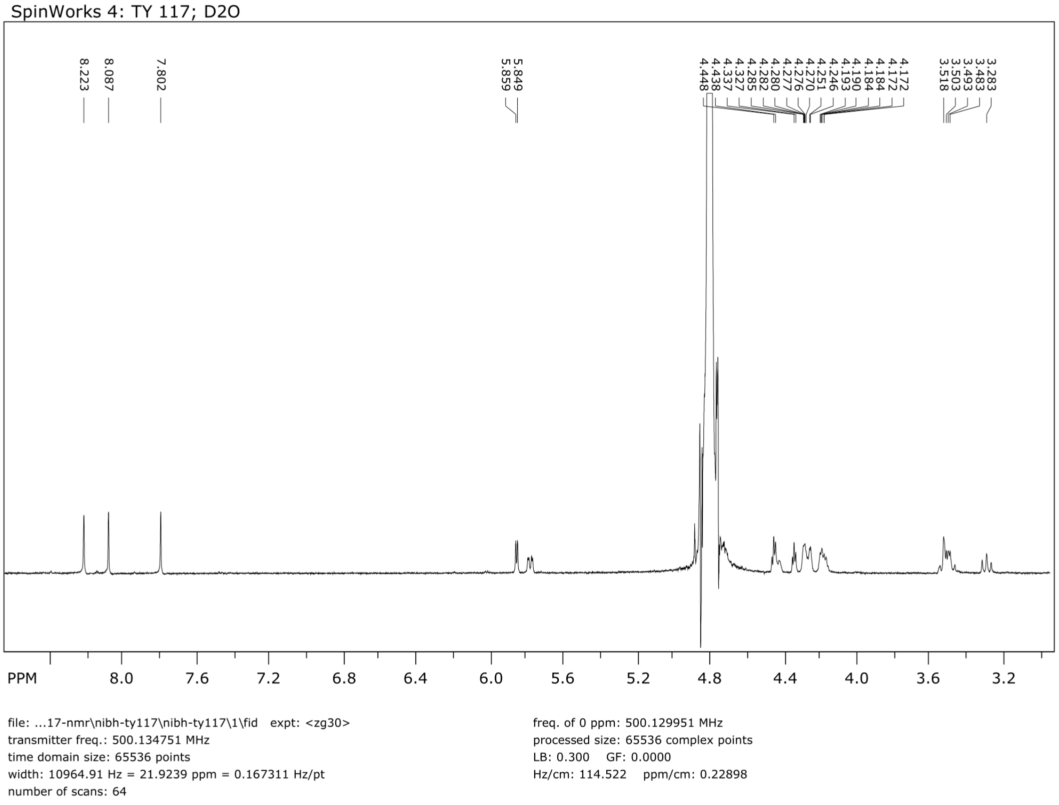

SpinWorks 4: TY 117; D2O

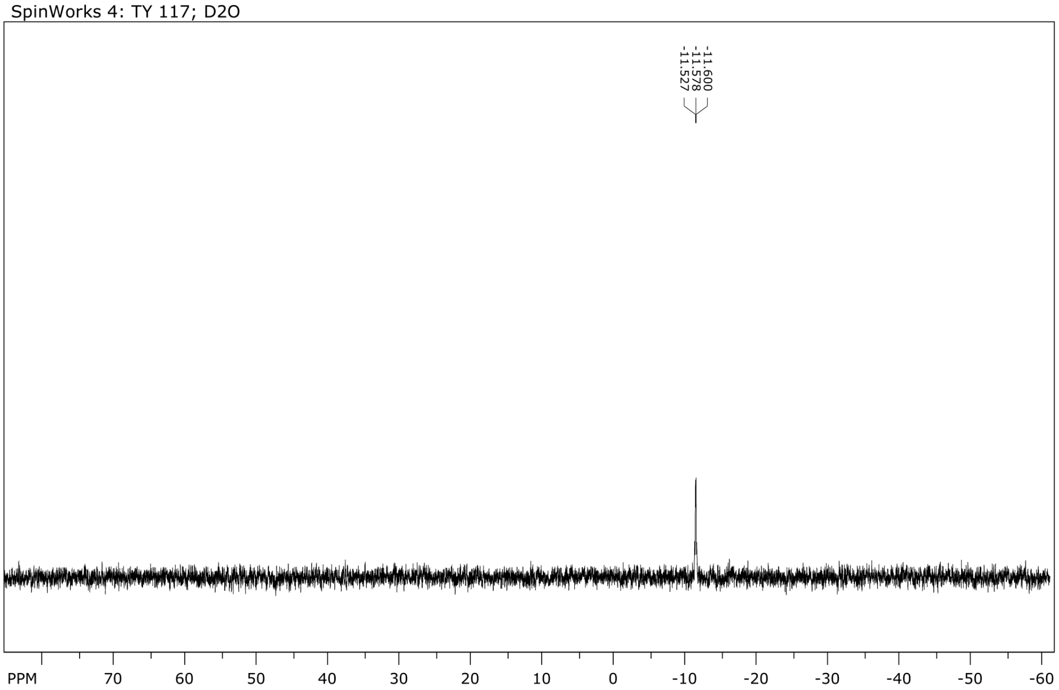

file: ...7-nmr\nibh-ty117\nibh-ty117\31\fid expt: <zg30>
transmitter freq.: 202.458794 MHz
time domain size: 65536 points
width: 29761.90 Hz = 147.0023 ppm = 0.454131 Hz/pt
number of scans: 160

freq. of 0 ppm: 202.456335 MHz
processed size: 32768 complex points
LB: 3.000 GF: 0.0000
Hz/cm: 1190.476 ppm/cm: 5.88009

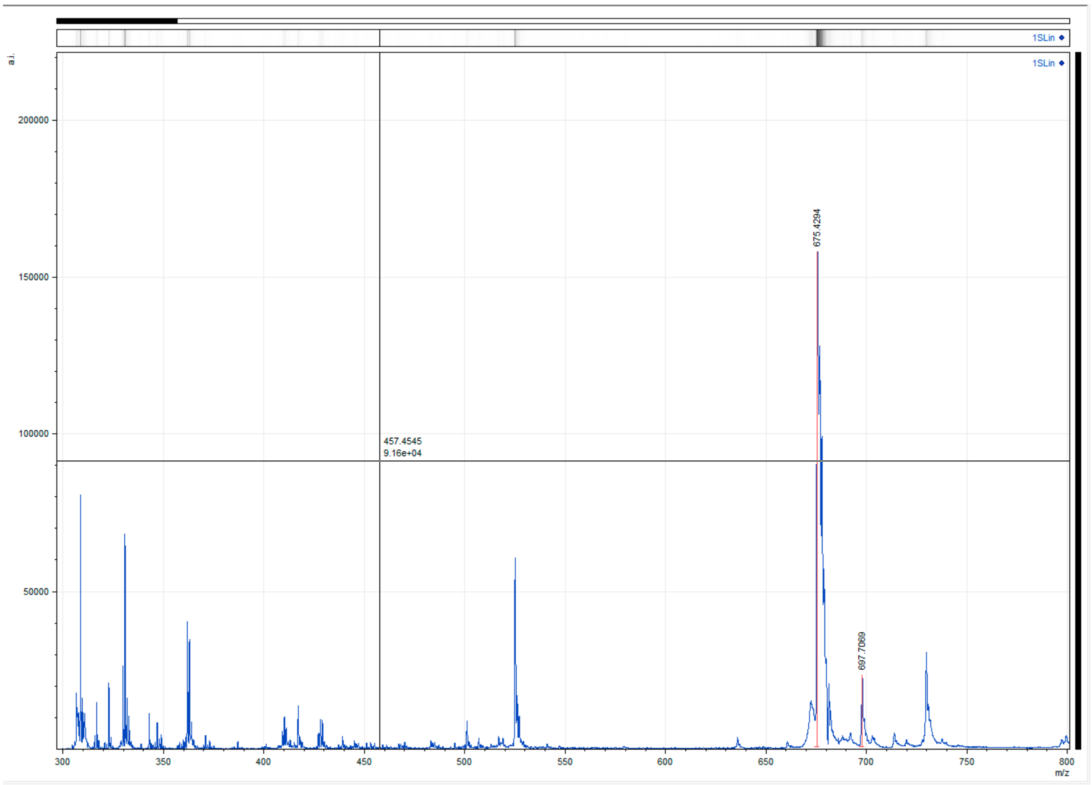

Adenosine-5'-*O*-{β-[6'-(cytosine-1-yl)-morpholine-2'-*O*-methyl]pyrophosphate} (4C)

[1]H (D_2O): 8.37 (s, 1H, *H2*-Ade), 8.09 (s, 1H, *H8*-Ade), 7.43 (d, *J* 7.5, 1H, *H6*-Cyt), 5.99 (d, *J* 5.9, 1H, *H1'*-rA), 5.73 (dd, *J* 10.4, 2.2, 1H, *H6'*), 5.68 (d, *J* 7.6, 1H, *H5*-Cyt), 4.63 (t, *J* 5.3, 1H, *H2'*-rA), 4.43 (br t, *J* 3.7, 1H, *H3'*-rA), 4.34-4.30 (m, 1H, *H4'*-rA), 4.29-4.24 (m, 1H, H2'), 4.23-4.05 (m, 4H, H5'-rA, 2'-CH_2O), 3.40-3.32 (m, 2H, H5', H3'), 3.07 (br t, *J* 11.9, 1H, H5'), 2.85 (dd, *J* 12.5, 10.8, 1H, H3').

[31]P (D_2O): -11.35 – (-11.50) (m).

MS MALDI-TOF (*m/z*): [M] calcd for $C_{19}H_{27}N_9O_{12}P_2$ 635.125; found 635.469.

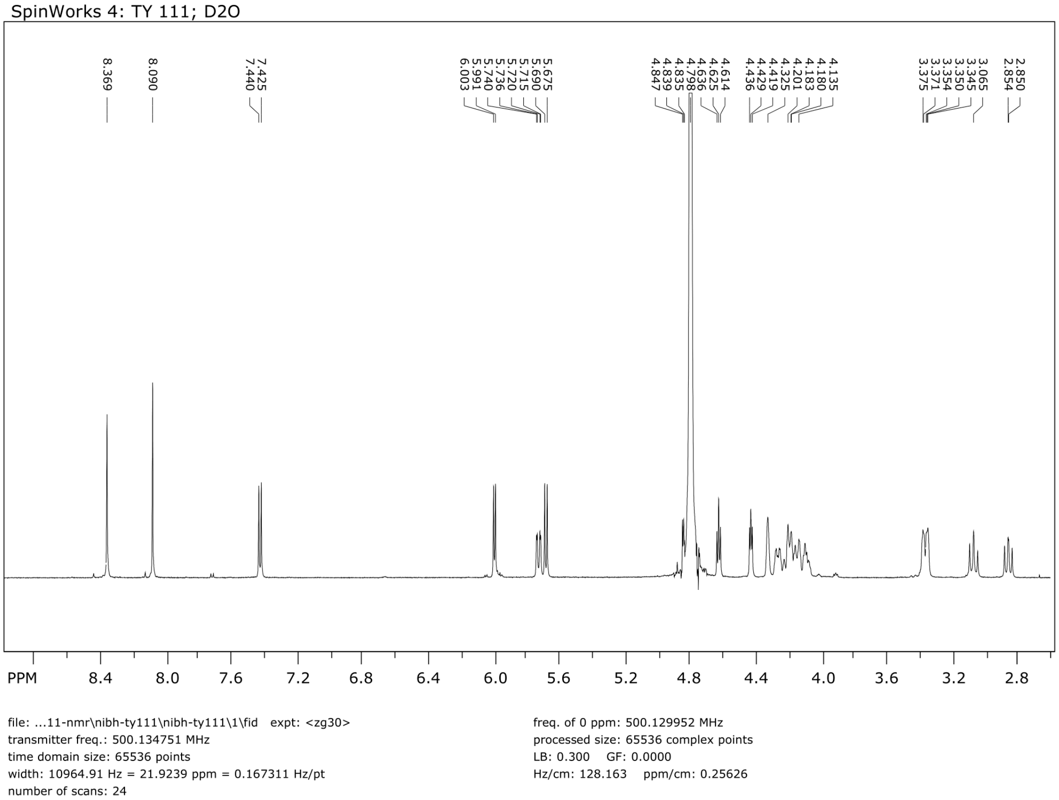

SpinWorks 4: TY 111; D2O

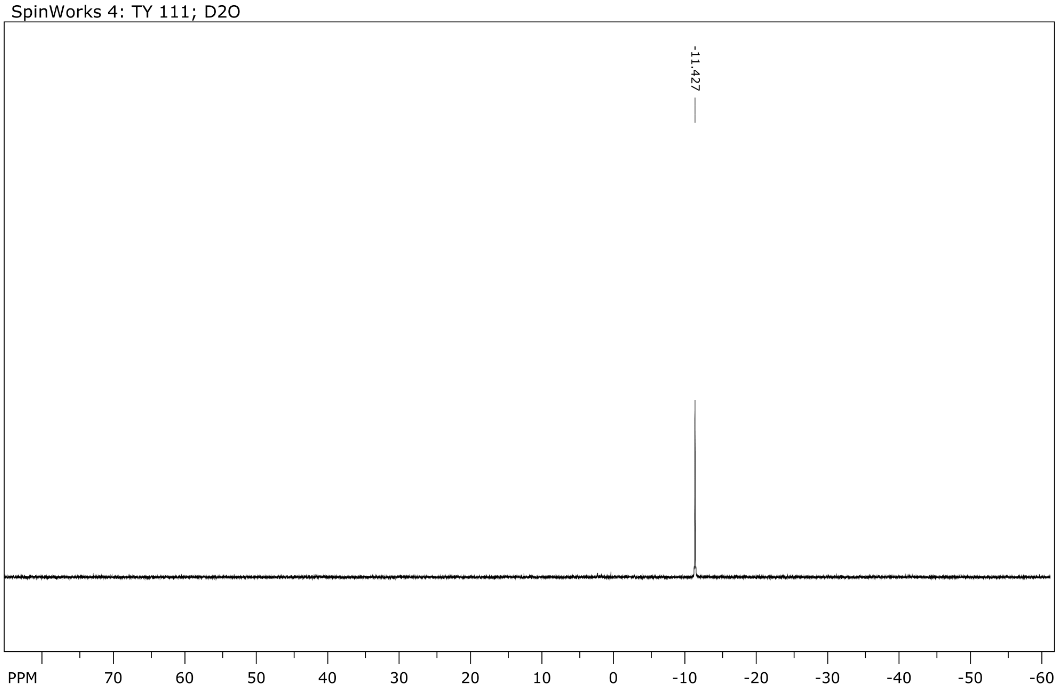

file: ...1-nmr\nibh-ty111\nibh-ty111\31\fid expt: <zg30>
transmitter freq.: 202.458794 MHz
time domain size: 65536 points
width: 29761.90 Hz = 147.0023 ppm = 0.454131 Hz/pt
number of scans: 88

freq. of 0 ppm: 202.456335 MHz
processed size: 32768 complex points
LB: 1.000 GF: 0.0000
Hz/cm: 1190.476 ppm/cm: 5.88009

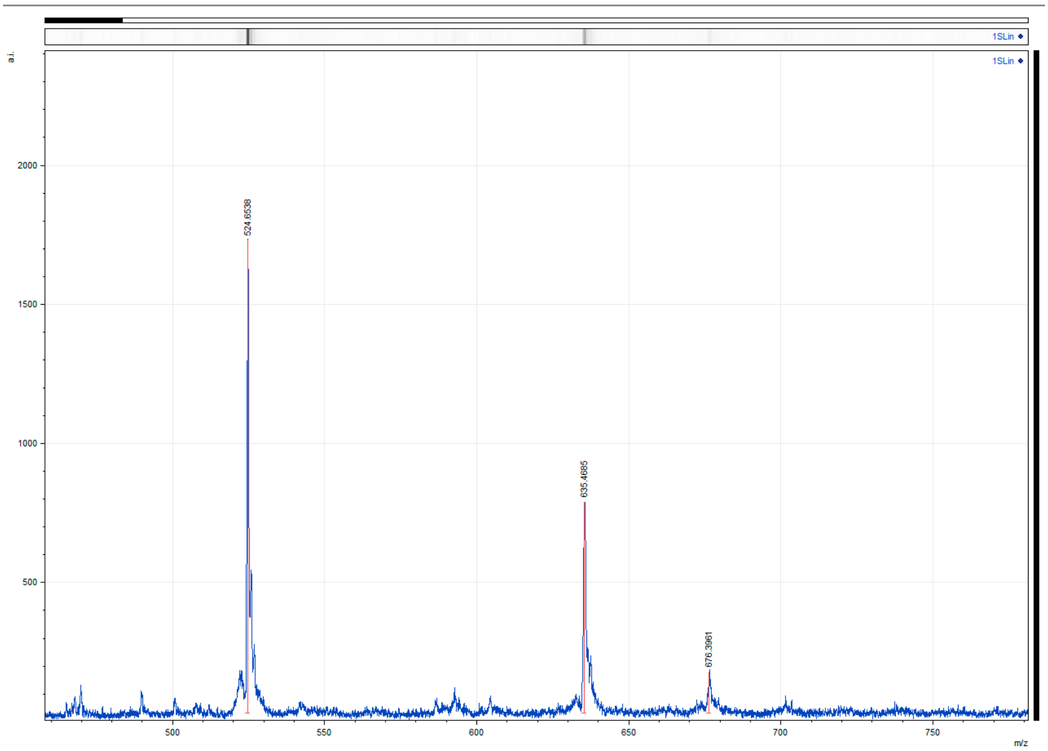

Adenosine-5’-*O*-{β-[6’-(uracil-1-yl)-morpholine-2’-*O*-methyl]pyrophosphate} (4U)

^{1}H (D_2O): 8.41 (s, 1H, *H2*-Ade), 8.13 (s, 1H, *H8*-Ade), 7.56 (d, *J* 7.6, 1H, *H6*-Ura), 5.99 (d, *J* 4.3, 1H, *H1’*-rA), 5.69-5.57 (m, 2H, *H6’*, *H5*-Ura), 4.43 (ap s, 1H, *H2’*-rA), 4.32 (ap s, 1H, *H3’*-rA), 4.26-4.15 (m, 4H, H5’-rA, 2’-CH_2O), 4.14-4.03 (m, 2H, *H4’*-rA, H2’), 3.26 (ap d, *J* 12.1, 2H, H5’, H3’), 2.96 (br t, *J* 12.1, 1H, H5’), 2.86 (br t, *J* 11.4, 1H, H3’).

^{31}P (D_2O): -11.44 (br s).

MS MALDI-TOF (*m/z*): $[M+H]^+$ calcd for $C_{19}H_{27}N_8O_{13}P_2^+$ 637.117; found 637.098.

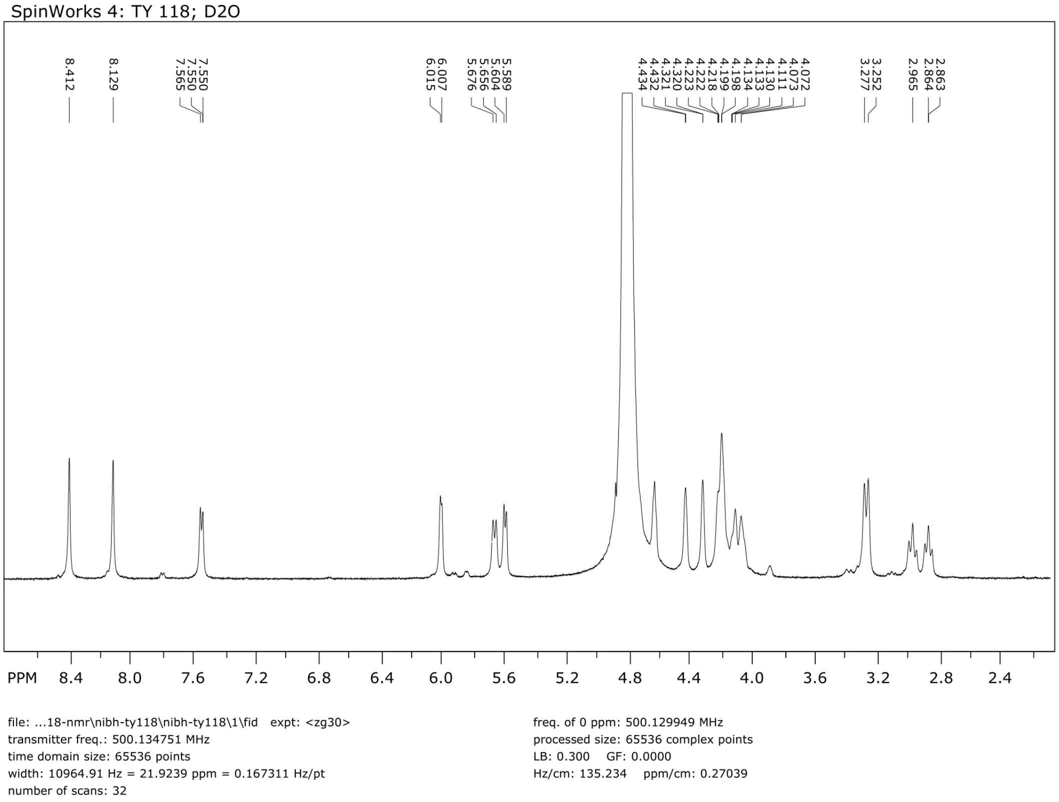

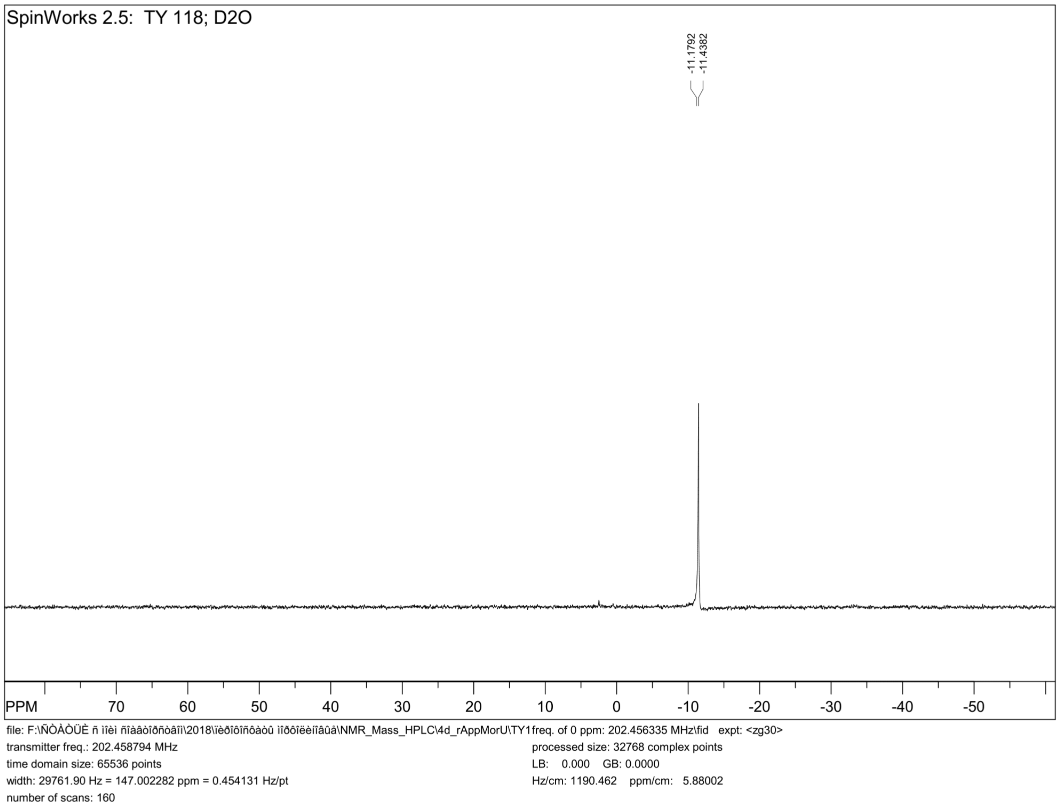
SpinWorks 2.5: TY 118; D2O
-11.1792
-11.4382
PPM 70 60 50 40 30 20 10 0 -10 -20 -30 -40 -50
file: F:\ÑÒÀÒÜÈ ñ ïîèì ñîàâòîðñòâîì\2018\ïèðîôîñôàòû ïîðôèðèíîâûõ\NMR_Mass_HPLC\4d_rAppMorU\TY1freq. of 0 ppm: 202.456335 MHz\fid expt: <zg30>
transmitter freq.: 202.458794 MHz
time domain size: 65536 points
width: 29761.90 Hz = 147.002282 ppm = 0.454131 Hz/pt
number of scans: 160
processed size: 32768 complex points
LB: 0.000 GB: 0.0000
Hz/cm: 1190.462 ppm/cm: 5.88002

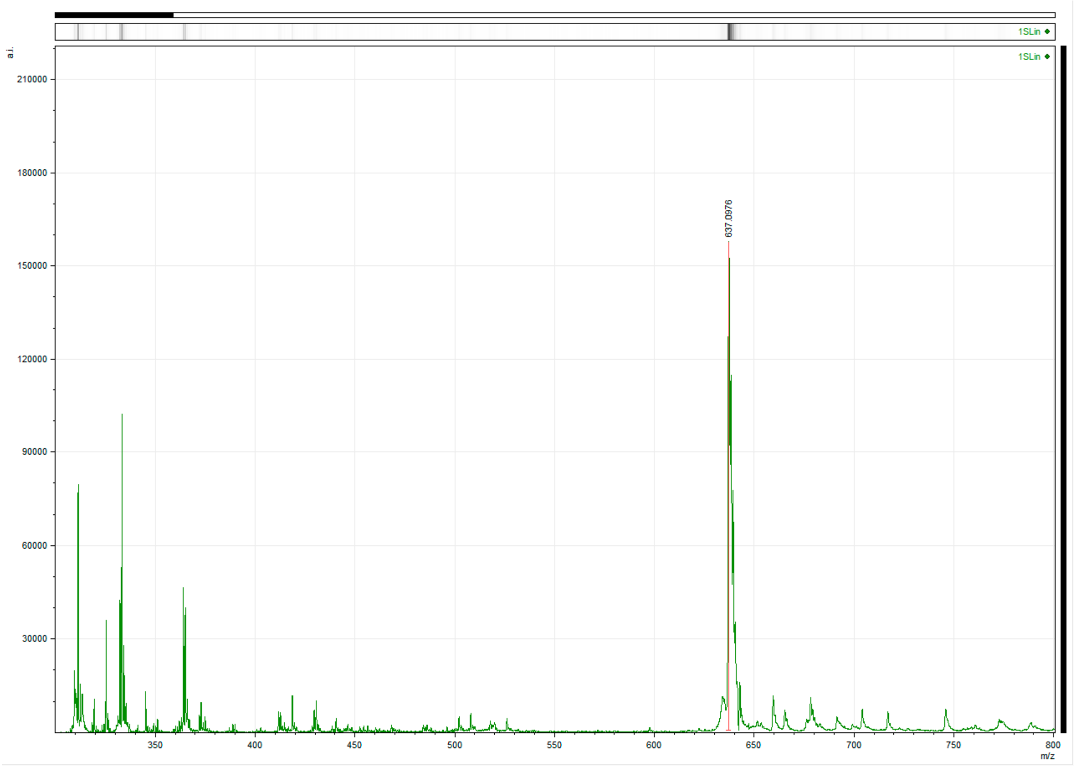
a.i.
210000
180000
150000
120000
90000
60000
30000
637.0976
1SLin
350 400 450 500 550 600 650 700 750 800
m/z

Adenosine-5'-*O*-{β-[6'-(thymine-1-yl)-morpholine-2'-*O*-methyl]pyrophosphate} (4T)

^{1}H (D_2O): 8.46 (s, 1H, *H2*-Ade), 8.19 (s, 1H, *H8*-Ade), 7.42 (s, 1H, *H6*-Thy), 6.02 (d, *J* 5.9, 1H, *H1'*-rA), 5.84 (dd, *J* 10.6, 2.2, 1H, *H6'*), 4.63 (ap t, *J* 5.3, 1H, *H2'*-rA), 4.46 (ap t, *J* 4.0, 1H, *H3'*-rA), 4.42-4.32 (m, 2H, H5'-rA), 4.3.0-4.14 (m, 4H, 2'-*CH₂*O, *H4'*-rA, H2'), 3.55-3.40 (m, 2H, *H5'*, *H3'*), 3.26-3.05 (m, 2H, H5'), 1.73 (s, 3H, *CH₃*-Thy).

^{31}P (D_2O): -11.04 (br s).

MS ESI (*m/z*): [M-H]$^-$ calcd for $C_{20}H_{26}N_8O_{13}P_2^-$ 649,118; found 648,792; [M-2H+Na]$^-$ calcd for $C_{20}H_{26}N_8NaO_{13}P_2^-$ 671.100; found 670.891.

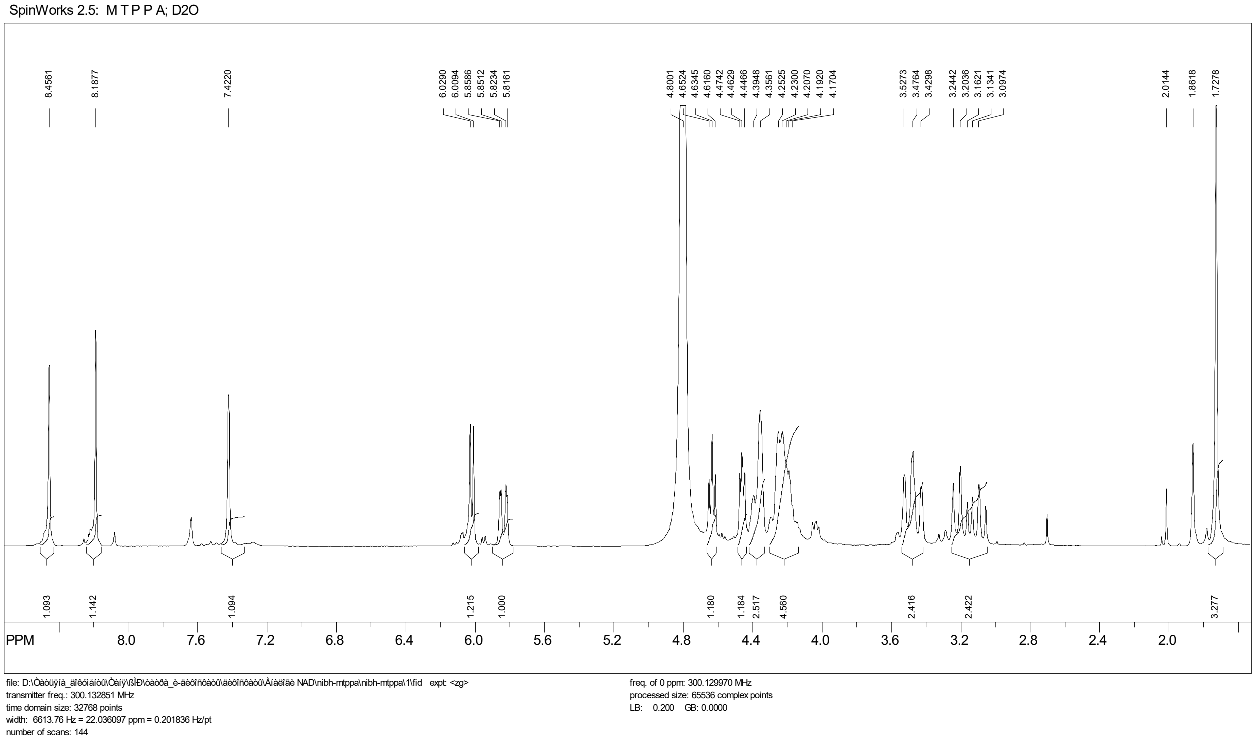

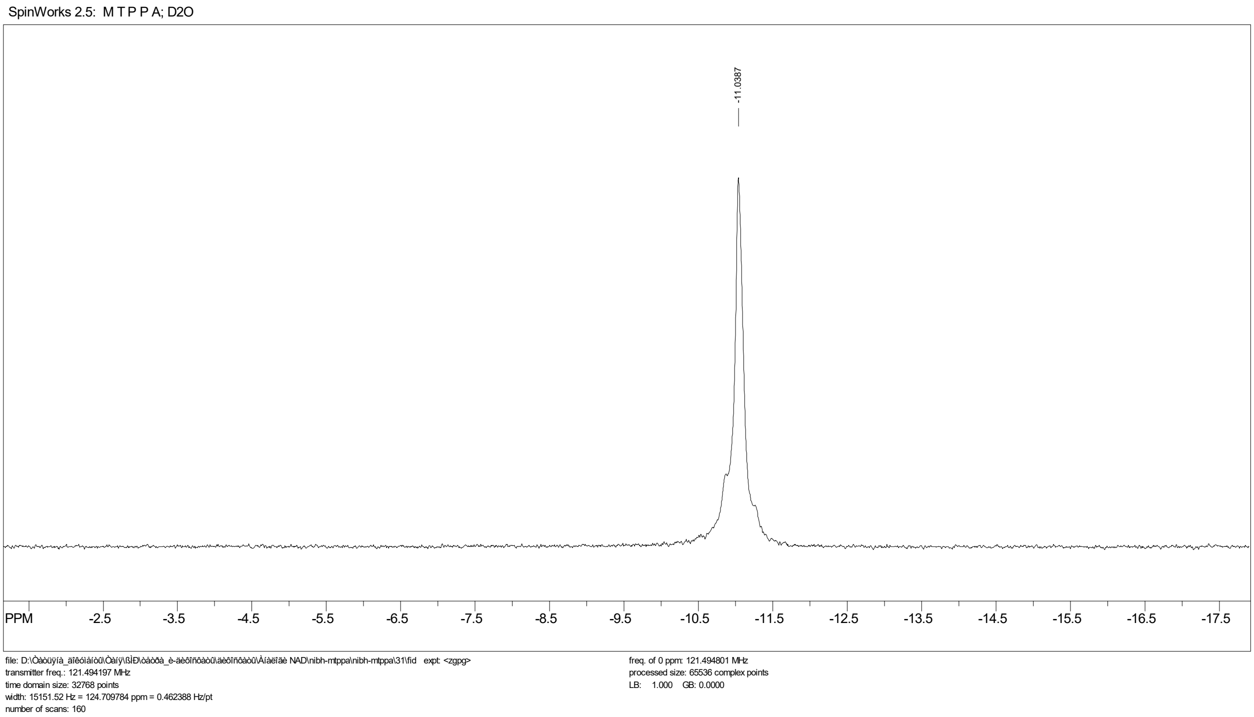
SpinWorks 2.5: M T P P A; D2O
-11.0387
PPM -2.5 -3.5 -4.5 -5.5 -6.5 -7.5 -8.5 -9.5 -10.5 -11.5 -12.5 -13.5 -14.5 -15.5 -16.5 -17.5
transmitter freq.: 121.494197 MHz
time domain size: 32768 points
width: 15151.52 Hz = 124.709784 ppm = 0.462388 Hz/pt
number of scans: 160
freq. of 0 ppm: 121.494801 MHz
processed size: 65536 complex points
LB: 1.000 GB: 0.0000

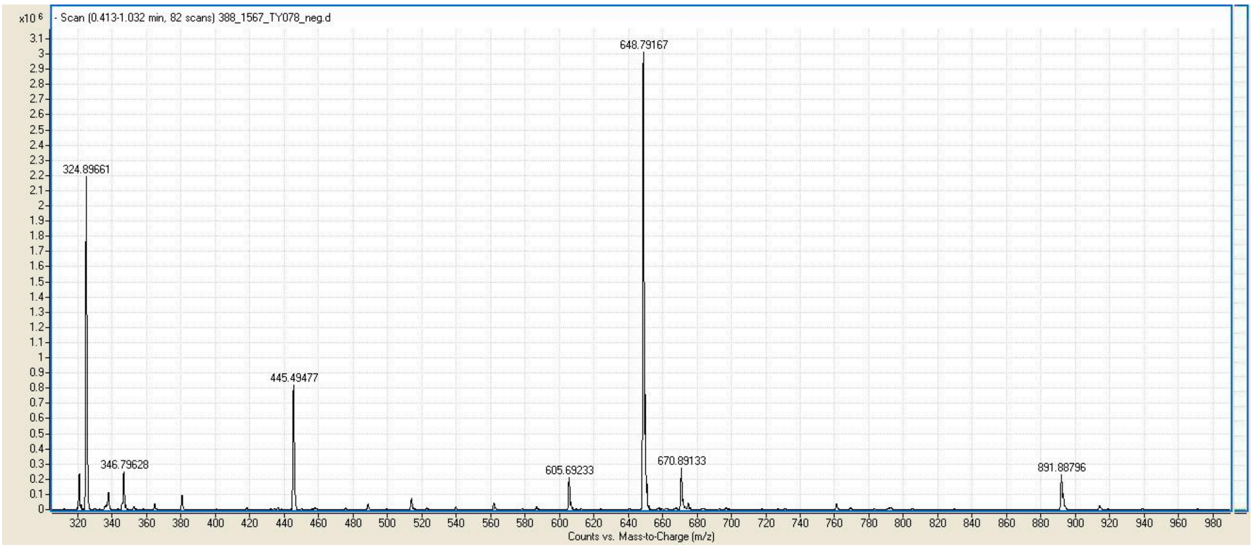
x10 6
- Scan (0.413-1.032 min, 82 scans) 388_1567_TY078_neg.d
324.89661
346.79628
445.49477
605.69233
648.79167
670.89133
891.88796
Counts vs. Mass-to-Charge (m/z)

Adenosine-5’-*O*-{β-[6’-(5-iodouracil-1-yl)-morpholine-2’-*O*-methyl]pyrophosphate} (4IU)

^{1}H (D_2O): 8.43 (s, 1H, *H2*-Ade), 8.17 (s, 1H, *H8*-Ade), 7.97 (s, 1H, *H6*-Ura), 6.03 (d, *J* 5.5, 1H, *H1’*-rA), 5.75 (dd, *J* 10.8, 2.3, 1H, *H6’*), 4.54 (t, *J* 5.3, 1H, *H2’*-rA), 4.39 (br t, *J* 4.3, 1H, *H3’*-rA), 4.35-4.29 (m, 2H, H5’-rA), 4.25-4.06 (m, 4H, 2’-*CH₂*O, *H4’*-rA, H2’), 3.44-3.35 (m, 2H, H5’, H3’), 3.13 (br t, *J* 12.3, 1H, H5’), 3.00 (dd, *J* 12.7, 11.1, 1H, H3’).

^{31}P (D_2O): -11.07 (ap s).

MS ESI (*m/z*): [M-H]$^-$ calcd for $C_{19}H_{24}IN_8O_{13}P_2^-$ 761.01; found 761.09.

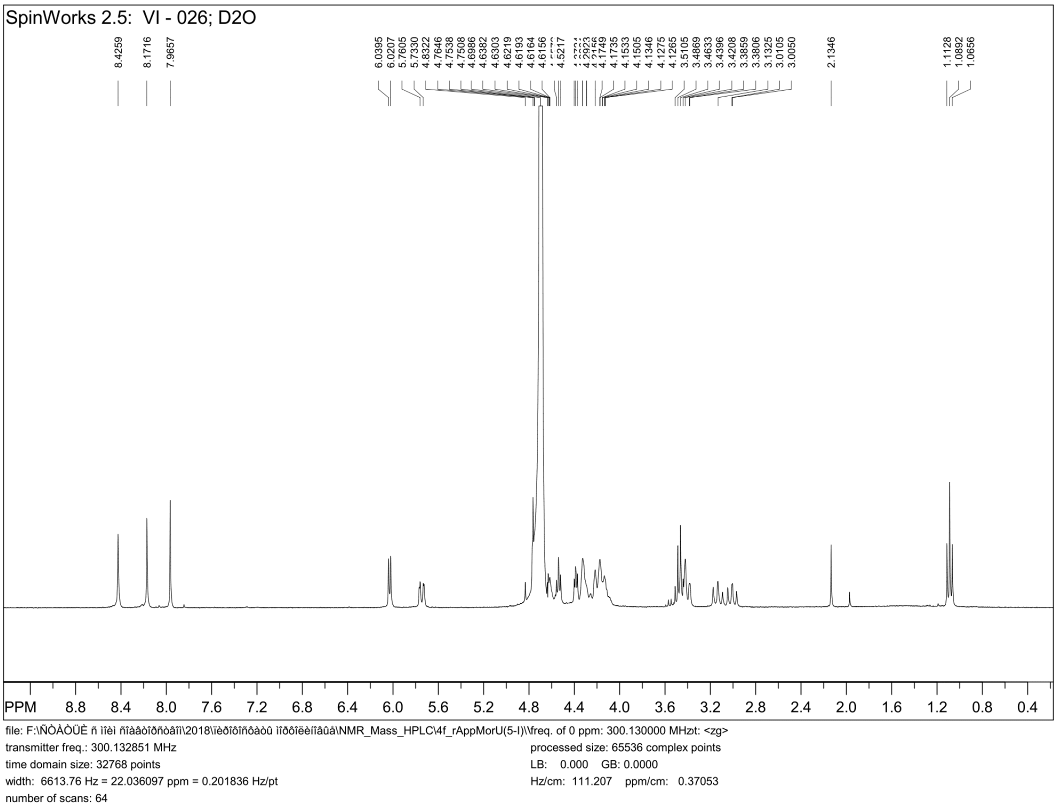

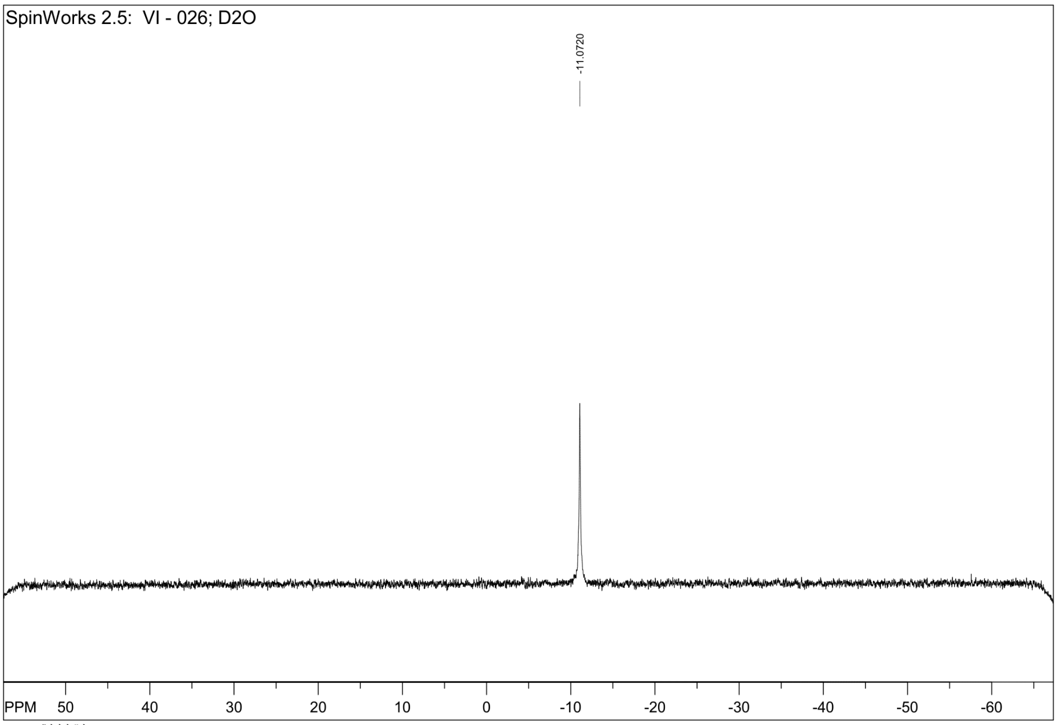

file: F:\ÑÒÀÒÜÈ ñ ìîèì ñîàâòîðñòâîì\2018\ïèðîôîñôàòû ìîðôîëèíîâûå\NMR_Mass_HPLC\4f_rAppMorU(5-I)\\freq. of 0 ppm: 121.494801 MHz:pt: <zgpg>
transmitter freq.: 121.494197 MHz
time domain size: 32768 points
width: 15151.52 Hz = 124.709784 ppm = 0.462388 Hz/pt
number of scans: 232
processed size: 65536 complex points
LB: 0.000 GB: 0.0000
Hz/cm: 606.064 ppm/cm: 4.98842

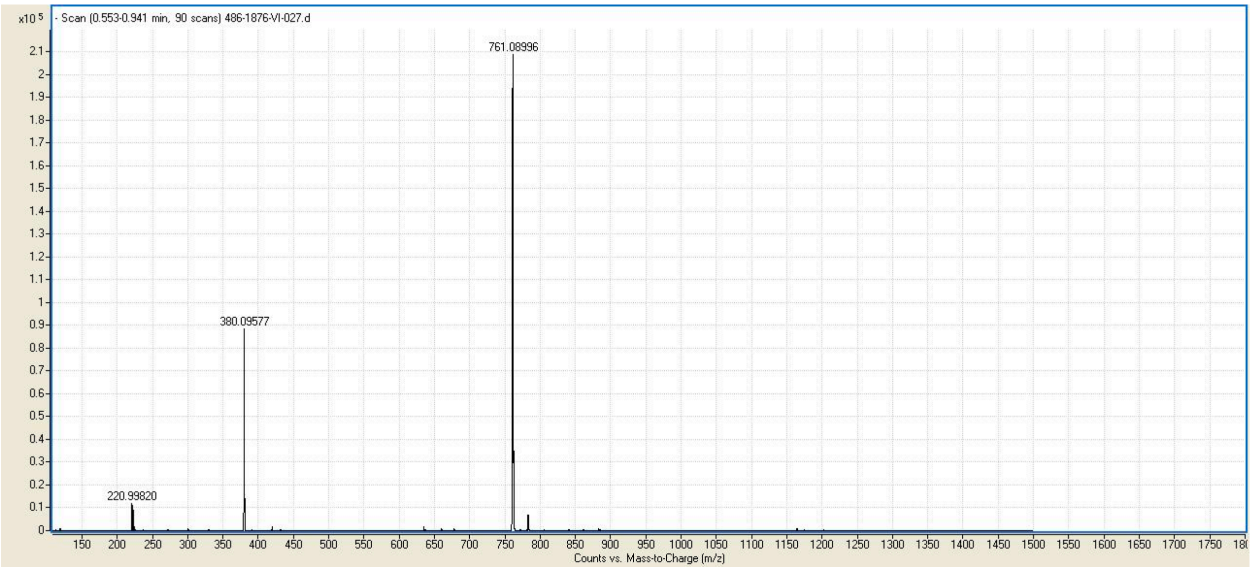

Adenosine-5’-*O*-{β-[6’-(5-bromouracil-1-yl)-morpholine-2’-*O*-methyl]pyrophosphate} (4BrU)

[1]H (D_2O): 8.50 (s, 1H, *H2*-Ade), 8.24 (s, 1H, *H8*-Ade), 8.03 (s, 1H, *H6*-Ura), 6.10 (d, *J* 5.7, 1H, *H1’*-rA), 5.84 (dd, *J* 10.5, 1.8, 1H, *H6’*), 4.64 (t, *J* 5.4, 1H, *H2’*-rA), 4.48 (br t, *J* 4.3, 1H, *H3’*-rA), 4.45-4.36 (m, 2H, H5’-rA), 4.36-4.18 (m, 4H, 2’-*CH2*O, *H4’*-rA, H2’), 3.57-3.47 (m, 2H, H5’, H3’), 3.23 (br t, *J* 12.4, 1H, H5’), 3.08 (dd, *J* 12.7, 10.5, 1H, H3’).

[31]P (D_2O): -11.09 (br s).

MS ESI (*m/z*): $[M-H]^-$ calcd for $C_{19}H_{24}BrN_8O_{13}P_2^-$ 713.02; found 712.99.

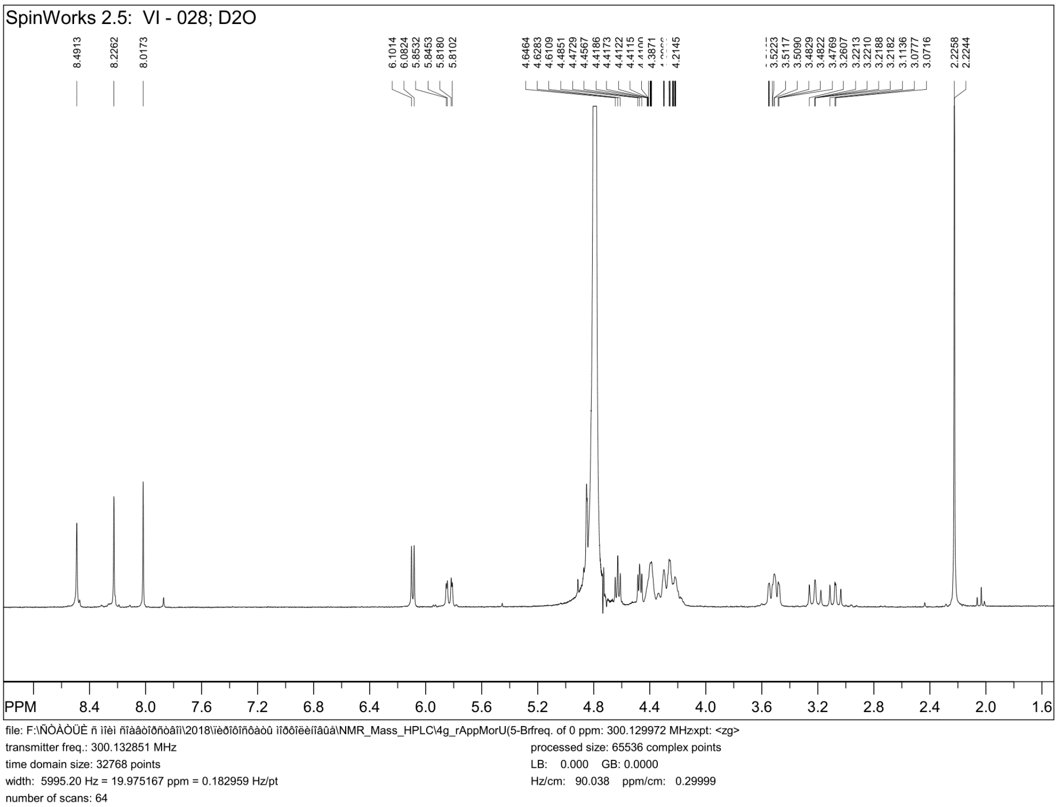

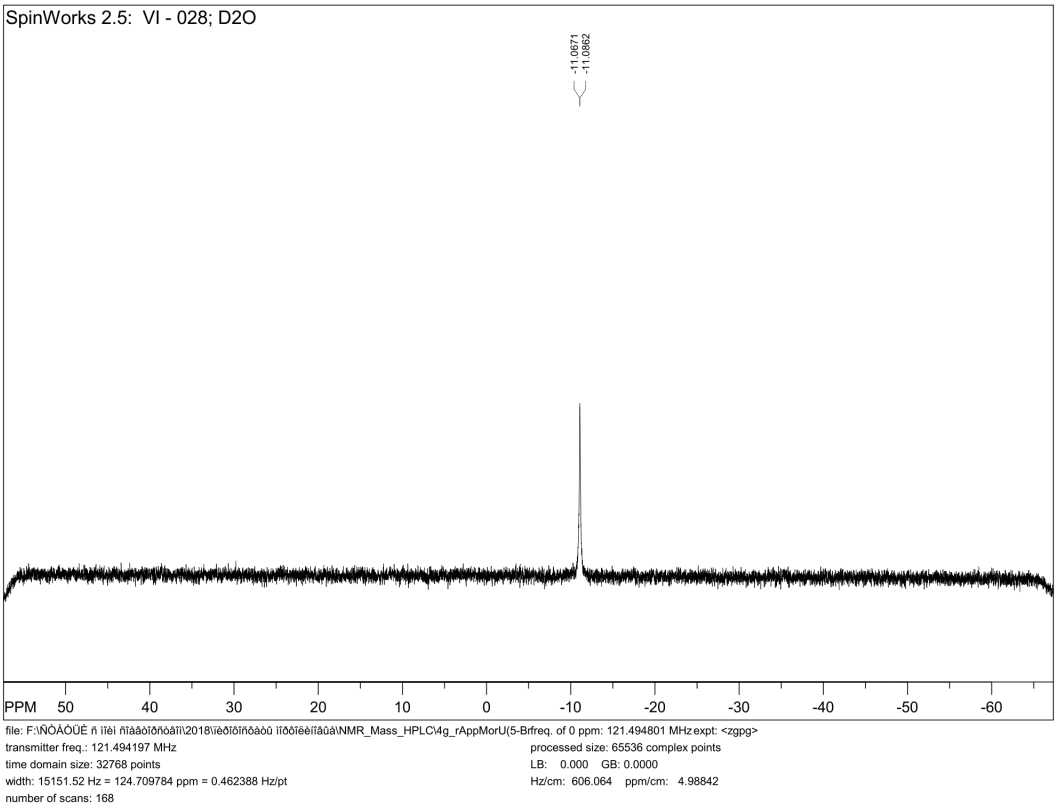
SpinWorks 2.5: VI - 028; D2O
-11.0671
-11.0862
PPM 50 40 30 20 10 0 -10 -20 -30 -40 -50 -60
file: F:\ÑÒÀÒÜÈ ñ ïîèì ñîàâòîðñòâîì\2018\ïèðîôîñôàòû ïîðôîëèíîâûå\NMR_Mass_HPLC\4g_rAppMorU(5-Br freq. of 0 ppm: 121.494801 MHz expt: <zgpg>
transmitter freq.: 121.494197 MHz
time domain size: 32768 points
width: 15151.52 Hz = 124.709784 ppm = 0.462388 Hz/pt
number of scans: 168
processed size: 65536 complex points
LB: 0.000 GB: 0.0000
Hz/cm: 606.064 ppm/cm: 4.98842

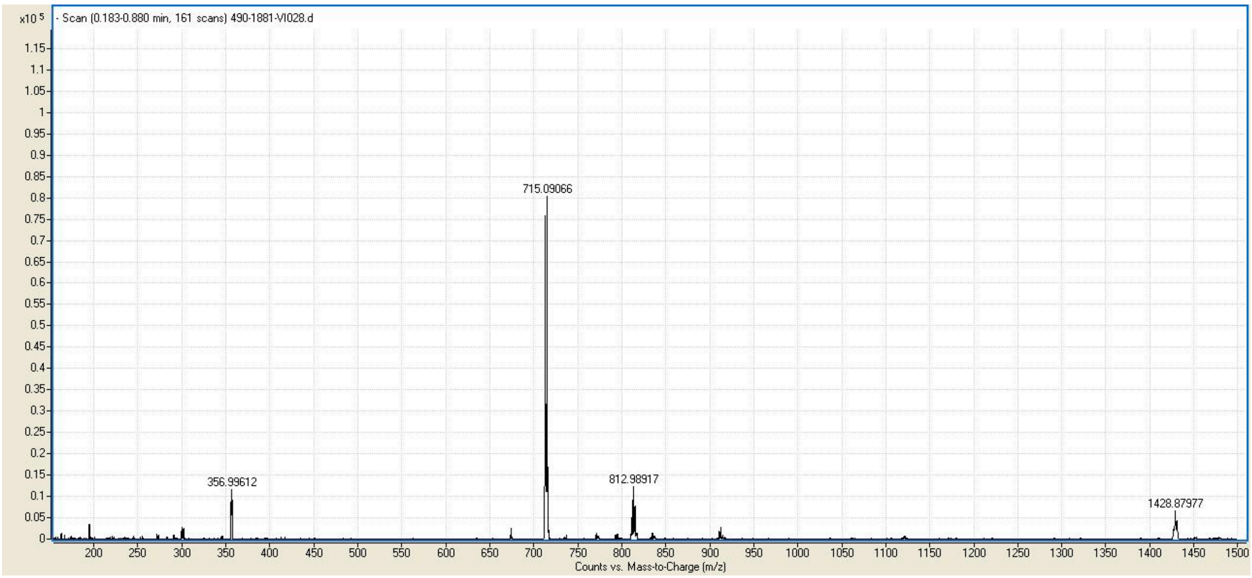
x10 5
- Scan (0.183-0.880 min, 161 scans) 490-1881-VI028.d
715.09066
356.99612
812.98917
1428.87977
Counts vs. Mass-to-Charge (m/z)

Adenosine-5’-*O*-{β-[6’-(5-chlorouracil-1-yl)-morpholine-2’-*O*-methyl]pyrophosphate} (4ClU)

[1]H (D_2O): 8.44 (s, 1H, *H2*-Ade), 8.20 (s, 1H, *H8*-Ade), 7.90 (s, 1H, *H6*-Ura), 5.98 (d, *J* 5.5, 1H, *H1’*-rA), 5.79 (dd, *J* 10.6, 2.1, 1H, *H6’*), 4.55 (t, *J* 5.3, 1H, *H2’*-rA), 4.37 (br t, *J* 4.3, 1H, *H3’*-rA), 4.34-4.24 (m, 2H, H5’-rA), 4.22-4.02 (m, 4H, 2’-CH_2O, *H4’*-rA, H2’), 3.47-3.36 (m, 2H, H5’, H3’), 3.11 (br t, *J* 12.6, 1H, H5’), 3.00 (br t, *J* 11.8, 1H, H3’).

[31]P (D_2O): -10.45 - (-11.67) (m).

MS MALDI-TOF (*m/z*): [M] calcd for $C_{19}H_{25}ClN_8O_{13}P_2$ 670.070; found 670.987; [M-H+Na] calcd for $C_{19}H_{24}ClN_8NaO_{13}P_2$ 670.070; found 692.985.

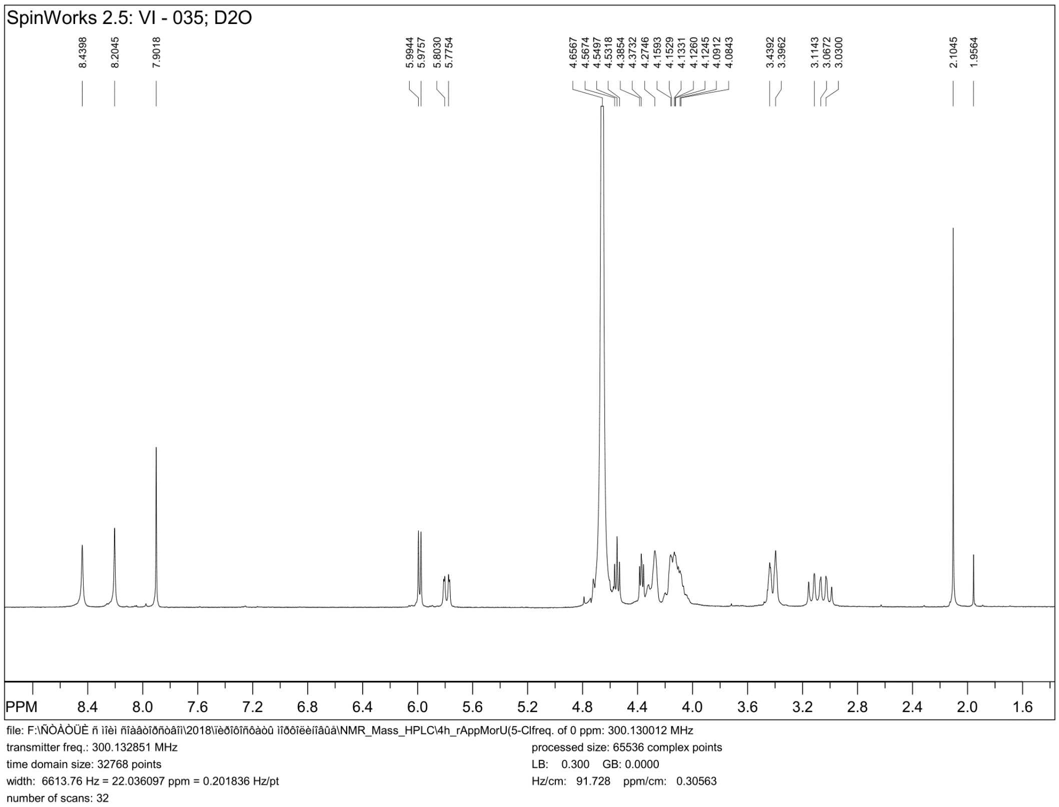

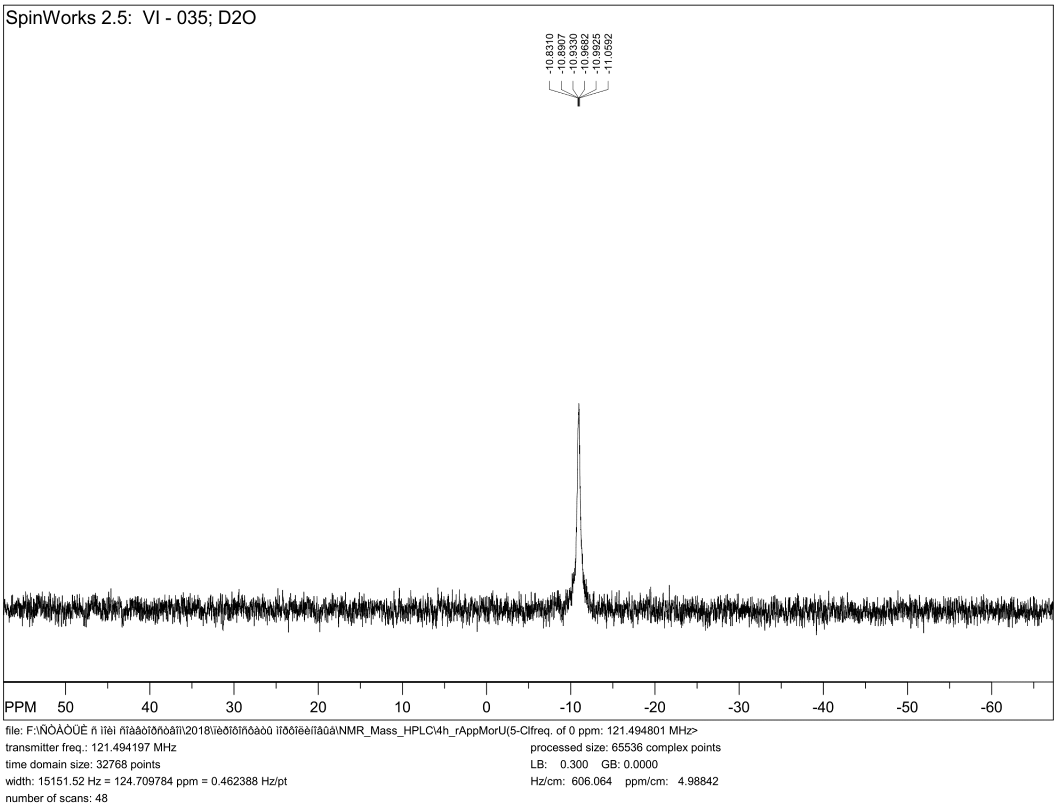
SpinWorks 2.5: VI - 035; D2O
-10.8310
-10.8907
-10.9330
-10.9682
-10.9925
-11.0592
PPM 50 40 30 20 10 0 -10 -20 -30 -40 -50 -60
file: F:\ÑÒÀÒÜÈ ñ ìîèì ñîàâòîðñòâîì\2018\ïèðîôîñôàòû ìîðôîëèíîâûå\NMR_Mass_HPLC\4h_rAppMorU(5-Clfreq. of 0 ppm: 121.494801 MHz>
transmitter freq.: 121.494197 MHz
time domain size: 32768 points
width: 15151.52 Hz = 124.709784 ppm = 0.462388 Hz/pt
number of scans: 48
processed size: 65536 complex points
LB: 0.300 GB: 0.0000
Hz/cm: 606.064 ppm/cm: 4.98842

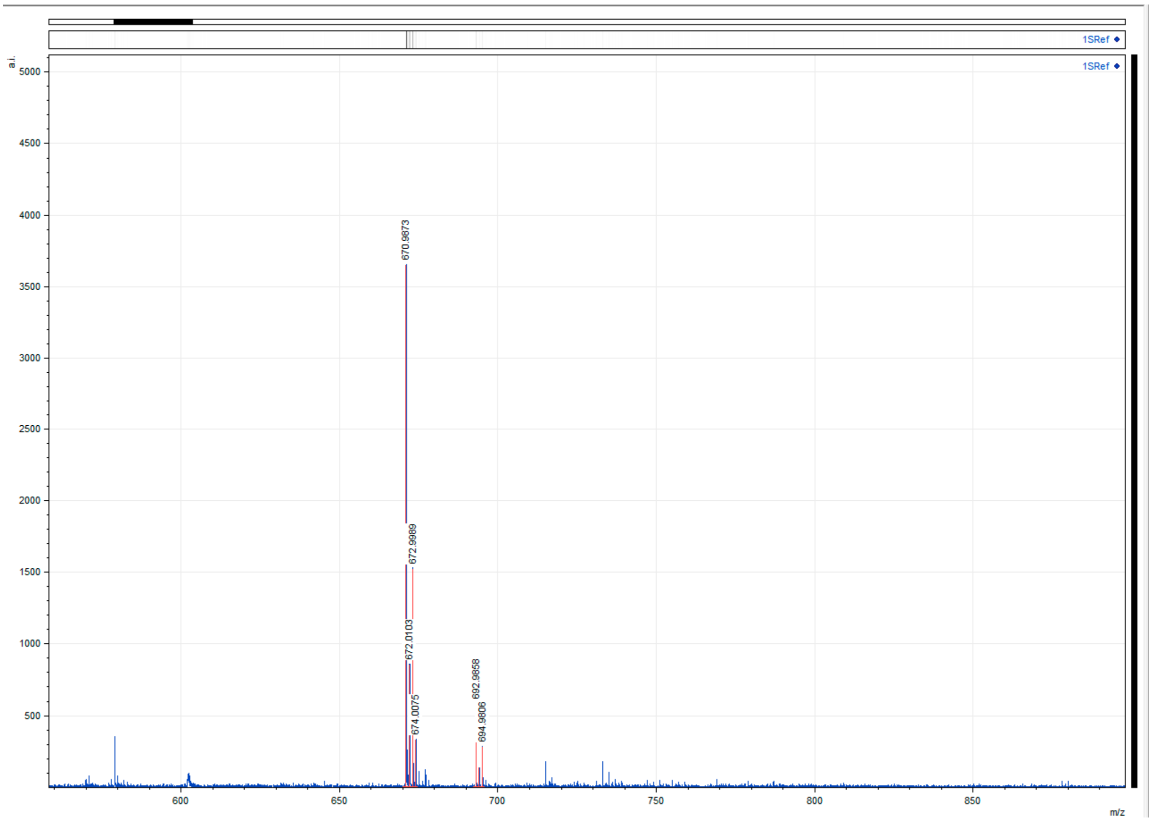
1SRef
a.i.
670.9873
672.9989
672.0103
674.0075
692.9858
694.9806
m/z

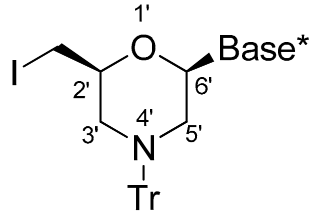

5A,G,C,U,T,IU,BrU,ClU

2'-Iodomethyl-4'-*N*-trityl-6'-(*N*⁶-benzoyladenine-9-yl)-morpholine (5A)

^{1}H (500 MHz, DMSO-d$_6$): 11.17 (s, 1H, *NH*), 8.75 (s, 1H, *H8*-Ade), 8.40 (s, 1H, *H2*-Ade), 8.03 (dt, *J* 7.2, 1.3, 2H, *o*-Bz), 7.62 (tt, *J* 7.4, 1.3, 1H, *p*-Bz), 7.53 (tt, *J* 7.4, 1.5, 2H, *m*-Bz), 7.51-7.44 (m, 6H, Tr), 7.34 (br t, *J* 7.7, 6H, Tr), 7.21 (br t, *J* 7.2, 3H, Tr), 6.48 (dd, *J* 9.9, 2.1, 1H, *H6'*), 4.37-4.31 (m, 1H, *H2'*), 3.38-3.28 (m, 2H, *CH$_2$*-I), 3.29 (t, *J* 5.3, 1H, *H5'*), 3.22 (dd, *J* 10.6, 5.6, 1H, *H3'*), 2.10 (dd, *J* 11.8, 10.2, 1H, *H3'*), 1.47 (dd, *J* 11.8, 10.2, 1H, *H5'*).

^{13}C (125 MHz, DMSO-d$_6$): 165.47, 151.76, 151.52, 150.37, 142.02, 133.27, 132.29, 128.83, 128.35, 128.31, 127.80, 126.32, 125.13, 79.79, 76.32, 74.89, 51.80, 51.38, 5.75.

MS MALDI-TOF (*m/z*): [M] calcd for $C_{36}H_{31}IN_6O_2$ 706.155; found 706.800.

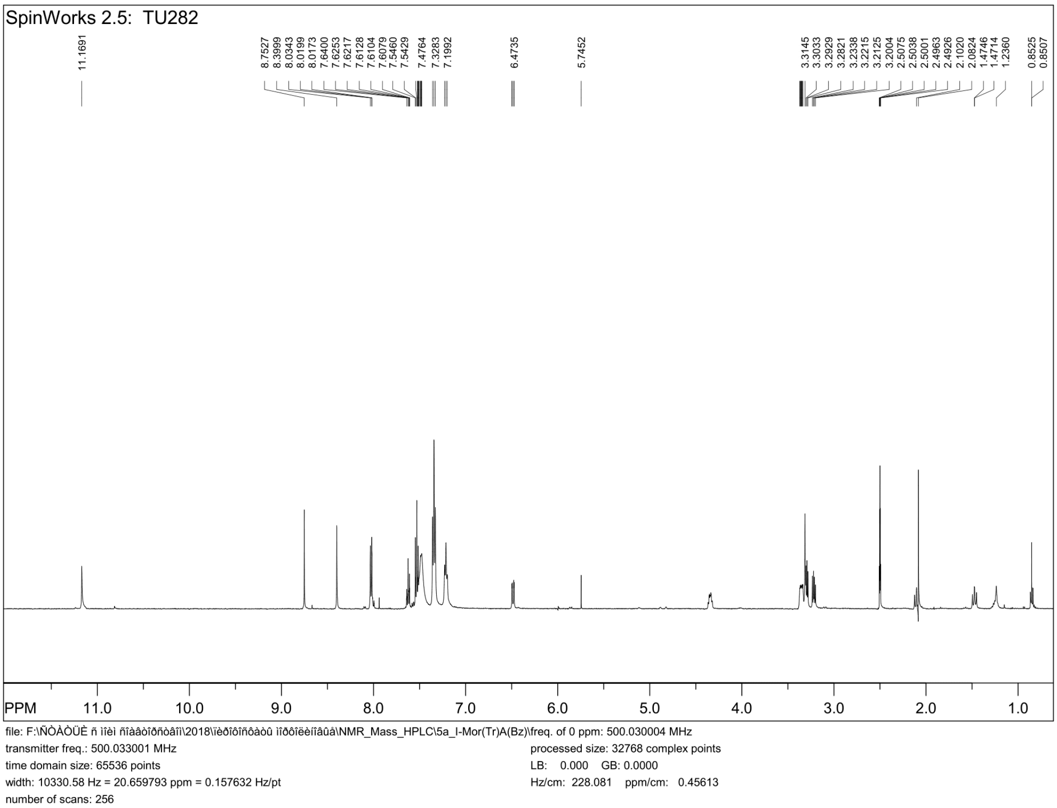

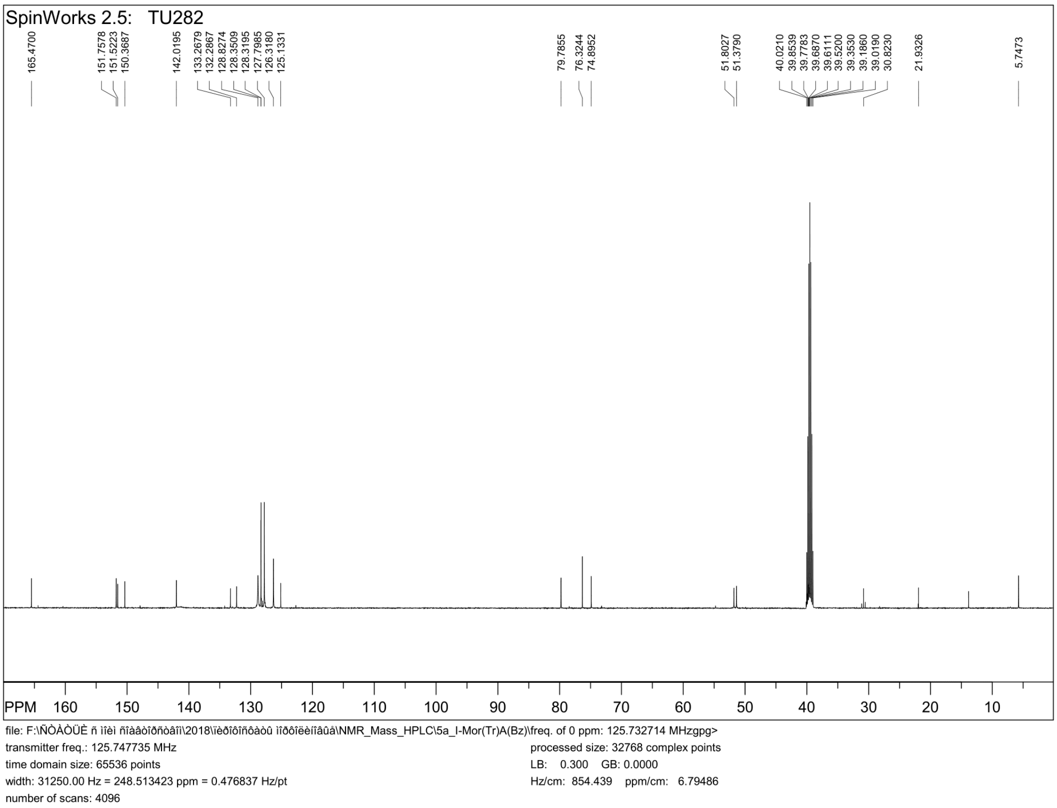
SpinWorks 2.5: TU282
165.4700
151.7578
151.5223
150.3687
142.0195
133.2679
132.2867
128.8274
128.3509
128.3195
127.7985
126.3180
125.1331
79.7855
76.3244
74.8952
51.8027
51.3790
40.0210
39.8539
39.7783
39.6870
39.6111
39.5200
39.3530
39.1860
39.0190
30.8230
21.9326
5.7473
PPM 160 150 140 130 120 110 100 90 80 70 60 50 40 30 20 10
file: F:\ÑÒÀÒÜÈ ñ ìîèì ñîàâòîðñòâîì\2018\ïèðîíôóðàíû ïîðôîëèíîâû\NMR_Mass_HPLC\5a_l-Mor(Tr)A(Bz)\freq. of 0 ppm: 125.732714 MHzgpg>
transmitter freq.: 125.747735 MHz
time domain size: 65536 points
width: 31250.00 Hz = 248.513423 ppm = 0.476837 Hz/pt
number of scans: 4096
processed size: 32768 complex points
LB: 0.300 GB: 0.0000
Hz/cm: 854.439 ppm/cm: 6.79486

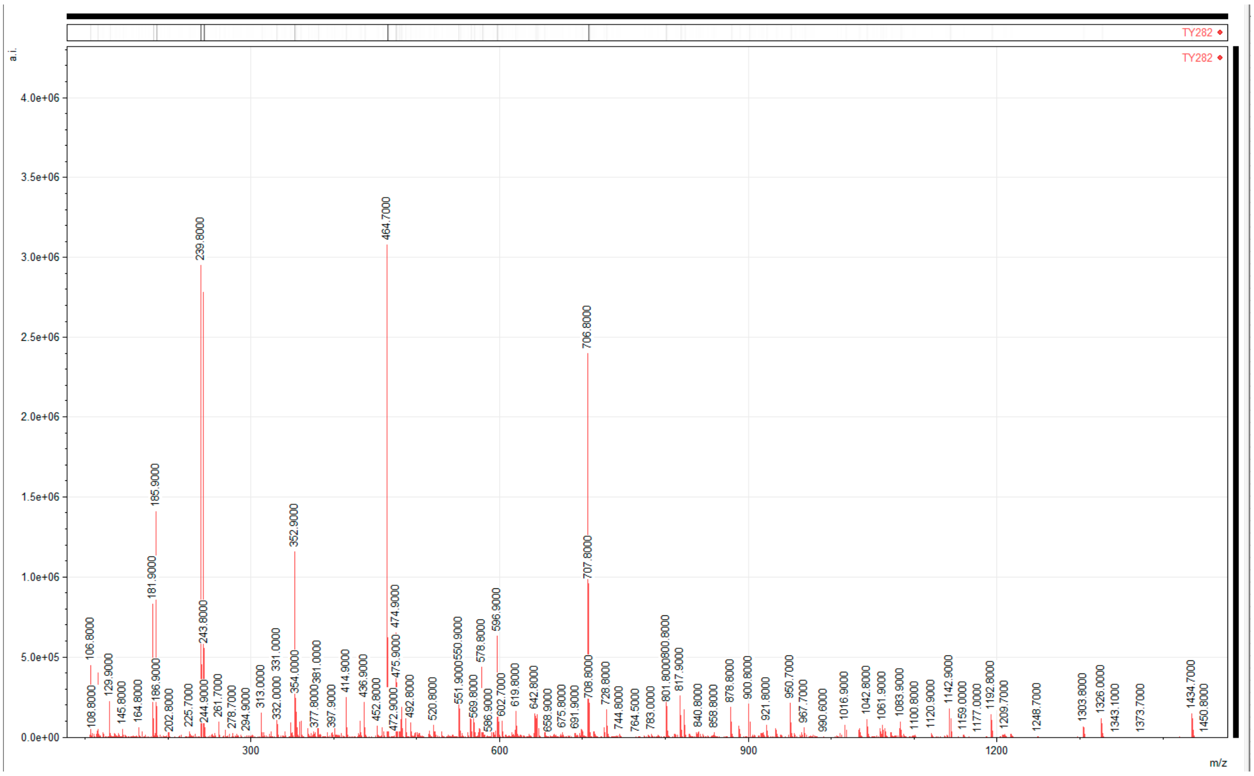
TY282
a.i.
4.0e+06
3.5e+06
3.0e+06
2.5e+06
2.0e+06
1.5e+06
1.0e+06
5.0e+05
0.0e+00
464.7000
239.8000
706.8000
185.9000
352.9000
707.8000
181.9000
474.9000
243.8000
596.9000
106.8000
550.9000
578.8000
300
600
900
1200
m/z

2’-Iodomethyl-4’-*N*-trityl-6’-(N^2-isobutyrylguanine-9-yl)-morpholine (5G)

^{1}H (500 MHz, DMSO-d$_6$): 12.11 (s, 1H, *NH*), 11.69 (s, 1H, *NH*-Gua), 7.85 (s, 1H, *H8*-Gua), 7.46-7.39 (m, 6H, Tr), 7.35 (br t, *J* 7.6, 6H, Tr), 7.21 (br t, *J* 6.9, 3H, Tr), 6.09 (dd, *J* 10.0, 2.2, 1H, *H6’*), 4.22-4.14 (m, 1H, *H2’*), 3.30-3.17 (m, 4H, *CH$_2$*-I, *H5’*, *H3’*), 2.85 (p, *J* 6.8, 1H, *CH*-iBu), 1.93 (dd, *J* 11.1, 10.2, 1H, *H3’*), 1.41 (dd, *J* 12.1, 10.1, 1H, *H5’*), 1.16 (br d, *J* 1.4, 3H, *CH$_3$*-iBu), 1.15 (br d, *J* 1.4, 3H, *CH$_3$*-iBu).

^{13}C (125 MHz, DMSO-d$_6$): 180.08, 154.67, 148.35, 147.90, 136.59, 128.25, 127.88, 126.37, 119.71, 79.08, 76.28, 74.99, 51.81, 51.53, 34.67, 18.86, 18.72, 5.67.

MS MALDI-TOF (*m/z*): [M-H]$^-$ calcd for $C_{33}H_{32}IN_6O_3^-$ 687.159; found 687.112; [M+Na]$^+$ calcd for $C_{33}H_{33}IN_6NaO_3^+$ 711.156; found 711.105.

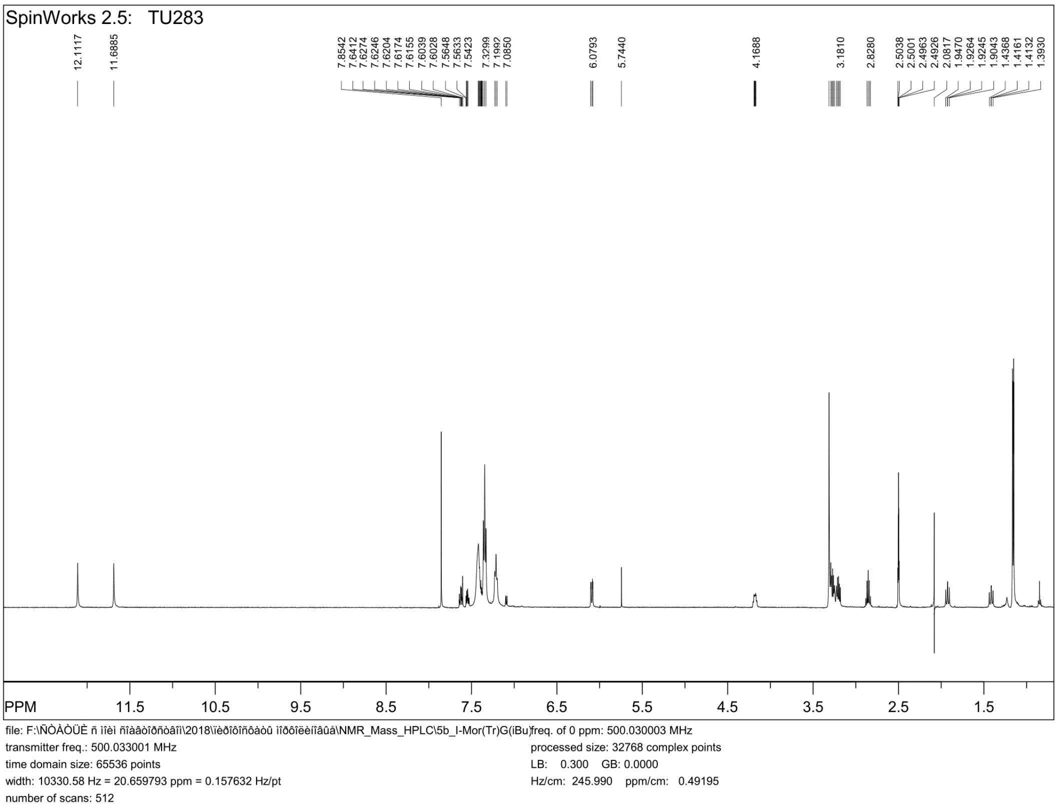

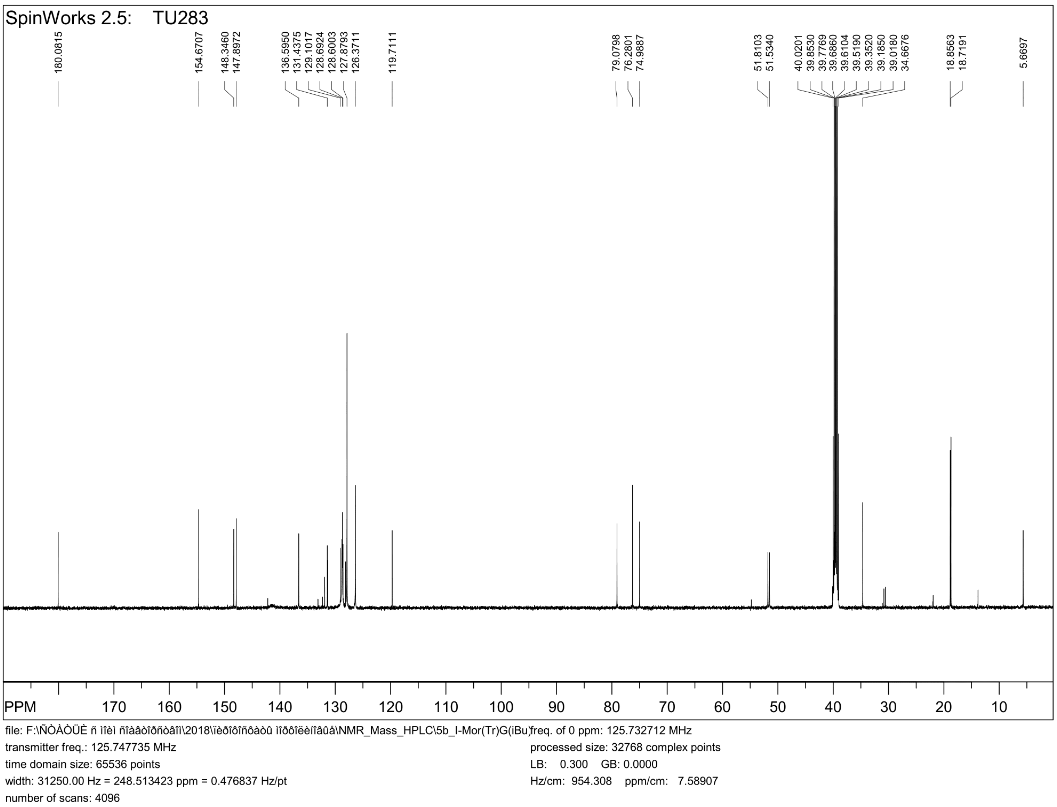
SpinWorks 2.5: TU283
180.0815
154.6707
148.3460
147.8972
136.5950
131.4375
129.1017
128.6924
128.6003
127.8793
126.3711
119.7111
79.0798
76.2801
74.9887
51.8103
51.5340
40.0201
39.8530
39.7769
39.6860
39.6104
39.5190
39.3520
39.1850
39.0180
34.6676
18.8563
18.7191
5.6697
PPM 170 160 150 140 130 120 110 100 90 80 70 60 50 40 30 20 10
file: F:\ÑÒÀÒÜÈ ñ ìîèì ñîàâòîðñòâîì\2018\ïèðîôîñôàòû ìîðôîëèíîâûå\NMR_Mass_HPLC\5b_I-Mor(Tr)G(iBu)
freq. of 0 ppm: 125.732712 MHz
transmitter freq.: 125.747735 MHz
processed size: 32768 complex points
time domain size: 65536 points
LB: 0.300 GB: 0.0000
width: 31250.00 Hz = 248.513423 ppm = 0.476837 Hz/pt
Hz/cm: 954.308 ppm/cm: 7.58907
number of scans: 4096

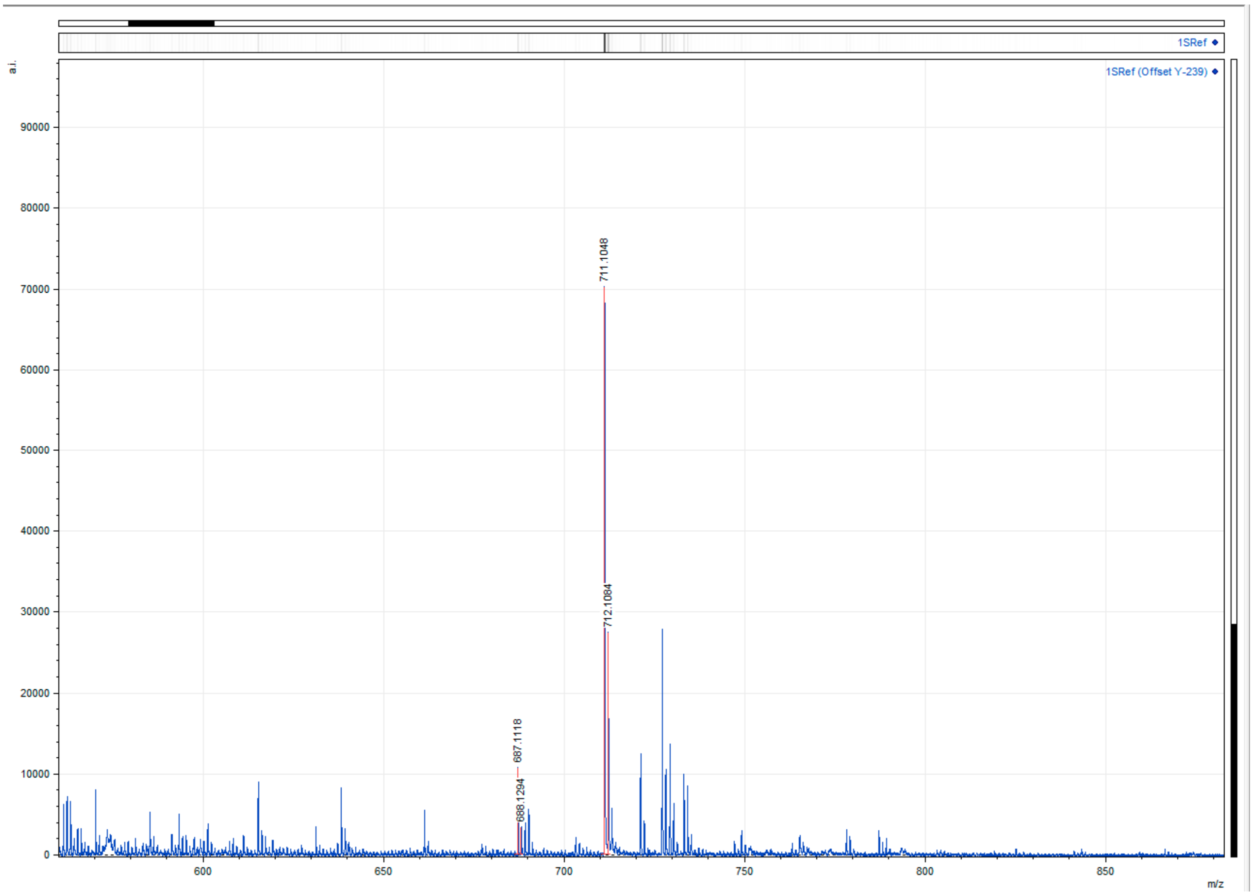
1SRef
1SRef (Offset Y-239)
a.i.
90000
80000
70000
60000
50000
40000
30000
20000
10000
0
711.1048
712.1084
687.1118
688.1294
600
650
700
750
800
850
m/z

2’-Iodomethyl-4’-*N*-trityl-6’-(N^4- benzoylcytosine-1-yl)-morpholine (5C)

^{1}H (400 MHz, DMSO-d_6:aceton-d_6, 2:1): 7.99 (br d, *J* 7.6, *o*-Bz), 7.89 (d, *J* 7.3, 1H, *H6*-Cyt), 7.64 (t, *J* 7.2, 1H, *p*-Bz), 7.54-7.40 (m, 8H, Tr, *m*-Bz), 7.33 (br t, *J* 7.3, 6H, Tr), 7.28 (d, *J* 7.6, 1H, *H5*-Cyt), 7.25-7.18 (m, 3H, Tr), 6.22 (br d, *J* 8.6, H6’), 4.30-4.22 (m, 1H, *H2’*), 3.43-3.12 (m, 6H, CH_2-I, *H5’*, *H3’*), 1.42 (t, *J* 11.2, 1H, *H3’*), 1.30 (t, *J* 10.2, 1H, *H3’*).

^{13}C (100 MHz, DMSO-d_6:aceton-d_6, 2:1): 163.34, 147.87, 144.85, 132.71, 128.94, 128.49, 128.39, 127.84, 127.47, 126.60, 126.40, 81.19, 76.41, 74.88, 51.11, 51.92, 6.06

MS ESI (*m/z*): [M] calcd for $C_{35}H_{31}IN_4O_3$ 682.144; found 682.900.

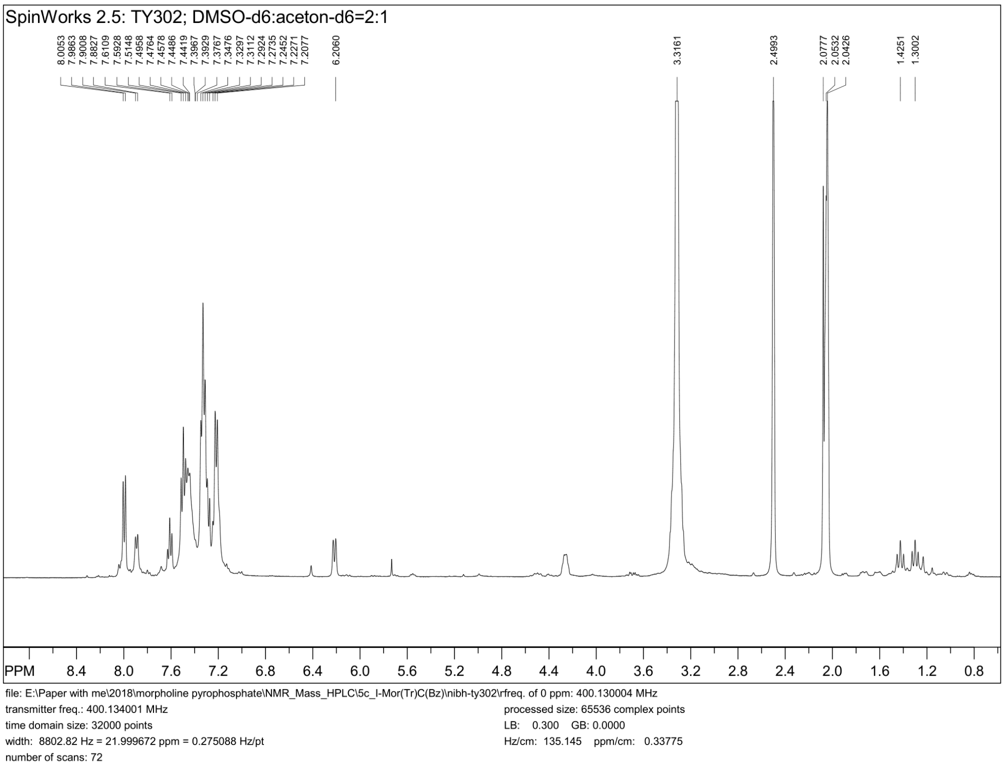

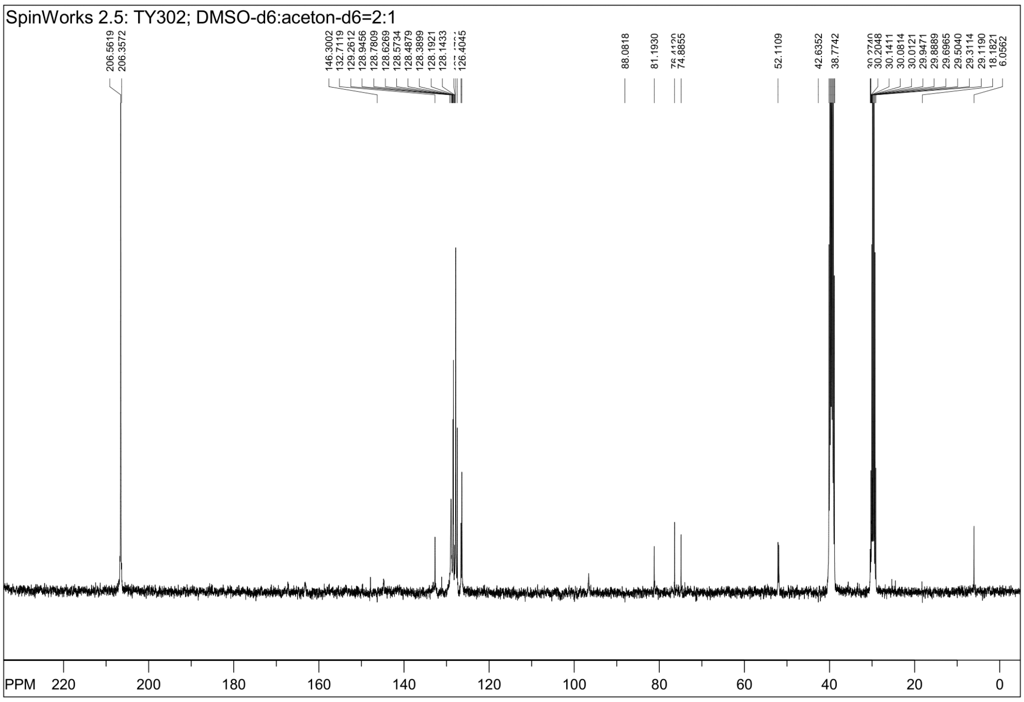

file: E:\Paper with me\2018\morpholine pyrophosphate\NMR_Mass_HPLC\5c_I-Mor(Tr)C(Bz)\nibh-ty302\rfreq. of 0 ppm: 100.612807 MHz
transmitter freq.: 100.624339 MHz
time domain size: 65536 points
width: 24038.46 Hz = 238.893111 ppm = 0.366798 Hz/pt
number of scans: 1904

processed size: 131072 complex points
LB: 2.000 GB: 0.0000
Hz/cm: 961.538 ppm/cm: 9.55572

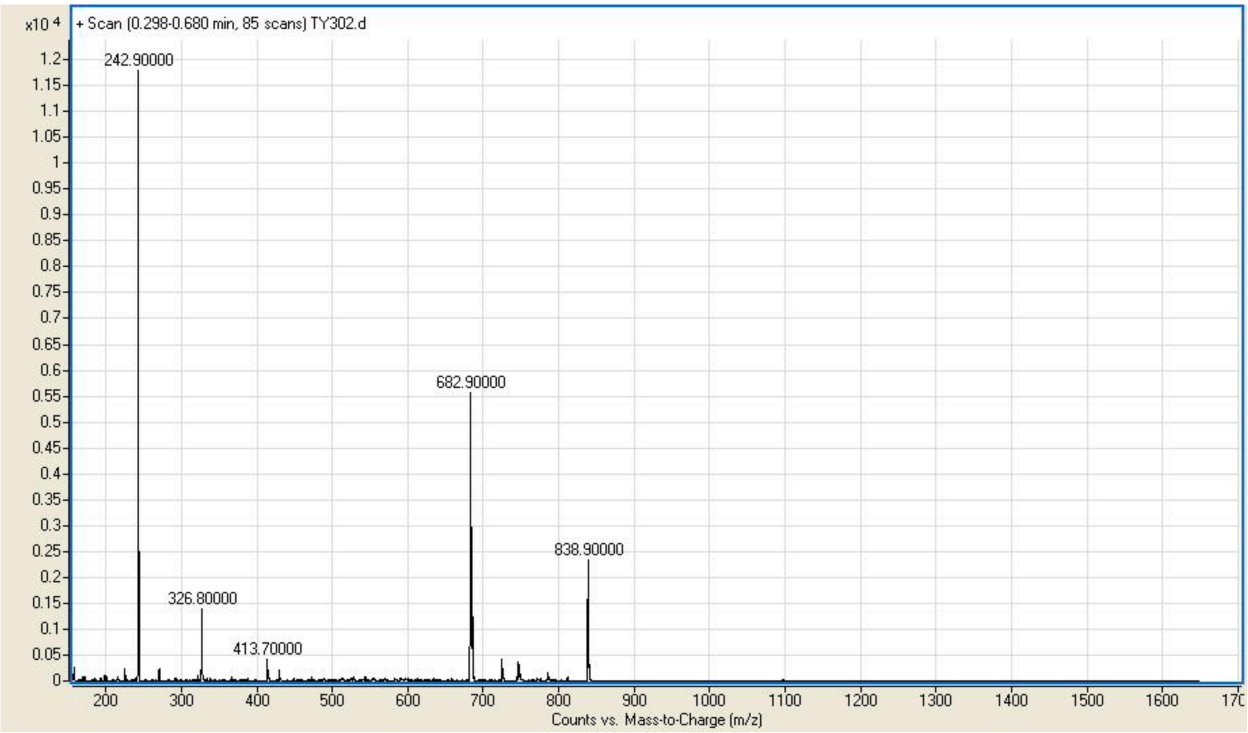

2’-Iodomethyl-4’-*N*-trityl-6’-(uracil-1-yl)-morpholine (5U)

^{1}H (500 MHz, DMSO-d_6): 11.31 (s, 1H, *NH*-Ura), 7.49-7.37 (m, 6H, Tr), 7.32 (m, 7H, Tr, *H6*-Ura), 7.20 (br t, *J* 6.8, 3H, Tr), 6.08 (dd, *J* 9.5, 2.3, 1H, *H6’*), 5.54 (dd, *J* 7.9, 2.0, 1H, *H5*-Ura), 4.20-4.13 (m, 1H, *H2’*), 3.30 (dd, *J* 10.4, 4.9, 1H, *H5’*), 3.24 (dd, *J* 10.4, 5.7, *H3’*), 3.22-3.14 (m, 2H, *CH_2*-I), 1.39-1.31 (m, 2H, *H3’*, *H5’*).

^{13}C (125 MHz, DMSO-d_6): 162.83, 149.69, 139.89, 128.82, 127.79, 126.32, 101.82, 79.74, 76.20, 74.35, 51.78, 51.29, 6.55.

MS ESI (*m/z*): [M] calcd for $C_{28}H_{26}IN_3O_3$ 579.44, found 579.19; $[M+Na]^+$ calcd for $C_{28}H_{26}IN_3NaO_3^+$ 602.43, found 601.99.

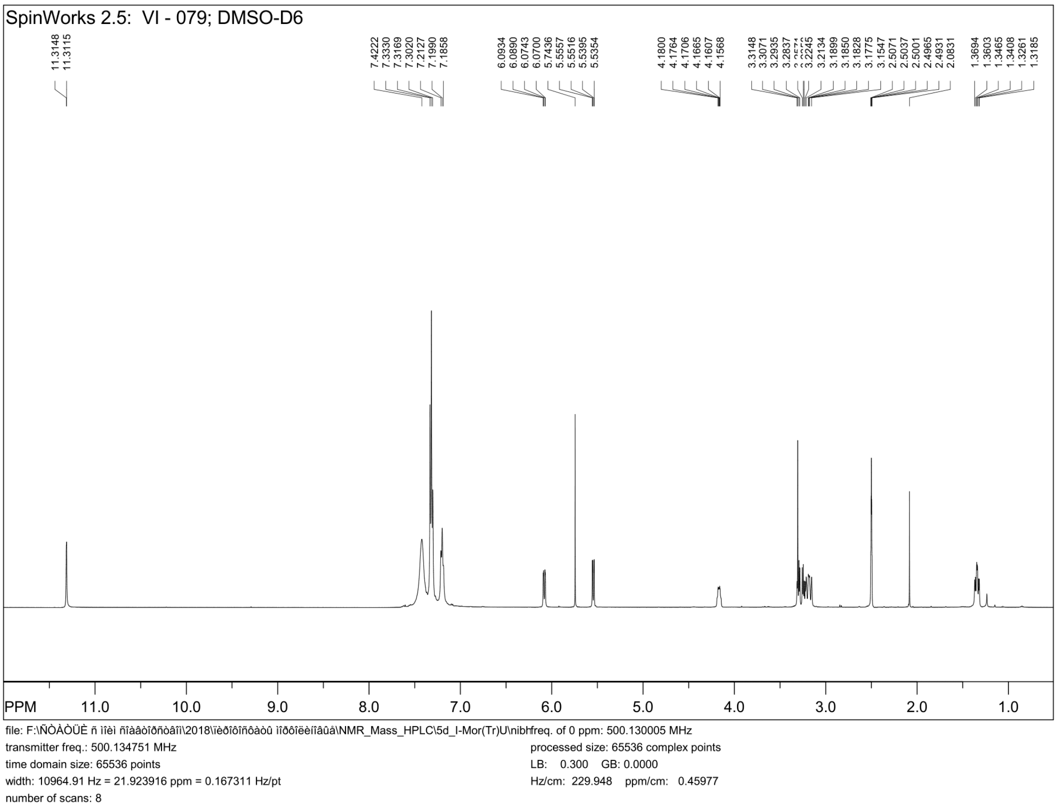

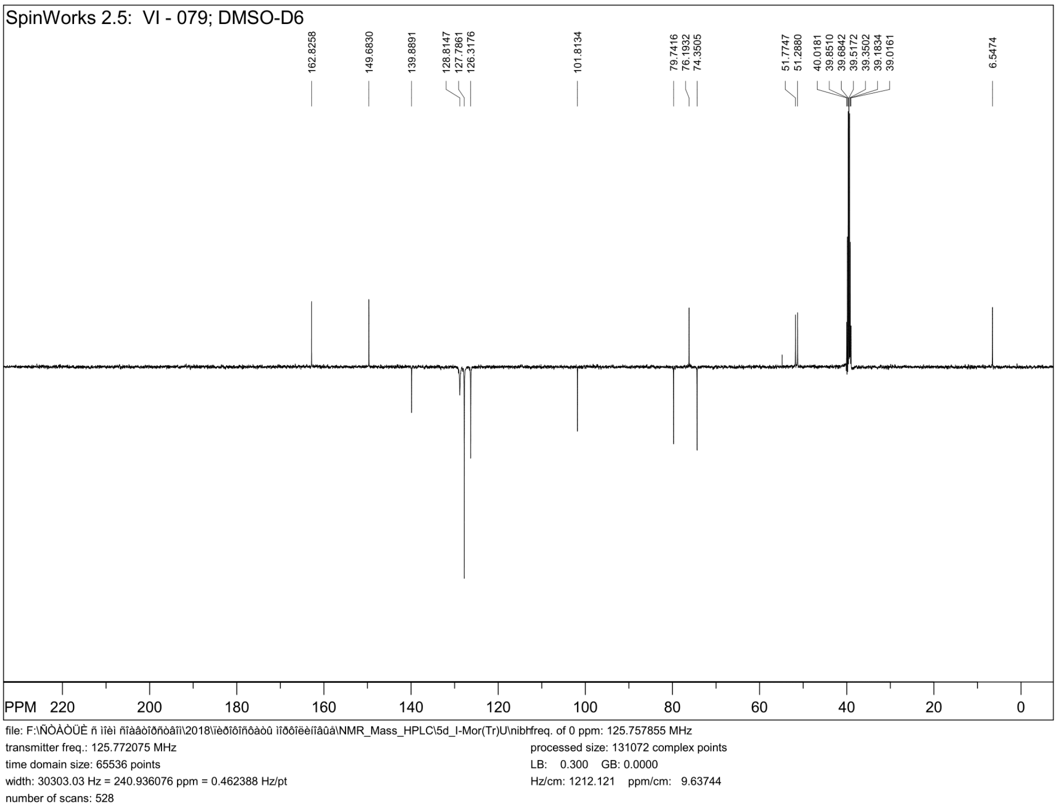
SpinWorks 2.5: VI - 079; DMSO-D6
162.8258
149.6830
139.8891
128.8147
127.7861
126.3176
101.8134
79.7416
76.1932
74.3505
51.7747
51.2880
40.0181
39.8510
39.6842
39.5172
39.3502
39.1834
39.0161
6.5474
PPM 220 200 180 160 140 120 100 80 60 40 20 0
file: F:\ÑÒÀÒÜÈ ñ ìîèì ñîàâòîðñòâîì\2018\ïèðîôîñôàòû ïîðôîëèíîâûå\NMR_Mass_HPLC\5d_I-Mor(Tr)U\nibhfreq. of 0 ppm: 125.757855 MHz
transmitter freq.: 125.772075 MHz
time domain size: 65536 points
width: 30303.03 Hz = 240.936076 ppm = 0.462388 Hz/pt
number of scans: 528
processed size: 131072 complex points
LB: 0.300 GB: 0.0000
Hz/cm: 1212.121 ppm/cm: 9.63744

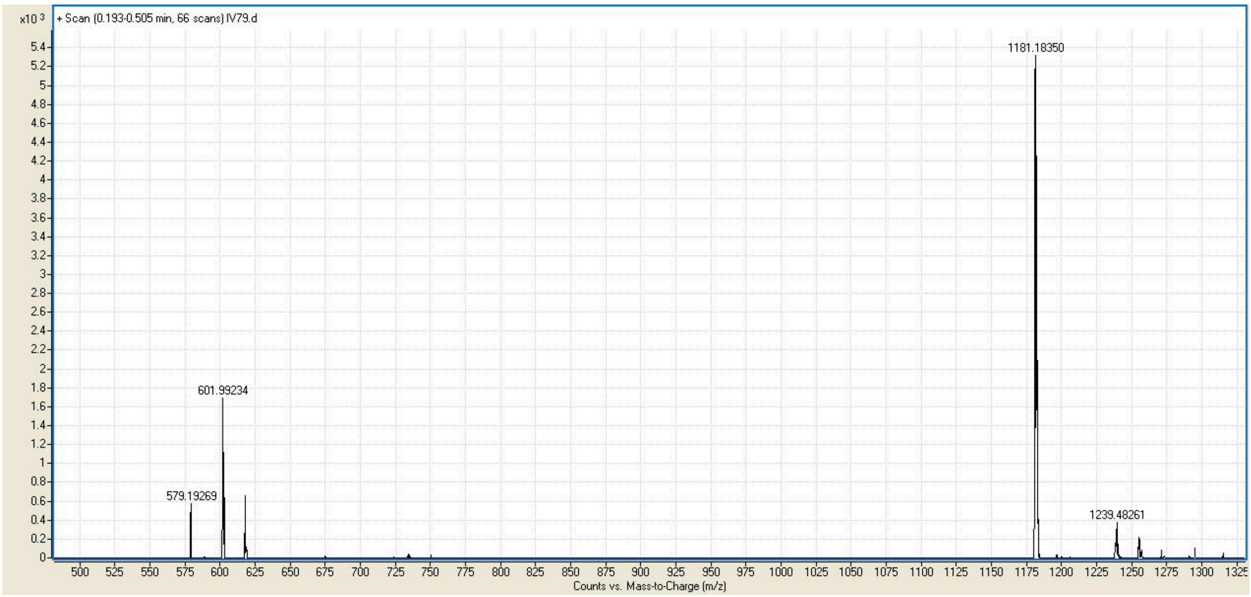
x10 3
+ Scan (0.193-0.505 min, 66 scans) IV79.d
1181.18350
601.99234
579.19269
1239.48261
Counts vs. Mass-to-Charge (m/z)

2’-Iodomethyl-4’-*N*-trityl-6’-(thymine-1-yl)-morpholine (5T)

^{1}H (500 MHz, DMSO-d$_6$): 11.31 (s, 1H, *NH*-Thy), 7.49-7.38 (m, 6H, Tr), 7.32 (br t, *J* 7.7, 6H, Tr), 7.23 (d, *J* 1.2, *H6*-Thy), 7.20 (br t, *J* 7.0, 3H, Tr), 6.08 (dd, *J* 9.6, 2.4, 1H, *H6’*), 4.22-4.16 (m, 1H, *H2’*), 3.29 (dd, *J* 10.0, 5.2, 1H, *H5’*), 3.23 (dd, *J* 10.6, 5.8, *H3’*), 3.22 (dt, *J* 11.8, 2.3, 1H, *CH$_2$*-I), 3.13 (dt, *J* 11.3, 2.3, 1H, *CH$_2$*-I), 1.68 (ap. d, *J* 1.1, 3H, *CH$_3$*-Thy), 1.39 (dd, *J* 11.2, 9.7, 1H, *H3’*), 1.35 (dd, *J* 10.6, 10.0, 1H, *H5’*).

^{13}C (125 MHz, DMSO-d$_6$): 163.41, 149.62, 143.33, 135.29, 128.76, 127.76, 126.26, 109.19, 79.72, 76.12, 74.59, 51.73, 50.07, 11.98, 6.23.

MS MALDI-TOF (*m/z*): [M-H]$^-$ calcd for $C_{29}H_{27}IN_3O_3^-$ 592.110; found 592.068.

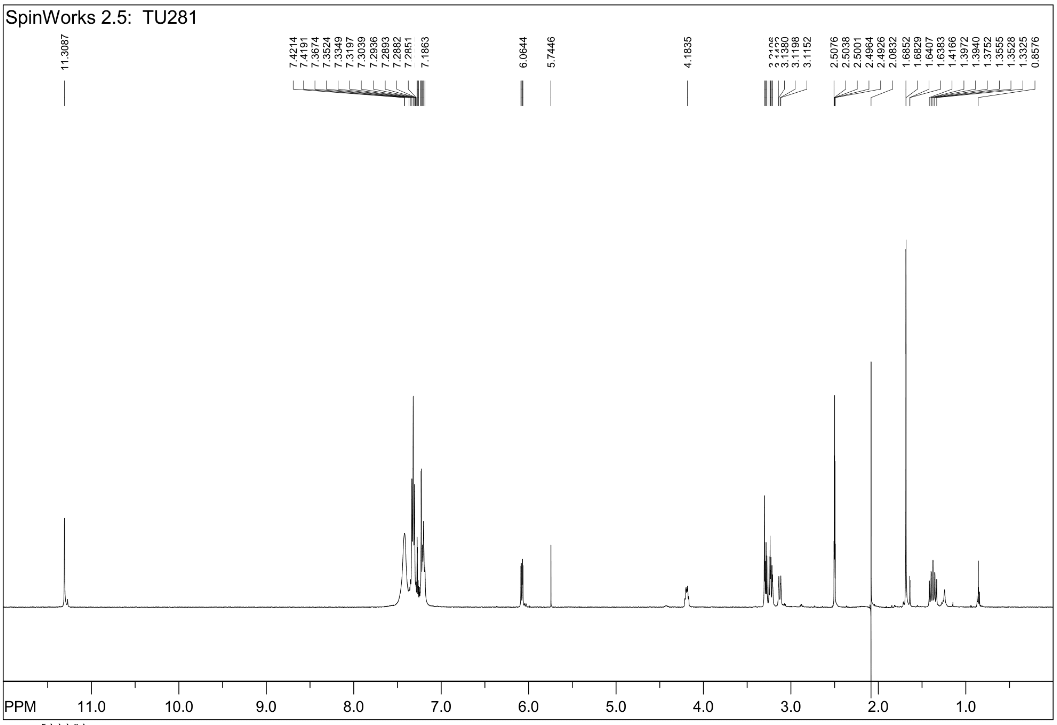

file: F:\ÑÒÀÒÜÈ ñ ïîèì ñîàâòîðñòâîì\2018\ìèðîôîñôàòû ìîðôîëèíîâûå\NMR_Mass_HPLC\5e_I-Mor(Tr)T\TU2freq. of 0 ppm: 500.030003 MHz
transmitter freq.: 500.033001 MHz
time domain size: 65536 points
width: 10330.58 Hz = 20.659793 ppm = 0.157632 Hz/pt
number of scans: 512
processed size: 32768 complex points
LB: 0.300 GB: 0.0000
Hz/cm: 240.164 ppm/cm: 0.48030

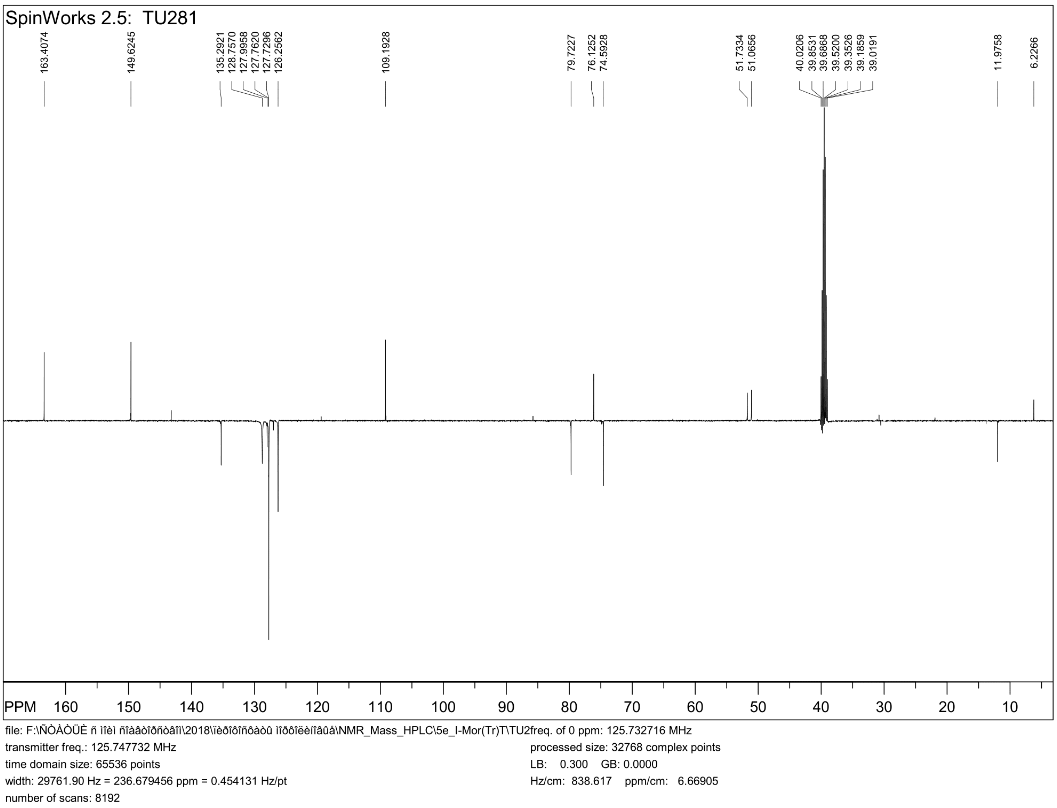
SpinWorks 2.5: TU281
163.4074
149.6245
135.2921
128.7570
127.9958
127.7820
127.7296
126.2562
109.1928
79.7227
76.1252
74.5928
51.7334
51.0656
40.0206
39.8531
39.6868
39.5200
39.3526
39.1859
39.0191
11.9758
6.2266
PPM 160 150 140 130 120 110 100 90 80 70 60 50 40 30 20 10
file: F:\ÑÒÀÒÜÈ ñ ìîèì ñîàâòîðñòâîì\2018\ïèðîîîñàòû ìîðôîëèíîâûå\NMR_Mass_HPLC\5e_l-Mor(Tr)T\TU2freq. of 0 ppm: 125.732716 MHz
transmitter freq.: 125.747732 MHz
time domain size: 65536 points
width: 29761.90 Hz = 236.679456 ppm = 0.454131 Hz/pt
number of scans: 8192
processed size: 32768 complex points
LB: 0.300 GB: 0.0000
Hz/cm: 838.617 ppm/cm: 6.66905

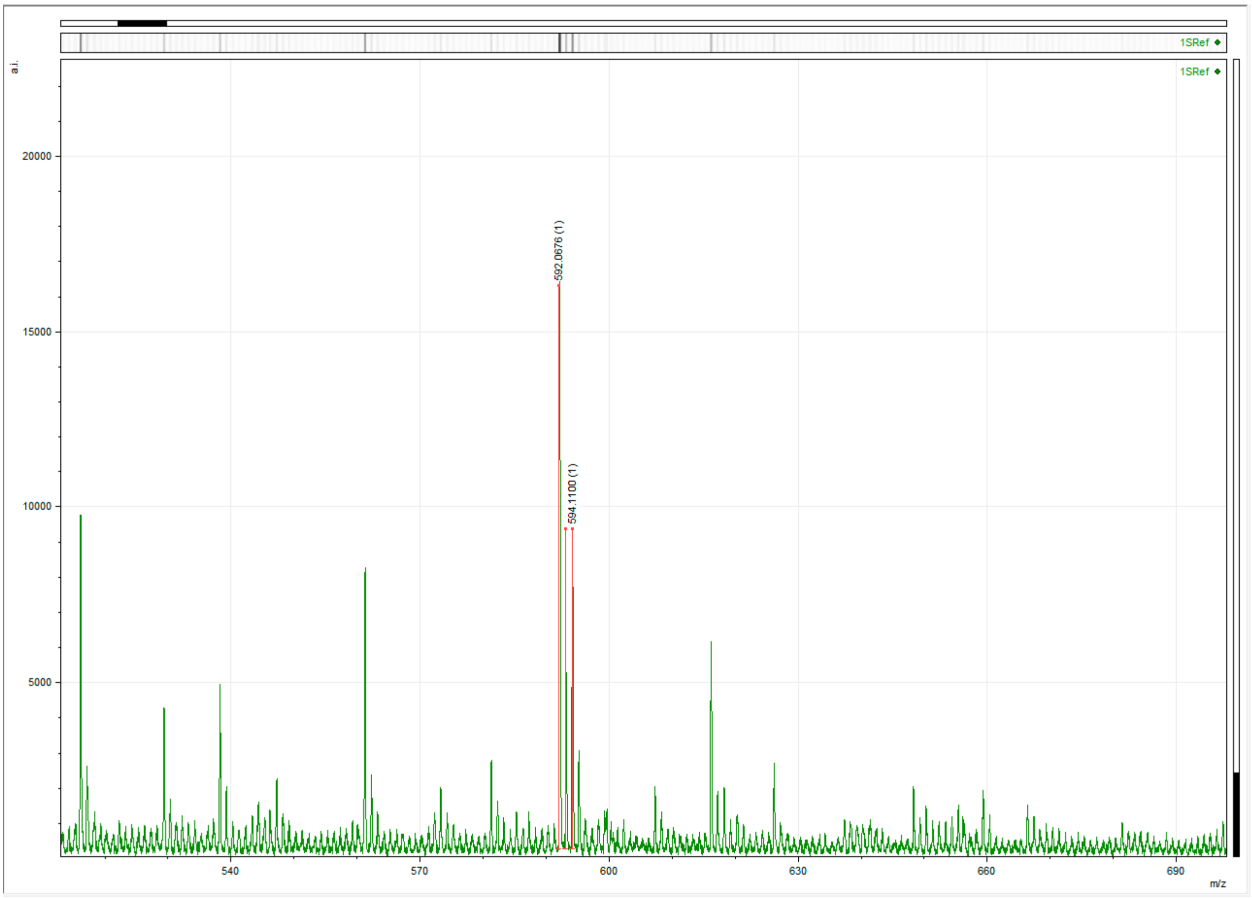
a.i.
1SRef
1SRef
20000
15000
10000
5000
592.0676 (1)
594.1100 (1)
540
570
600
630
660
690
m/z

2’-Iodomethyl-4’-*N*-trityl-6’-(5-iodouracil-1-yl)-morpholine (5IU)

^{1}H (500 MHz, DMSO-d$_6$): 11.69 (s, 1H, *NH*-Ura), 7.77 (s, 1H, *H6*-Ura), 7.48-7.37 (m, 6H, Tr), 7.32 (br t, *J* 7.2, 6H, Tr), 7.20 (br t, *J* 5.8, 3H, Tr), 6.05 (dd, *J* 9.3, 1.4, 1H, *H6’*), 4.20-4.13 (m, 1H, *H2’*), 3.33-3.27 (m, 2H, *H5’, H3’*), 3.19 (br t, *J* 9.3, 2H, *CH$_2$*-I), 1.39 (t, *J* 11.3, 1H, *H3’*), 1.32 (t, *J* 9.8, 1H, *H5’*).

^{13}C (100 MHz, DMSO-d$_6$): 160.22, 149.32, 143.98, 128.79, 127.75, 126.29, 80.20, 76.14, 74.43, 69.52, 51.63, 51.39, 6.65.

MS ESI (m/z): [M+H]$^+$ calcd for $C_{28}H_{26}I_2N_3O_3^+$ 706.006, found 705.700; [M+Na]+ calcd for $C_{28}H_{25}I_2NaN_3O_3^+$ 727.988, found 727.500.

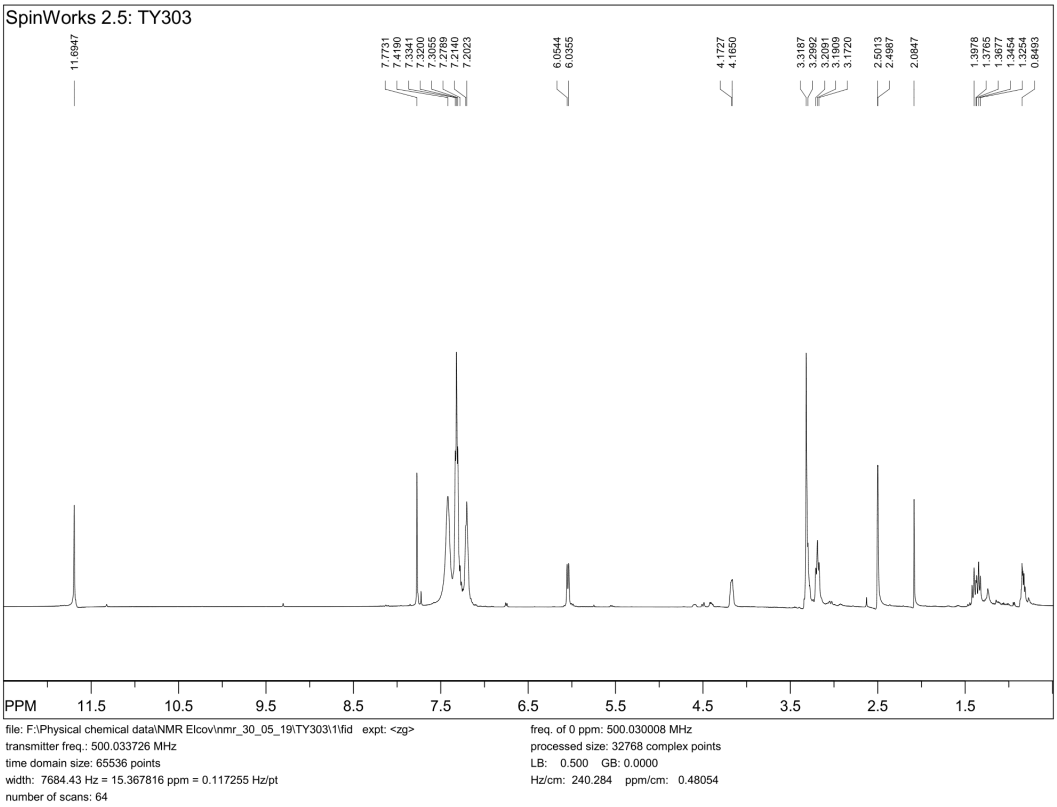

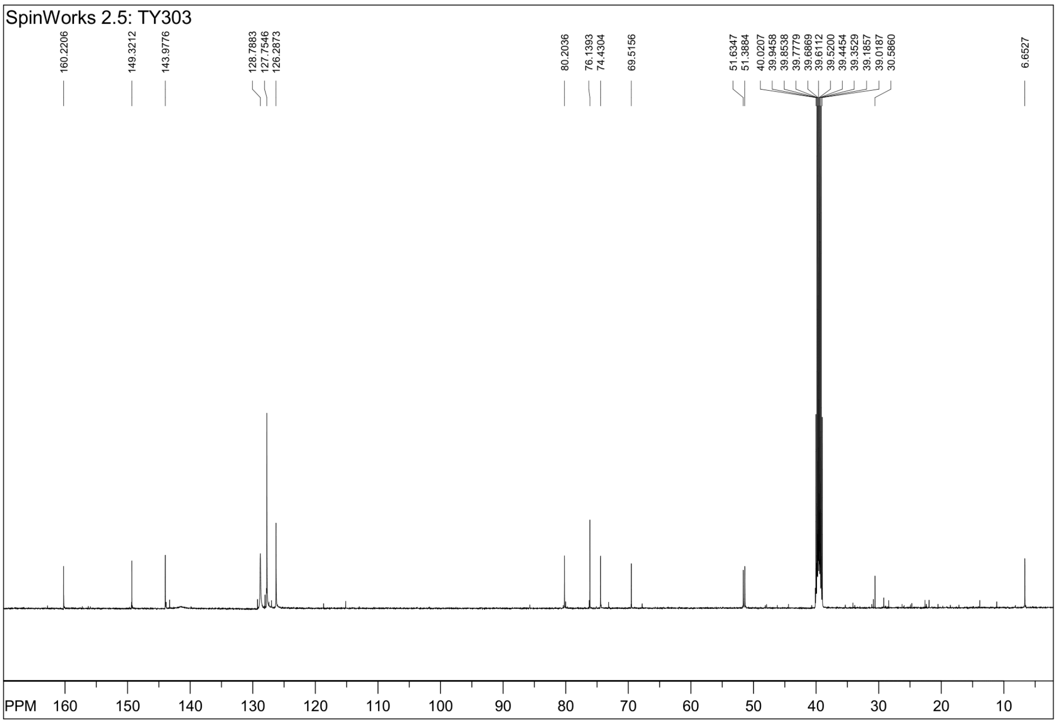

file: F:\Physical chemical data\NMR Elcov\nmr_30_05_19\TY303\1301\fid expt: <zgpg>
transmitter freq.: 125.747735 MHz
time domain size: 65536 points
width: 29761.90 Hz = 236.679450 ppm = 0.454131 Hz/pt
number of scans: 15360

freq. of 0 ppm: 125.732713 MHz
processed size: 32768 complex points
LB: 0.500 GB: 0.0000
Hz/cm: 843.591 ppm/cm: 6.70860

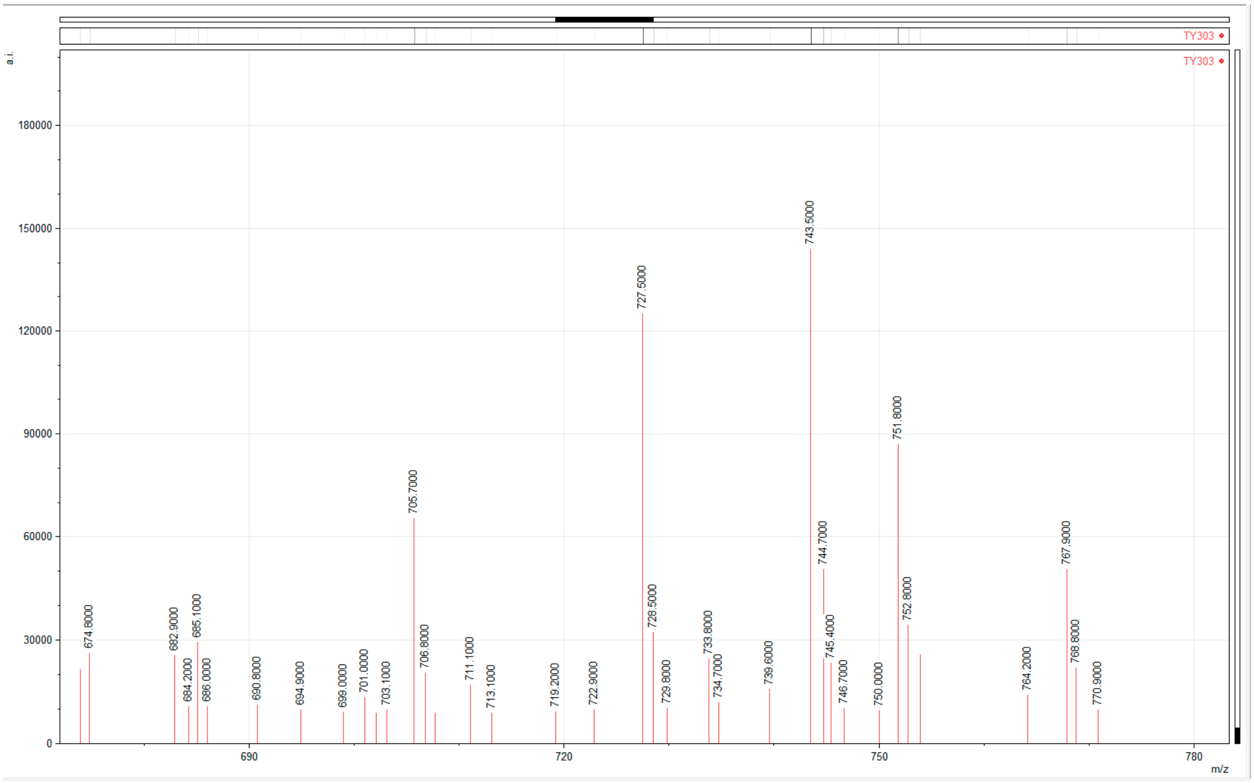

2’-Iodomethyl-4’-*N*-trityl-6’-(5-bromouracil-1-yl)-morpholine (5BrU)

^{1}H (500 MHz, DMSO-d_6): 7.78 (s, 1H, *H6*-Ura), 7.50-7.37 (m, 6H, Tr), 7.32 (br t, *J* 7.5, 6H, Tr), 7.20 (br t, *J* 6.7, 3H, Tr), 6.06 (dd, *J* 9.4, 2.0, 1H, *H6’*), 4.24-4.16 (m, 1H, *H2’*), 3.32-3.25 (m, 2H, *CH$_2$*-I), 3.20 (br tt, *J* 11.0, 1.7, 2H, *H5’, H3’*), 1.39 (t, *J* 11.8, 1H, *H3’*), 1.37 (t, *J* 11.2, 1H, *H5’*).

^{13}C (125 MHz, DMSO-d_6): 158.83, 148.94, 139.38, 128.77, 127.72, 126.25, 95.81, 80.26, 76.12, 74.59, 54.76, 51.57, 51.31, 6.37.

MS MALDI-TOF (*m/z*): [M] calcd for $C_{28}H_{25}BrIN_3O_3$ 657.012; found 657.997.

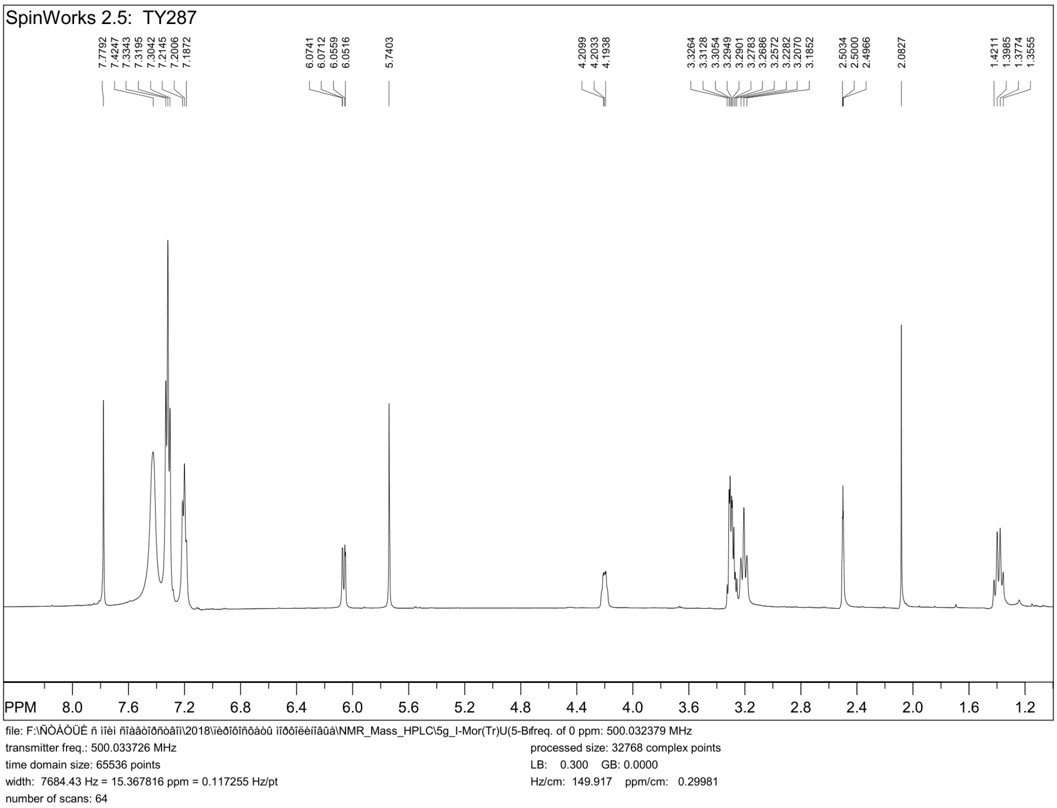

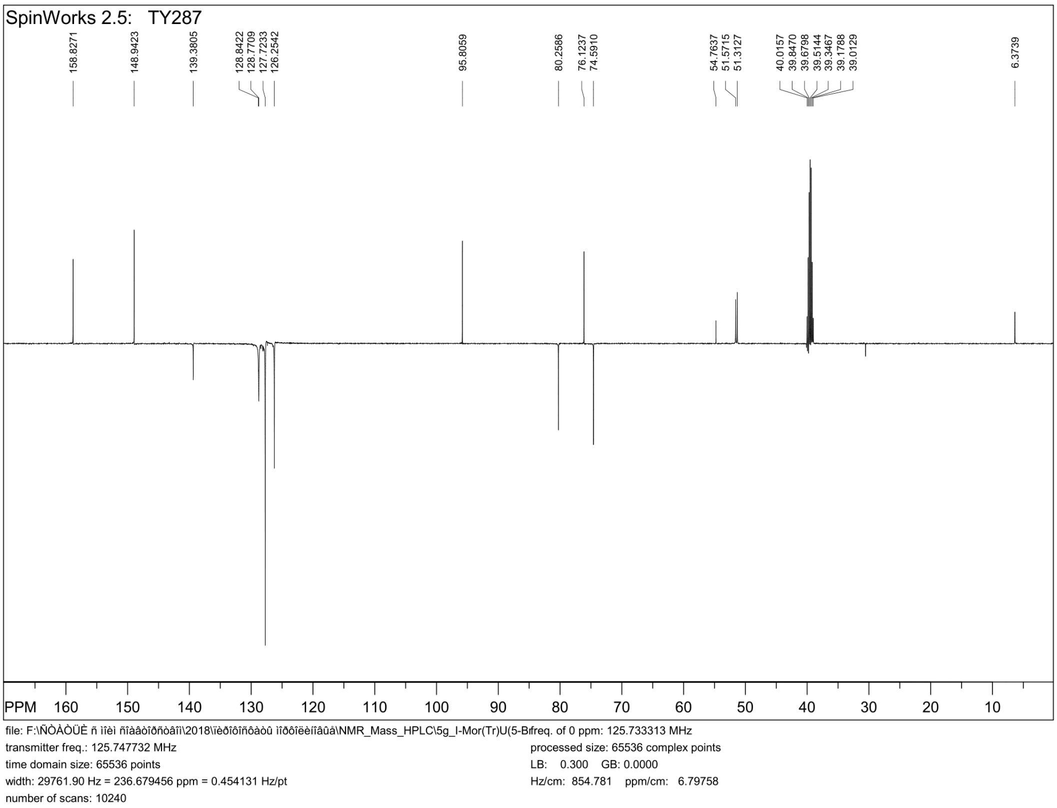
SpinWorks 2.5: TY287
158.8271
148.9423
139.3805
128.8422
128.7709
127.7233
126.2542
95.8059
80.2586
76.1237
74.5910
54.7637
51.5715
51.3127
40.0157
39.8470
39.6798
39.5144
39.3467
39.1788
39.0129
6.3739
PPM 160 150 140 130 120 110 100 90 80 70 60 50 40 30 20 10
file: F:\ÑÒÀÒÜÈ ñ ïîèì ñîâàòîðñòâî\2018\ïèðîöîñîàòû ïîðôîëèíîâûå\NMR_Mass_HPLC\5g_I-Mor(Tr)U(5-Bfreq. of 0 ppm: 125.733313 MHz
transmitter freq.: 125.747732 MHz
time domain size: 65536 points
width: 29761.90 Hz = 236.679456 ppm = 0.454131 Hz/pt
number of scans: 10240
processed size: 65536 complex points
LB: 0.300 GB: 0.0000
Hz/cm: 854.781 ppm/cm: 6.79758

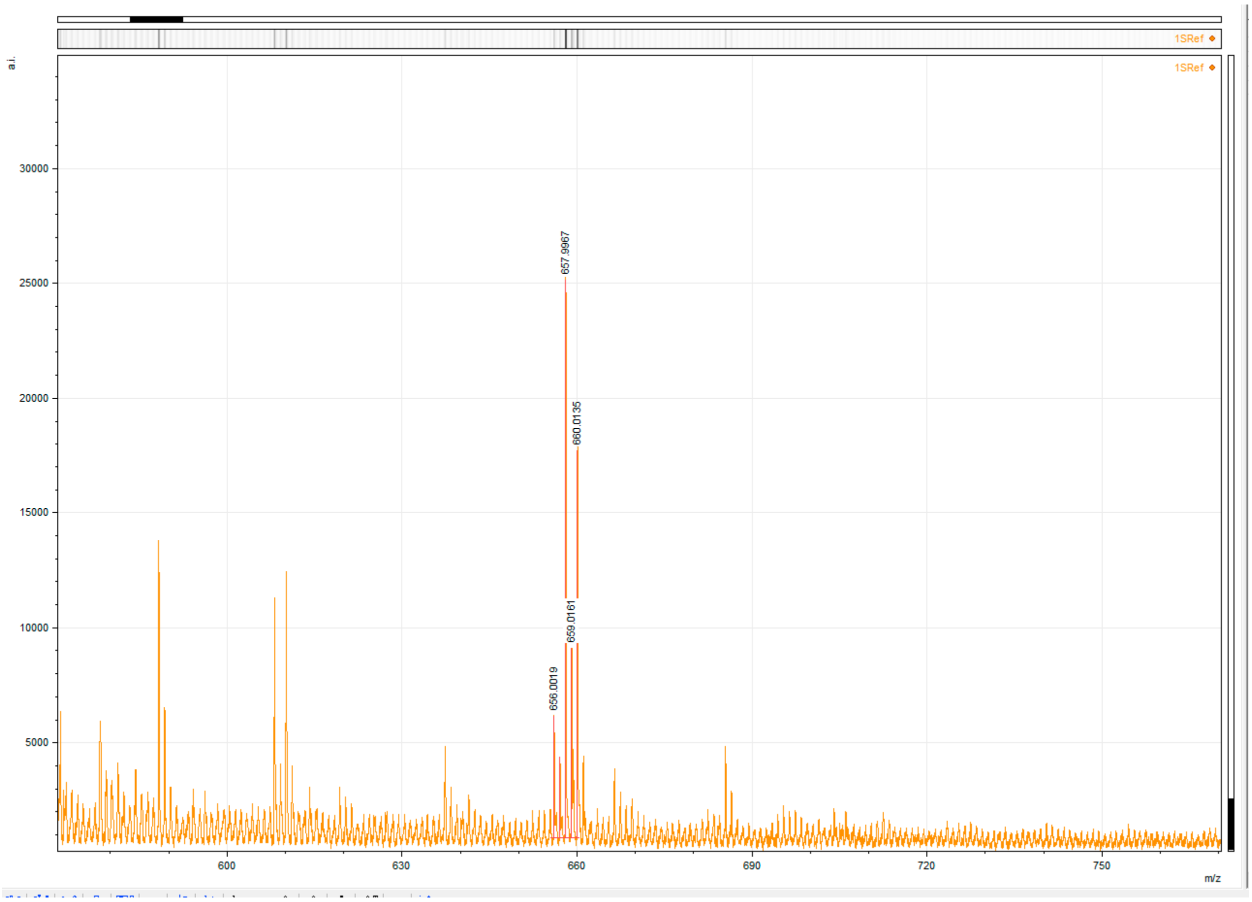
a.i.
1SRef
30000
25000
20000
15000
10000
5000
657.9967
660.0135
659.0161
656.0019
600
630
660
690
720
750
m/z

2’-Iodomethyl-4’-*N*-trityl-6’-(5-chlorouracil-1-yl)-morpholine (5ClU)

^{1}H (500 MHz, DMSO-d_6): 7.72 (s, 1H, *H6*-Ura), 7.49-7.37 (m, 6H, Tr), 7.32 (br t, *J* 7.4, 6H, Tr), 7.20 (br t, *J* 6.3, 3H, Tr), 6.06 (dd, *J* 9.4, 2.0, 1H, *H6’*), 4.25-4.18 (m, 1H, *H2’*), 3.31-3.25 (m, 2H, CH_2-I), 3.23-3.17 (m, 2H, *H5’, H3’*), 1.42-1.34 (m, 2H, *H3’*, *H5’*).

^{13}C (125 MHz, DMSO-d_6): 158.79, 148.79, 137.12, 128.79, 127.75, 126.28, 107.26, 80.23, 76.14, 74.66, 51.58, 51.29, 6.37.

MS ESI (m/z): [M-H]$^-$ calcd for $C_{28}H_{24}ClIN_3O_3^-$ 612.88, found 612.09; [M-2H+Na]$^-$ calcd for $C_{28}H_{24}ClINaN_3O_3^-$ 634.86, found 634.69.

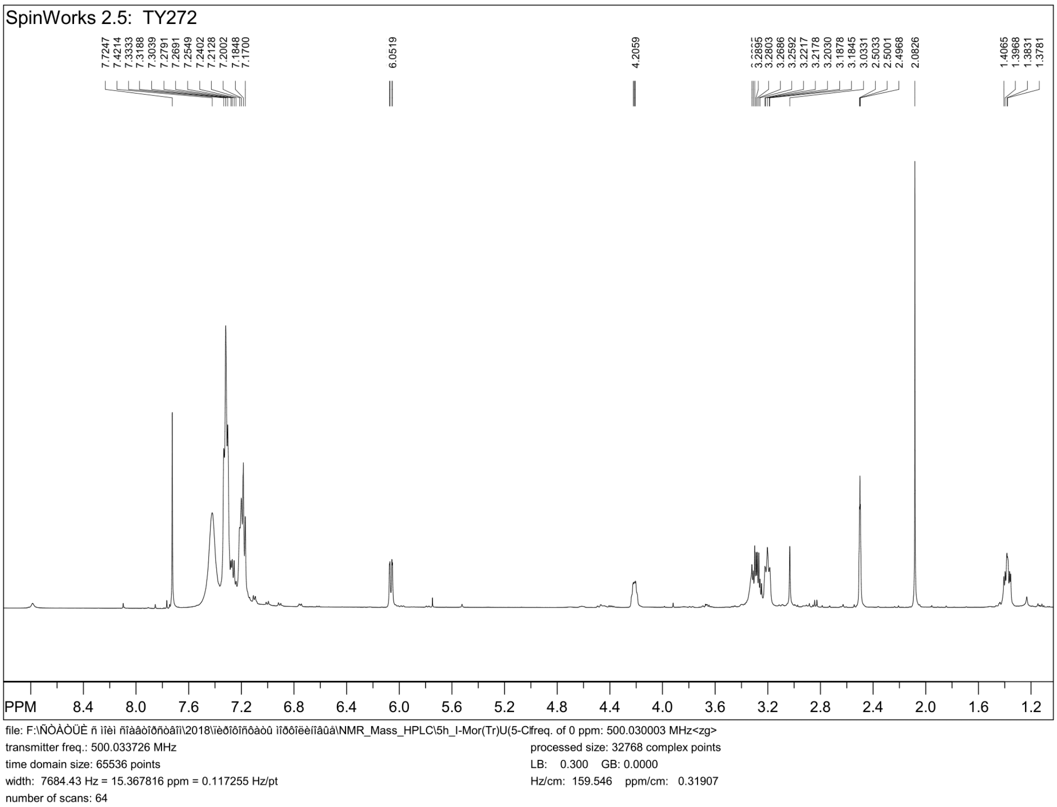

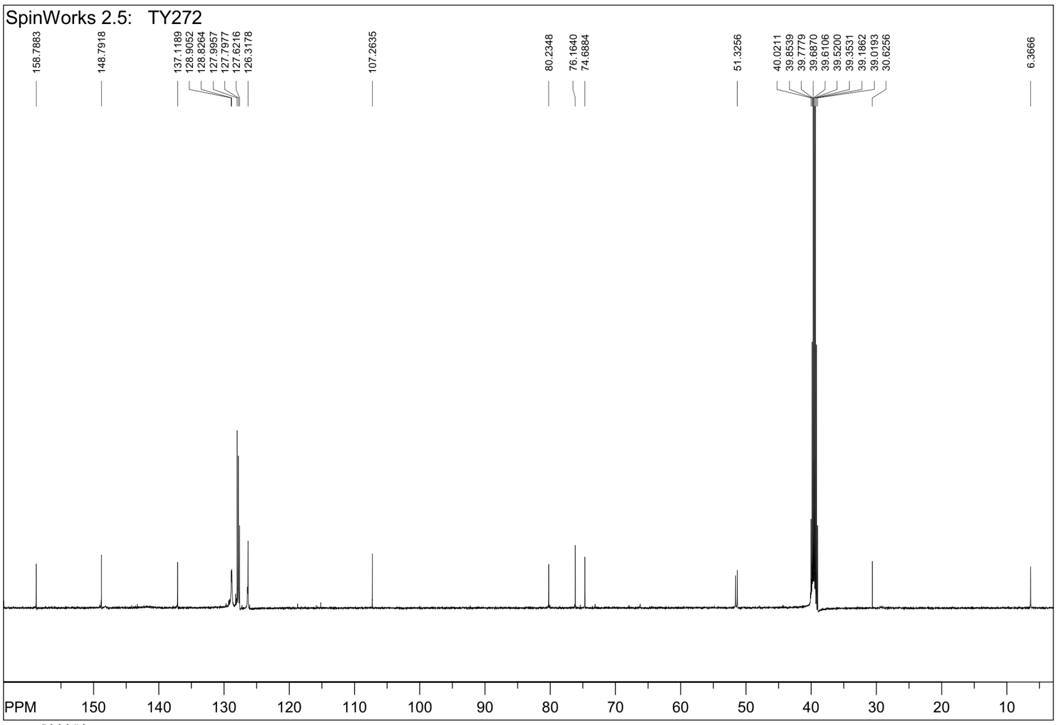

file: F:\ÑÒÀÒÜË ñ ïîèì ñîàâàòîðñòâîì\2018\ìèðîïîñòàòü ïîðôîëèìâûà\NMR_Mass_HPLC\5h_I-Mor(Tr)U(5-Clfreq. of 0 ppm: 125.732708 MHzpt: <zgpg>

transmitter freq.: 125.747735 MHz

time domain size: 65536 points

width: 29761.90 Hz = 236.679450 ppm = 0.454131 Hz/pt

number of scans: 2048

processed size: 32768 complex points

LB: 0.300 GB: 0.0000

Hz/cm: 809.399 ppm/cm: 6.43669

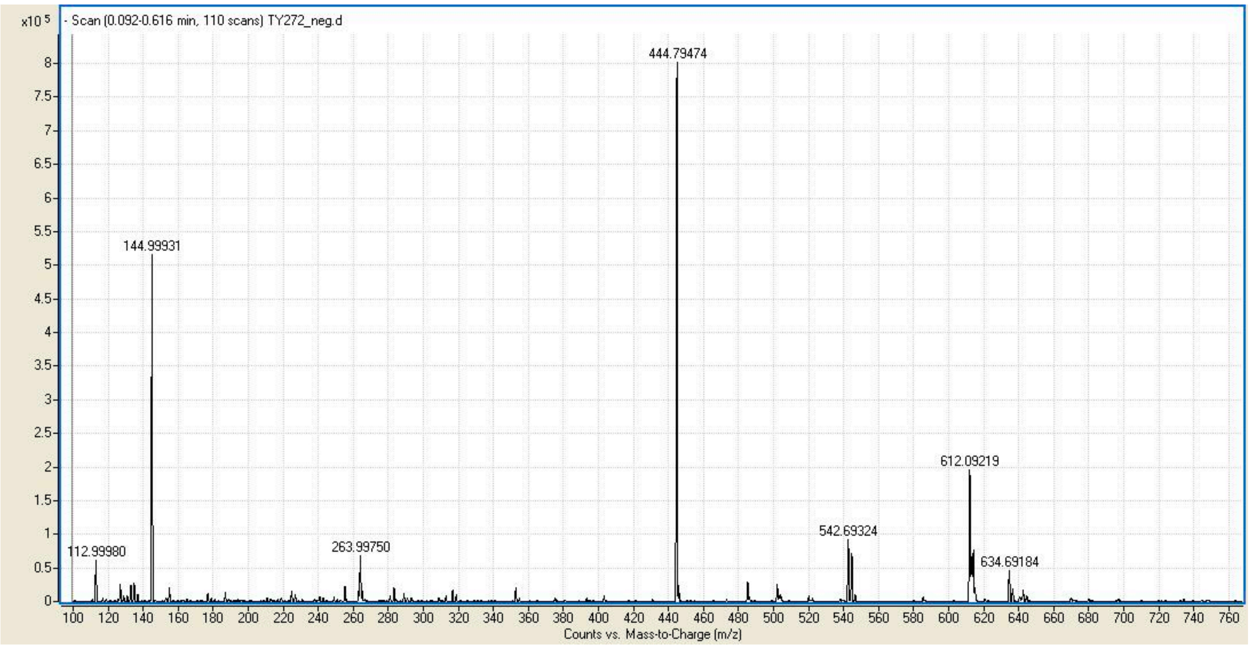

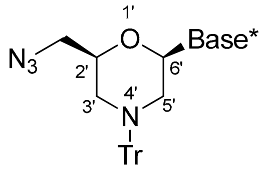

6A,G,C,U,T,IU,BrU,ClU

2'-Azidomethyl-4'-*N*-trityl-6'-(N^6-benzoyladenine-9-yl)-morpholine (6A)

^{1}H (500 MHz, DMSO-d_6): 11.18 (s, 1H, *NH*), 8.75 (s, 1H, *H8*-Ade), 8.42 (s, 1H, *H2*-Ade), 8.02 (d, *J* 7.5, 2H, *o*-Bz), 7.63 (t, *J* 7.2, 1H, *p*-Bz), 7.53 (t, *J* 7.7, 2H, *m*-Bz), 7.51-7.42 (m, 6H, Tr), 7.34 (br t, *J* 7.4, 6H, Tr), 7.21 (br t, *J* 6.2, 3H, Tr), 6.48 (dd, *J* 9.8, 1.5, 1H, *H6'*), 4.58-4.51 (m, 1H, *H2'*), 3.47-3.35 (m, 3H, CH_2-N_3, *H5'*), 3.09 (br d, *J* 11.6, 1H, *H3'*), 2.09 (t, *J* 10.6, 1H, *H3'*), 1.53 (t, *J* 11.0, 1H, *H5'*).

^{13}C (125 MHz, DMSO-d_6): 165.51, 151.80, 151.54, 150.39, 142.00, 133.28, 132.36, 128.90, 128.41, 128.39, 127.87, 126.37, 125.15, 79.80, 76.41, 74.77, 51.65, 51.55, 49.28.

IR ν_{max}/cm^{-1} 2100 (N_3)

MS ESI (m/z): [M] calcd for $C_{36}H_{31}N_9O_2$ 621.260, found 621.900.

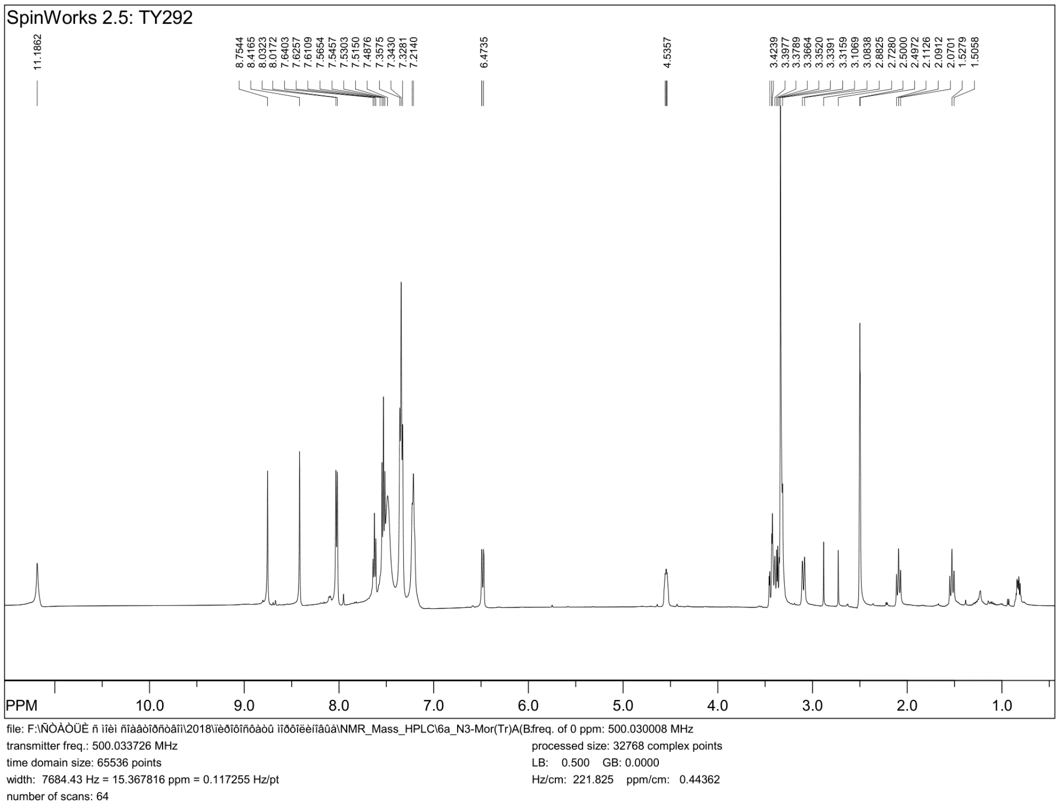

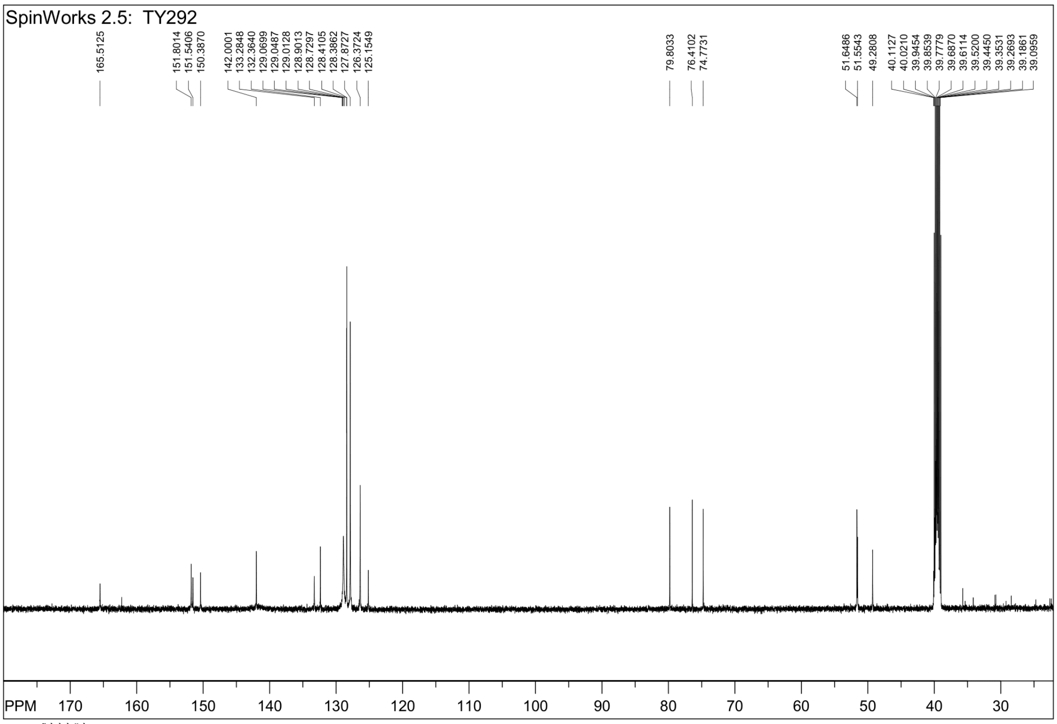

file: F:\ÑÒÀÒÜÈ ñ ìîèì ñîàâòîðñòâîì\2018\ïèðîôîñôàòû ìîðôîëèíîâûå\NMR_Mass_HPLC\6a_N3-Mor(Tr)A(B;freq. of 0 ppm: 125.732708 MHz
transmitter freq.: 125.747735 MHz
time domain size: 65536 points
width: 31250.00 Hz = 248.513423 ppm = 0.476837 Hz/pt
number of scans: 2048

processed size: 32768 complex points
LB: 0.000 GB: 0.0000
Hz/cm: 794.386 ppm/cm: 6.31730

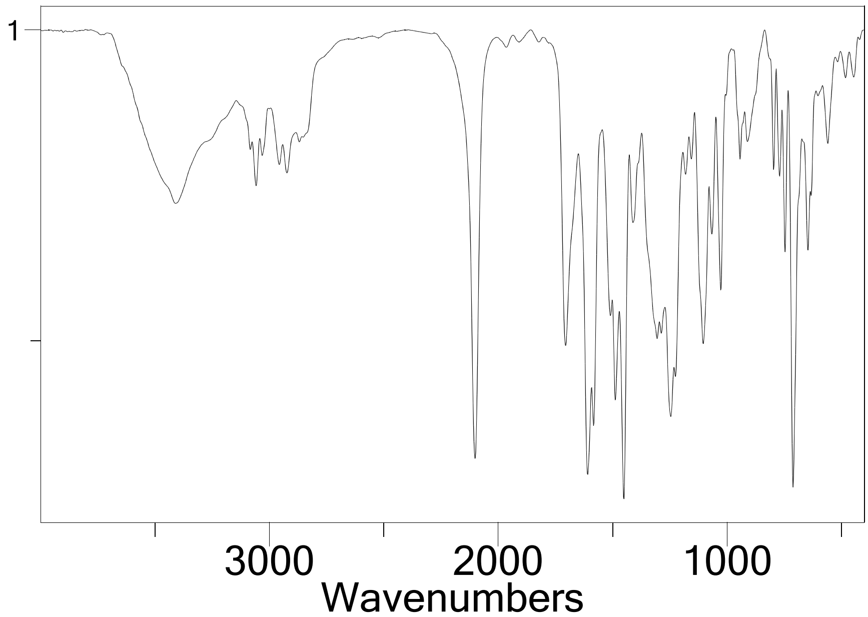

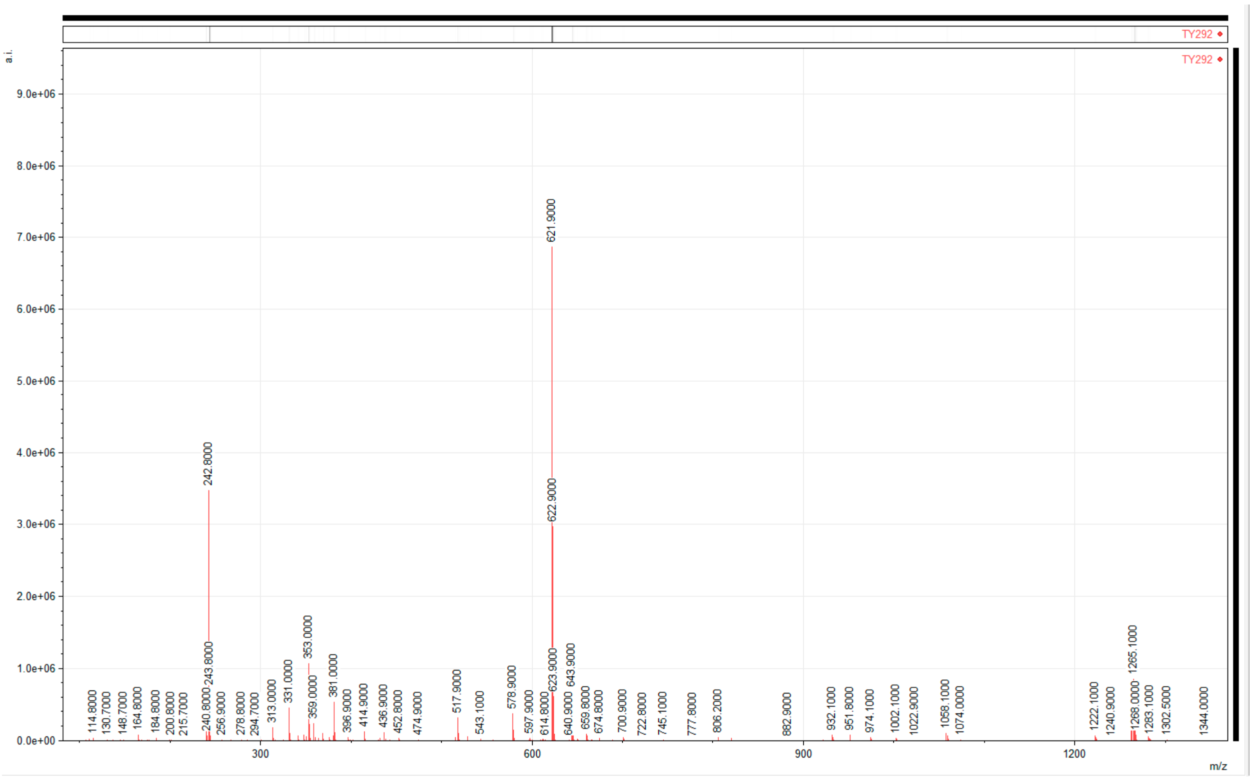
TY292
a.i.
9.0e+06
8.0e+06
7.0e+06
6.0e+06
5.0e+06
4.0e+06
3.0e+06
2.0e+06
1.0e+06
0.0e+00
300
600
900
1200
m/z
621.9000
622.9000
242.8000
243.8000
353.0000
381.0000
331.0000
1265.1000
643.9000
623.9000

2’-Azidomethyl-4’-*N*-trityl-6’-(N^2-isobutyrylguanine-9-yl)-morpholine (6G)

^{1}H (500 MHz, DMSO-d_6): 12.12 (s, 1H, *NH*), 11.73 (s, 1H, *NH*-Gua), 7.88 (s, 1H, *H8*-Gua), 7.48-7.39 (m, 6H, Tr), 7.34 (br t, *J* 6.7, 6H, Tr), 7.21 (br t, *J* 6.4, 3H, Tr), 6.08 (br d, *J* 9.9, 1H, *H6’*), 4.44-4.34 (m, 1H, *H2’*), 3.45 (dd, *J* 12.9, 3.5, 1H, *CH$_2$*-N$_3$), 3.37-3.34 (m , 1H, *CH$_2$*-N$_3$), 3.28 (br d, *J* 11.3, 1H, *H3’*), 3.05 (br d, *J* 11.6, 1H, *H5’*), 2.85 (p, *J* 6.8, 1H, *CH*-iBu), 1.93 (t, *J* 10.8, 1H, *H3’*), 1.46 (t, *J* 11.2, 1H, *H5’*), 1.16 (br d, *J* 5.6, 6H, 2×*CH$_3$*-iBu).

^{13}C (125 MHz, DMSO-d_6): 180.14, 154.74, 148.46, 147.95, 136.52, 128.79, 127.91, 126.38, 119.68, 79.09, 76.34, 74.84, 51.63, 51.58, 49.24, 34.72, 18.89, 18.75.

IR ν_{max}/cm^{-1} 2100 (N$_3$)

MS ESI (m/z): [M] calcd for $C_{33}H_{33}N_9O_3$ 603.271, found 603.800.

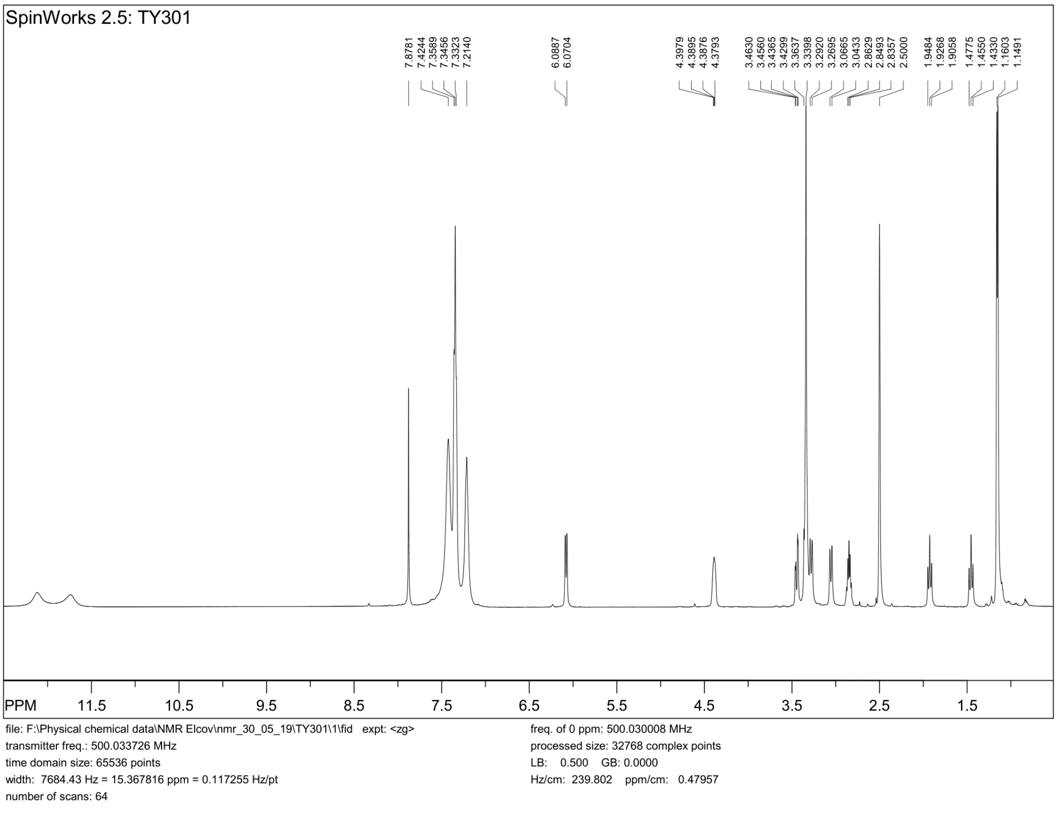

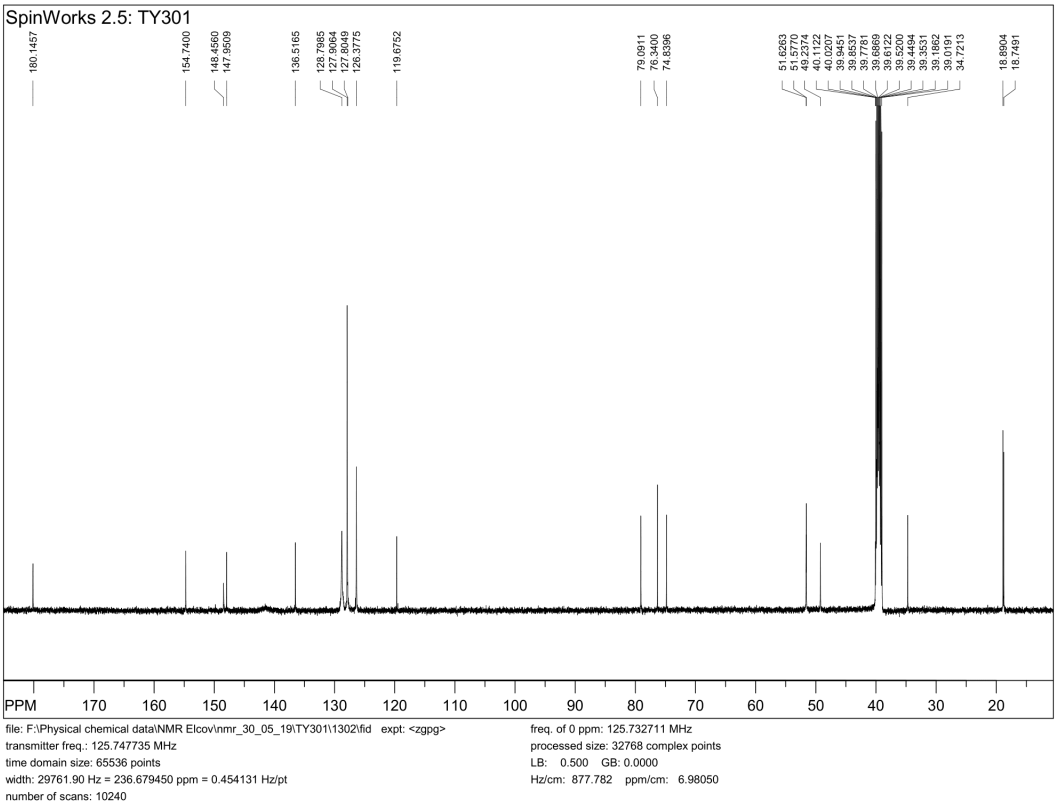
SpinWorks 2.5: TY301
180.1457
154.7400
148.4560
147.9509
136.5165
128.7985
127.9064
127.8049
126.3775
119.6752
79.0911
76.3400
74.8396
51.6263
51.5770
49.2374
40.1122
40.0207
39.9451
39.8537
39.7781
39.6869
39.6122
39.5200
39.4494
39.3531
39.1862
39.0191
34.7213
18.8904
18.7491
PPM 170 160 150 140 130 120 110 100 90 80 70 60 50 40 30 20
file: F:\Physical chemical data\NMR Elcov\nmr_30_05_19\TY301\1302\fid expt: <zgpg>
transmitter freq.: 125.747735 MHz
time domain size: 65536 points
width: 29761.90 Hz = 236.679450 ppm = 0.454131 Hz/pt
number of scans: 10240
freq. of 0 ppm: 125.732711 MHz
processed size: 32768 complex points
LB: 0.500 GB: 0.0000
Hz/cm: 877.782 ppm/cm: 6.98050

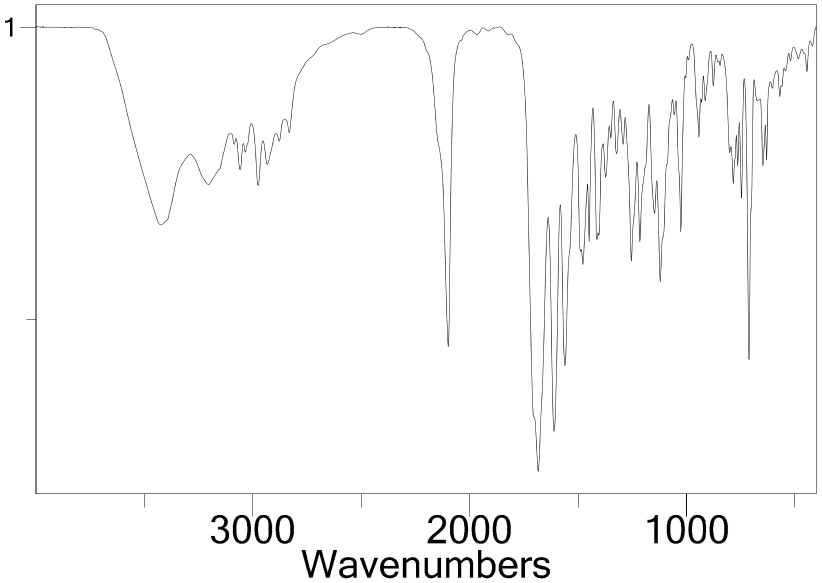
1
3000
2000
1000
Wavenumbers

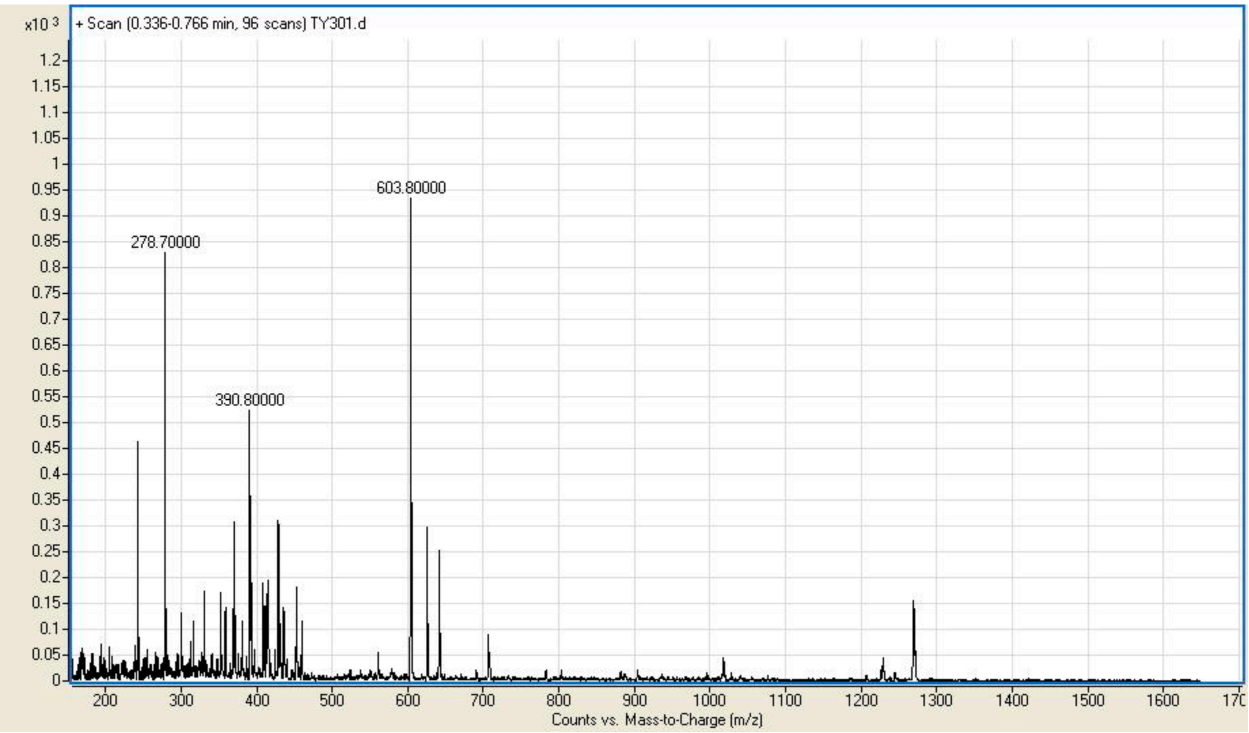
x10 3
+ Scan (0.336-0.766 min, 96 scans) TY301.d
603.80000
278.70000
390.80000
Counts vs. Mass-to-Charge (m/z)

2’-Azidomethyl-4’-*N*-trityl-6’-(*N*^{4}-benzoylcytosine-1-yl)-morpholine (6C)

^{1}H (500 MHz, DMSO-d$_6$): 11.23 (s, 1H, *NH*), 7.96 (br d, *J* 7.4, 2H, *o*-Bz), 7.89 (d, *J* 7.3, 1H, *H6*-Cyt), 7.61 (t, *J* 7.3, 1H, *p*-Bz), 7.49 (t, *J* 7.3, 2H, *m*-Bz), 7.47-7.13 (m, 16H, Tr, *H5*-Cyt), 6.18 (dd, *J* 9.5, 1.9, *H6’*), 4.53-4.42 (m, 1H, *H4’*), 3.03 (dt, *J* 11.7, 2.2, 1H, *H5’*), 1.76-0.97 (m, 5H, *H3’*,*H5’*, *CH$_2$*-N$_3$).

^{13}C (125 MHz, DMSO-d$_6$): 167.40, 163.23, 156.71, 153.76, 144.82, 133.12, 132.82, 128.93, 128.49, 127.96, 96.60, 81.19, 76.42, 75.02, 52.12, 51.81, 49.30, 47.56.

IR ν_{max}/cm^{-1} 2100 (N$_3$)

MS ESI (m/z): [M] calcd for $C_{35}H_{31}N_7O_3$ 597.25, found 597.90; [M-H+Na] calcd for $C_{35}H_{30}NaN_7O_3$ 620.23, found 619.80.

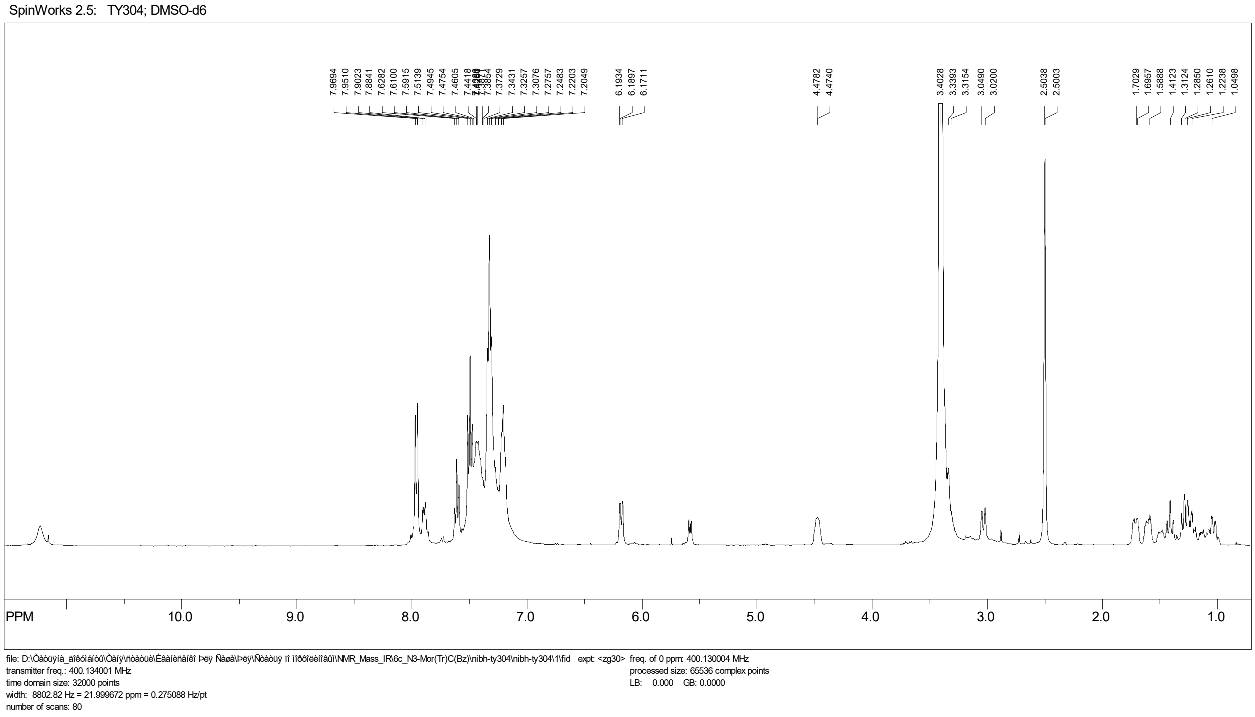

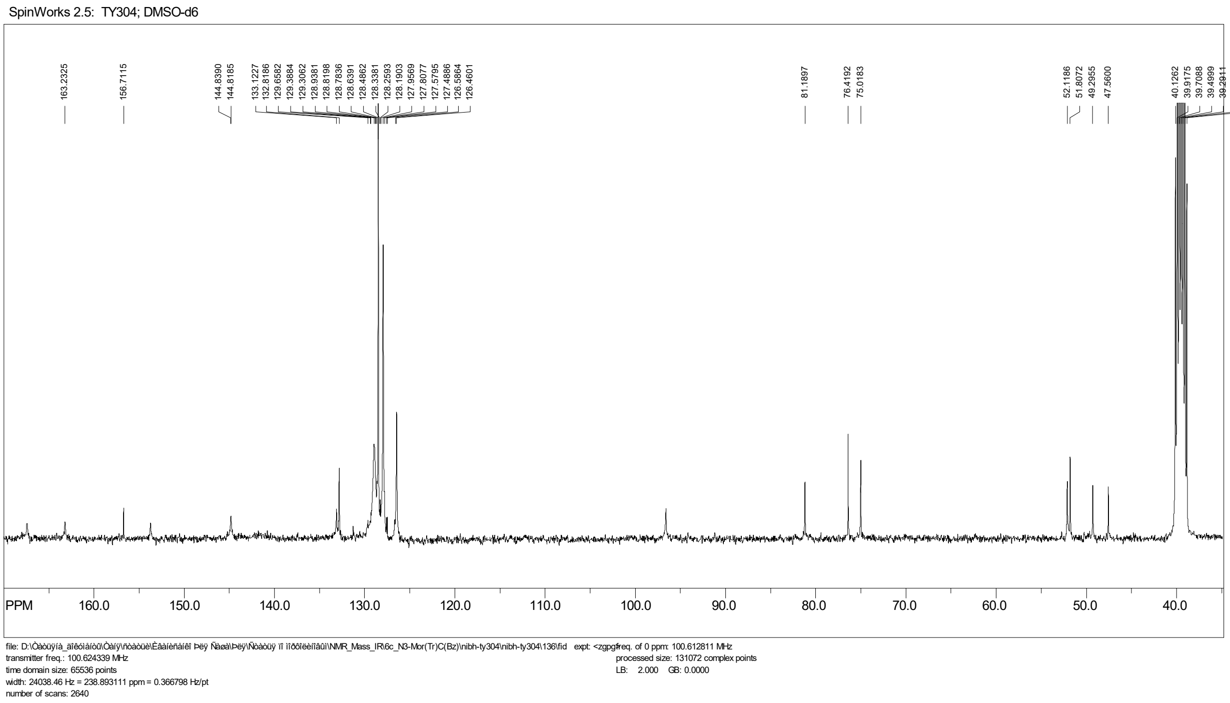
SpinWorks 2.5: TY304; DMSO-d6
163.2325
156.7115
144.8390
144.8185
133.1227
132.8186
129.6582
129.3884
129.3062
128.9381
128.8198
128.7836
128.6391
128.4862
128.3381
128.2593
128.1903
127.9569
127.8077
127.5795
127.4886
126.5864
126.4601
81.1897
76.4192
75.0183
52.1186
51.8072
49.2955
47.5600
40.1262
39.9175
39.7088
39.4999
39.2911
39.0823
38.8738
PPM
160.0
150.0
140.0
130.0
120.0
110.0
100.0
90.0
80.0
70.0
60.0
50.0
40.0
freq. of 0 ppm: 100.612811 MHz
transmitter freq.: 100.624339 MHz
processed size: 131072 complex points
time domain size: 65536 points
LB: 2.000 GB: 0.0000
width: 24038.46 Hz = 238.893111 ppm = 0.366798 Hz/pt
number of scans: 2640

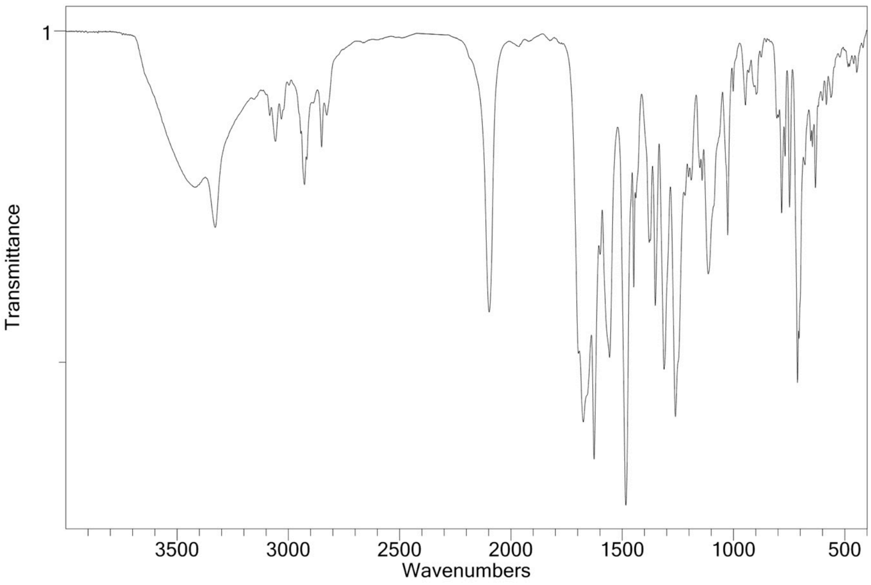
1
Transmittance
3500
3000
2500
2000
1500
1000
500
Wavenumbers

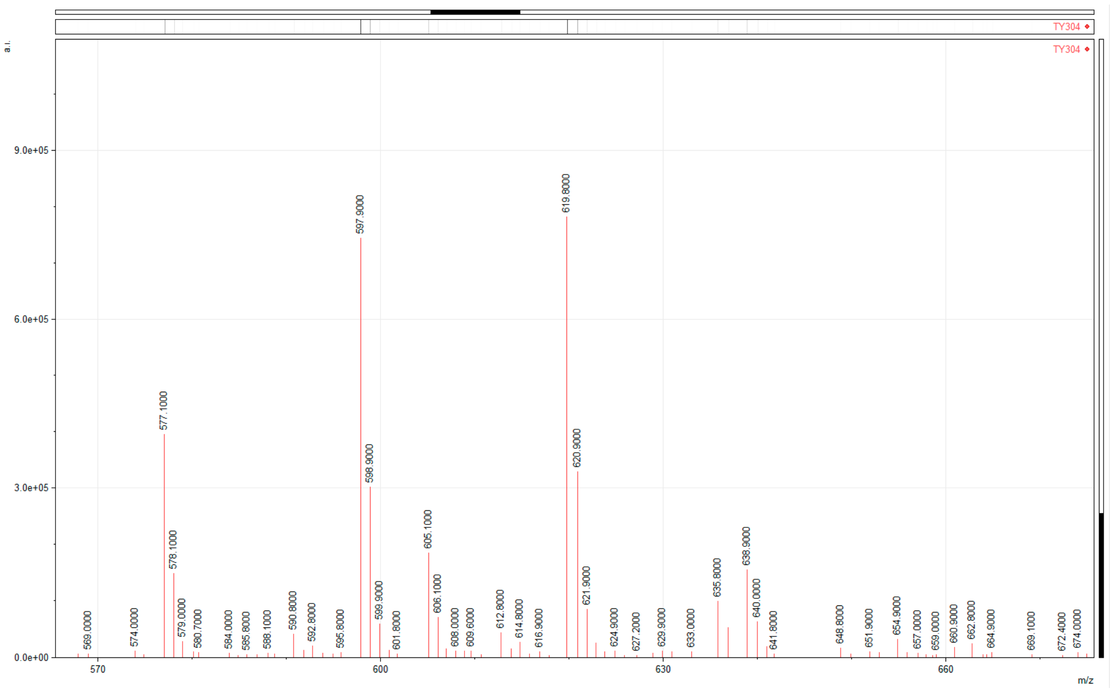

TY304
a.i.
9.0e+05
6.0e+05
3.0e+05
0.0e+00
570
600
630
660
m/z
577.1000
597.9000
619.8000
598.9000
620.9000
605.1000
638.9000
635.8000
578.1000

2’-Azidomethyl-4’-*N*-trityl-6’-(uracil-1-yl)-morpholine (6U)

^{1}H (500 MHz, DMSO-d_6): 11.32 (s, 1H, *NH*-Ura), 7.49-7.37 (m, 6H, Tr), 7.34 (d, *J* 8.1, 1H, H6-Ura), 7.32 (t, *J* 7.6, 6H, Tr), 7.20 (t, *J* 6.8, 3H, Tr), 6.07 (dd, *J* 9.5, 2.4, 1H, *H6’*), 5.52 (d, *J* 8.1, 1H, *H5*-Ura), 4.46-4.39 (m, 1H, *H2’*), 3.41 (dd, *J* 13.0, 3.8, 1H, *CH_2*-N_3), 3.34 (dd, *J* 13.1, 6.2, 1H, *CH_2*-N_3), 3.20 (dt, *J* 11.4, 2.2, 1H, *H5’*), 2.99 (dt, *J* 11.8, 2.2, 1H, *H3’*), 1.36 (br t, *J* 10.8, 2H, *H3’*, *H5’*).

^{13}C (125 MHz, DMSO-d_6): 162.76, 149.69, 142.22, 139.79, 128.81, 127.76, 126.28, 101.70, 79.79, 76.24, 74.73, 51.68, 51.30, 49.11.

IR ν_{max}/cm^{-1} 2100 (N_3)

MS ESI (m/z): $[M-H]^-$ calcd for $C_{28}H_{25}N_6O_3$ 493.19, found 493.3.

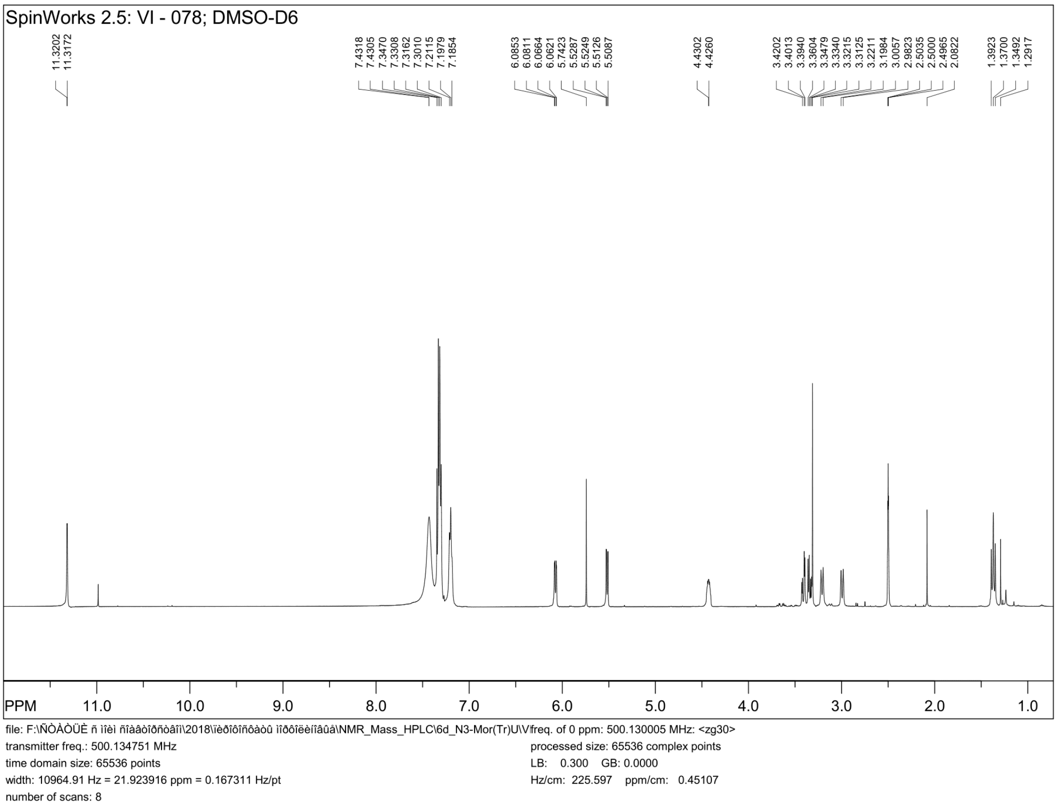

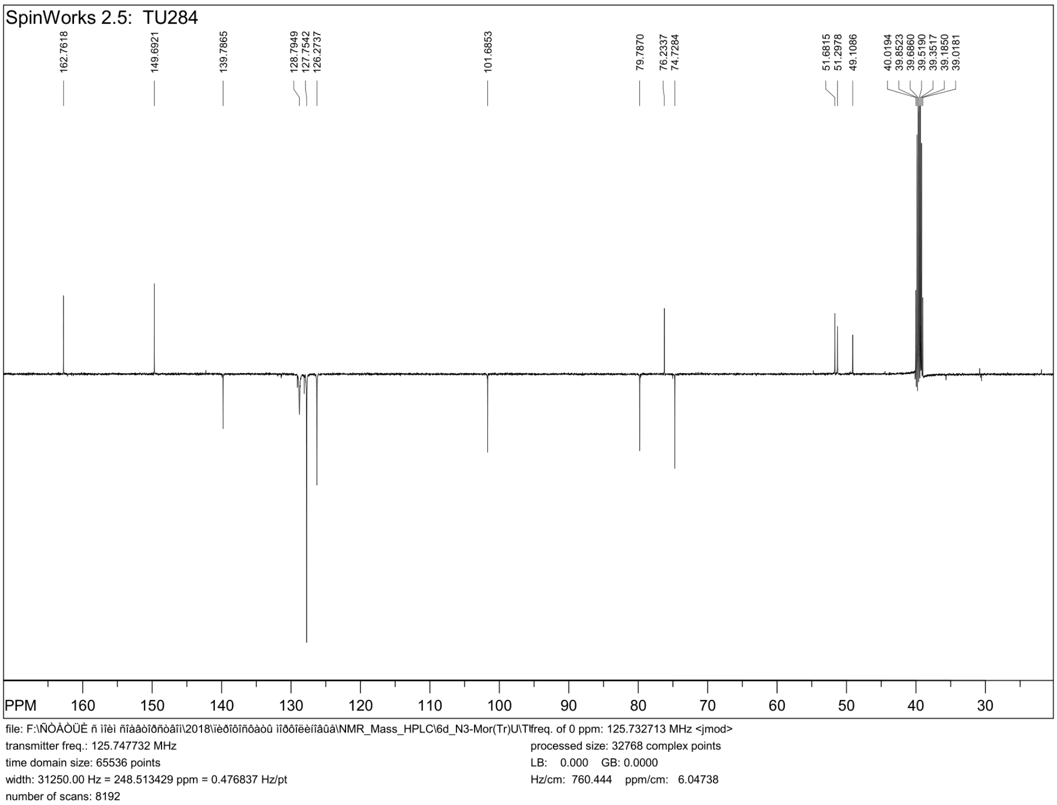
SpinWorks 2.5: TU284
162.7618
149.6921
139.7865
128.7949
127.7542
126.2737
101.6853
79.7870
76.2337
74.7284
51.6815
51.2978
49.1086
40.0194
39.8523
39.6860
39.5190
39.3517
39.1850
39.0181
PPM 160 150 140 130 120 110 100 90 80 70 60 50 40 30
file: F:\ÑÒÀÒÜÈ ñ ìîèì ñîàâòîðñòâîì\2018\ïèðîôîñôàòû ìîðôîëèíîâûå\NMR_Mass_HPLC\6d_N3-Mor(Tr)U\Tl freq. of 0 ppm: 125.732713 MHz <jmod>
transmitter freq.: 125.747732 MHz
time domain size: 65536 points
width: 31250.00 Hz = 248.513429 ppm = 0.476837 Hz/pt
number of scans: 8192
processed size: 32768 complex points
LB: 0.000 GB: 0.0000
Hz/cm: 760.444 ppm/cm: 6.04738

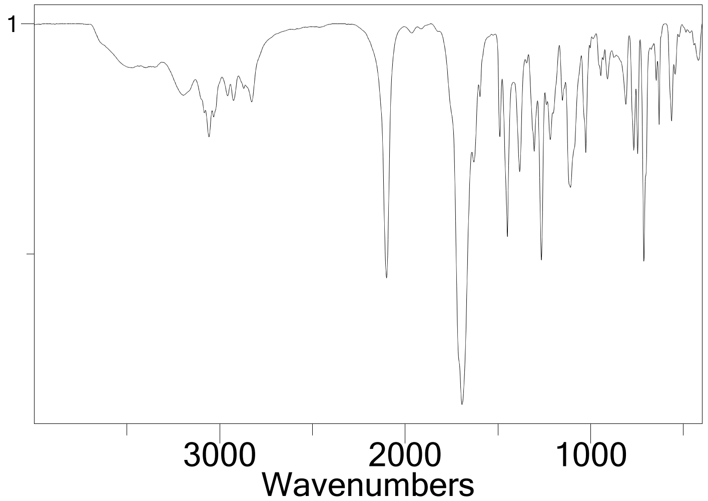
1
3000
2000
1000
Wavenumbers

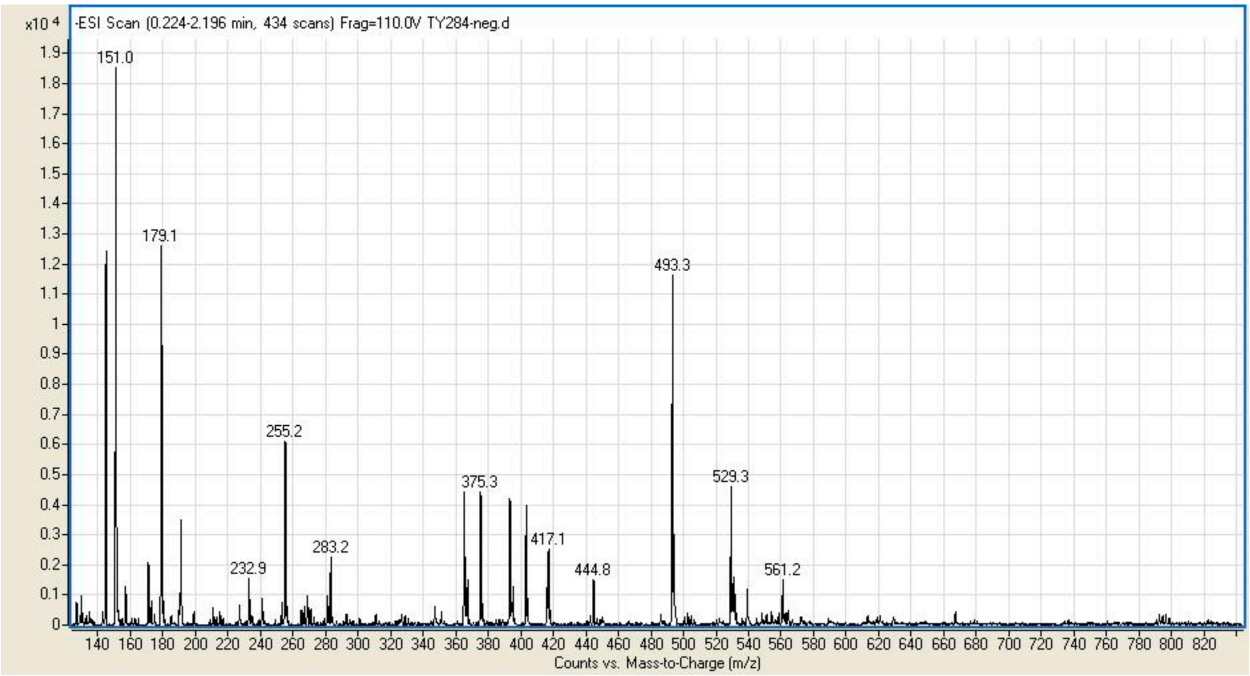

-ESI Scan (0.224-2.196 min, 434 scans) Frag=110.0V TY284-neg.d
x10 4
151.0
179.1
255.2
232.9
283.2
375.3
417.1
444.8
493.3
529.3
561.2
Counts vs. Mass-to-Charge (m/z)

2’-Azidomethyl-4’-*N*-trityl-6’-(thymine-1-yl)-morpholine (6T)

^{1}H (500 MHz, DMSO-d_6): 11.34 (s, 1H, *NH*-Thy), 7.52-7.37 (m, 6H, Tr), 7.31 (br t, *J* 7.2, 6H, Tr), 7.24 (s, *H6*-Thy), 7.19 (br t, *J* 6.5, 3H, Tr), 6.08 (dd, *J* 9.8, 1.6, 1H, *H6’*), 4.47-4.40 (m, 1H, *H2’*), 3.29 (dd, *J* 10.0, 5.2, 1H, *H5’*), 3.43 (dd, *J* 13.0, 3.4, 1H, *CH$_2$*-N$_3$), 3.32 (dd, *J* 12.5, 5.9, 1H, *CH$_2$*-N$_3$), 3.19 (br d, *J* 11.2, 1H, *H5’*), 2.99 (br d, *J* 11.6, 1H, *H3’*), 1.66 (s, 3H, *CH$_3$*-Thy), 1.42 (td, *J* 10.6, 3.7, 2H, *H3’*, *H5’*).

^{13}C (125 MHz, DMSO-d_6): 163.49, 149.73, 143.28, 135.27, 128.83, 127.80, 126.31, 109.20, 79.89, 76.23, 74.77, 51.71, 51.20, 49.14, 11.92.

IR ν_{max}/cm^{-1} 2100 (N$_3$)

MS ESI (m/z): [M-H]$^-$ calcd for $C_{29}H_{28}N_6O_3^-$ 507.2, found 506.9.

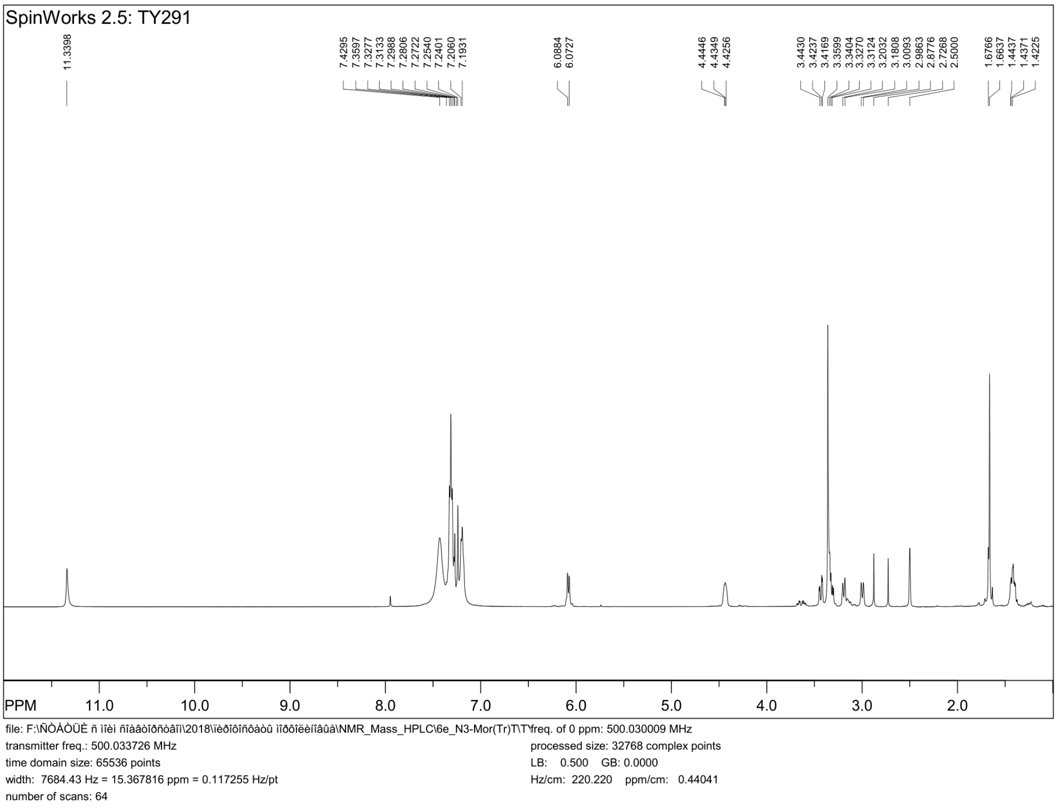

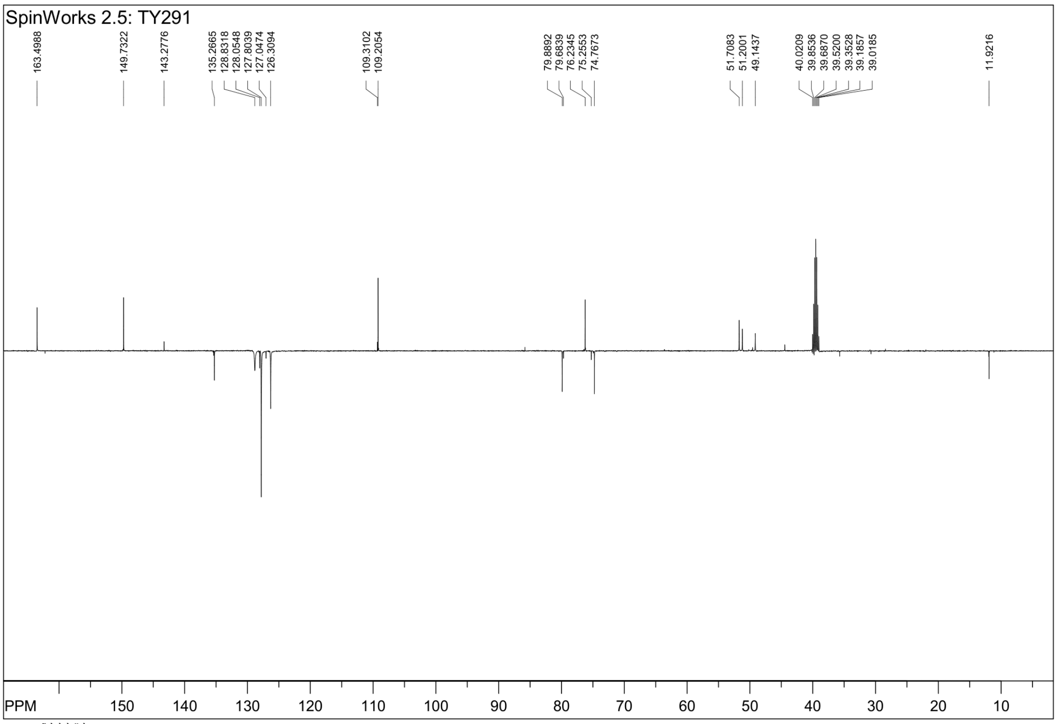

file: F:\ÑÒÀÒÜÈ ñ ìîèì ñîàâòîðñòâîì\2018\ïèðîîîñòàòû ìîðôîëèíîâûå\NMR_Mass_HPLC\6e_N3-Mor(Tr)T\T'freq. of 0 ppm: 125.732709 MHz
transmitter freq.: 125.747732 MHz
time domain size: 65536 points
width: 31250.00 Hz = 248.513429 ppm = 0.476837 Hz/pt
number of scans: 2048
processed size: 32768 complex points
LB: 0.500 GB: 0.0000
Hz/cm: 840.731 ppm/cm: 6.68585

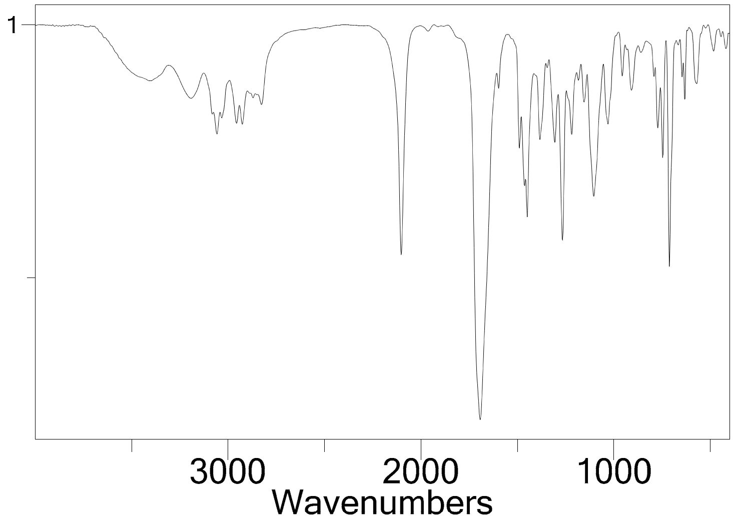

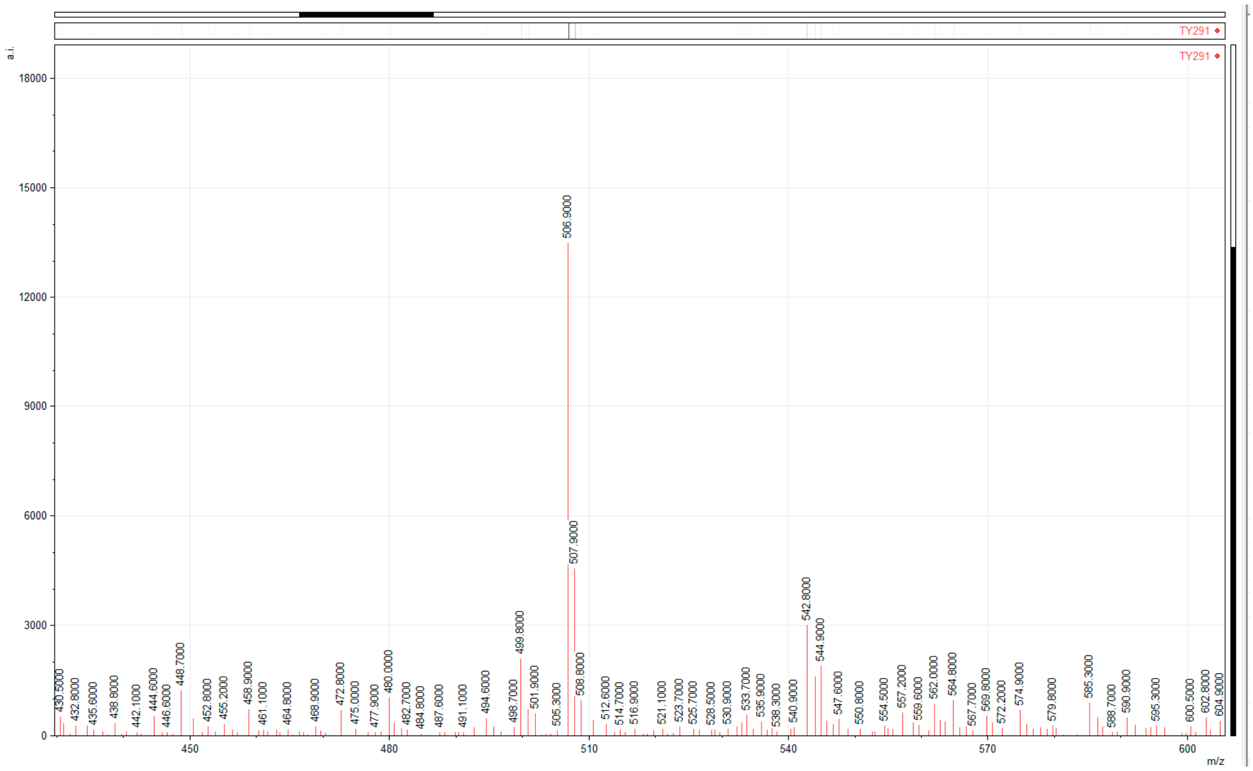

TY291
a.i.
18000
15000
12000
9000
6000
3000
0
506.9000
507.9000
542.8000
544.9000
499.8000
m/z
450
480
510
540
570
600

2'-Azidomethyl-4'-*N*-trityl-6'-(5-iodouracil-1-yl)-morpholine (6IU)

^{1}H (500 MHz, DMSO-d_6): 11.71 (br s, 1H, *NH*-Ura), 7.76 (s, 1H, *H6*-Ura), 7.50-7.16 (m, 15H, Tr), 6.02 (dd, *J* 9.1, 1.6, 1H, *H6'*), 4.49-4.39 (m, 1H, *H4'*), 3.86-3.76 (m, 1H, *H5'*), 3.54-3.42 (m, 2H, CH_2-N_3), 3.24, (br d, *J* 10.9, 1H, *H5'*), 2.96 (br d, *J* 10.9, 1H, *H3'*), 1.50-1.33 (m, 2H, *H3'*, *H5'*).

^{13}C (125 MHz, DMSO-d_6): 160.36, 149.42, 143.90, 129.00, 127.86, 126.36, 80.44, 76.23, 75.02, 69.56, 51.55, 50.41, 48.97.

IR ν_{max}/cm^{-1} 2100 (N_3)

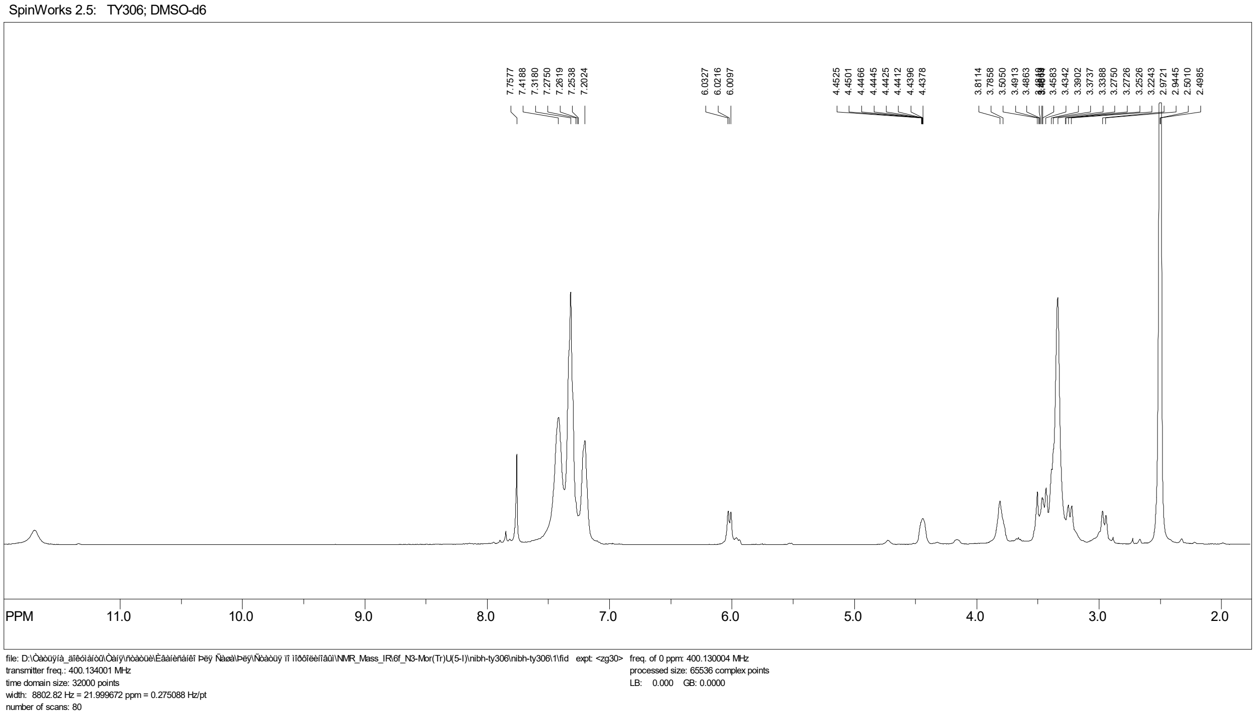

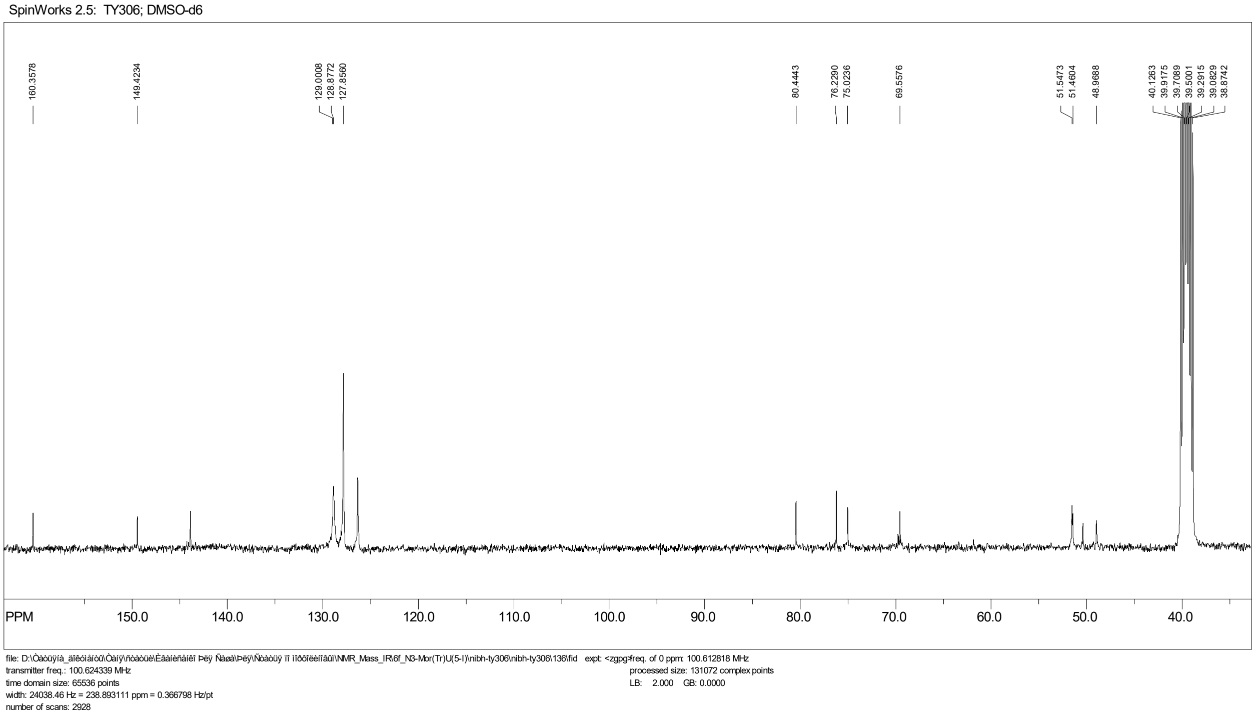

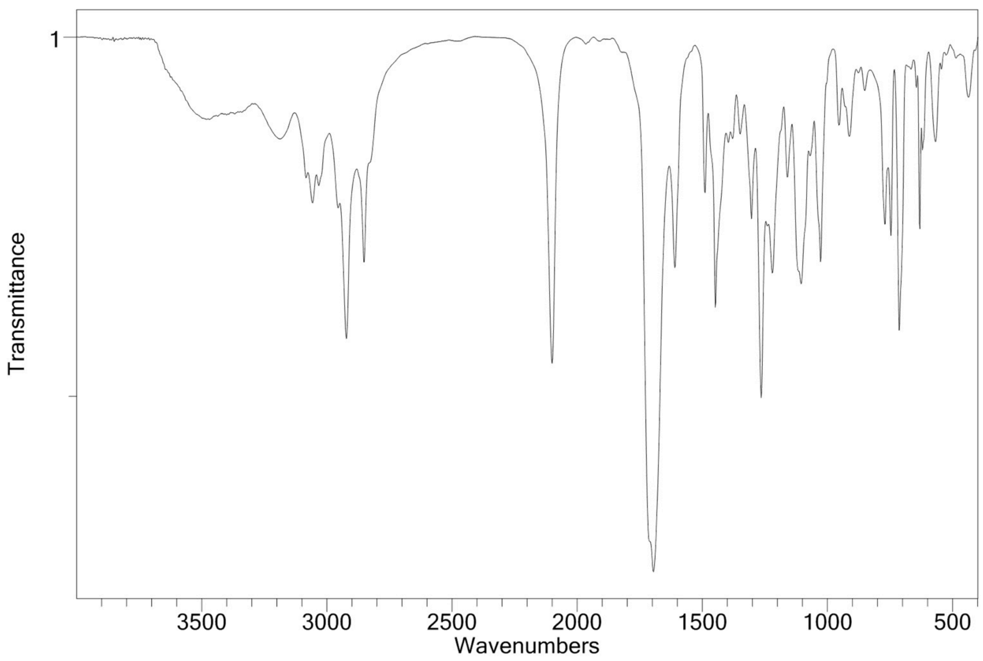
1
Transmittance
3500
3000
2500
2000
1500
1000
500
Wavenumbers

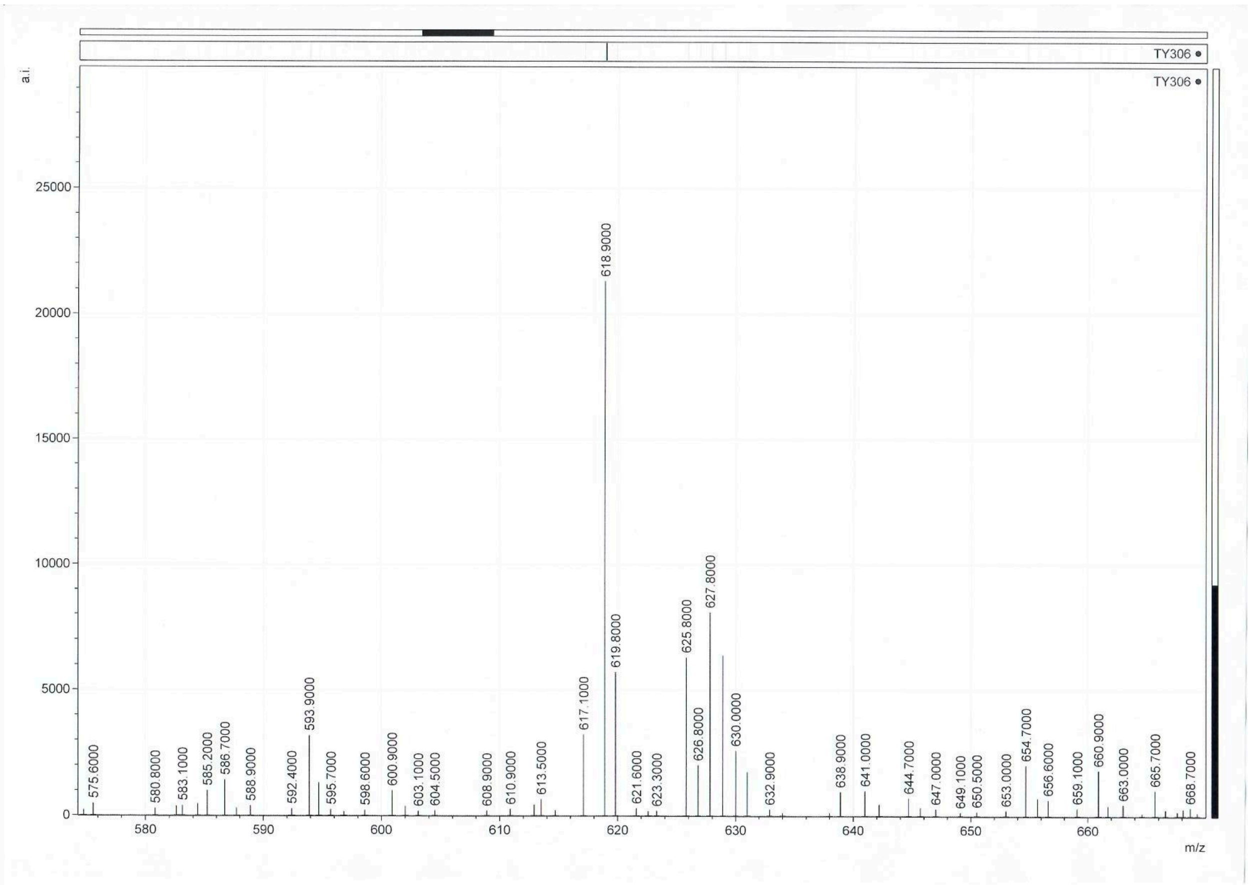
TY306
TY306
a.i.
25000
20000
15000
10000
5000
0
575.6000
580.8000
583.1000
585.2000
586.7000
588.9000
592.4000
593.9000
595.7000
598.6000
600.9000
603.1000
604.5000
608.9000
610.9000
613.5000
617.1000
618.9000
619.8000
621.6000
623.3000
625.8000
626.8000
627.8000
630.0000
632.9000
638.9000
641.0000
644.7000
647.0000
649.1000
650.5000
653.0000
654.7000
656.6000
659.1000
660.9000
663.0000
665.7000
668.7000
580
590
600
610
620
630
640
650
660
m/z

2’-Azidomethyl-4’-*N*-trityl-6’-(5-bromouracil-1-yl)-morpholine (6BrU)

^{1}H (500 MHz, DMSO-d_6): 11.82 (s, 1H, *NH*-Ura), 7.79 (s, 1H, H6-Ura), 7.54-7.37 (m, 6H, Tr), 7.32 (t, *J* 7.2, 6H, Tr), 7.20 (t, *J* 6.1, 3H, Tr), 6.05 (dd, *J* 9.3, 1.8, 1H, *H6’*), 4.49-4.40 (m, 1H, *H2’*), 3.42 (dd, *J* 13.1, 3.8, 1H, *CH*$_2$-N$_3$), 3.37 (dd, *J* 13.3, 6.2, 1H, *CH*$_2$-N$_3$), 3.26 (dt, *J* 11.3, 2.3, 1H, *H5’*), 2.97 (dt, *J* 11.6, 2.2, 1H, *H3’*), 1.47-1.36 (m, 2H, *H3’*, *H5’*).

^{13}C (125 MHz, DMSO-d_6): 158.89, 149.03, 139.31, 128.81, 127.75, 126.26, 95.77, 80.44, 76.20, 74.94, 51.56, 51.34, 48.97.

IR ν_{max}/cm^{-1} 2100 (N$_3$)

MS MALDI-TOF (*m/z*): [M-H]$^-$ calcd for $C_{28}H_{24}BrN_6O_3^-$ 571.110; found 571.366.

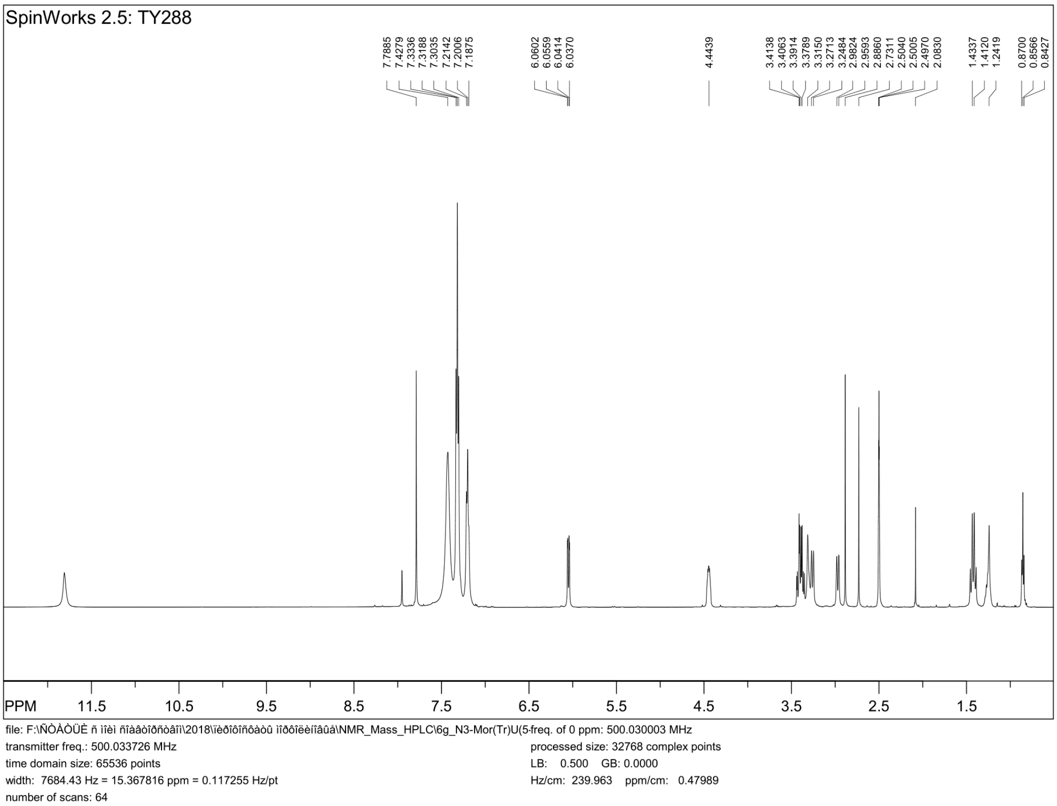

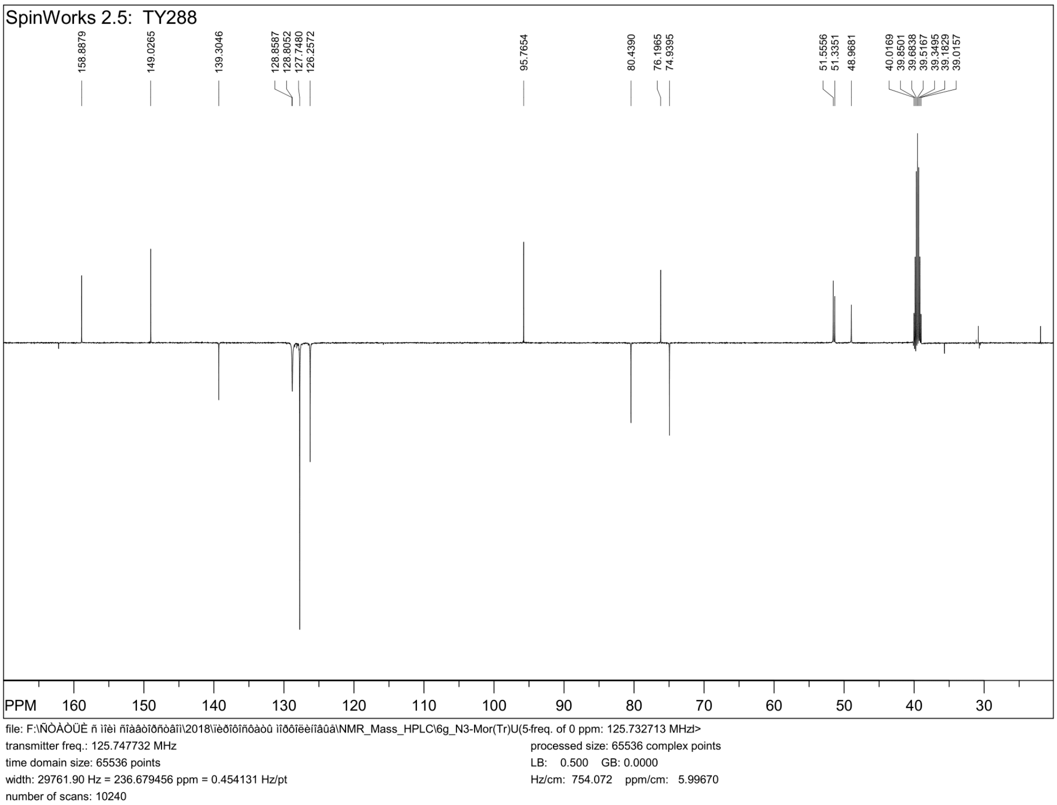
SpinWorks 2.5: TY288
158.8879
149.0265
139.3046
128.8587
128.8052
127.7480
126.2572
95.7654
80.4390
76.1965
74.9395
51.5556
51.3351
48.9681
40.0169
39.8501
39.6838
39.5167
39.3495
39.1829
39.0157
PPM 160 150 140 130 120 110 100 90 80 70 60 50 40 30
file: F:\ÑÒÀÒÜÈ ñ ìîèì ñîàâòîðñòâîì\2018\ïèðîõîñôàòû ïîðôèðèíîâûå\NMR_Mass_HPLC\6g_N3-Mor(Tr)U(5-freq. of 0 ppm: 125.732713 MHz|>
transmitter freq.: 125.747732 MHz
time domain size: 65536 points
width: 29761.90 Hz = 236.679456 ppm = 0.454131 Hz/pt
number of scans: 10240
processed size: 65536 complex points
LB: 0.500 GB: 0.0000
Hz/cm: 754.072 ppm/cm: 5.99670

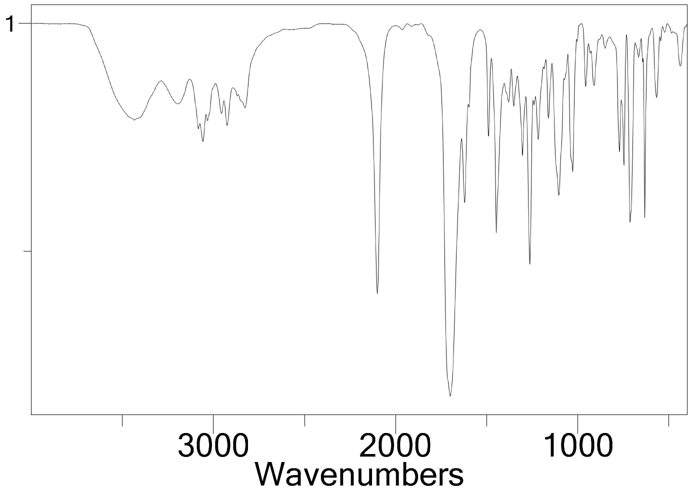
1
3000
2000
1000
Wavenumbers

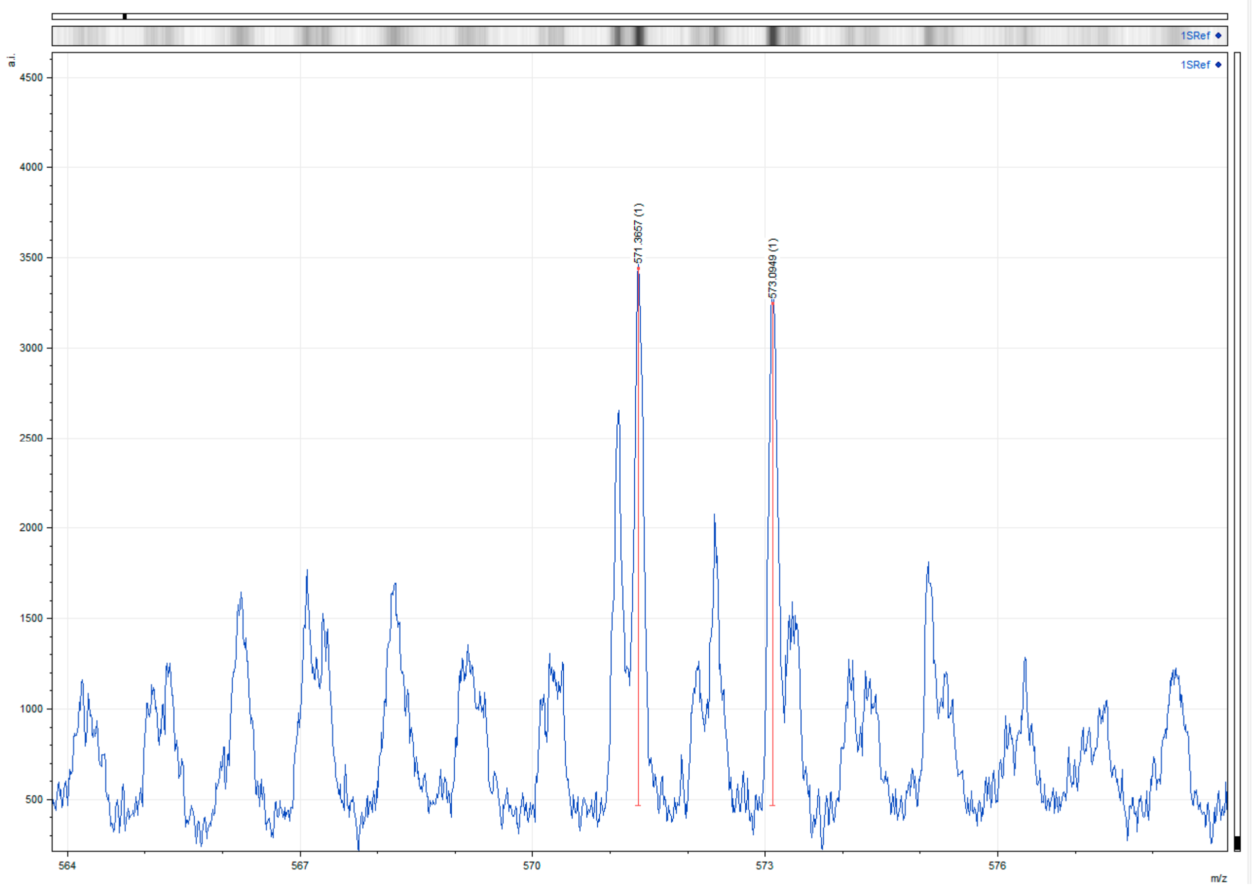
1SRef
1SRef
a.i.
4500
4000
3500
3000
2500
2000
1500
1000
500
571.3657 (1)
573.0949 (1)
564
567
570
573
576
m/z

2’-Azidomethyl-4’-*N*-trityl-6’-(5-chlorouracil-1-yl)-morpholine (6ClU)

^{1}H (500 MHz, DMSO-d_6): 11.84 (s, 1H, *NH*-Ura), 7.74 (s, 1H, H6-Ura), 7.51-7.37 (m, 6H, Tr), 7.32 (t, *J* 7.6, 6H, Tr), 7.20 (t, *J* 6.7, 3H, Tr), 6.04 (dd, *J* 9.6, 2.4, 1H, *H6’*), 4.48-4.40 (m, 1H, *H2’*), 3.42 (dd, *J* 13.0, 3.8, 1H, *CH_2*-N_3), 3.38 (dd, *J* 13.0, 6.2, 1H, *CH_2*-N_3), 3.25 (dt, *J* 11.5, 2.1, 1H, *H5’*), 2.97 (dt, *J* 11.7, 2.2, 1H, *H3’*), 1.43 (dd, *J* 11.3, 5.2, 1H, *H3’*), 1.40 (dd, *J* 11.2, 3.9, 1H, *H5’*).

^{13}C (125 MHz, DMSO-d_6): 158.74, 148.79, 137.04, 128.81, 127.76, 126.27, 107.19, 80.36, 76.19, 74.90, 51.57, 51.28, 48.98.

IR ν_{max}/cm^{-1} 2100 (N_3)

MS MALDI-TOF (*m/z*): [M-H]$^-$ calcd for $C_{28}H_{24}ClN_6O_3^-$ 527.160; found 527.122.

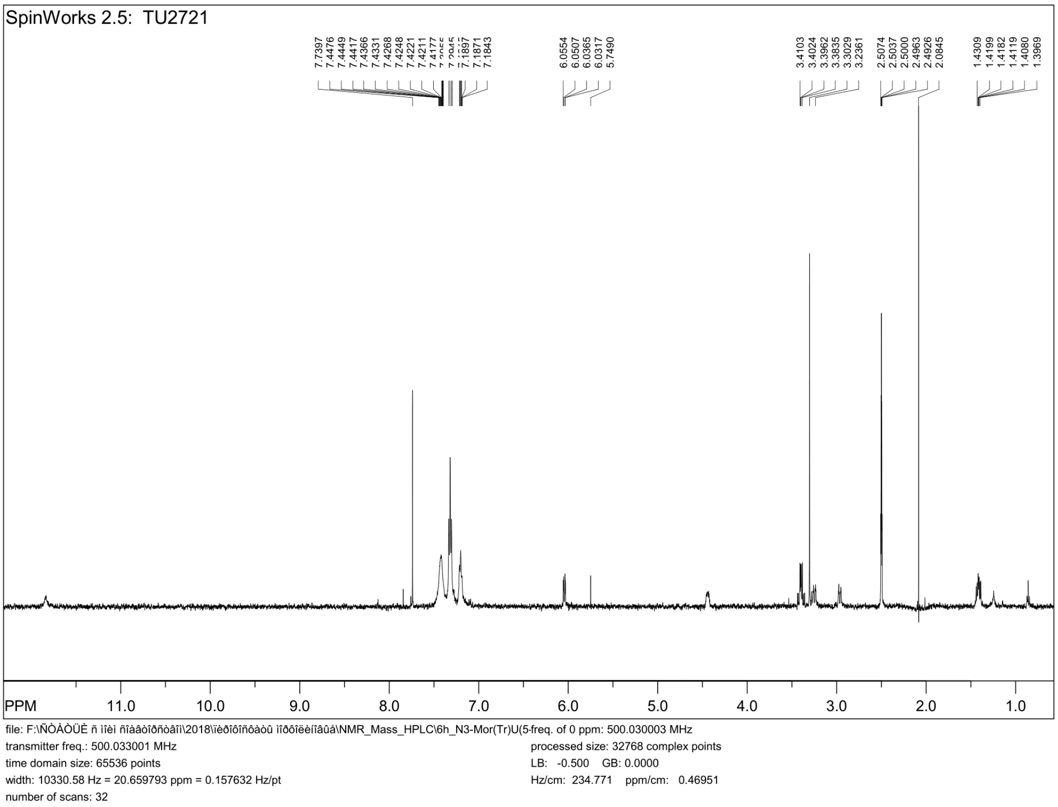

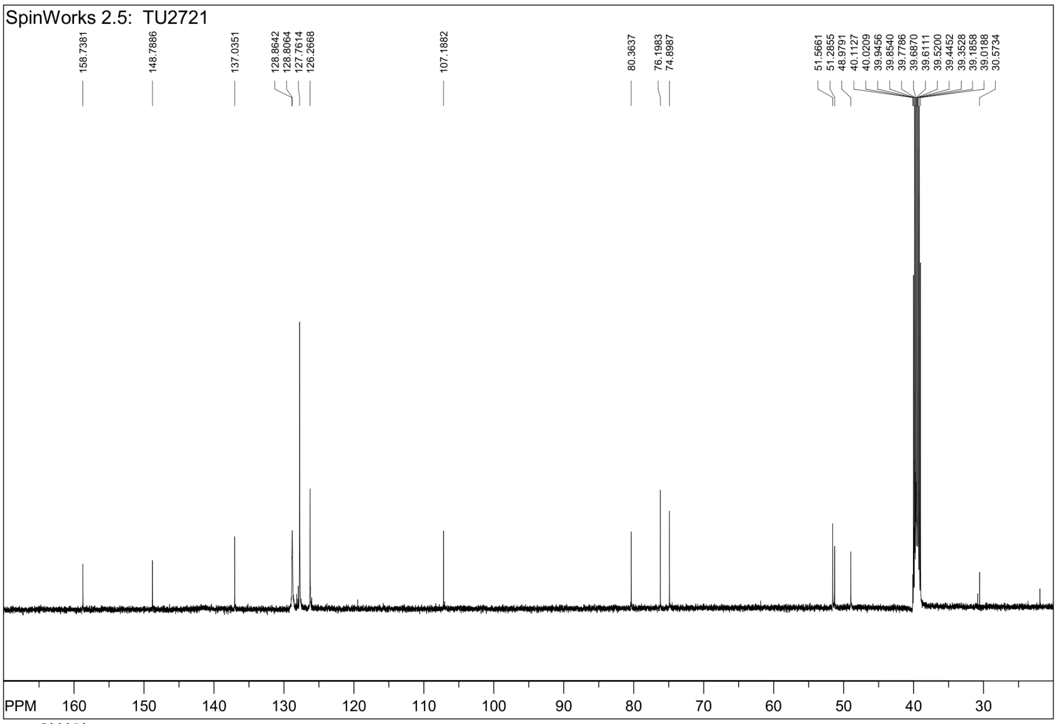

file: F:\ÑÒÀÒÜÈ ñ ìîèì ñîàâòîðñòâîì\2018\ïèðîôîñôàòû ìîðôîëèíîâûå\NMR_Mass_HPLC\6h_N3-Mor(Tr)U(5-freq. of 0 ppm: 125.732712 MHzɒg>
transmitter freq.: 125.747735 MHz
time domain size: 65536 points
width: 29761.90 Hz = 236.679450 ppm = 0.454131 Hz/pt
number of scans: 4096

processed size: 32768 complex points
LB: -0.500 GB: 0.0000
Hz/cm: 754.694 ppm/cm: 6.00165

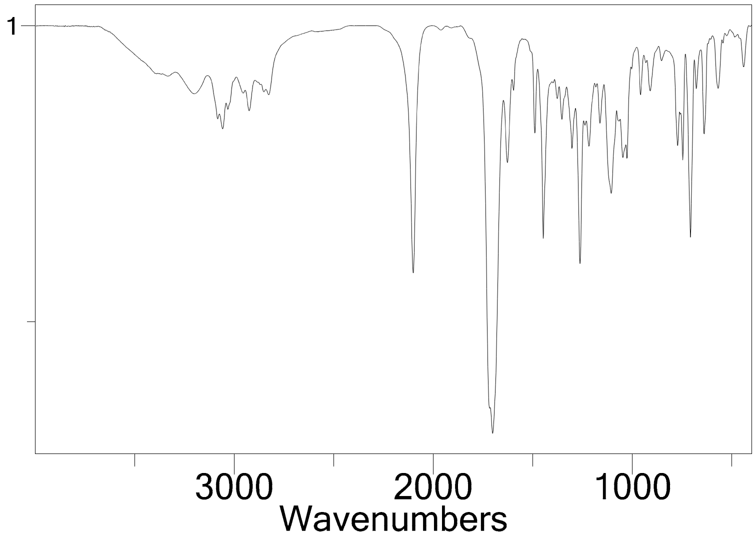

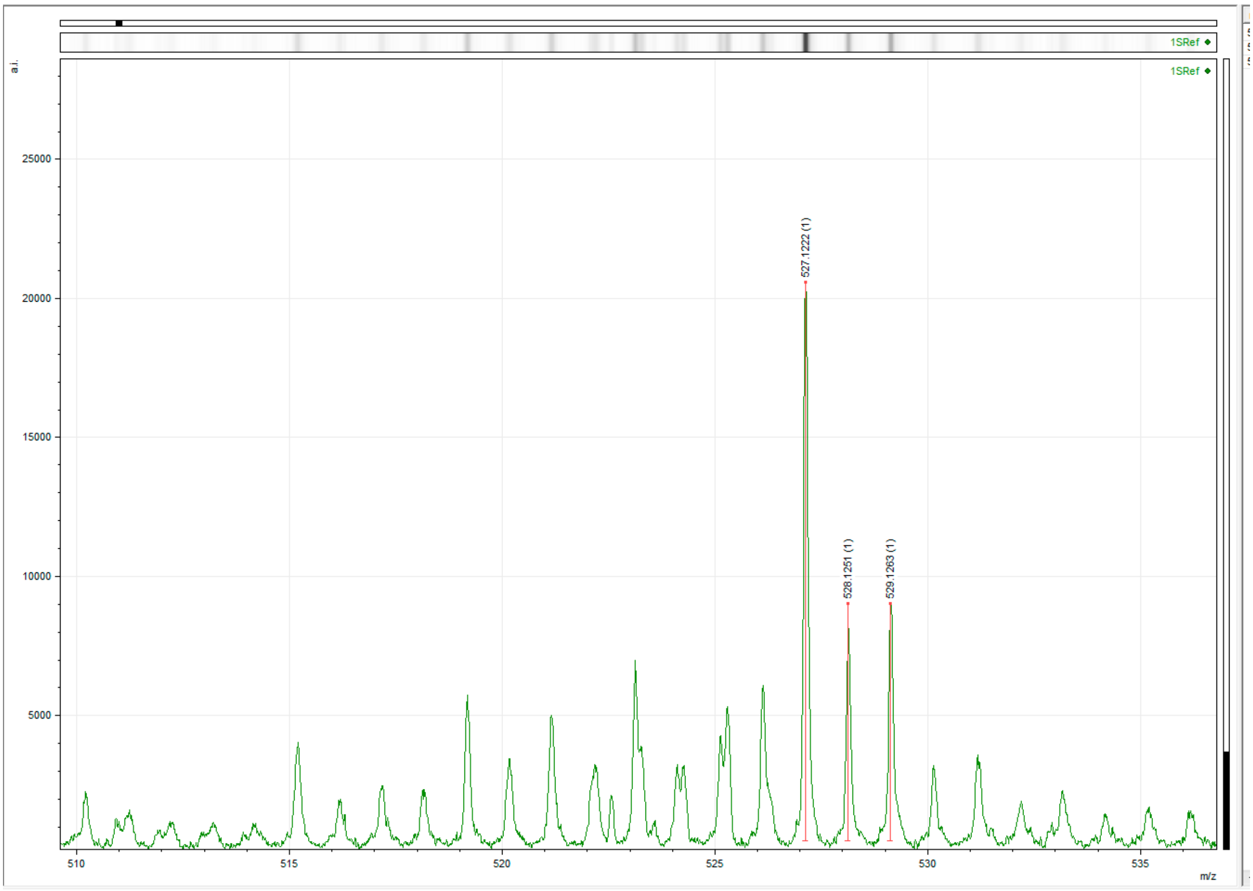
1SRef
a.i.
25000
20000
15000
10000
5000
527.1222 (1)
528.1251 (1)
529.1263 (1)
510
515
520
525
530
535
m/z

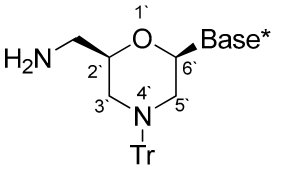

7A,G,C,U,T,IU,BrU,ClU

2'-Aminomethyl-4'-*N*-trityl-6'-(N^6-benzoyladenine-9-yl)-morpholine (7A)

^{1}H (500 MHz, DMSO-d$_6$): 8.74 (s, 1H, *H8*-Ade), 8.48 (s, 1H, *H2*-Ade), 8.03 (d, *J* 7.3, 2H, *o*-Bz), 7.63-7.43 (m, 9H, *p*-Bz, *m*-Bz, Tr), 7.33 (br t, *J* 7.3, 6H, Tr), 7.20 (br t, *J* 6.4, 3H, Tr), 6.41 (dd, *J* 9.7, 1.8, 1H, *H6'*), 4.31-4.22 (m, 1H, *H2'*), 3.39 (br d, *J* 11.3, 1H, *H5'*), 3.17 (br d, *J* 11.9, 1H, *H3'*), 2.67-2.63 (m, 2H, *CH$_2$*-NH$_2$), 2.05 (t, *J* 10.6, 1H, *H3'*), 1.47 (t, *J* 11.0, 1H, *H5'*).

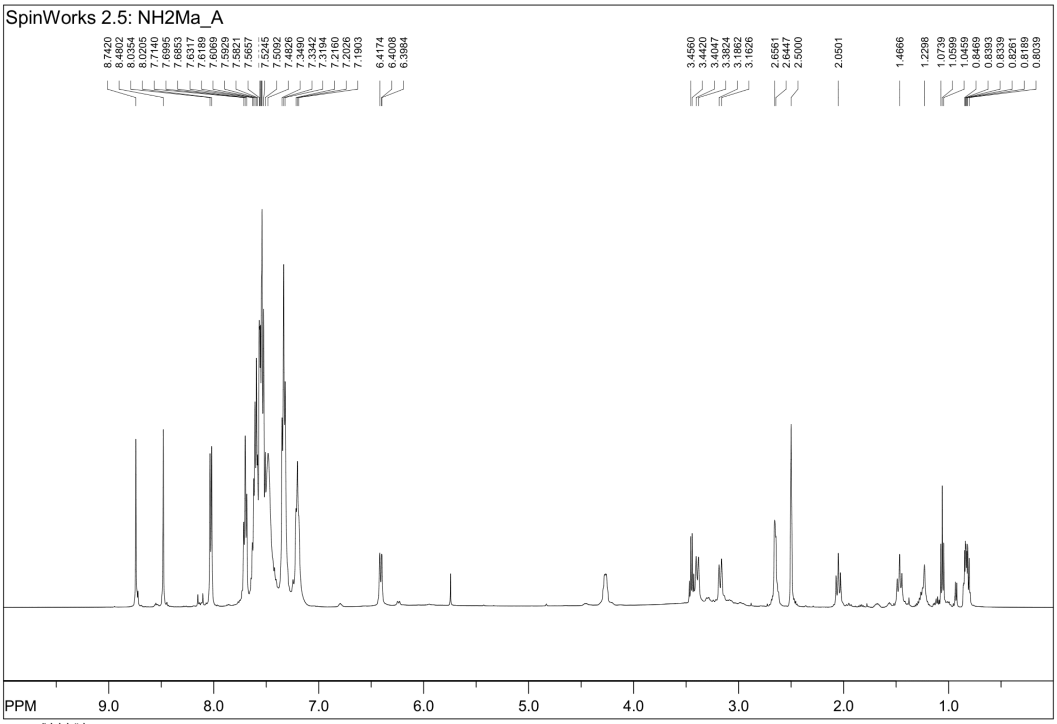

file: F:\ÑÒÀÒÜÈ ñ ìîèì ñîàâòîðñòâîì\2018\ïèðîîîñôàòû ìîðôîëèíîâûå\NMR_Mass_HPLC\7a_NH2-Mor(Tr)A(freq. of 0 ppm: 500.030009 MHz
transmitter freq.: 500.033001 MHz | processed size: 32768 complex points
time domain size: 65536 points | LB: 0.500 GB: 0.0000
width: 10330.58 Hz = 20.659793 ppm = 0.157632 Hz/pt | Hz/cm: 200.030 ppm/cm: 0.40003
number of scans: 32

2'-Aminomethyl-4'-*N*-trityl-6'-(N^2-isobutyrylguanine-9-yl)-morpholine (7G)

^{1}H (500 MHz, DMSO-d_6): 7.96 (s, 1H, *H8*-Gua), 7.45-7.39 (m, 6H, Tr), 7.35 (br t, *J* 7.3, 6H, Tr), 7.22 (br t, *J* 7.1, 3H, Tr), 6.08 (dd, *J* 9.8, 1.8, 1H, *H6'*), 4.50-4.43 (m, 1H, *H2'*), 3.37-3.23 (m, 3H, *H5'*, *CH*$_2$-NH$_2$), 3.17 (br d, *J* 11.9, 1H, *H3'*), 2.89-2.80 (m, 1H, *CH*-iBu), 11.88 (t, *J* 10.8, 1H, *H3'*), 1.46 (t, *J* 11.1, 1H, *H5'*), 1.18-1.14 (m, 6H, *CH*$_3$-iBu).

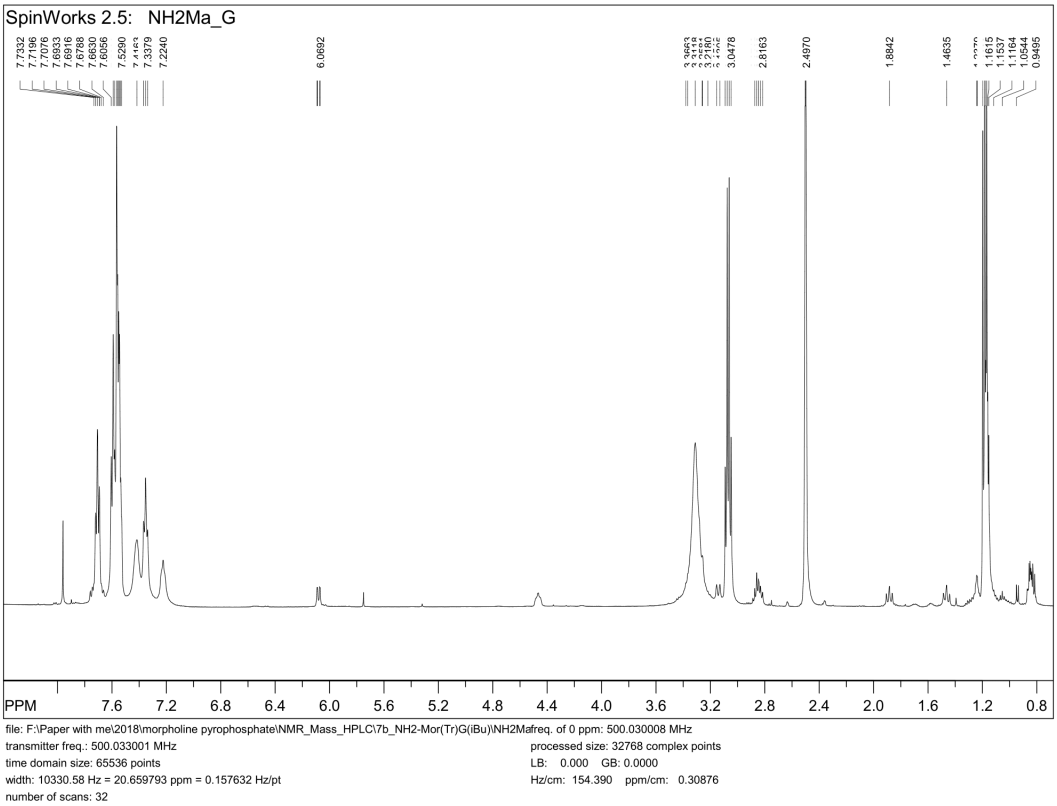

2'-Aminomethyl-4'-*N*-trityl-6'-(*N*[4]-benzoylcytosine-1-yl)-morpholine (7C)

^{1}H (500 MHz, DMSO-d$_6$): 8.01 (d, *J* 7.0, 2H, *H5,H6*-Cyt), 7.56-7.09 (m, 20H, *Tr,Bz*), 6.52 (app d, 1H, *NH*-Cyt), 6.06 (app d, *J* 8.7, 1H, *H6'*), 4.06-3.95 (m, 1H, *H2'*), 3.16 (br d, *J* 10.5, 1H, *H5'*), 3.07 (br d, *J* 11.2, 1H, *H3'*), 2.60-2.51 (m, 2H, *CH$_2$*-NH$_2$), 1.32-1.02 m, 3H, *H3',H5*).*'*

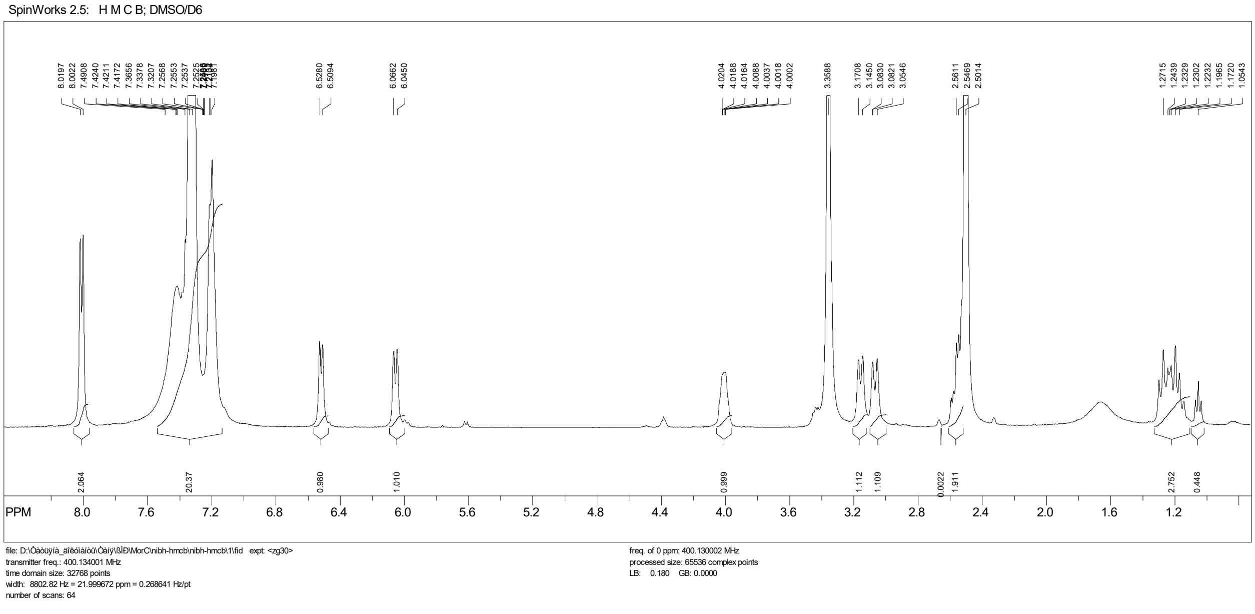

2'-Aminomethyl-4'-*N*-trityl-6'-(uracil-1-yl)-morpholine (7U)

^{1}H (500 MHz, DMSO-d$_6$): 7.46-7.37 (m, 7H, Tr, H6-Ura), 7.31 (t, *J* 7.3, 6H, Tr), 7.19 (t, *J* 6.2, 3H, Tr), 5.98 (dd, *J* 9.5, 1.5, 1H, *H6'*), 5.48 (d, *J* 8.0, 1H, *H5*-Ura), 4.12-4.06 (m, 1H, *H2'*), 3.18 (br d, *J* 11.0, 1H, *H5'*), 3.06 (br d, *J* 11.8, 1H, *H3'*), 2.59-2.55 (m, 2H, *CH$_2$*-NH$_2$), 1.32 (t, *J* 9,5, 1H, *H3'*), 1.30 (t, *J* 10.2, 1H, *H5'*).

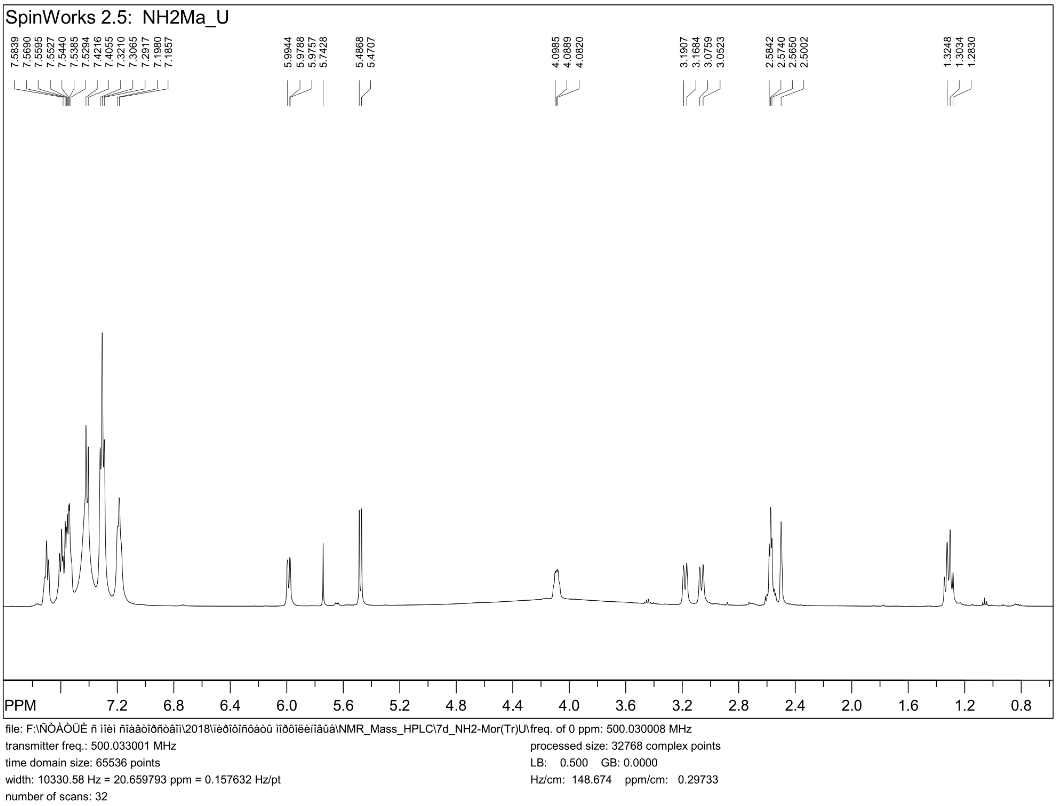

2’-Aminomethyl-4’-*N*-trityl-6’-(thymine-1-yl)-morpholine (7T)

^{1}H (500 MHz, DMSO-d_6): 7.47-7.38 (m, 7H, Tr), 7.37 (s, *H6*-Thy), 7.31 (t, *J* 7.2, 6H, Tr), 7.19 (t, *J* 5.5, 3H, Tr), 6.01 (dd, *J* 9.3, 1,5, 1H, *H6’*), 5.48 (d, *J* 8.0, 1H, *H5*-Ura), 4.26-4.19 (m, 1H, *H2’*), 3.15 (br d, *J* 11.1, 1H, *H5’*), 3.06 (br d, *J* 11.8, 1H, *H3’*), 2.77 (dd, *J* 13.4, 3.8, 1H, CH_2-NH_2), 2.70 (dd, *J* 13.3, 6.4, 1H, CH_2-NH_2), 1.69 (s, 3H, CH_3-Thy), 1.42-1.34 (m, 2H, *H3’*, *H5’*).

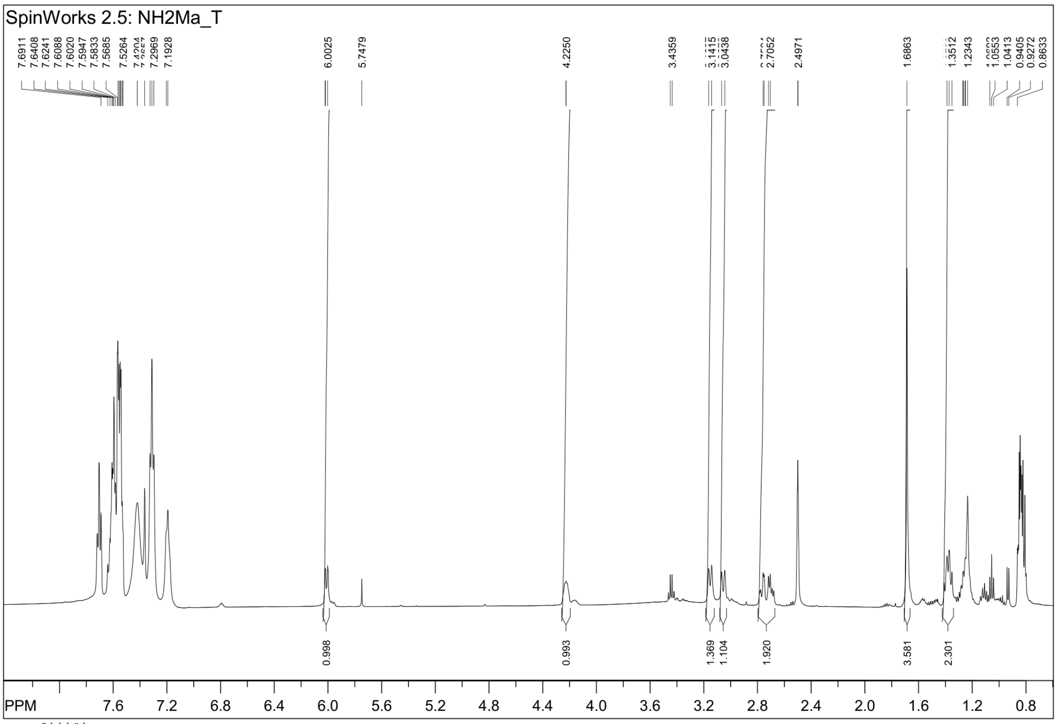

file: F:\ÑÒÀÒÜÈ ñ ïîèì ñîàâòîðñòâî\2018\ìèðîîîñôàòû ìîðôîëèíîâûå\NMR_Mass_HPLC\7e_NH2-Mor(Tr)T\lfreq. of 0 ppm: 500.030008 MHz
transmitter freq.: 500.033001 MHz
time domain size: 65536 points
width: 10330.58 Hz = 20.659793 ppm = 0.157632 Hz/pt
number of scans: 32
processed size: 32768 complex points
LB: 0.500 GB: 0.0000
Hz/cm: 156.442 ppm/cm: 0.31286

2’-Aminomethyl-4’-*N*-trityl-6’-(5-iodouracil-1-yl)-morpholine (7IU)

^{1}H (500 MHz, DMSO-d$_6$): 7.82 (s, 1H, *H6*-Ura) 7.76-7.37 (m, 6H, Tr,), 7.31 (t, *J* 7.3, 6H, Tr), 7.19 (t, *J* 6.6, 3H, Tr), 5.95 (dd, *J* 9.6, 1.8, 1H, *H6’*), 4.15-4.07 (m, 1H, *H2’*), 3.19 (br d, *J* 11.1, 1H, *H5’*), 3.03 (br d, *J* 11.7, 1H, *H3’*), 2.66-2.61 (m, 2H, *CH$_2$*-NH$_2$), 1.37 (t, *J* 10.9, 1H, *H3’*), 1.36 (t, *J* 10.1, 1H, *H5’*).

^{13}C (125 MHz, DMSO-d$_6$): 160.42, 149.56, 144.20, 128.87, 127.75, 126.24, 80.49, 77.34, 76.18, 69.56, 51.61, 49.55, 43.37.

MS ESI (*m/z*): [M-H]$^-$ calcd for $C_{28}H_{26}IN_4O_3^-$ 593.11; found 593.29.

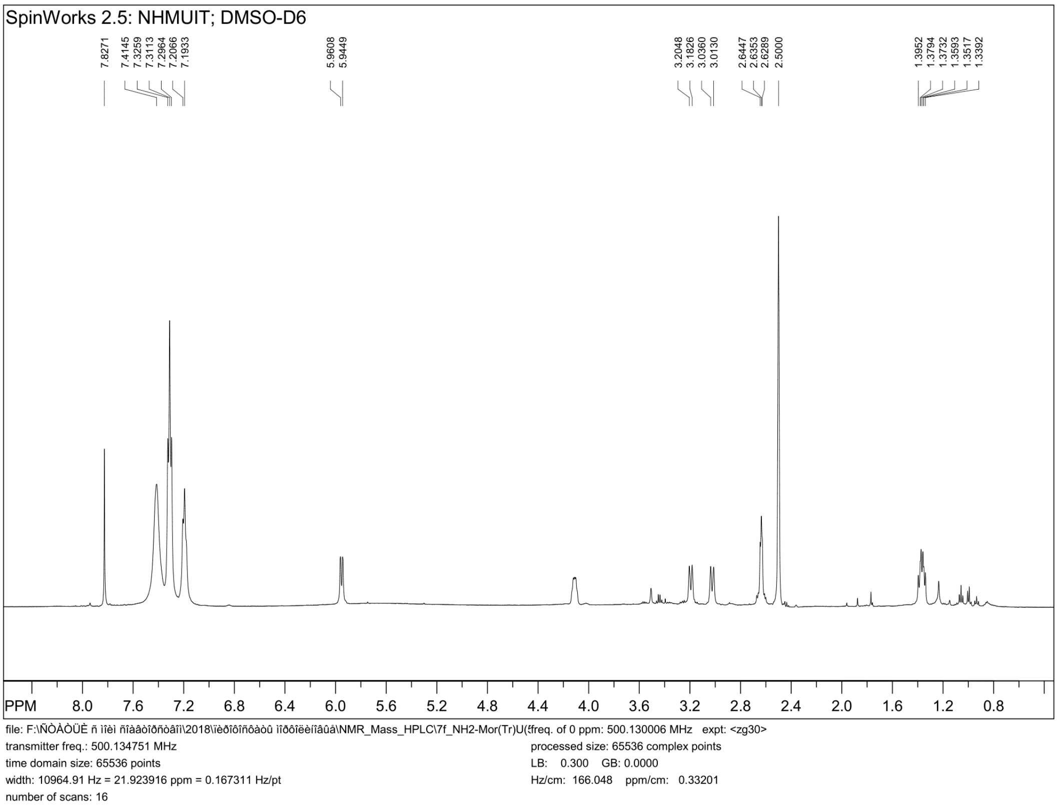

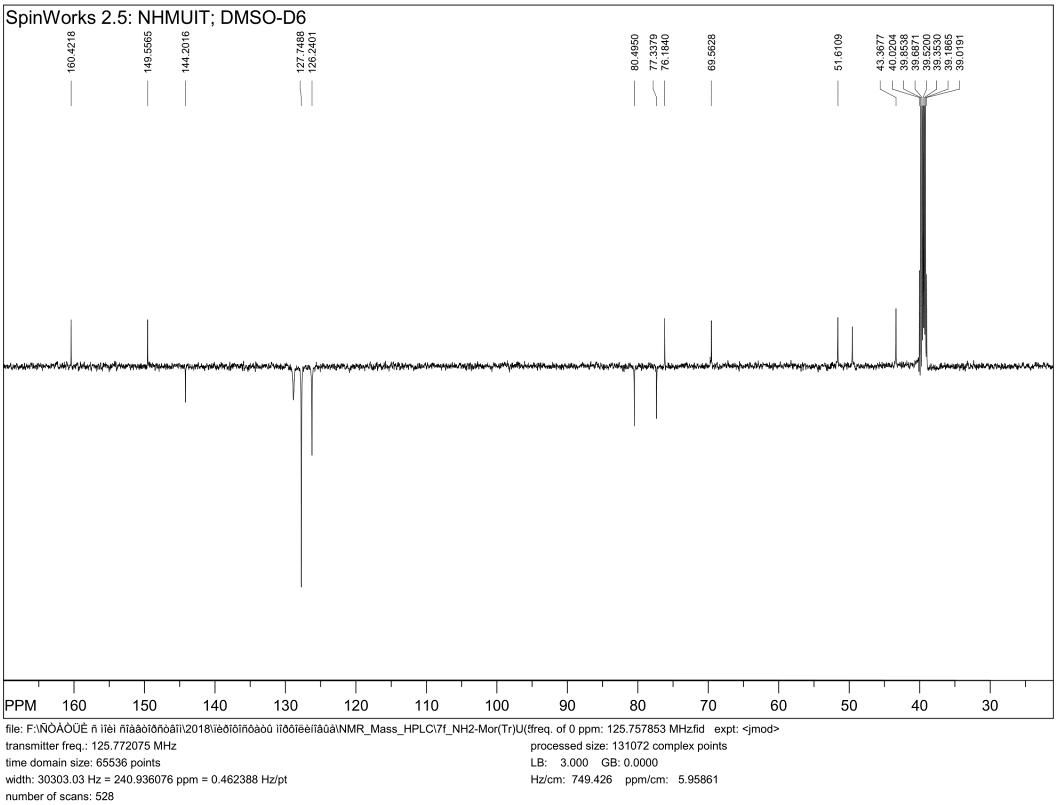
SpinWorks 2.5: NHMUIT; DMSO-D6
160.4218
149.5565
144.2016
127.7488
126.2401
80.4950
77.3379
76.1840
69.5628
51.6109
43.3677
40.0204
39.8538
39.6871
39.5200
39.3530
39.1865
39.0191
PPM 160 150 140 130 120 110 100 90 80 70 60 50 40 30
file: F:\ÑÒÀÒÜÈ ñ ìîèì ñîàâòîðñòâîì\2018\ìèðîíîñòàòû ìîðôîëèíîâûå\NMR_Mass_HPLC\7f_NH2-Mor(Tr)U(5freq. of 0 ppm: 125.757853 MHz.fid expt: <jmod>
transmitter freq.: 125.772075 MHz
time domain size: 65536 points
width: 30303.03 Hz = 240.936076 ppm = 0.462388 Hz/pt
number of scans: 528
processed size: 131072 complex points
LB: 3.000 GB: 0.0000
Hz/cm: 749.426 ppm/cm: 5.95861

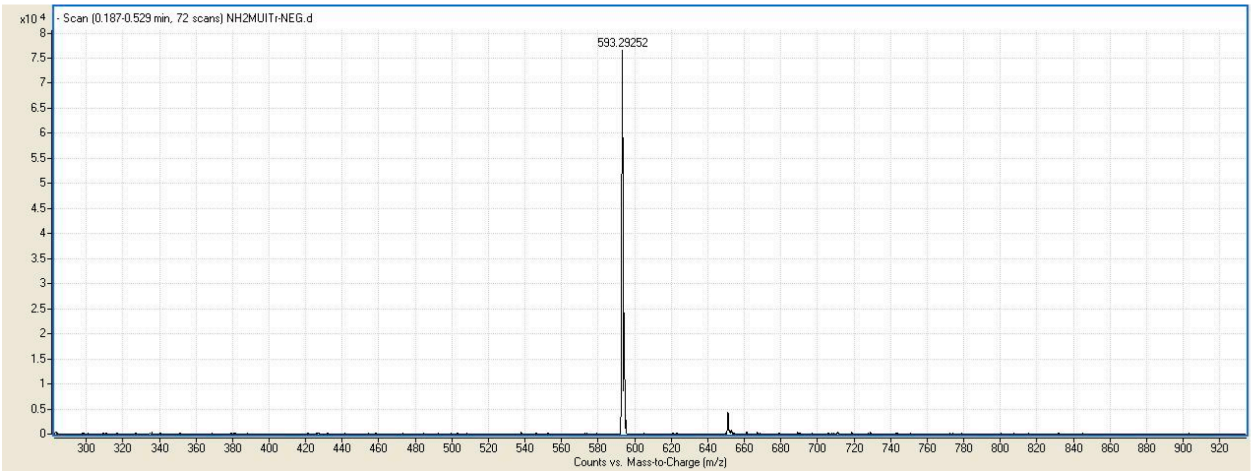
x10 4
- Scan (0.187-0.529 min, 72 scans) NH2MUITr-NEG.d
593.29252
Counts vs. Mass-to-Charge (m/z)

2’-Aminomethyl-4’-*N*-trityl-6’-(5-bromouracil-1-yl)-morpholine (7BrU)

^{1}H (400 MHz, $CDCl_3$+DMSO-d_6): 7.28 (s, 1H, *H6*-Ura) 7.10-6.96 (m, 6H, Tr,), 6.88 (t, *J* 7.4, 6H, Tr), 6.77 (t, *J* 6.8, 3H, Tr), 5.65 (d, *J* 8.5, 1H, *H6’*), 3.90-3.78 (m, 1H, *H2’*), 2.93 (br d, *J* 11.2, 1H, *H5’*), 2.79 (br d, *J* 11.4, 1H, *H3’*), 2.20 (br s, 2H, *CH_2*-NH_2), 1.03 (t, *J* 10.6, 1H, *H3’*), 0.94 (t, *J* 11.1, 1H, *H5’*)

^{13}C (125 MHz, $CDCl_3$+DMSO-d_6): 158.46, 148.33, 138.32, 128.12, 126.89, 125.50, 95.51, 79.98, 77.10, 75.66, 51.04, 48.82, 39.94.

MS ESI (*m/z*): $[M-H]^-$ calcd for $C_{28}H_{26}BrN_4O_3^-$ 545.12; found 545.30.

SpinWorks 2.5: NMTUB; CDCl3 + DMSO-d6

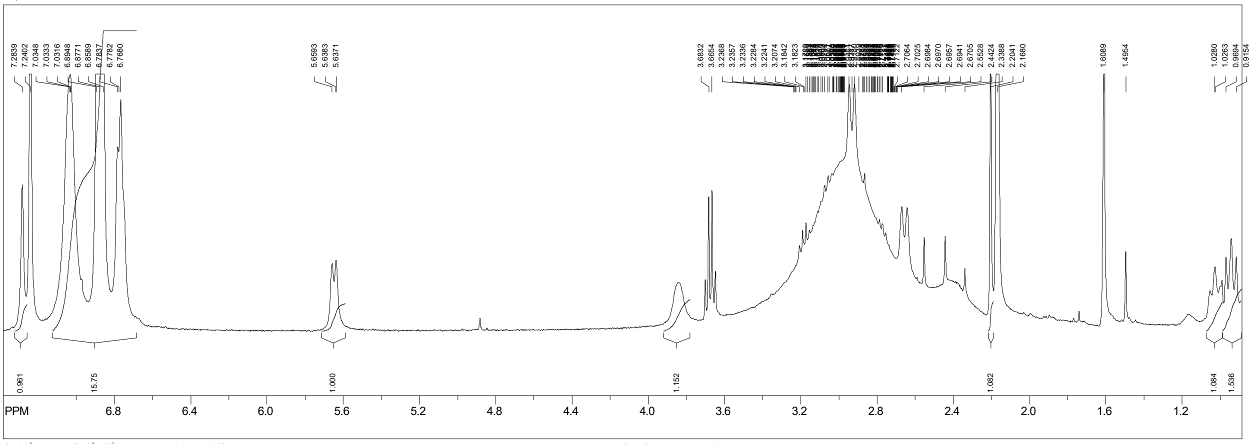

file: D:\Òàòüÿíà_äîêóìåíòû\Òàíÿ\ßÌÐ\Yulya\nibh-nmtub\nibh-nmtub\1\fid expt: <zg30>
transmitter freq.: 400.134001 MHz
time domain size: 32000 points
width: 8802.82 Hz = 21.999672 ppm = 0.275088 Hz/pt
number of scans: 32

freq. of 0 ppm: 400.130018 MHz
processed size: 65536 complex points
LB: 0.300 GB: 0.0000

SpinWorks 2.5: NMTUB 13; CDCL3

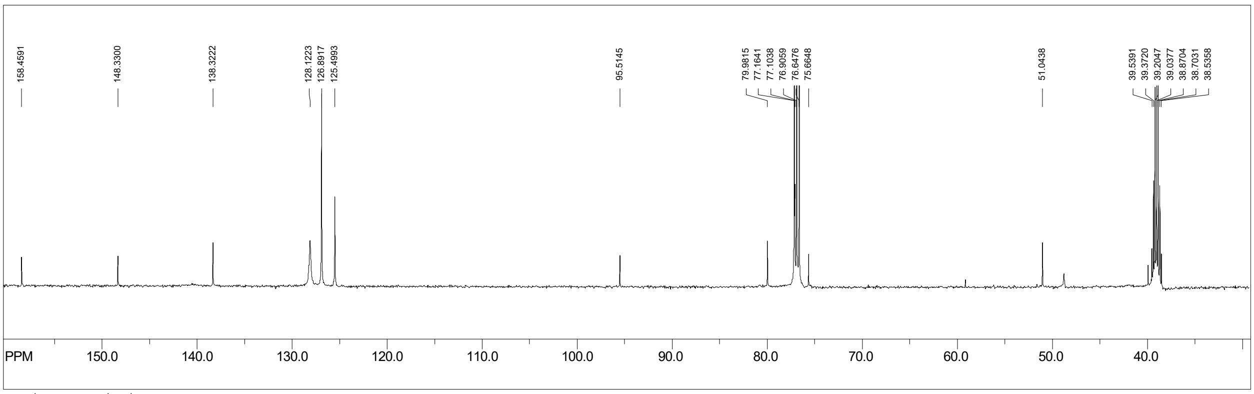

file: D:\Òàòüÿíà_äîêóìåíòû\Òàíÿ\ßÌÐ\Yulya\nibh-nmtub13\nibh-nmtub13\136\fid expt: <zgpg>
transmitter freq.: 125.772075 MHz
time domain size: 65536 points
width: 30303.03 Hz = 240.936076 ppm = 0.462388 Hz/pt
number of scans: 2432

freq. of 0 ppm: 125.757883 MHz
processed size: 131072 complex points
LB: 2.000 GB: 0.0000

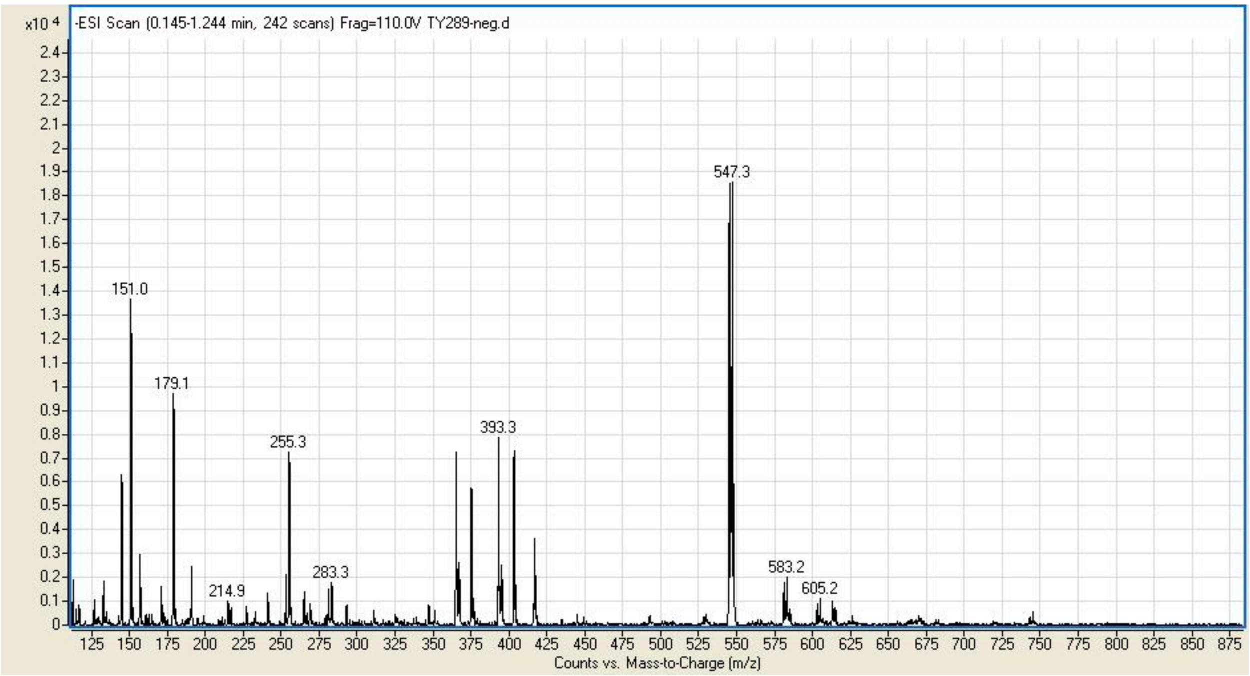

x10 4
-ESI Scan (0.145-1.244 min, 242 scans) Frag=110.0V TY289-neg.d
547.3
151.0
179.1
393.3
255.3
283.3
214.9
583.2
605.2
Counts vs. Mass-to-Charge (m/z)

2’-Aminomethyl-4’-*N*-trityl-6’-(5-chlorouracil-1-yl)-morpholine (7ClU)

^{1}H (400 MHz, $CDCl_3$+DMSO-d_6): 7.10 (s, 1H, *H6*-Ura) 7.22-7.05 (m, 6H, Tr,), 6.96 (t, *J* 7.2, 6H, Tr), 6.85 (t, *J* 7.1, 3H, Tr), 5.74 (dd, *J* 9.5, 2.0, 1H, *H6’*), 3.82-3.75 (m, 1H, *H2’*), 3.02 (dt, *J* 11.2, 2.2, 1H, *H5’*), 2.73 (dt, *J* 11.8, 2.6, 1H, *H3’*), 2.39 (br d, *J* 5.6, 2H, *CH_2*-NH_2), 1.05 (t, *J* 11.3, 1H, *H3’*), 1.01 (t, *J* 10.4, 1H, *H5’*)

^{13}C (125 MHz, $CDCl_3$+DMSO-d_6): 158.90, 148.57, 135.74, 128.45, 127.18, 125.79, 107.86, 80.20, 79.15, 76.01, 51.56, 49.40, 43.75.

MS ESI (*m/z*): [M-H+Na]$^+$ calcd for $C_{28}H_{26}ClN_4NaO_3^-$ 524.16; found 524.02.

SpinWorks 2.5: NMTUC; CDCl3 + DMSO-d6

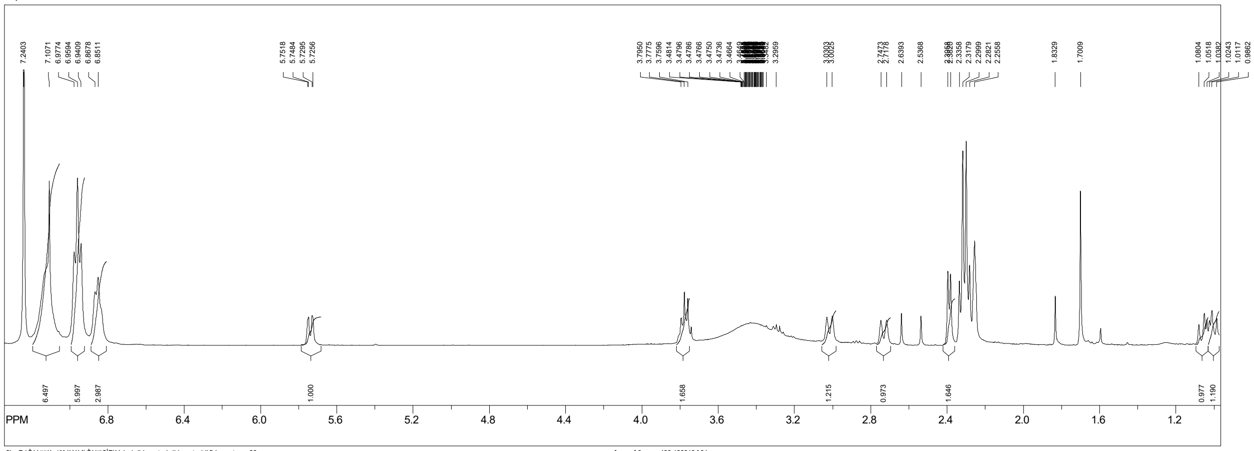

file: D:\Òàòüÿíà_äîêóìåíòû\Òàíÿ\ßÌÐ\Yulya\nibh-nmtuc\nibh-nmtuc\1\fid expt: <zg30>
transmitter freq.: 400.134001 MHz
time domain size: 32000 points
width: 8802.82 Hz = 21.999672 ppm = 0.275088 Hz/pt
number of scans: 32

freq. of 0 ppm: 400.130018 MHz
processed size: 65536 complex points
LB: 0.300 GB: 0.0000

SpinWorks 2.5: NMTUC 13; CDCL3

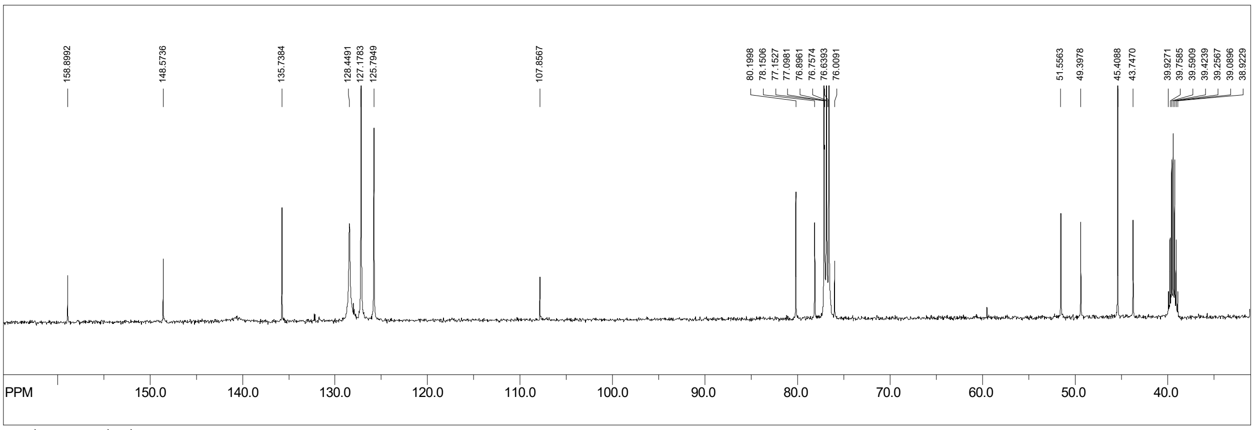

file: D:\Òàòüÿíà_äîêóìåíòû\Òàíÿ\ßÌÐ\Yulya\nibh-nmtuc13\nibh-nmtuc13\136\fid expt: <zgpg>
transmitter freq.: 125.772075 MHz
time domain size: 65536 points
width: 30303.03 Hz = 240.936076 ppm = 0.462388 Hz/pt
number of scans: 2384

freq. of 0 ppm: 125.757856 MHz
processed size: 131072 complex points
LB: 2.000 GB: 0.0000

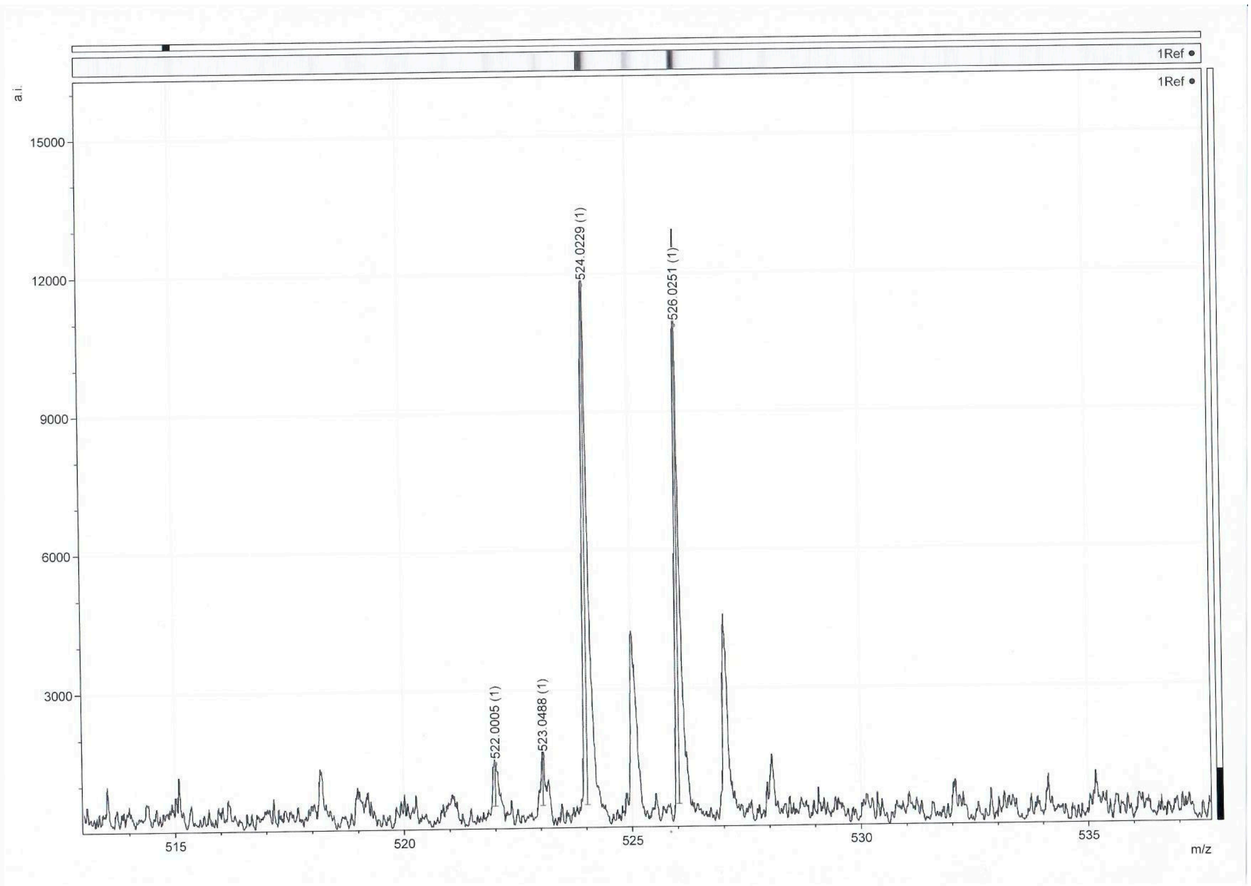
1Ref
1Ref
a.i.
15000
12000
9000
6000
3000
524.0229 (1)
526.0251 (1)
522.0005 (1)
523.0488 (1)
515
520
525
530
535
m/z

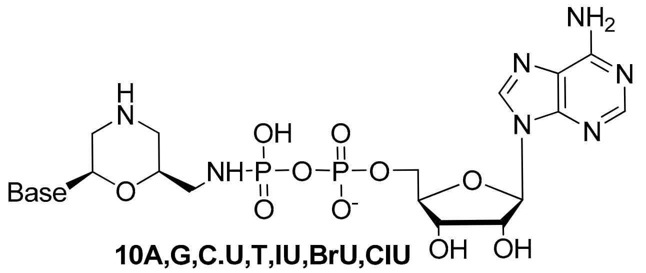

Adenosine-5'-*O*-{β-[6'-(adenine-9-yl)-morpholine-2'-*NH*-methyl]pyrophosphate} (10A)

MS ESI (*m/z*): $[M-H]^-$ calcd for $C_{20}H_{27}N_{12}O_{10}P_2^-$ 657.15; found 656.99.

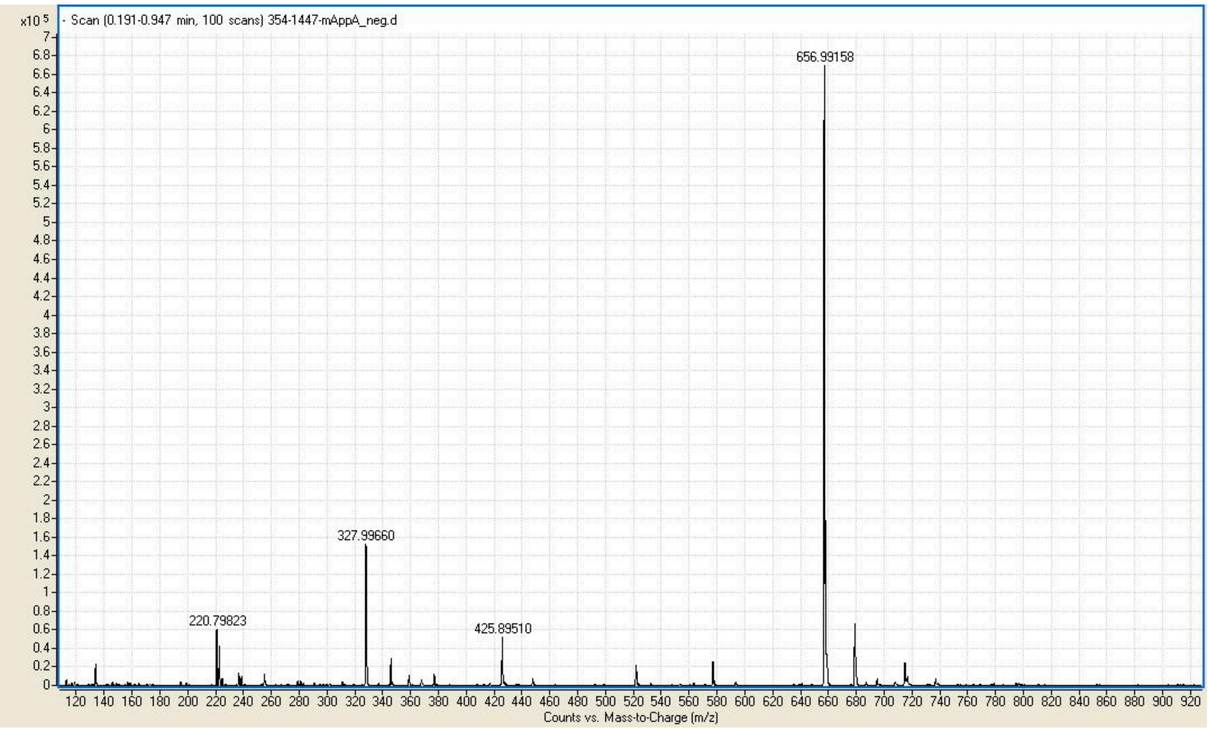

Adenosine-5'-O-{β-[6'-(guanine-9-yl)-morpholine-2'-*NH*-methyl]pyrophosphate} (10G)

MS ESI (*m/z*): $[M-H]^-$ calcd for $C_{20}H_{27}N_{12}O_{11}P_2^-$ 673.140; found 673.434.

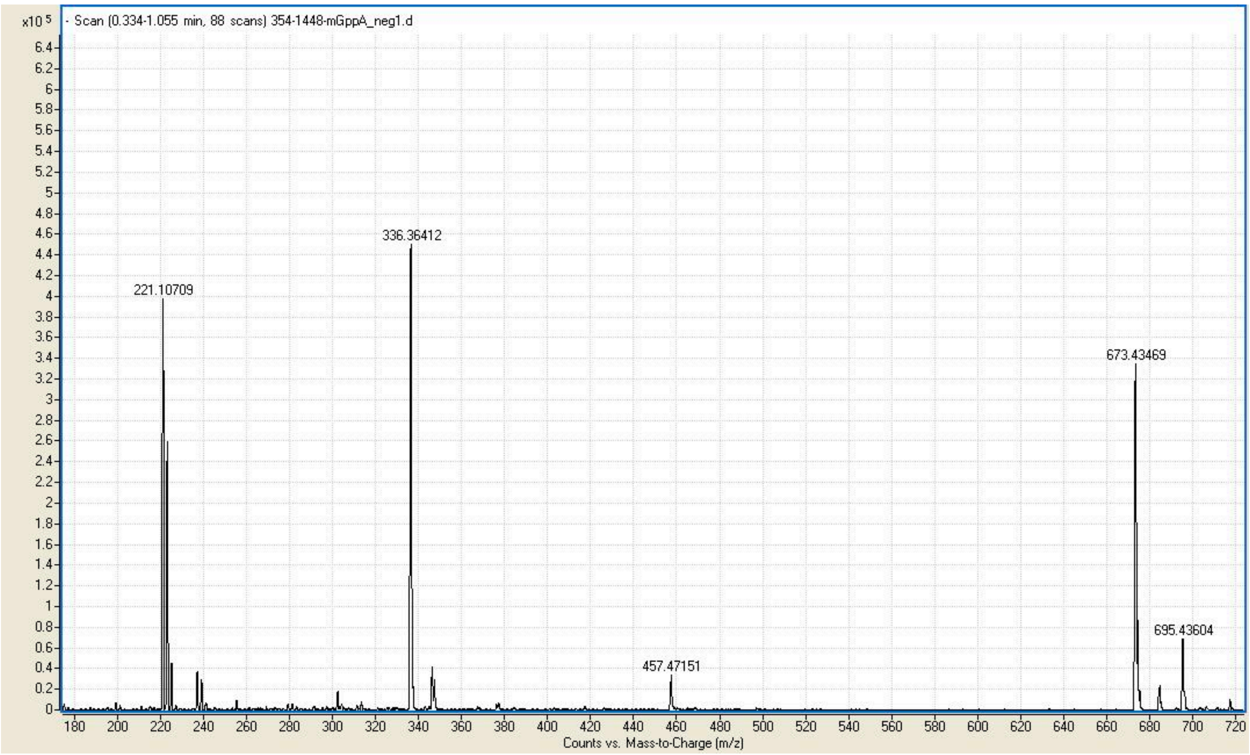

Adenosine-5’-*O*-{β-[6’-(cytosine-1-yl)-morpholine-2’-*NH*-methyl]pyrophosphate} (10C)

MS ESI (*m/z*): $[M-H]^-$ calcd for $C_{19}H_{27}N_{10}O_{11}P_2^-$ 633.134; found 633.432.

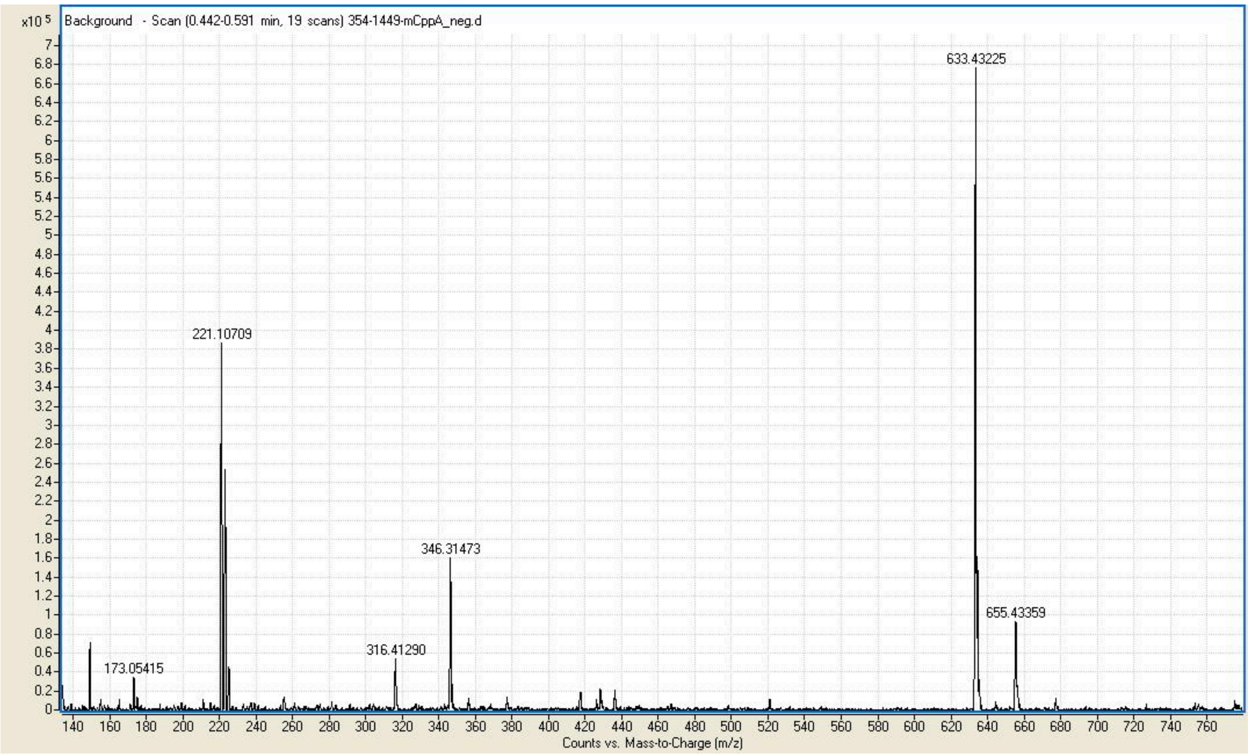

Adenosine-5'-*O*-{β-[6'-(uracil-1-yl)-morpholine-2'-*NH*-methyl]pyrophosphate} (10U)

MS MALDI-TOF (*m/z*): $[M+H]^+$ calcd for $C_{19}H_{28}N_9O_{12}P_2^+$ 636.133; found 636.053.

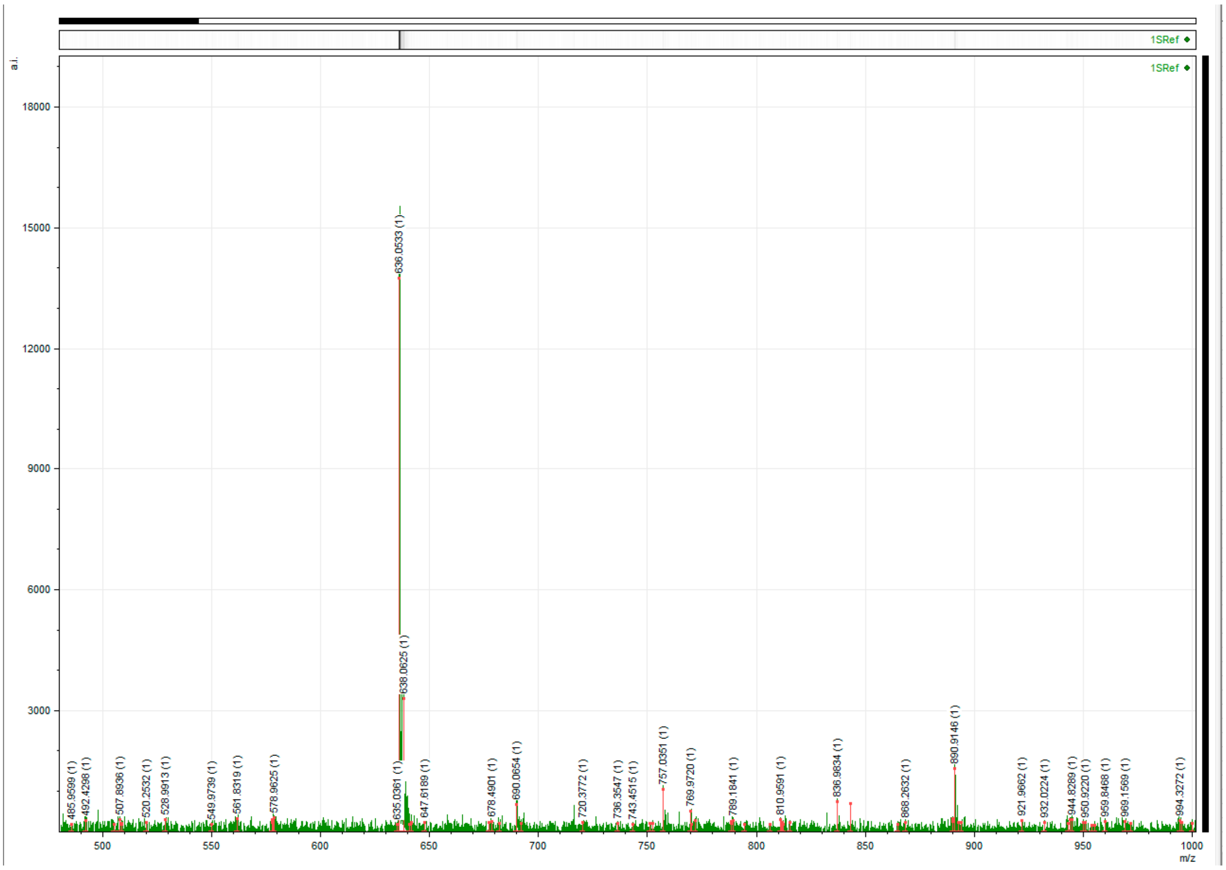

Adenosine-5’-*O*-{β-[6’-(thymine-1-yl)-morpholine-2’-*NH*-methyl]pyrophosphate} (10T)

MS MALDI-TOF (*m/z*): $[M+H]^+$ calcd for $C_{20}H_{30}N_9O_{12}P_2^+$ 650.148; found 650.654.

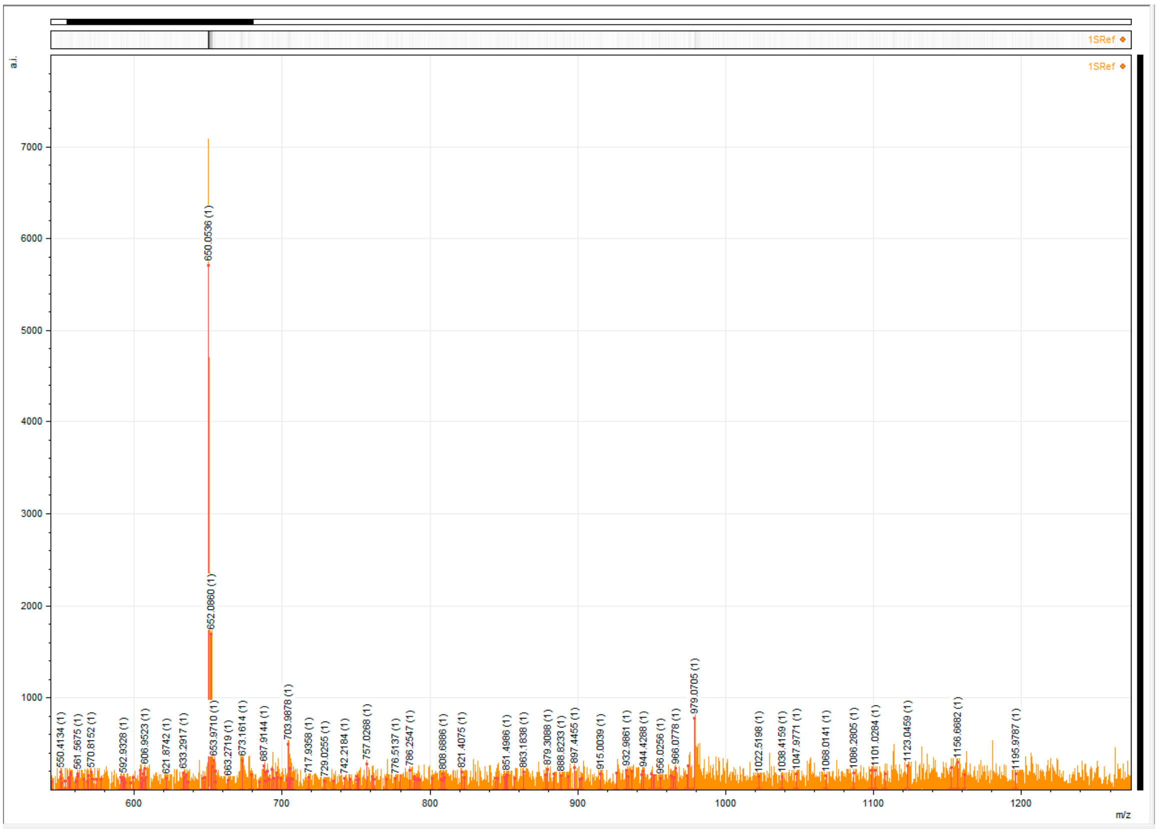

Adenosine-5’-*O*-{β-[6’-(5-iodouracil-1-yl)-morpholine-2’-*NH*-methyl]pyrophosphate} (10IU)

MS ESI (*m/z*): [M-H]$^-$ calcd for $C_{19}H_{25}IN_9O_{12}P_2^-$ 760.015; found 760.189.

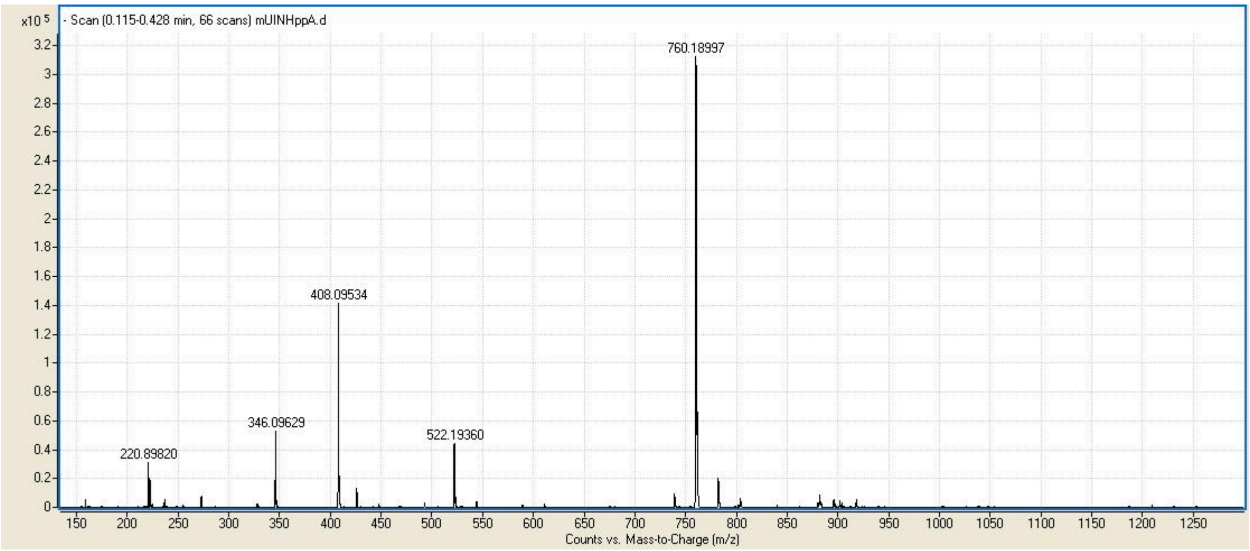

Adenosine-5'-*O*-{β-[6'-(5-bromouracil-1-yl)-morpholine-2'-*NH*-methyl]pyrophosphate} (10BrU)

MS MALDI-TOF (*m/z*): [M-H]⁻ calcd for $C_{19}H_{25}BrN_9O_{12}P_2^-$ 712.029; found 712.196; [M-3H+2Na]⁻ calcd for $C_{19}H_{23}BrN_9Na_2O_{12}P_2^-$ 755.993; found 755.232.

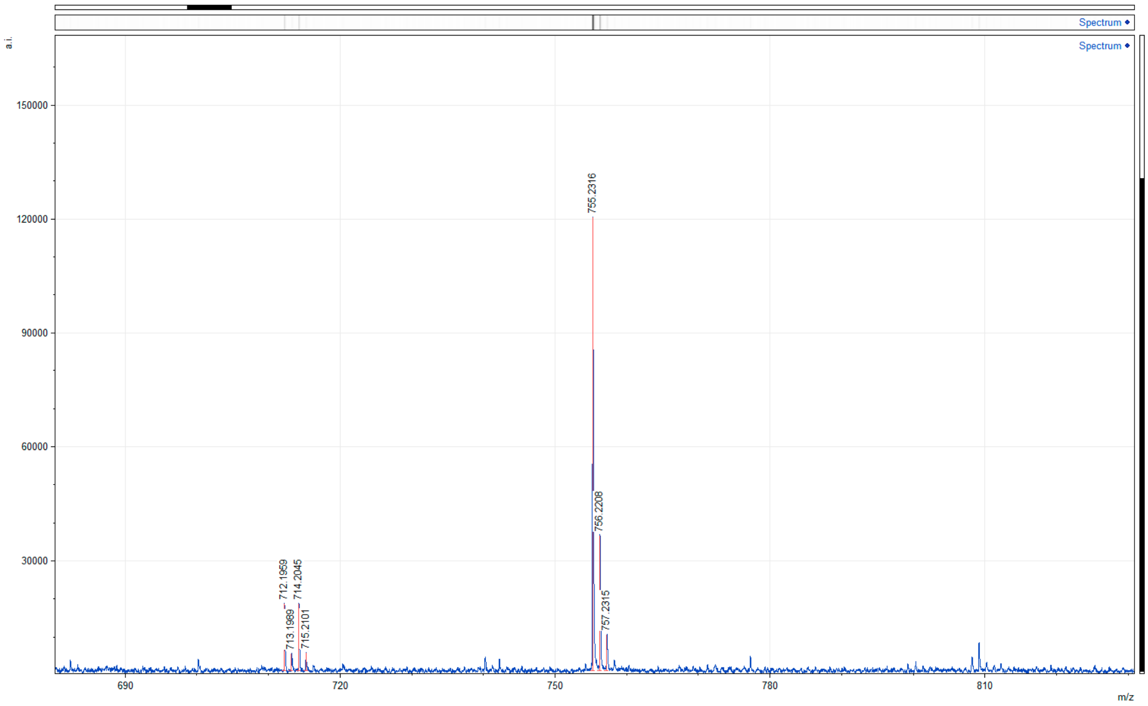

Adenosine-5'-*O*-{β-[6'-(5-chlorouracil-1-yl)-morpholine-2'-*NH*-methyl]pyrophosphate} (10ClU)

MS MALDI-TOF (*m/z*): $[M+H]^+$ calcd for $C_{19}H_{27}ClN_9O_{12}P_2^-$ 670.09; found 670.04.

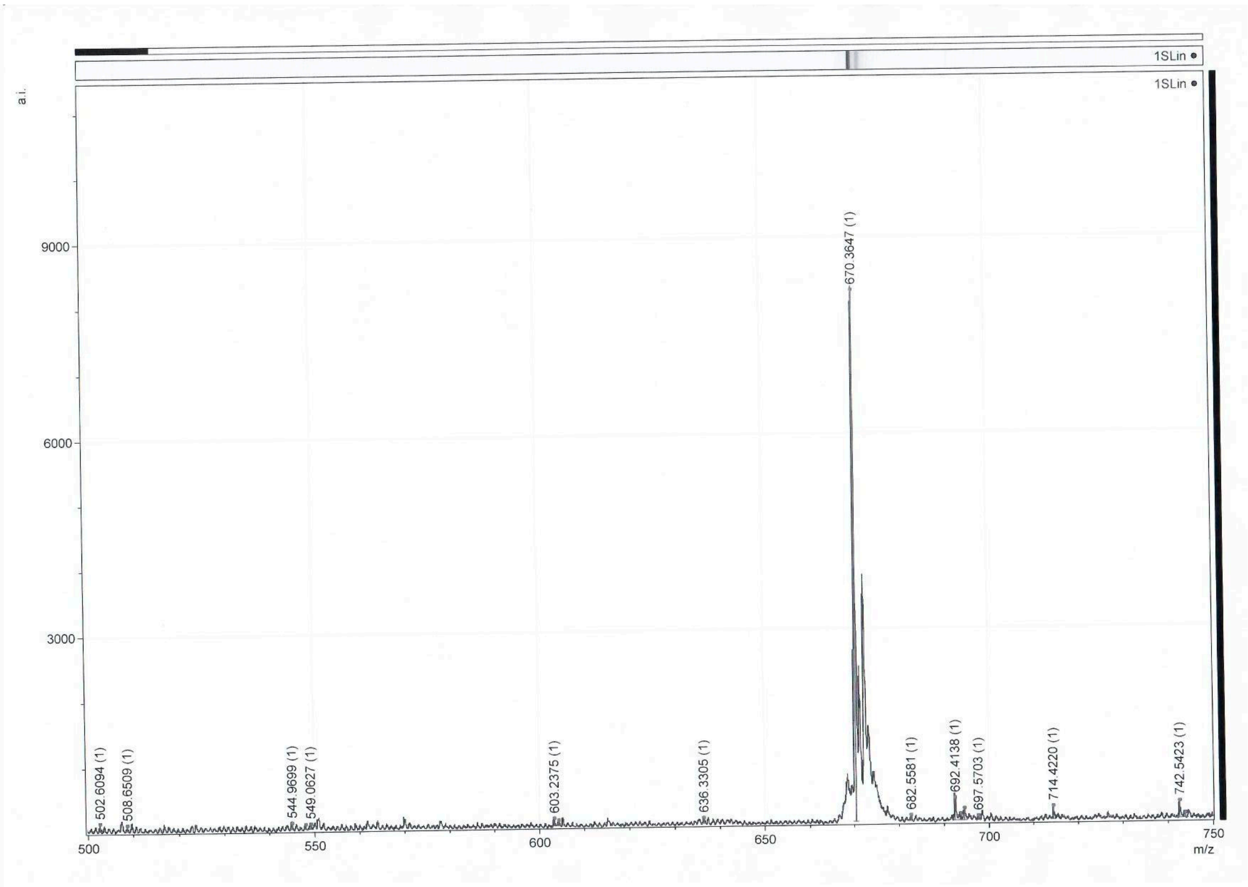

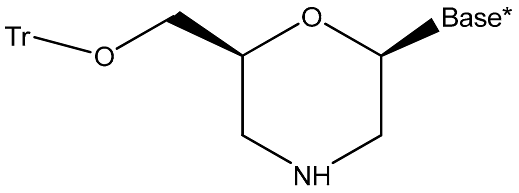

8U

2'-(Trityl-*O*-methyl)-6'-(uracil-1-yl)-morpholine (8U)

^{1}H (500 MHz, DMSO-d6): 7.62 (d, *J* 8.3, 1H, *H6*-Ura), 7.34 (dt, *J* 7.1, 1.5, 6H, Tr), 7.29 (tt, *J* 7.4, 1.5, 6H, Tr), 7.23 (tt, *J* 7.2, 2.3, 3H, Tr), 5.65 (d, *J* 7.9, 1H, *H5*-Ura), 5.57 (dd, *J* 10.0, 2.7, 1H, *H6'*), 3.93-3.87 (m, 1H, *H2'*), 3.04 (dd, *J* 9.9, 5.2, 1H, CH_2-OTr), 2.89 (dd, *J* 9.7, 5.0, 1H, *CH_2*-OTr), 2.86-2.79 (m, 2H, *H3', H5'*), 2.59 (br t, *J* 11.5, 1H, *H3'*), 2.42 (dd, *J* 11.8, 1H, *H5'*).

^{13}C (125 MHz, DMSO-d6): 163.52, 150.38, 143.80, 141.07, 128.49, 128.22, 127.44, 102.02, 86.31, 80.00, 77.09, 64.83, 48.18, 46.58.

MS ESI (*m/z*): $[M\text{-}H]^-$ calcd for $C_{28}H_{26}N_3O_4^-$ 468.19; found 468.19.

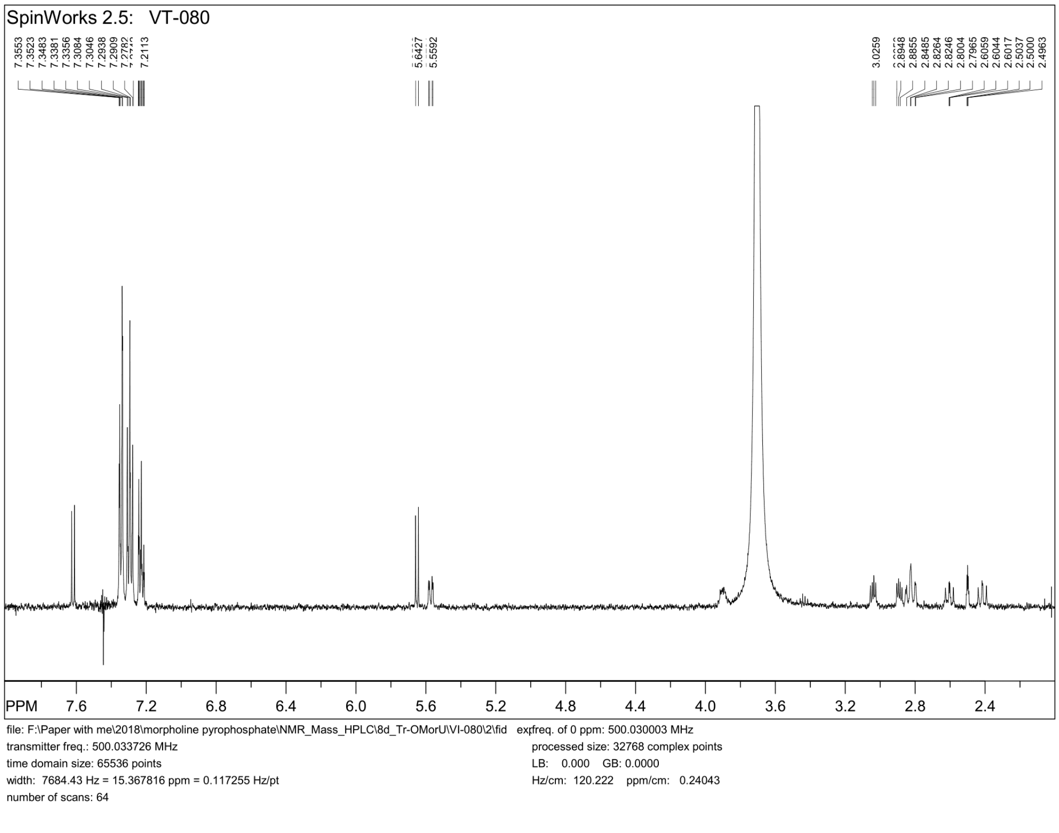

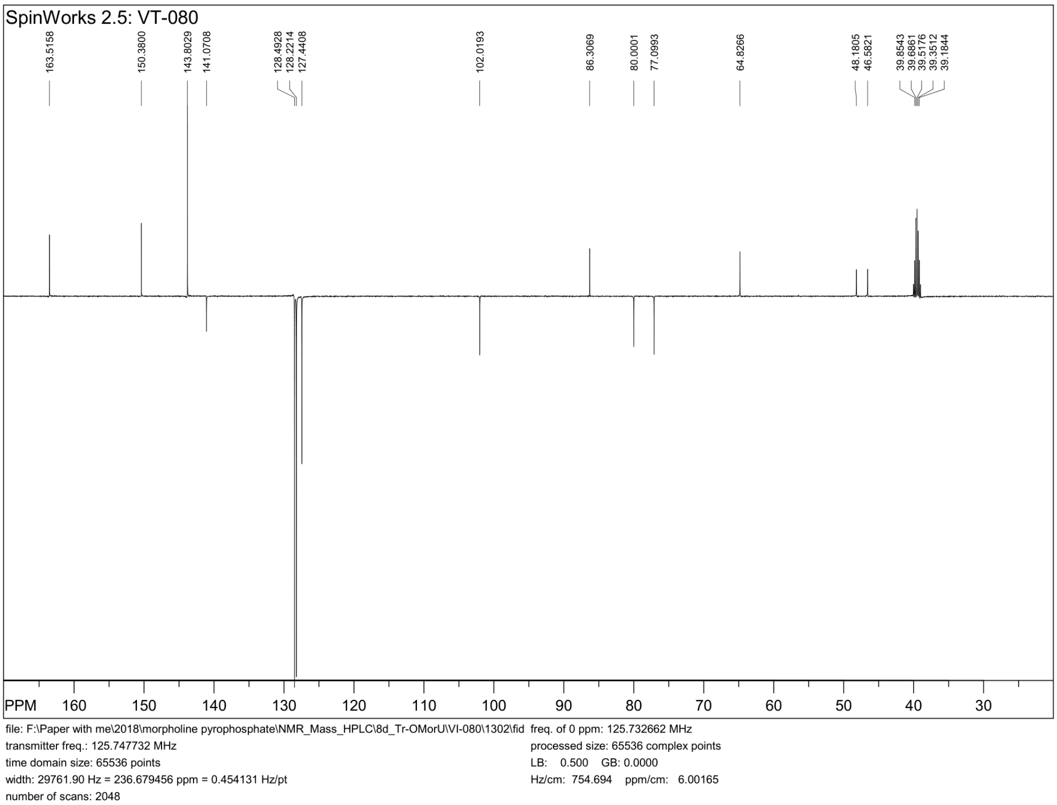
SpinWorks 2.5: VT-080
163.5158
150.3800
143.8029
141.0708
128.4928
128.2214
127.4408
102.0193
86.3069
80.0001
77.0993
64.8266
48.1805
46.5821
39.8543
39.6861
39.5176
39.3512
39.1844
PPM 160 150 140 130 120 110 100 90 80 70 60 50 40 30
file: F:\Paper with me\2018\morpholine pyrophosphate\NMR_Mass_HPLC\8d_Tr-OMorU\VI-080\1302\fid
transmitter freq.: 125.747732 MHz
time domain size: 65536 points
width: 29761.90 Hz = 236.679456 ppm = 0.454131 Hz/pt
number of scans: 2048
freq. of 0 ppm: 125.732662 MHz
processed size: 65536 complex points
LB: 0.500 GB: 0.0000
Hz/cm: 754.694 ppm/cm: 6.00165

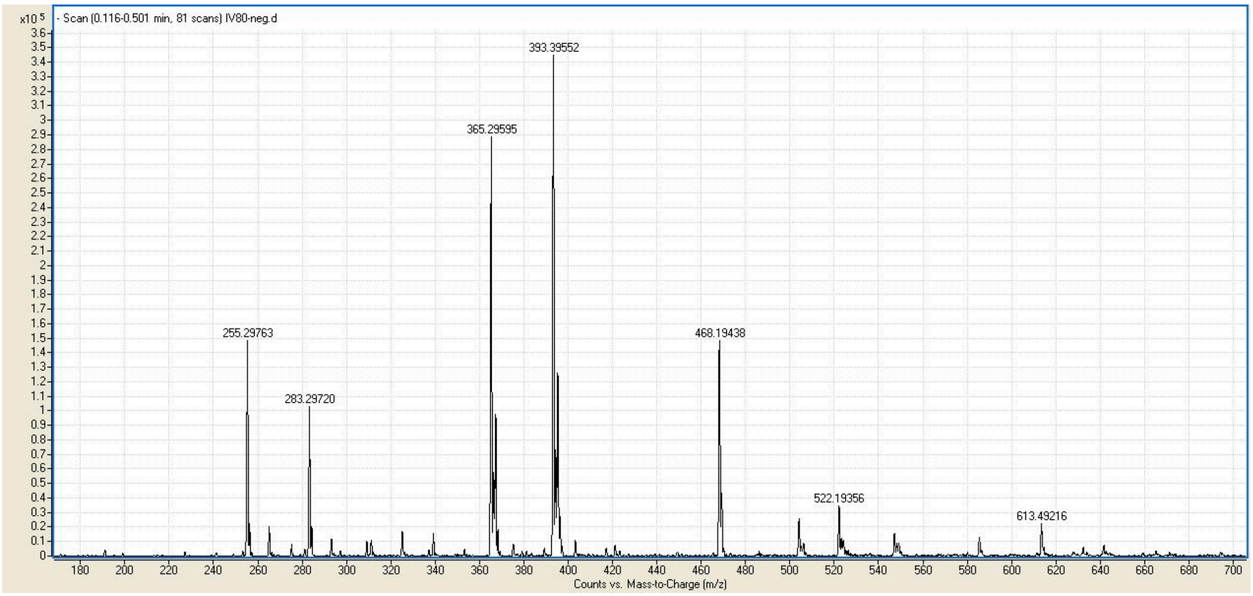
x10 5
- Scan (0.116-0.501 min, 81 scans) IV80-neg.d
393.39552
365.29595
255.29763
283.29720
468.19438
522.19356
613.49216
Counts vs. Mass-to-Charge (m/z)

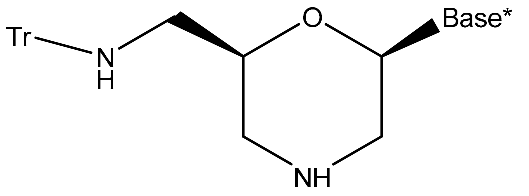

9U

2'-(trityl-*NH*-methyl)-6'-(uracil-1-yl)morpholine (9U)

^{1}H (500 MHz, DMSO-d$_6$): 7.50 (d, *J* 8.1, 1H, *H6*-Ura), 7.47-7.15 (m, 15H, Tr), 6.01 (dd, *J* 9.5, 1.5, 1H, *H6'*), 5.76 (s, 1H, CH$_2$-*NH*Tr), 5.52 (d, *J* 8.1, 1H, *H5*-Ura), 4.34-4.23 (m, 1H, *H2'*), 3.18 (br d, *J* 11.2, 1H, *CH$_2$*-NHTr), 3.04 (br d, *J* 11.2, 1H, *CH$_2$*-NHTr), 2.88-2.80 (m, 1H, *H3'*), 2.78-2.68 (m, 1H, *H5'*), 1.33 (br t, *J* 10.5, 2H, *H3'*, *H5'*).

^{13}C (125 MHz, DMSO-d$_6$): 163.00, 149.84, 140.65, 128.87, 127.90, 126.39, 101.56, 79.91, 76.29, 74.67, 54.96, 51.49.18, 49.38, 41.87.

MS MALDI-TOF (*m/z*): $[M+Na]^+$ calcd for $C_{28}H_{28}N_4NaO_3^-$ 491.21; found 490.90.

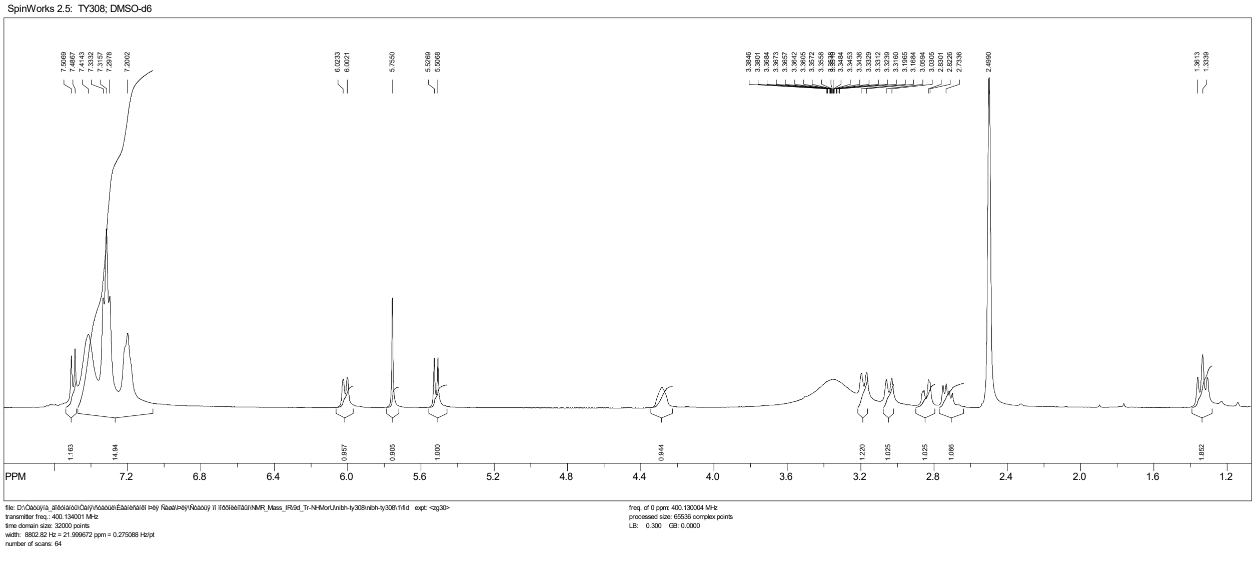

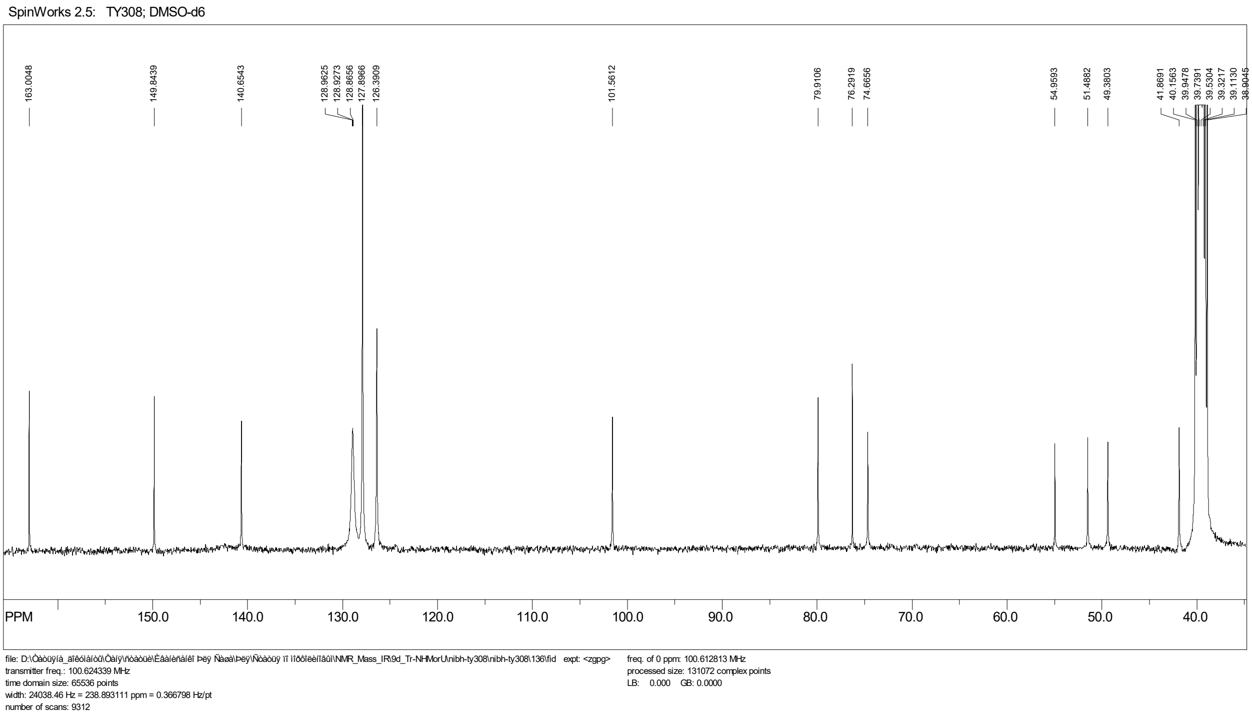
SpinWorks 2.5: TY308; DMSO-d6
163.0048
149.8439
140.6543
128.9625
128.9273
128.8656
127.8966
126.3909
101.5612
79.9106
76.2919
74.6656
54.9593
51.4882
49.3803
41.8691
40.1563
39.9478
39.7391
39.5304
39.3217
39.1130
38.9045
PPM
150.0
140.0
130.0
120.0
110.0
100.0
90.0
80.0
70.0
60.0
50.0
40.0
file: D:\Òàòüÿíà_äîêóìåíòû\Òàíÿ\ñòàòüè\Èâàíèñåíêî Þëÿ Ñàøà\Þëÿ\Ñòàòüÿ ïî ìîðôîëèíîâûì\NMR_Mass_IR\9d_Tr-NHMorU\nibh-ty308\nibh-ty308\136\fid expt: <zgpg>
transmitter freq.: 100.624339 MHz
time domain size: 65536 points
width: 24038.46 Hz = 238.893111 ppm = 0.366798 Hz/pt
number of scans: 9312
freq. of 0 ppm: 100.612813 MHz
processed size: 131072 complex points
LB: 0.000 GB: 0.0000

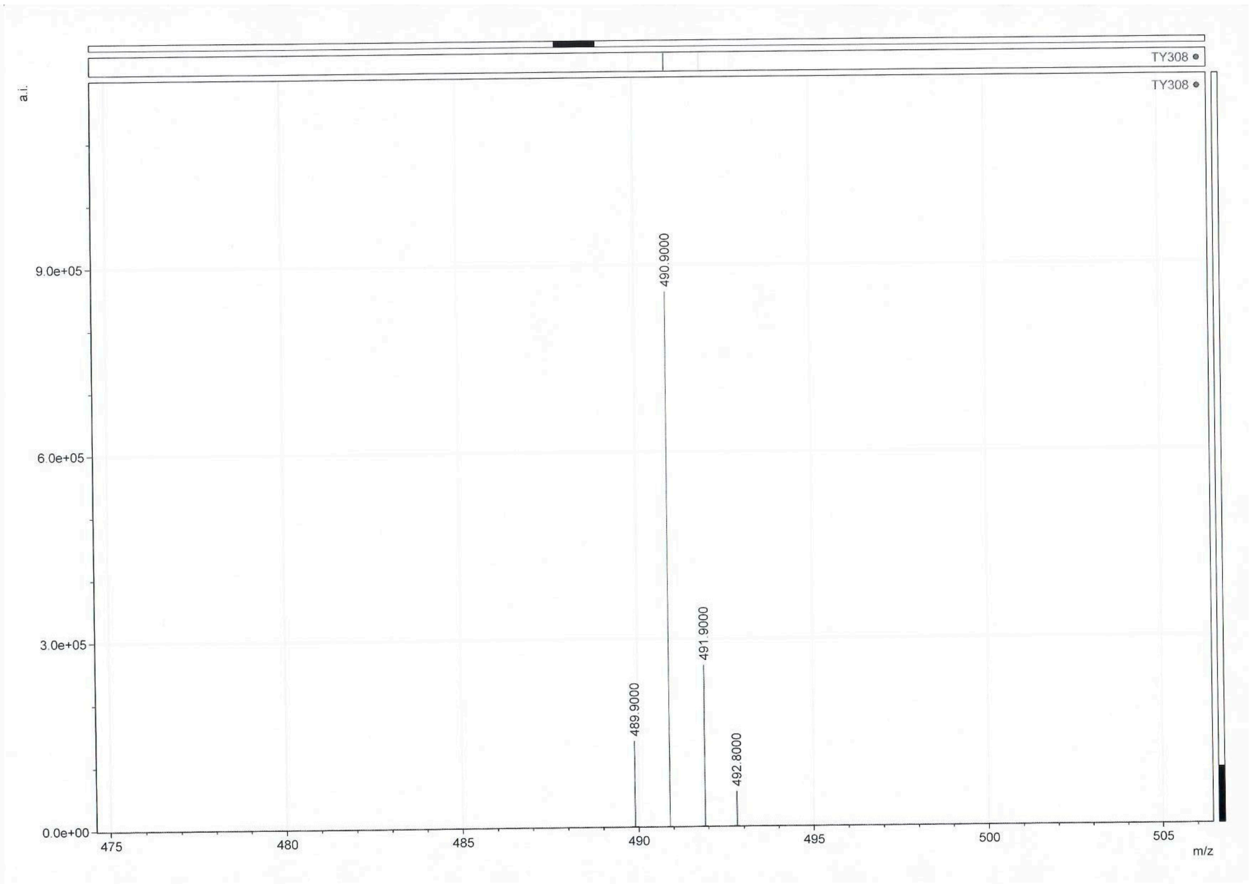
TY308
TY308
a.i.
9.0e+05
6.0e+05
3.0e+05
0.0e+00
490.9000
491.9000
489.9000
492.8000
475
480
485
490
495
500
505
m/z

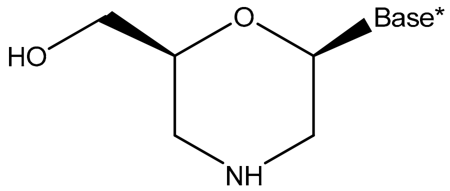

11A,G,C,U,T,IU,BrU,ClU

2'-Hydroxymethyl-6'-(adenine-9-yl)-morpholine 11A

^{1}H (300 MHz, DMSO-d6): 8.28 (s, 1H, *H8*-Ade), 8.12 (s, 1H, *H2*-Ade), 7.21 (s, 2H, *NH2*-Ade), 5.70 (dd, 1H, *J* 10.4, 2.3, *H6'*), 3.76-3.70 (m, 1H, *H4'*), 3.40-3.31 (m, 2H, *CH2*-OH), 3.19 (dd, *J* 12.0, 10.4, 1H, *H5'*), 3.03 (dd, *J* 12.1, 1.9, 1H, *H3'*), 2.87 (dd, *J* 12.1, 1.9, 1H, *H3'*), 2.46 (m, 1H, *H5'*) 1.87 (s, 1.5H, *CH3*-Ac).

^{13}C (125 MHz, DMSO-d6): 171.9, 155.98, 152.65, 148.97, 138.76, 118.45, 79.24, 78.32, 62.01, 48.20, 46.07, 20.98.

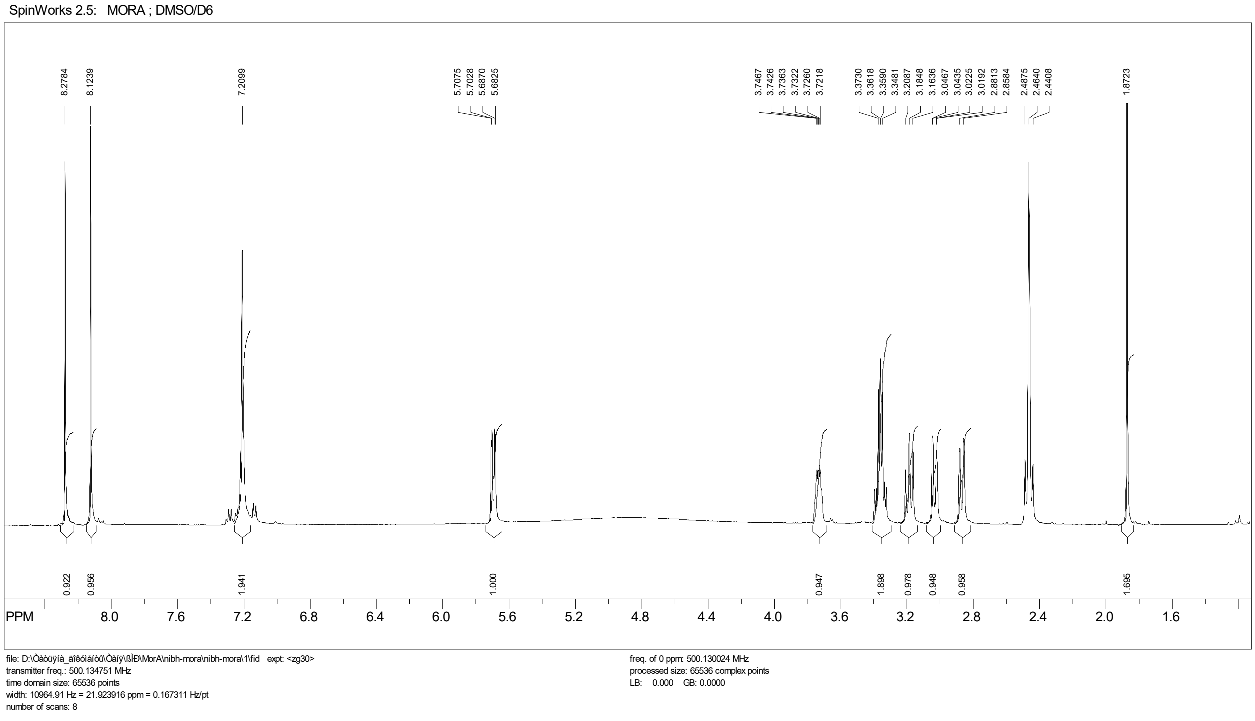

SpinWorks 2.5: MORA ; DMSO/D6

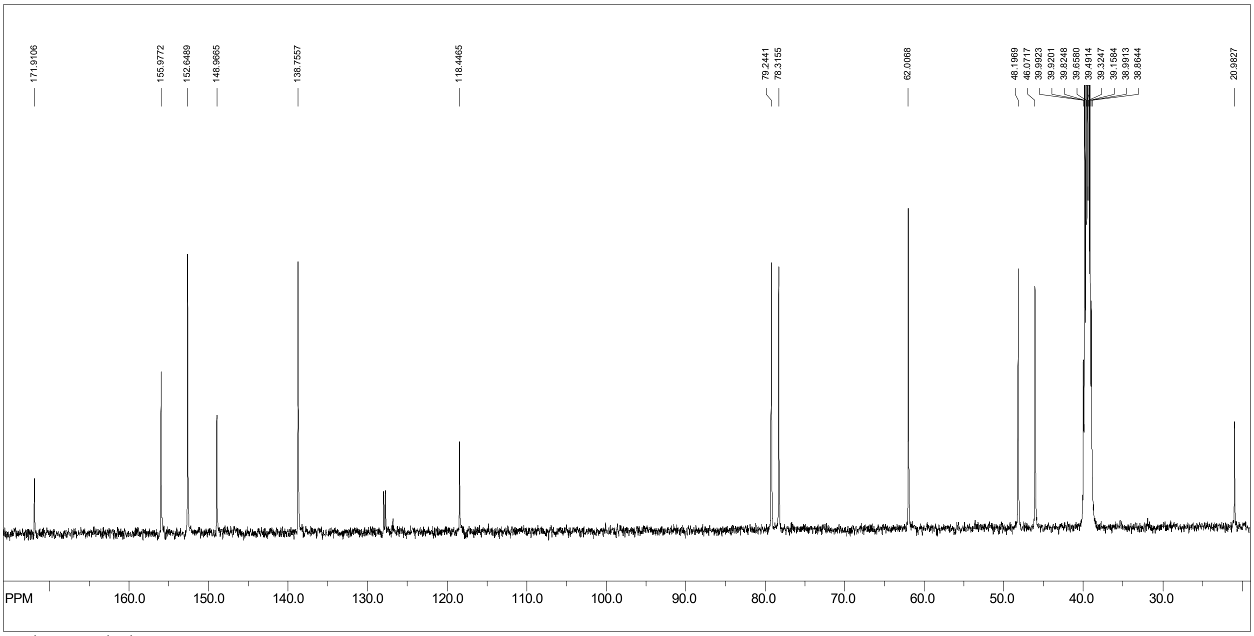

file: D:\Òàòüÿíà_äîêóìåíòû\Òàíÿ\ßÌÐ\MorA\nibh-mora\nibh-mora\136\fid expt: <zgpg>
transmitter freq.: 125.772075 MHz
time domain size: 65536 points
width: 30303.03 Hz = 240.936076 ppm = 0.462388 Hz/pt
number of scans: 496

freq. of 0 ppm: 125.757859 MHz
processed size: 131072 complex points
LB: 0.000 GB: 0.0000

2'-Hydroxymethyl-6'-(guanine-9-yl)-morpholine 11G

^{1}H (500 MHz, DMSO-d$_6$): 7.84 (s, 1H, *H8*-Gua), 6.61 (s, 2H, *NH$_2$*-Gua), 5.45 (dd, 1H, *J* 10.2, 2.7, *H6'*), 3.68-3.61 (m, 1H, *H4'*), 3.42-3.32 (m, 2H, *CH$_2$*-OH), 3.03 (dd, *J* 12.0, 10.4, 1H, *H5'*), 2.95 (dd, *J* 12.1, 2.2, 1H, *H3'*), 2.83 (dd, *J* 12.1, 2.2, 1H, *H3'*), 2.42 (dd, *J* 12.2, 11.0, 1H, *H5'*),) 1.85 (s, 2H, *CH$_3$*-Ac).

^{13}C (125 MHz, DMSO-d$_6$): 175.68, 158.74, 155.77, 152.58, 136.78, 118.10, 80.94, 80.58, 63.99, 50.52, 48.24, 24.20.

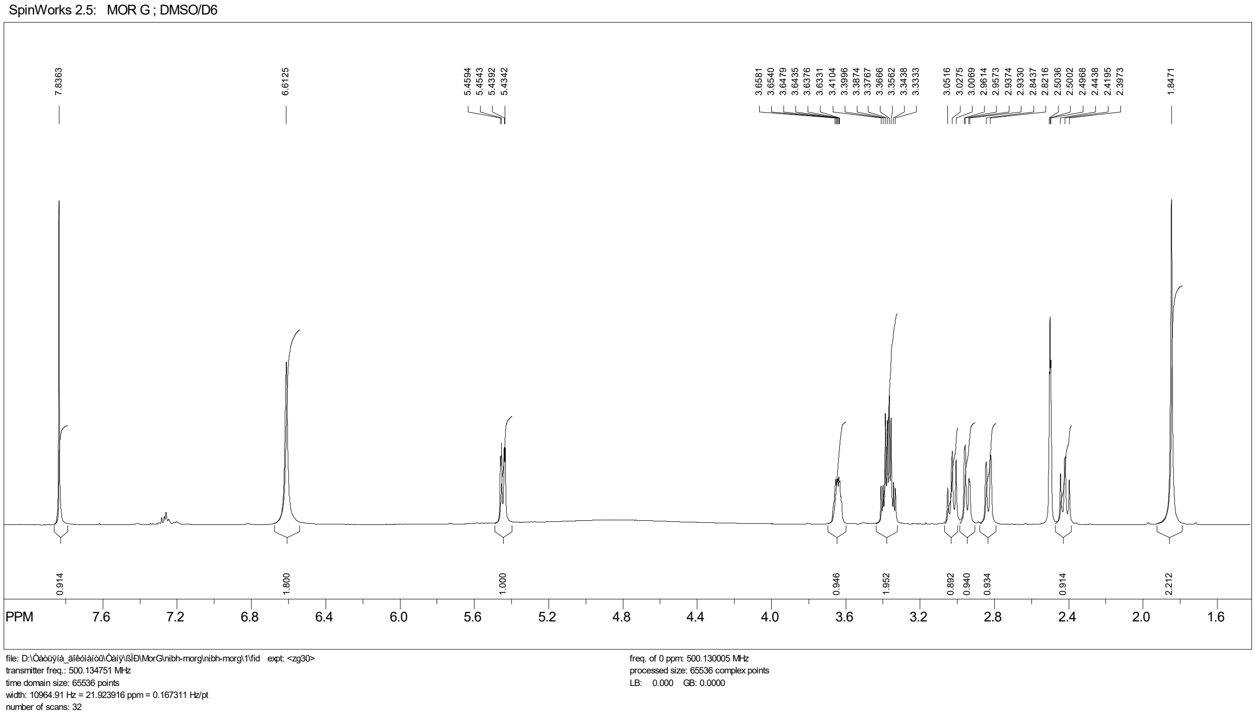

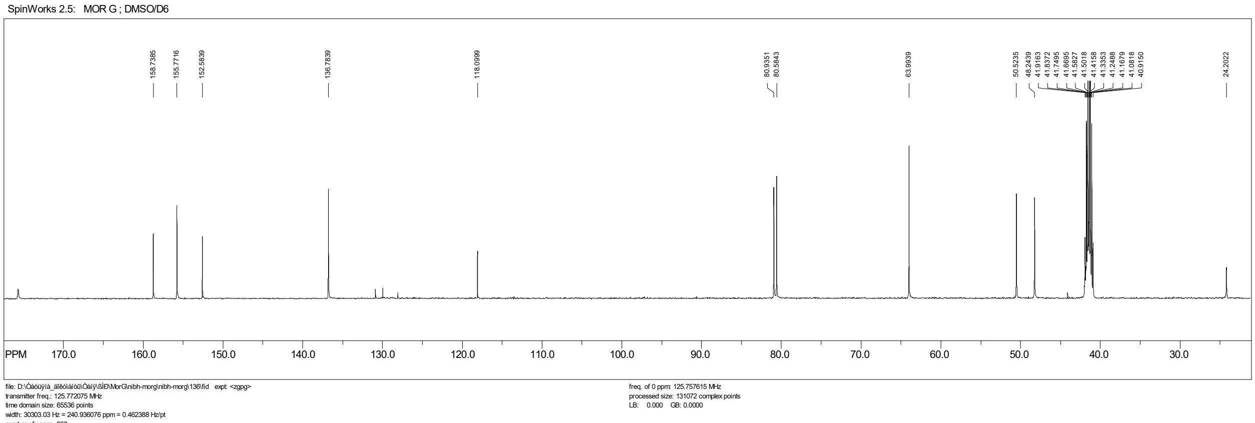

2’-Hydroxymethyl-6’-(cytozine-1-yl)-morpholine 11C

^{1}H (500 MHz, DMSO-d_6): 7.58 (d, *J* 7.4, 1H, *H6*-Cyt), 7.16 (br s, 2H, *NH_2*-Cyt), 5.72 (d, *J* 7.4, 1H, *H5*-Cyt), 5.58 (dd, 1H, *J* 9.7, 2.3, *H6’*), 3.67-3.60 (m, 1H, *H4’*), 3.43-3.33 (m, 2H, *CH_2*-OH), 2.85-2.75 (m, 2H, *H5’*,*H3’*), 2.43 (dd, *J* 11.7, 10.2, 1H, *H3’*), 2.35 (dd, *J* 12.0, 11.2, 1H, *H5’*),) 1.90 (s, 2H, *CH_3*-Ac).

^{13}C (125 MHz, DMSO-d_6): 172.05, 165.42, 154.41, 141.21, 93.72, 80.38, 78.69, 62.17, 48.89, 46.23, 21.10.

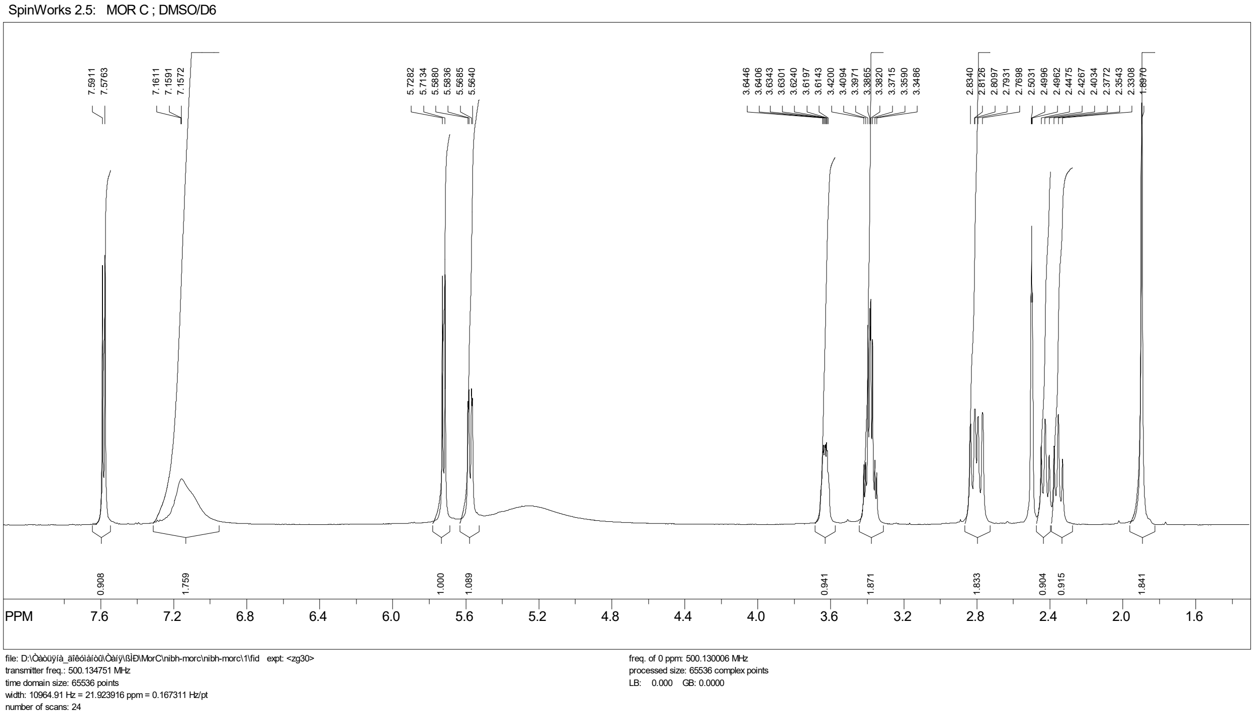

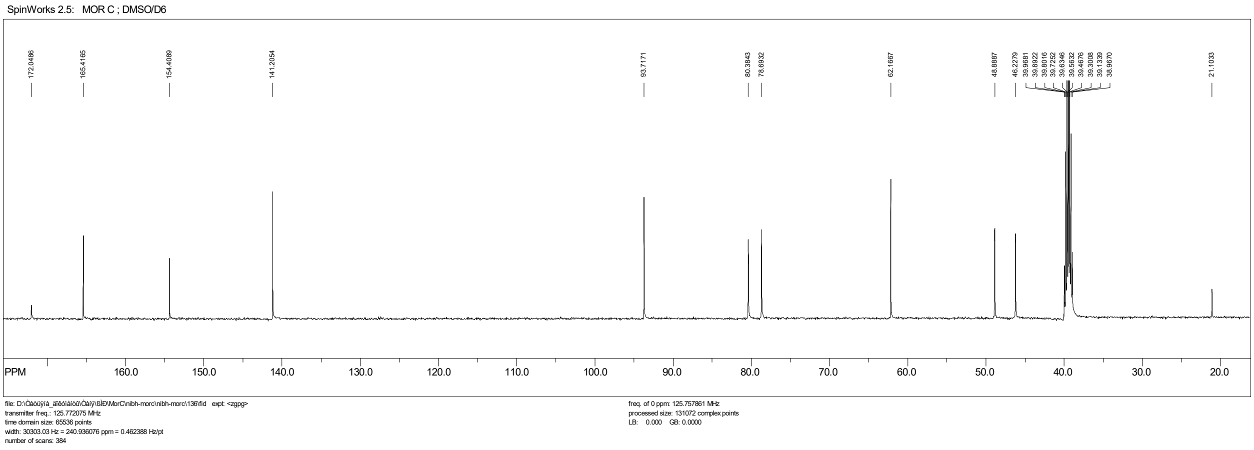

2’-Hydroxymethyl-6’-(uracil-1-yl)-morpholine 11U

^{1}H (500 MHz, DMSO-d$_6$): 7.63 (d, *J* 8.1, 1H, *H6*-Ura), 5.57 (d, *J* 8.1, 1H, *H5*-Ura), 5.49 (dd, 1H, *J* 10.0, 2.5, *H6’*), 3.65-3.59 (m, 1H, *H4’*), 3.39-3.31 (m, 2H, *CH$_2$*-OH), 2.80 (dd, *J* 12.3, 2.1, 1H, *H5’*), 2.73 (dd, *J* 12.5, 1.5, 1H, *H3’*), 2.55 (dd, *J* 11.9, 10.2, 1H, *H3’*), 2.34 (dd, *J* 12.4, 10.8, 1H, *H5’*),) 1.86 (s, 2H, *CH$_3$*-Ac).

^{13}C (125 MHz, DMSO-d$_6$): 172.02, 162.89, 150.03, 140.89, 101.50, 79.74, 78.97, 62.08, 48.12, 46.02, 21.07.

SpinWorks 2.5: MORU; DMSO/D6

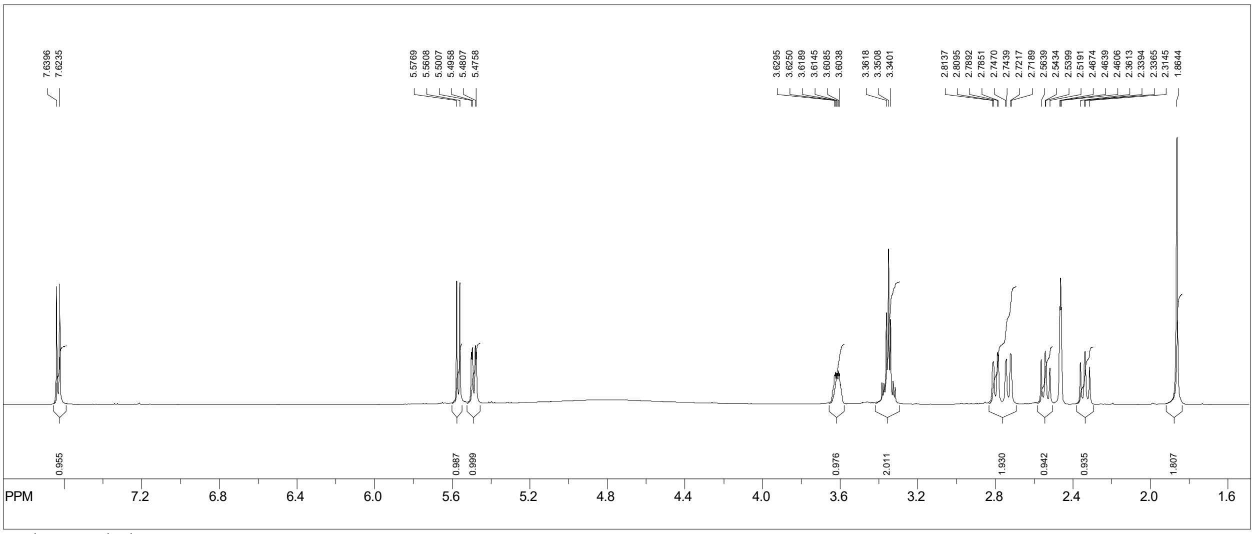

file: D:\Ðàáîòû\à_ãîëóáåâà\ÐàìÿàÖÐ\MorU\nibh-moru\nibh-moru\1\fid expt: <zg30>
transmitter freq.: 500.134751 MHz
time domain size: 65536 points
width: 10964.91 Hz = 21.923916 ppm = 0.167311 Hz/pt
number of scans: 32

freq. of 0 ppm: 500.130024 MHz
processed size: 65536 complex points
LB: 0.000 GB: 0.0000

SpinWorks 2.5: MORU; DMSO/D6

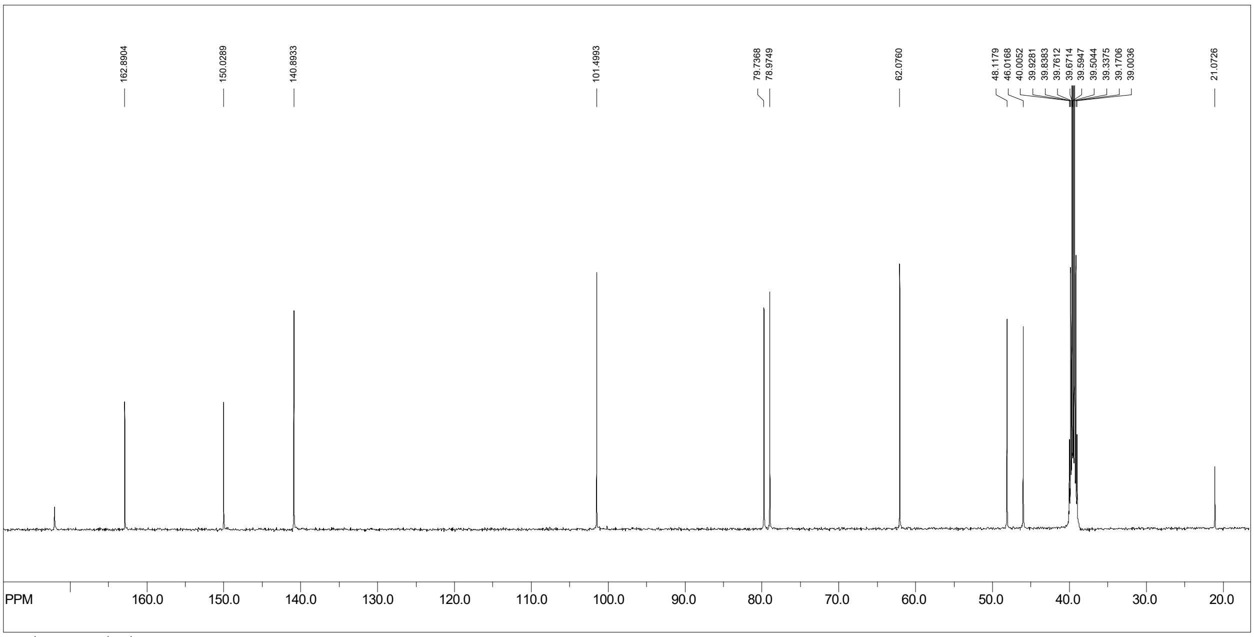

file: D:\Ðàáîòû\à_ãîëóáåâà\ÐàìÿàÖÐ\MorU\nibh-moru\nibh-moru\136\fid expt: <zgpg>
transmitter freq.: 125.772075 MHz
time domain size: 65536 points
width: 30303.03 Hz = 240.936076 ppm = 0.462388 Hz/pt
number of scans: 624

freq. of 0 ppm: 125.757855 MHz
processed size: 131072 complex points
LB: 0.000 GB: 0.0000

2’-Hydroxymethyl-6’-(thymine-1-yl)-morpholine 11T

^{1}H (500 MHz, DMSO-d$_6$): 7.55 (q, *J* 1.1, 1H, *H6*-Thy), 5.58 (dd, 1H, *J* 9.9, 2.3, *H6’*), 3.71-3.66 (m, 1H, *H4’*), 3.44-3.36 (m, 2H, *CH$_2$*-OH), 2.88-2.80 (m, 2H, *H5’,H3’*), 2.71 (dd, *J* 12.0, 10.5, 1H, *H3’*), 2.44 (dd, *J* 12.4, 11.4, 1H, *H5’*),) 1.91 (s, 1.5H, *CH$_3$*-Ac), 1.78 (d, *J* 1.1, 3H, *CH$_3$*-Thy).

^{13}C (125 MHz, DMSO-d$_6$): 171.87, 163.51, 149.99, 136.30, 109.20, 79.12, 78.39, 61.94, 47.35, 45.60, 20.95, 11.86.

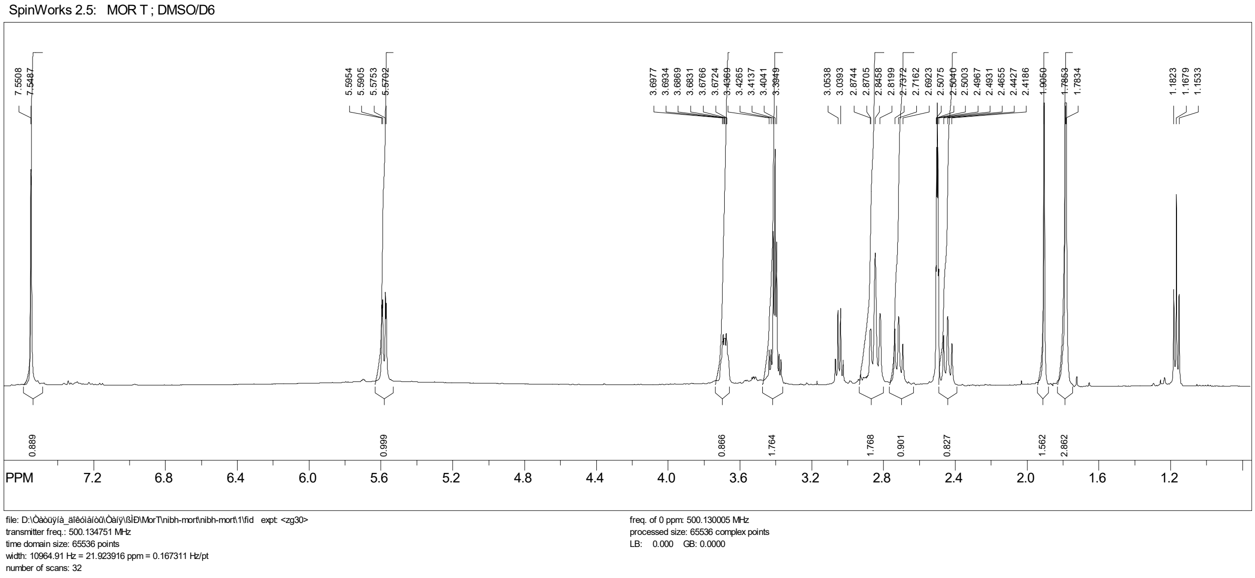

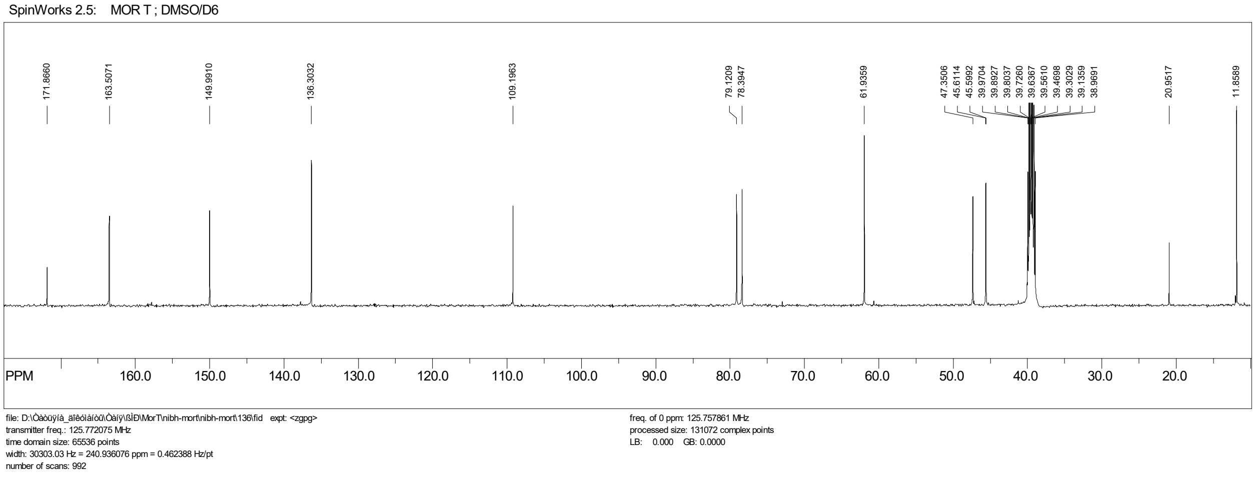

2’-Hydroxymethyl-6’-(5-iodouracil-1-yl)-morpholine 11IU

^{1}H (500 MHz, DMSO-d_6): 8.01 (s, 1H, *H6*-Ura), 5.57 (dd, 1H, *J* 10.1, 2.5, *H6’*), 3.74-3.68 (m, 1H, *H4’*), 3.43 (d, *J* 5.0, 2H, *CH_2*-OH), 2.91 (dd, *J* 12.4, 2.4, 1H, *H5’,*), 2.83 (dd, *J* 12.8, 2.0, 1H, *H3’*), 2.71 (dd, *J* 12.4, 10.2, 1H, *H3’*), 2.54 (dd, *J* 12.5, 11.1, 1H, *H5’*), 1.90 (s, 1.5H, *CH_3*-Ac).

^{13}C (125 MHz, DMSO-d_6): 171.87, 160.22, 149.64, 144.78, 79.47, 78.37, 69.63, 61.83, 47.28, 45.66, 45.13, 20.98.

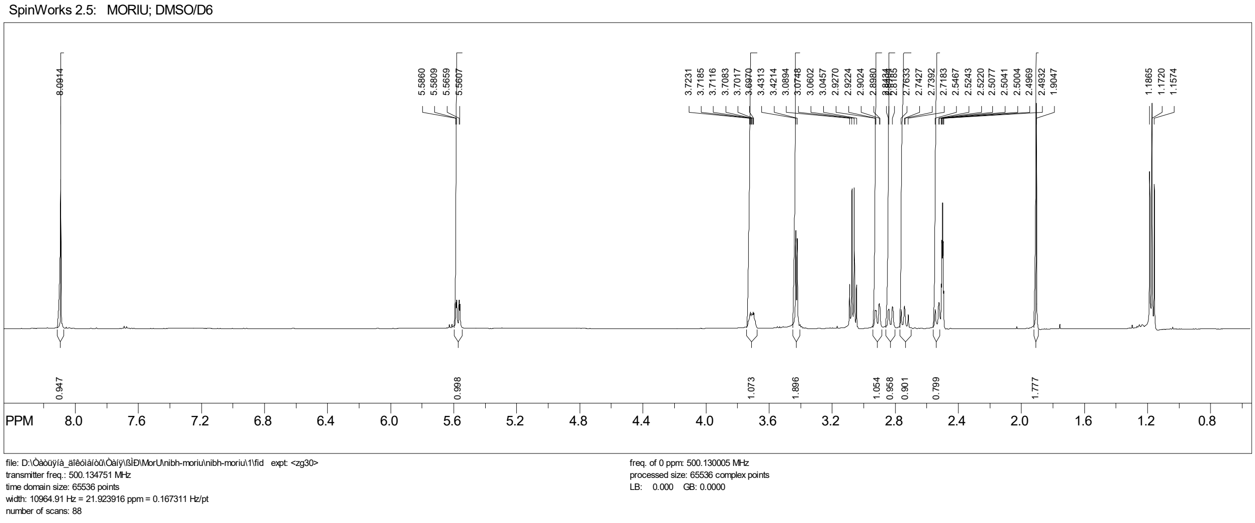

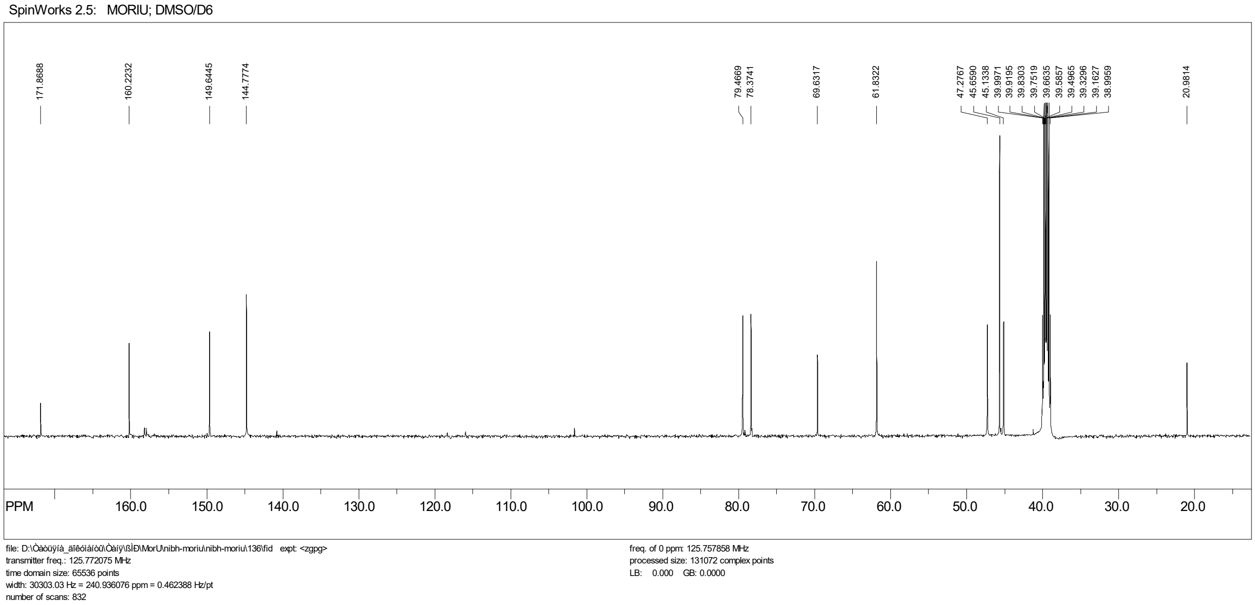

2’-Hydroxymethyl-6’-(5-bromouracil-1-yl)-morpholine 11BrU

^{1}H (500 MHz, DMSO-d$_6$): 8.12 (s, 1H, *H6*-Ura), 5.56 (dd, 1H, *J* 10.1, 2.6, *H6’*), 3.73-3.67 (m, 1H, *H4’*), 3.42 (d, *J* 5.0, 2H, *CH$_2$*-OH), 2.90 (dd, *J* 12.4, 2.5, 1H, *H5’,*), 2.80 (dd, *J* 12.5, 1.8, 1H, *H3’*), 2.74 (dd, *J* 12.2, 10.2, 1H, *H3’*), 2.50 (m, *H5’*),) 1.90 (s, 2H, *CH$_3$*-Ac).

^{13}C (125 MHz, DMSO-d$_6$): 171.88, 158.89, 149.29, 140.32, 95.88, 79.75, 78.56, 61.88, 47.45, 45.66, 45.27, 20.97.

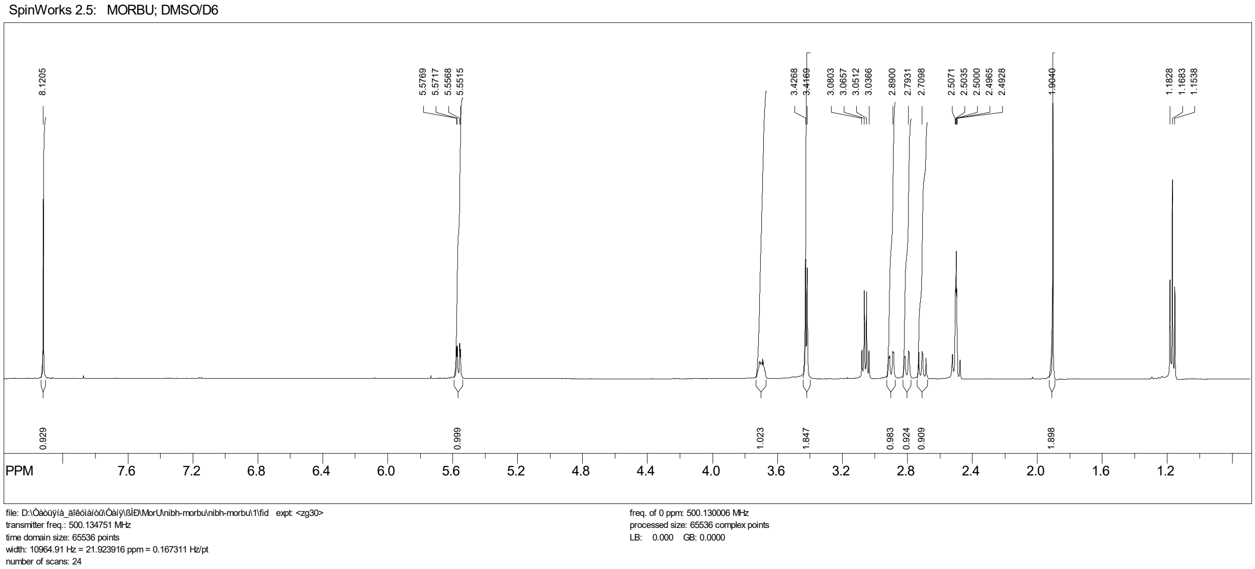

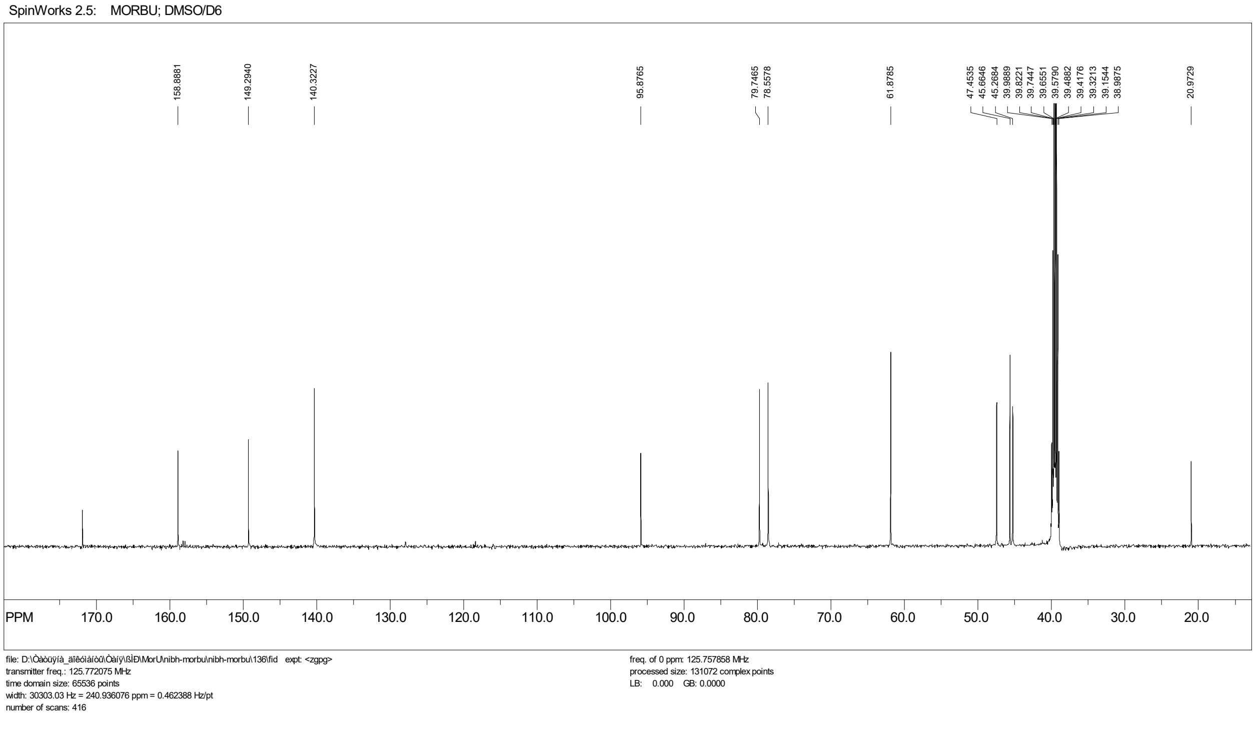

2’-Hydroxymethyl-6’-(5-chlorouracil-1-yl)-morpholine 11ClU

^{1}H (500 MHz, DMSO-d$_6$): 8.03 (s, 1H, *H6*-Ura), 5.51 (dd, 1H, *J* 10.1, 2.7, *H6’*), 3.67-3.62 (m, 1H, *H4’*), 3.38 (d, *J* 5.0, 2H, *CH$_2$*-OH), 2.84 (dd, *J* 12.3, 2.4, 1H, *H5’,*), 2.74 (dd, *J* 12.7, 2.0, 1H, *H3’*), 2.63 (dd, *J* 12.1, 10.2, 1H, *H3’*), 2.44 (dd *J* 12.5, 11.1, *H5’*),) 1.87 (s, 0.8H, *CH$_3$*-Ac).

^{13}C (125 MHz, DMSO-d$_6$): 171.88, 158.76, 149.09, 138.03, 107.25, 79.92, 78.76, 61.94, 47.67, 45.46, 20.95.

SpinWorks 2.5: MORCU; DMSO/D6

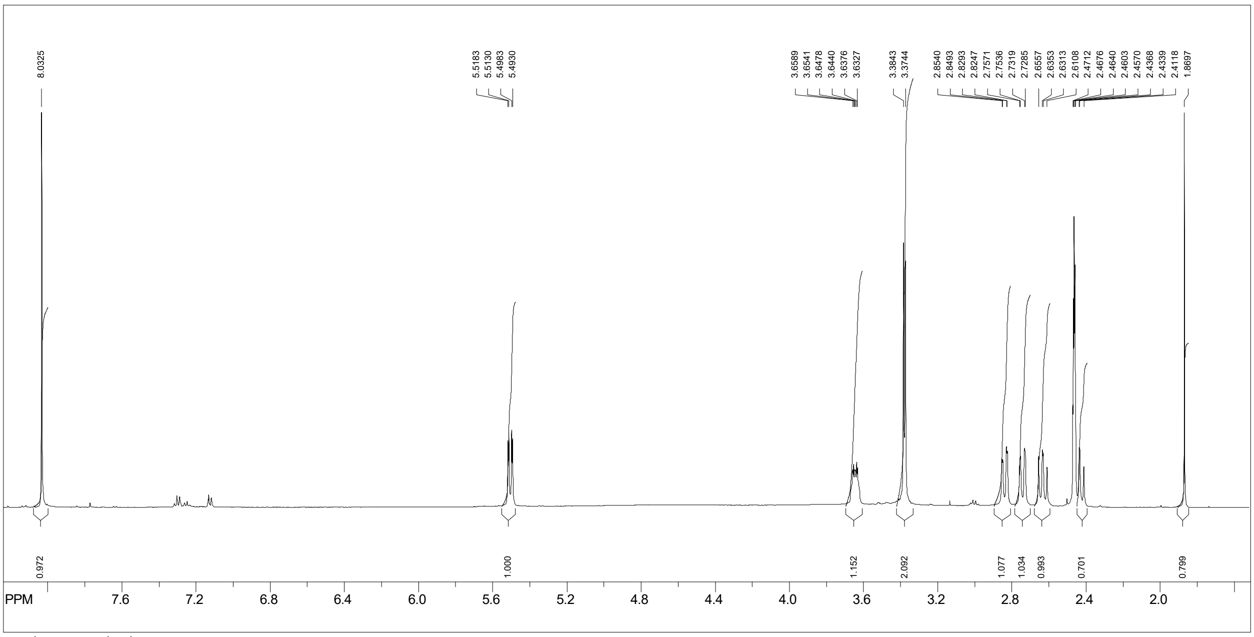

file: D:\Ôàòüÿíà_ãîëóáåâà\Ôàéÿ\ßÌÐ\MorU\nibh-morcu\nibh-morcu\1\fid expt: <zg30>
transmitter freq.: 500.134751 MHz
time domain size: 65536 points
width: 10964.91 Hz = 21.923916 ppm = 0.167311 Hz/pt
number of scans: 16

freq. of 0 ppm: 500.130024 MHz
processed size: 65536 complex points
LB: 0.000 GB: 0.0000

SpinWorks 2.5: MORCU; DMSO/D6

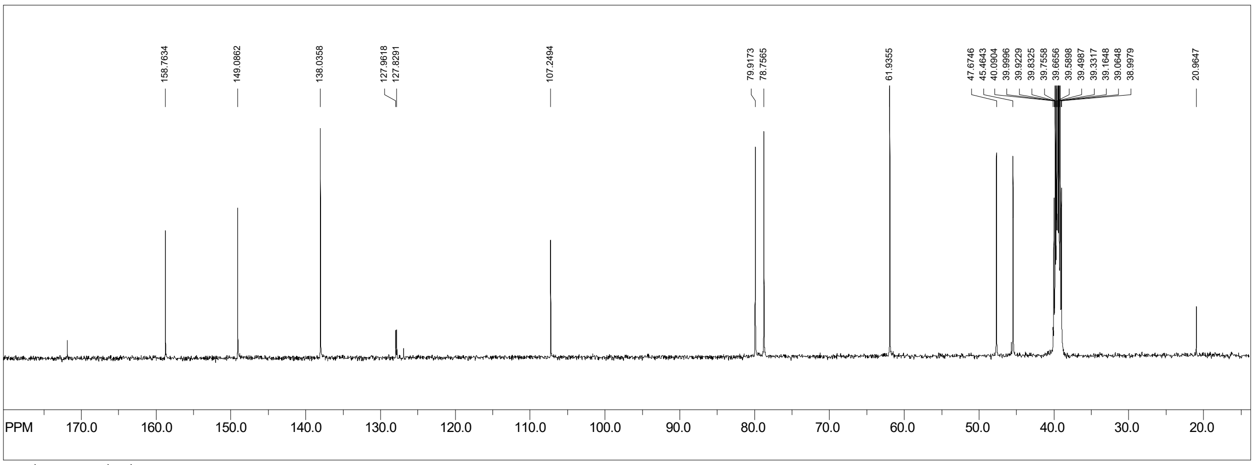

file: D:\Ôàòüÿíà_ãîëóáåâà\Ôàéÿ\ßÌÐ\MorU\nibh-morcu\nibh-morcu\136\fid expt: <zgpg>
transmitter freq.: 125.772075 MHz
time domain size: 65536 points
width: 30303.03 Hz = 240.936076 ppm = 0.462388 Hz/pt
number of scans: 784

freq. of 0 ppm: 125.757858 MHz
processed size: 131072 complex points
LB: 0.000 GB: 0.0000

Determination of the type of inhibition

Table S3. Vmax and Km values for different concentrations of **4IU, 10IU, 11IU, 3-AB**

	**4IU**		**10IU**		**11IU**		**MAB**	
Inhibitor concentration, μM	Kм, μM	Vmax, RFU/sec	Kм, μM	Vmax, RFU/sec	Kм, μM	Vmax, RFU/sec	Kм, μM	Vmax, RFU/sec
0	5.2	0.17	6.0	0.22	13	0.072	7.4	0.17
50	6.6	0.16	6.5	0.19	22	0.066	13	0.16
100	5.4	0.15	12.5	0.18	29	0.066	21	0.16
500	7.6	0.14	33.4	0.17	134	0.060	64	0.16

Table S4. Vmax and Km values for different concentrations of **10A**

**10A**, μM	Kм, μM	Vmax, RFU/sec
0	7.2	0.14
300	16	0.090
600	25	0.068
900	26	0.062
